# Discovery of a Dual SENP1 and SENP2 Inhibitor

**DOI:** 10.3390/ijms232012085

**Published:** 2022-10-11

**Authors:** Michael Brand, Elias Benjamin Bommeli, Marc Rütimann, Urs Lindenmann, Rainer Riedl

**Affiliations:** Institute of Chemistry and Biotechnology, Competence Center for Drug Discovery, Zurich University of Applied Sciences (ZHAW), Einsiedlerstrasse 31, 8820 Wädenswil, Switzerland

**Keywords:** medicinal chemistry, SENP, deubiquitinating enzymes, structure activity relationship, inhibitors, cancer, small molecules, drug discovery

## Abstract

SUMOylation is a reversible post–translational modification (PTM) involving covalent attachment of small ubiquitin-related modifier (SUMO) proteins to substrate proteins. Dysregulation of SUMOylation and deSUMOylation results in cellular malfunction and is linked to various diseases, such as cancer. Sentrin-specific proteases (SENPs) were identified for the maturation of SUMOs and the deconjugation of SUMOs from their substrate proteins. Hence, this is a promising target tackling the dysregulation of the SUMOylation process. Herein, we report the discovery of a novel protein-protein interaction (PPI) inhibitor for SENP1-SUMO1 by virtual screening and subsequent medicinal chemistry optimization of the hit molecule. The optimized inhibitor ZHAWOC8697 showed IC_50_ values of 8.6 μM against SENP1 and 2.3 μM against SENP2. With a photo affinity probe the SENP target was validated. This novel SENP inhibitor represents a new valuable tool for the study of SUMOylation processes and the SENP-associated development of small molecule-based treatment options.

## 1. Introduction

SUMOylation is a reversible post-translational modification that targets a variety of proteins by attachment of small ubiquitin-like modifiers (SUMOs) [1,2]. SUMO proteins are similar to ubiquitin and are considered members of the ubiquitin-like protein family. It has been shown that SUMOylation and deSUMOylation has a major influence on cellular processes such as transcription, DNA damage response or cell division [3,4,5,6]. SUMOylation is regulated by a cascade of reactions catalyzed by SUMO-specific activating enzyme E1, conjugation enzyme E2, and ligase enzyme E3, whereas deSUMOylation is regulated by the family of Sentrin-specific proteases (SENPs) [7]. SENPs are cysteine proteases, which are responsible for the activation of proSUMOs to SUMOs and removal of small ubiquitin-like modifiers (SUMOs) from the post-translational modified proteins.

All SUMOs are expressed as proSUMOs. At the C-terminus, during the maturation of proSUMOs to SUMOs, two to eleven amino acids are proteolytically cleaved by SENP endopeptidases. In total, there are six different human SENPs (1, 2, 3, 5, 6, and 7), which have a sequence similarity between 20 and 60%.

SUMOylation is of great importance for the regulated cell cycle and genome stability. If SENP1 expression is incorrectly regulated, serious diseases, such as prostate [8,9], bladder [10], multiple myeloma [11], pancreatic [12] or neuroblastoma cancer [13] can occur. SENP1 is therefore a clinically highly relevant antitumor target and of growing interest for developing novel treatment options for cancer patients [14,15]. Other research showed that SENP1 deSUMOylates and stabilizes hypoxia-inducible factor 1α (HIF-1α) during hypoxia [16].

**Figure 1:**
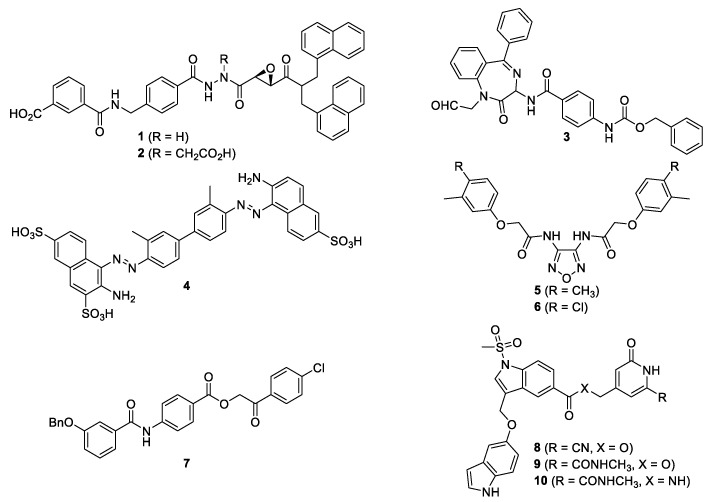
Selection of reported small molecule SENP1 inhibitors (**1**–**10**).

Early research on SENP1 inhibitors focused on peptide-based probes derived from SUMO1 protein, which covalently bind to cysteine in the catalytic site of the protease [17,18]. The first small molecule inhibitors reported in 2011 were also covalent inhibitors targeting the catalytic site resulting in two-digit micromolar inhibitors (**1**–**3**) (Figure 1) [19,20]. Later, non-covalent inhibitors (**4**–**10**) for SENP1 were developed [21,22,23,24,25,26], with the most potent compounds **7** and **9** having IC_50_ values of 1.08 [21] and 0.99 µM [25], respectively (Figure 1). Herein, we report the identification and biological evaluation of novel, non-covalent, small molecule SENP1 inhibitors, resulting, to the best of our knowledge, in one of the most potent non-covalent SENP1 inhibitors currently known.

## 2. Results

### 2.1. Virtual Screening

**Figure 2:**
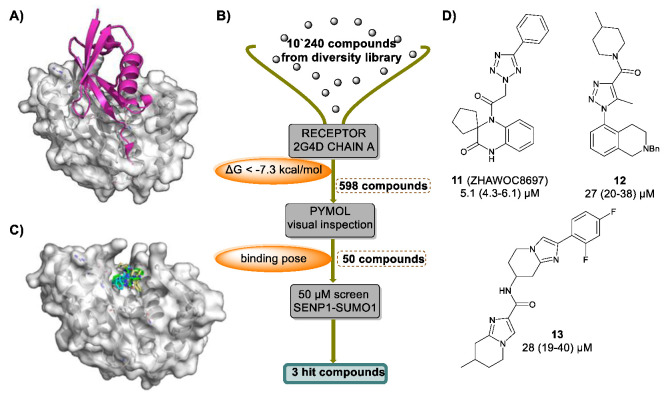
(**A**) X-ray crystal structure of SUMO1 (cartoon: magenta)-SENP1 (PDB code: 2G4D); (**B**) Virtual screening process against the protein-protein interaction between SENP1 and SUMO1; (**D**) The hit structures **11** (ZHAWOC8697), **12** and **13** are shown with the corresponding *in vitro* measured IC_50_ value and the proposed binding mode (**C**).

We envisaged a virtual screening on the interface between SENP1 and SUMO1 (PDB code: 2G4D) proteins to identify new structural elements for the development of novel SENP1 inhibitors (Figure 2A). An in-house diversity library containing 10,240 compounds was screened against the SENP1-SUMO1 interface using AutoDock Vina [27] and resulted in 598 virtual hits with a binding score of <−7.3 kcal/mol (Figure 2B). After visual inspection of the compounds in the hypothesized binding mode to SENP1, 50 promising compounds were shortlisted for screening at 50 µM by a fluorescence-based assay against the catalytic domain of SENP1, using SUMO1-AMC as substrate. At this concentration, five compounds showed a SENP1 activity decrease of >50% (Figure 2C,D). Two of the hit molecules had solubility issues at the tested concentration and were not further investigated. IC_50_ values were determined of the remaining three hit molecules with the most promising compound **11** (ZHAWOC8697) showing an IC_50_ of 5.1 µM (Figure 2D).

**Scheme 1:**
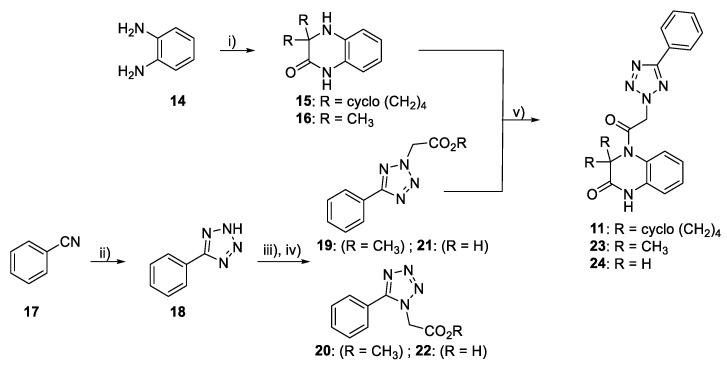
Synthetic scheme for hit compound **11** and the analogues **23** and **24**. Reagents and conditions: (i) cyclopentanone or acetone, **14** (diamine), CHCl_3_, BnEt_3_NCl, CH_2_Cl_2_, 33% NaOH (aq.), 0 °C—rt, overnight, 73% for **15**; 71% for **16**; (ii) NaN_3_, NH_4_Cl, 120 °C, overnight, 87%; (iii) methyl chloroacetate, Et_3_N, MeCN, 70 °C, 2 h, 9:1 mixture of the *2H-* (**19**, 81%) and *1H*- substituted tetrazole (**20**, 9%); (iv) LiOH, THF/H_2_O, rt, 16 h, 100%; (v) 1: **19** (carboxylic acid), oxalyl chloride, DMF (cat.), CH_2_Cl_2_, rt, 15 min, 2: DHQ (**15**, **16** or **25**), Et_3_N, rt, 3 h, 13% (**11**), 7% (**23**), 17% (**24**).

To confirm the activity of the spiro dihydroquinoxalinone (DHQ), compound **11** was resynthesized to validate the activity and the structural identity. Additionally, the importance of the spirocyclic dihydroquinoxalinone was investigated through the synthesis of *gem*-dimethyl and non-substituted dihydroquinoxalinone analogues. The synthesis of the spiro dihydroquinoxalinone (**15**) and *gem*-dimethyl (**16**) building block was performed according to Lai et al. [28] with a Bargellini reaction of 1,2-diaminobenzene (**14**) and cyclopentanone or acetone, chloroform and NaOH to obtain the compounds **15** and **16** in 71% and 73% yield, respectively (Scheme 1).

The synthesis of the phenyl tetrazole building block **21** commenced with a [2 + 3] dipolar cycloaddition of benzonitrile **17** with sodium azide to generate tetrazole **18**. Subsequently the phenyl tetrazole **18** was *N*-alkylated with methyl chloroacetate to the desired 1*H*-(**19**) and 2*H*-regioisomer (**20**), followed by ester hydrolysis to obtain compounds **21** and **22**. The 2*H*-tetrazole **21** was converted to an acid chloride with oxalyl chloride and directly coupled to either the spirocyclic (**15**), *gem*-dimethyl (**16**) or non-substituted (**25**) DHQ derivative to isolate the hit molecule **11** and its close analogues **23** and **24** (Scheme 1).

The resynthesized hit molecule **11** and its derivatives **23** and **24** were tested for the inhibition of the catalytic activity of SENP1 (Table 1). Pleasingly, the inhibition of the resynthesized hit compound **11** was confirmed with an IC_50_ of 8.6 µM. Interestingly, the *gem*-dimethyl (**23**) and non-substituted (**24**) DHQ derivatives showed reduced inhibition by a factor of 1.5 and 10, respectively (Table 1). This raises the question, as to whether this is due to the reduced compound lipophilicity or the steric restriction of the amide bond [29] between the DHQ amine and the bulky carboxylic amide phenyl tetrazole substituent. MD calculations for the rotational barrier of this amide bond were performed by umbrella sampling using Amber 17. The results indicate that with the *gem*-dimethyl and non-substituted dihydroquinoxalinone derivatives two preferred orientations of the amide linker with an angle of 44° and 136° or 22° and 158° are possible, whereas the spirocyclic compound has just one energy minimum at 120° due to steric clash with the bulky spirocyclopentane DHQ scaffold (Figure S1).

### 2.2. Fragments

To investigate this theory, structurally relevant fragments were tested in the SENP1 inhibition assay at concentrations of up to 1 mM. The spirocyclic compound **15** had an IC_50_ of 116 μM, whereas the *gem*-dimethyl DHQ derivative **16** showed only around 50% inhibition at 500 µM (Table 2). The sterically hindered amide bond is not present in those fragments, allowing us to conclude that the different orientations of the amide bond do not result in different ligand binding affinities. 

Various modifications of fragment **15** were synthesized to gain a better understanding of the important features and aiding inhibitor optimization. Compound **26** indicates that the secondary amine is not crucial and can be replaced by an ether resulting in comparable activity (Table 2). Increasing the spirocyclic ring size to increase the lipophilic character was beneficial for the cyclooctane derivative **28** (IC_50_: 51 µM), whereas a slightly reduced activity of the cyclohexyl analogue **27** (IC_50_: 261 µM) was measured. The spiro cyclobutyl compound **29** had a slightly increased potency compared to **15** (Table 2). This is a surprising observation, that the spirocyclic ring size is equally important to the lipophilicity. Incorporation of an oxetane (**30**) or tetrahydropyran (**31**) into the spirocyclic compound resulted in a comparable inhibition profile similar to the aliphatic 4- (**29**) or 6-membered (**27**) derivatives (Table 2). In addition, with the heterospirocyclic compounds, the 6-membered spirocyclic compound **31** is less active compared to the 4-membered analogue **30**. The inverse amide **32** was self-fluorescent at concentrations of up 10 µM and hence no IC_50_ could be determined. Reduction of the amide bond of **15** to the amine **33** resulted in a loss of activity indicating that this hydrogen bond acceptor is crucial (Table 2). Modifications of the aromatic part by introducing a pyridine (**34**) resulted in a complete loss of activity up to 500 µM. Meanwhile, replacing the benzene moiety to a thiophene (**35** and **36**) gave a three-fold affinity increase, which is also a solid improvement in terms of ligand efficiency from 0.37 to 0.44 (Table 2). The attachment of substituents to the aromatic core of the dihydroquinoxalinone, either with an electron-donating methoxy group (**37**, **39**) or an electron-withdrawing group such as methyl ester (**38**, **40**) or nitro (**41**), was well tolerated overall but showed a remarkable dependence on the point of attachment, suggesting defined interactions. Thus, the best affinities in this series were measured with electron-withdrawing groups in the 7- and 8-position (**38**, **41**).

### 2.3. Modifications

**Scheme 2:**
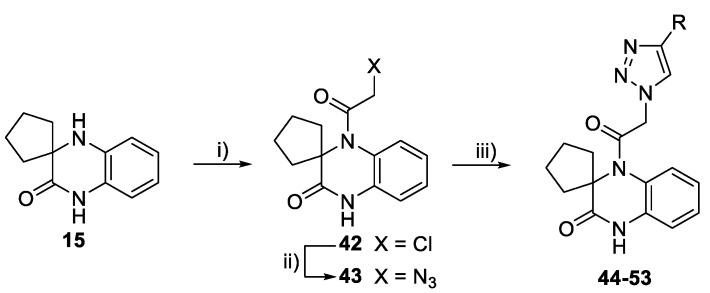
Synthetic scheme of 1,2,3-triazole derivatives (**44**–**53**). Reagents and conditions: (i) 2-chloro acetic chloride, Et_3_N, DMF, 0 °C, 30 min, 98%; (ii) NaN_3_, DMF, 80 °C, 30 min, 78%; (iii) R-alkyne, CuSO_4,_ sodium ascorbate, *t*-BuOH, H_2_O, DMSO, rt, 14–20 h, 15–47%.

Subsequently, the influence of modifications of the phenyl substituent on the heteroaromatic moiety was investigated with respect to the inhibitory effect. For easier synthetic accessibility, the tetrazole was replaced by a 1,2,3 triazole. The synthesis for those derivatives commenced by an *N*-acylation of DHQ fragment **15** with 2-chloroacetic chloride to obtain intermediate **42** (Scheme 2). Substitution of the chlorine with azide by an S_N_2 reaction yielded the azide derivative **43**, which was used to react with different alkyne substrates by copper catalyzed azide-alkyne coupling to obtain the desired triazole derivatives **44–53** in moderate yields. 

In the fluorescence-based assay, the triazole derivative **44** showed a three-fold activity decrease compared to tetrazole **11** with an IC_50_ of 23 µM (Table 3). Despite the reduced activity, the structure activity relationship (SAR) can be well compared to the tetrazole derivatives within this compound series. In general, a flat SAR was observed. Exchanging the phenyl ring (**44**) by a saturated cyclohexyl (**45**) maintained the inhibition profile. A subtle activity decrease was measured with a benzyl (**46**) or 3-benzoic acid (**47**) substituent (Table 3). Having a substituent on the para position of the phenyl ring, such as the electron-deficient methyl ester (**48**) or electron-rich methoxy (**49**), resulted in a slightly increased inhibition of SENP1 with an IC_50_ of 7.5 and 13 µM, respectively (Table 3). Replacement of the phenyl ring with a basic 2-pyridine analogue (**50**) showed only slight inhibition at 200 µM (Table 3). This is not surprising as SENP1 is a lysine rich protein, hence, electronic repulsion of the ligand with the protein may explain this activity decrease. Replacement of the phenyl group with a cyclopropyl (**51**), carboxylic acid (**52**) or ethyl methyl ether (**53**) diminished the SENP1 activity. In addition, other phenyl tetrazole replacements (Supplementary compounds S1–S7) such as phenols, anilines and indolines were tested, however, all of them had drastically reduced inhibition (Table S1). Additionally, the 1*H*-phenyl tetrazole DHQ derivative (**54**) was synthesized by coupling **15** with **22** in an analogous manner to the procedure described in Scheme 1 and tested, which resulted in a five-fold activity reduction compared to the 2*H* derivative (**11**). Compound **55** was synthesized by an amide coupling of thieno [3,4-b]pyrazine (**35**) with the 2*H*-tetrazole (**21**) and is equipotent to the DHQ derivative (**11**). 

### 2.4. Mode of Action and Target Verification

**Figure 3:**
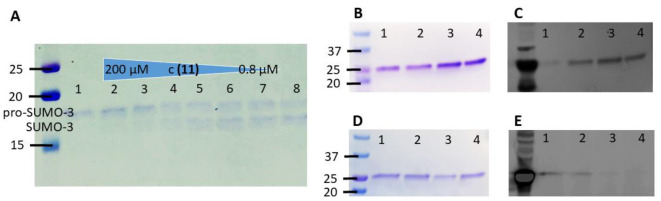
SDS page gels of the maturation of proSUMO3 (**A**) and photo crosslinking experiments with **56** on SENP1 (**B**–**E**). (**A**) Endopeptidase inhibition of **11** against the maturation of proSUMO3 (c = 5 µM) with SENP1 (200 nM) incubated at 37 °C for 1 h. 1: no SENP1 added; 2–7: 3-fold dilution of **11** starting at 200 µM and final concentration of 0.8 µM; 8: no **11** added; (**B**,**C**) crosslinking experiments of **56** (200 µM) with SENP1 (10 µM) followed by CuAAc of the crosslinked probe to azide-fluor 545. 1: no UV irradiation 2–4): UV irradiation for 30 min; (**D**,**E**) crosslinking competition experiment of **56** (200 µM) with SENP1 (10 µM) followed by CuAAc of the crosslinked probe to azide-fluor 545. 1–2: no competitor inhibitor, 3–4: **11** (400 µM) was added. SDS gels were visualized by Coomassie blue stain (**B**,**D**) and fluorescence (Ex. 520 nm; Em. 575 nm) (**C**,**E**).

To confirm **11** is active on the native substrates and not just on the SUMO1-AMC test substrate in the fluorogenic assay, a protocol for gel-based assay for the recombinant human proSUMO substrate was adopted from Mikolajczyk et al. [31]. For proSUMO1, the terminal four amino acids are cleaved by SENP1 in the maturation process of the protein, whereas for proSUMO3 there are 12 amino acids. In the case of proSUMO1, the protein bands on the SDS gel could not be separated. However, with proSUMO3 a good separation of the matured protein with a 15% SDS polyacrylamide gel was achieved. For this reason, the inhibitory endopeptidase activity of **11** on SENP1 was determined with the proSUMO3 substrate. It could be clearly demonstrated that a full inhibition of SENP1 was observed at an inhibitor concentration of 200 µM, whereas at lower concentrations a dose-dependent response was observed (Figure 3A). This confirms that the inhibitor does not only show activity on the SUMO-AMC test substrate, but also on native substrates, which underlines its biological significance and makes it a useful tool for further biological studies.

The photo affinity probe **56** consisting of the spiro DHQ scaffold coupled to a minimalist terminal alkyne-containing diazirine photo crosslinker [32] was synthesized to confirm the inhibitor-protein interaction (Scheme S1). With the fluorescent SENP1-SUMO1-AMC bioassay this compound showed an IC_50_ value of 24 µM (Table 3). In comparison, the *n*-octyl derivative **57**, which does not include the photo labile group, showed similar IC_50_ data, indicating the diazirine group did not interfere in the fluorescent assay. To assess the interaction of SENP1 with the photo affinity probe, **56** was incubated with purified SENP1 and crosslinked via UV irradiation at 350 nM. The protein ligand mixture was coupled by CuAAc with the fluorescent TAMRA-azide dye and separated by SDS page electrophoresis. A fluorescent band was visible indicating the covalent binding of the probe to SENP1, confirming its interaction (Figure 3B,C). A competition experiment with the photo probe **56** and **11** under the same conditions showed that the fluorescently labelled band of SENP1 was significantly decreased, indicating a selective binding to the protein (Figure 3D,E).

**Figure 4:**
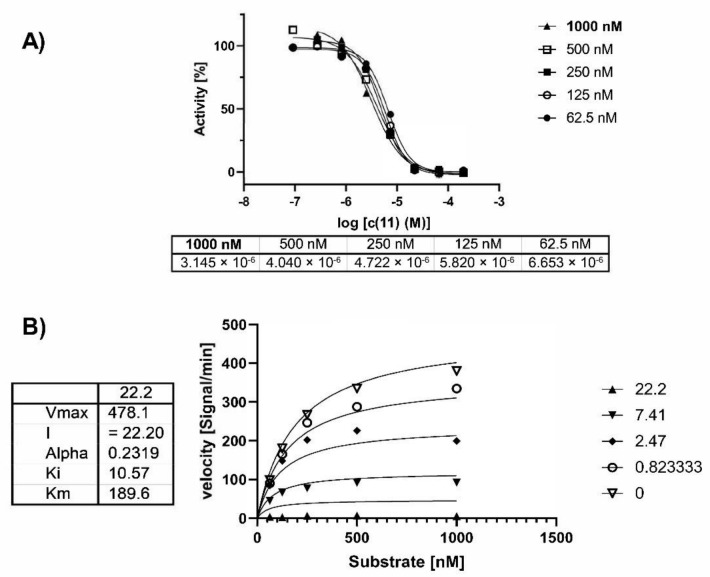
(**A**) Mode of inhibition study: IC_50_ curves for compound **11** (ZHAWOC8697) against SENP1 at different substrate (SUMO1-AMC) concentrations; (**B**) global fit of Michaelis-Menten plots (GraphPad Prism).

A mode of inhibition study with **11** was performed by kinetic measurement with SENP1 and SUMO1-AMC employing five different substrate concentrations (Figure 4). The IC_50_ values decreased slightly by lowering the SUMO1-AMC substrate concentration from 1000 nM to 62.5 nM, indicating a slight binding preference to the SUMO1-SENP1 complex (Figure 4A). On analysis of the mixed inhibition model using GraphPad Prism on the global Michaelis-Menten plot, a K_I_ of 10.6 µM and alpha of 0.23 was observed (Figure 4B). The alpha value indicates that a slight preference for the SUMO1-SENP1 complex is demonstrated. 

### 2.5. Target Selectivity

The selectivity of the most promising compounds **11**, **55** and the most potent published SENP1 inhibitor **7** was evaluated against SUMO2 and SUMO3 as well as SENP2 and the two DUB proteins, UCHL1 and Ataxin 3 (Table 4). The compounds **11** and **55** showed a moderate selectivity for the SUMO1 substrate over the SUMO2 and SUMO3 substrates, whereas **7** resulted in no significant differences. Surprisingly, this compound series had a slightly increased activity on the SENP2 protein. SENP2 has been identified by others to modulate atherosclerosis [33], neurodegenerative diseases [34], fatty acid metabolism [35] and adipogenesis [36]. With this wide range of functions, our discovered inhibitors could also be used to explore further the understudied SENP2 biology.

## 3. Materials and Methods

### 3.1. Computational Studies

#### 3.1.1. Virtual Screening

All docking experiments were performed using AutoDock Vina [27] and PyMol 2.3.5 (The PyMOL Molecular Graphics System, Version 2.0 Schrödinger, LLC) for visualization. SENP1 from co-crystal structure (PDB-code: 2G4D [37]) was used as receptor. The receptor was first prepared by deleting the solvent molecules and SUMO1 protein. In a second step, AutoDockTools package (version 1.5.6) was employed to add the hydrogen atoms and the corresponding charges to the atoms and generate the necessary receptor.pdbqt. The structure preparation of our in-house diversity library was performed with OpenBabel [38] (version 3.1.1) and the python script “prepare_ligand4.py” from the AutodockTools [39,40] package and included 3D structure generation, the addition of hydrogens and partial charges using Gasteiger charges. With AutodockTools, the search space (x = −35.8, y = −25.6, z = 22.4) and grid size (40, 40, 40) were defined and the “exhaustiveness” of the search parameter was set to 9. For the number of binding modes, the default setting of 9 was used. 

#### 3.1.2. Molecular Dynamics Simulations

To validate the conformational preferences of the DHQ tetrazole amide bond, umbrella sampling simulations were carried out for compounds **11**, **23** and **24** calculating the potential of mean force (PMF) as a function of the torsion angles. Prior to umbrella sampling, each small molecule was solvated (TIP3P) and equilibrated at 300 K for 100 ps. The C-N bond was varied from 0 to 180° using umbrella windows spaced 3° apart. Each window was then subjected to a 50 ps incrementally restrained equilibration prior to a 100 ps restrained simulation at constant temperature (25 °C) and pressure (1 atm). The torsions of the central residue were restrained using a harmonic penalty function with a force constant of 200 kcal/mol rad^2^ for each window with 3° intervals about each torsion angle. From each set, the unbiased potential of mean force was reconstructed using the weighted histogram analysis method (WHAM) [41,42].

### 3.2. Chemistry

#### 3.2.1. General Information

All reagents and solvents were purchased from Sigma-Aldrich, Alfa Aesar, Enamine, or Fluorochem and used as received. All NMR spectra were recorded on a Bruker AVANCE III HD 500 One Bay spectrometer with a magnetic field of 11.75 T and a 5 mm SmartProbe BB(F)-H-D. For ^1^H-NMR spectra, a frequency of 500 MHz resulted. Chemical shifts are reported in ppm from tetramethylsilane as internal standard in CDCl_3_ or from [D_6_]DMSO as an internal standard (δ = 2.50). Data are reported as follows: chemical shift, multiplicity (s = singlet, d = doublet, t = triplet, q = quartet, quint = quintet, br = broad, m = multiplet), coupling constants (Hz), integration. For ^13^C-NMR spectra, a frequency of 126 MHz resulted. Chemical shifts are reported in ppm from tetramethylsilane as internal standard in CDCl_3_ or from DMSO-d_6_ (δ = 39.52). Purity was assayed by HPLC (Interchim Strategy C-18 column, 4.6 mm × 250 mm) with a gradient of 5−100% methanol in 0.2% aqueous acetic acid with UV detection at λ = 254 nm. All final compounds were obtained with ≥95% purity. 

#### 3.2.2. General Procedure A for the Bargellini Reaction 

To an ice-cooled suspension of diamine **14** (1 eq.), ketone (2.5 eq.), chloroform (2 eq.) and benzyltriethylammonium chloride (0.05 eq.) in dichloromethane (V_DCM_ = n_Diamin_ 2 mol/L) was added an aq. 33% NaOH solution (5 eq.) over 45 min. The reaction mixture was allowed to warm to ambient temperature and stirred overnight. EtOAc (100 mL) was added to the reddish suspension and the organic layer was washed with brine (2 × 50 mL), dried (Na_2_SO_4_), filtered and concentrated in vacuo. 

#### 3.2.3. General Procedure B for CuAAc

To a solution of azide **43** (50 mg, 0.175 mmol, 1.0 eq) and alkyne (0.175 mmol, 1.0 eq) dissolved in *^t^*BuOH/H_2_O/DMSO (1:1:1; 8 mL) was added a 0.05 M aq. CuSO_4_ solution (700 μL, 0.035 mmol, 0.2 eq) and 0.05 M aq. sodium ascorbate solution (1.75 mL, 0.088 mmol, 0.5 eq). The reaction mixture was stirred at ambient temperature overnight. The suspension was taken up with EtOAc (80 mL), extracted with brine (40 mL), 0.2 M aq. EDTA solution (2 × 40 mL), brine (2 × 40 mL), dried (Na_2_SO_4_), filtered and concentrated in vacuo. 

#### 3.2.4. 3′,4′-Dihydro-1′H-spiro[cyclopentane-1,2′-quinoxalin]-3′-one (**15**)

The reaction with 1,2-diaminobenzene (**14**) (2.16 g, 20.0 mmol, 1.0 eq) and cyclopentanone was performed analogous to the general procedure A. The residue was purified by a silica column eluting with a gradient of 0 to 50% EtOAc in cyclohexane to afford a brownish solid of **15** (2.96 g, 14.6 mmol, 73%). Mp 174 °C [Lit [28]: 176–179 °C]; ^1^H-NMR (500 MHz, (CD_3_)_2_SO): *δ* 10.51 (s, 1H), 6.78–6.68 (m, 3H), 6.62–6.57 (m, 1H), 6.03 (s, 1H), 2.03–1.95 (m, 2H), 1.78–1.71 (m, 2H), 1.65–1.51 (m, 4H). ^13^C-NMR (126 MHz, (CD_3_)_2_SO): *δ* 171.0, 134.7, 127.1, 122.9, 118.3, 114.8, 114.3, 65.2, 36.9, 24.8. LRMS (ESI) *m*/*z* [M+H]^+^ 203; RP-HPLC: RT 11.11 min; purity: 94.1%.

#### 3.2.5. 3,3-Dimethyl-1,2,3,4-tetrahydroquinoxalin-2-one (**16**)

The reaction with 1,2-diaminobenzene (**14**) (2.16 g, 20.0 mmol) and acetone was performed analogous to the general procedure A. The residue was purified by a silica column eluting with a gradient of 10 to 70% EtOAc in cyclohexane to afford a yellowish solid of **16** (2.51 g, 14.3 mmol, 71%). Mp 178 °C [Lit [43]: 178 °C]; ^1^H-NMR (500 MHz, (CD_3_)_2_SO): *δ* 10.13 (s, 1H), 6.76 (ddd, *J =* 7.6, 7.6, 1.2 Hz, 1H), 6.73 (dd, *J =* 7.6, 1.2 Hz, 1H), 6.67 (dd, *J =* 7.6, 1.2, 1H), 5.94 (s, 1H), 1.21 (s, 6H). ^13^C-NMR (126 MHz, (CD_3_)_2_SO): *δ* 170.7, 134.3, 126.6, 123.1, 118.2, 114.8, 114.1, 54.6, 25.3. LRMS (ESI) *m*/*z* [M+H]^+^ 177; RP-HPLC: RT 9.97 min; purity: 98.2%.

#### 3.2.6. 5-Phenyl-2*H*-1,2,3,4-tetrazole (**18**)

A suspension of NaN_3_ (3.00 g, 46.2 mmol, 2.3 eq), NH_4_Cl (2.50 g, 46.7 mmol, 2.3 eq) and benzonitrile (**17**) (2.1 mL, 20.4 mmol, 1 eq) in DMF (40 mL) was stirred at 120 °C overnight. The white suspension was cooled with an ice bath and water (20 mL) was added. The resulting colorless solution was acidified with 1 M HCl (35 mL) leading to precipitation of a white solid. The phenyltetrazole was filtered off and dried in a vacuum-drying cabinet (40 °C, overnight) to afford a white solid of **18** (2.65 g, 18.1 mmol, 91%). ^1^H-NMR (500 MHz, (CD_3_)_2_SO): *δ* 8.08–8.03 (m, 2H), 7.64–7.57 (m, 3H). ^13^C-NMR (126 MHz, (CD_3_)_2_SO): *δ* 155.9 (HMBC), 131.2, 129.4, 127.0, 124.1. LRMS (ESI) *m*/*z* [M+H]^+^ 147.

#### 3.2.7. Methyl 2-(5-phenyl-2*H*-1,2,3,4-tetrazol-2-yl)acetate (**19**) and methyl 2-(5-phenyl-1*H*-1,2,3,4-tetrazol-2-yl)acetate (**20**)

A solution of **18** (2.31 g, 15.8 mmol, 1 eq) and Et_3_N (8.8 mL, 63.5 mmol, 4.0 eq) in MeCN (100 mL) was added to a refluxing solution of methyl chloroacetate (2.8 mL, 31.9 mmol, 2.0) and stirred at reflux for 2 h. The reaction was left to cool to ambient temperature and was then concentrated in vacuo. The residue was separated by a silica column eluting with a gradient of 0 to 20% TBME in toluene to afford **19** as a colorless solid (2.66 g, 12.2 mmol, 77%) and **20** as a yellowish oil (0.44 g, 2.02 mmol, 13%). **19**: ^1^H-NMR (500 MHz, CDCl_3_): *δ* 8.19–8.14 (m, 2H), 7.53–7.45 (m, 3H), 5.47 (s, 2H), 3.83 (s, 3H). ^13^C-NMR (126 MHz, CDCl_3_): *δ* 165.8, 165.7, 130.7, 129.1, 127.2, 127.1, 53.4, 53.4. LRMS (ESI) *m*/*z* [M+H]^+^ 219. **20**: ^1^H-NMR (500 MHz, CDCl_3_): *δ* 7.66–7.62 (m, 2H), 7.62–7.57 (m, 1H), 7.57–7.53 (m, 2H), 5.20 (s, 2H), 3.80 (s, 3H). ^13^C-NMR (126 MHz, CDCl_3_): *δ* 166.1, 155.4, 131.7, 129.6, 128.8, 123.5, 53.5, 48.7. LRMS (ESI) *m*/*z* [M+H]^+^ 219.

#### 3.2.8. 2-(5-Phenyl-2H-1,2,3,4-tetrazol-2-yl)acetic acid (**21**)

LiOH × H_2_O (1.04 g, 24.7 mmol, 2 eq) was added to a solution of **19** (2.66 g, 12.2 mmol, 1 eq) in THF (30 mL) and water (20 mL). The reaction was stirred at ambient temperature overnight. The reaction mixture was acidified with 32% HCl to pH = 1 and brine (100 mL) was added and the resulting mixture was extracted with EtOAc (3 × 100 mL). The combined org. layers were dried (Na_2_SO_4_), filtered and concentrated in vacuo to obtain a white solid of **21** (2.45 g, 12.0 mmol, 98%) which was used without further purification. Mp 179-184 °C decomp. [Lit [44]: 186–187 °C]; ^1^H-NMR (500 MHz, (CD_3_)_2_SO): *δ* 13.76 (br s, 1H), 8.10–8.06 (m, 2H), 7.61–7.53 (m, 3H), 5.76 (s, 2H). ^13^C-NMR (126 MHz, (CD_3_)_2_SO): *δ* 167.5, 164.2, 130.7, 129.3, 126.7, 126.4 53.6. LRMS (ESI) *m*/*z* [M+H]^+^ 205.

#### 3.2.9. 2-(5-Phenyl-1*H*-1,2,3,4-tetrazol-1-yl)acetic acid (**22**)

LiOH × H_2_O (0.17 g, 4.05 mmol, 2 eq) was added to a solution of **20** (0.44 g, 2.02 mmol, 1 eq) in THF (4.9 mL) and water (3.2 mL). The reaction was stirred at ambient temperature overnight. The reaction mixture was acidified with 32% aq. HCl to pH = 1 and brine (20 mL) were added and the resulting mixture was extracted with EtOAc (3 × 20 mL). The combined org. layers were dried (Na_2_SO_4_), filtered and concentrated in vacuo to obtain a yellowish solid of **22** (0.37 g, 1.81 mmol, 90%) which was used without further purification. Mp 171–178 °C decomp. [Lit [45]: 180–182 °C]; ^1^H-NMR (500 MHz, (CD_3_)_2_SO): *δ* 13.75 (br s, 1H), 7.78–7.74 (m, 2H), 7.67–7.59 (m, 3H), 5.51 (s, 2H). ^13^C-NMR (126 MHz, (CD_3_)_2_SO): *δ* 167.7, 154.6, 131.4, 129.3, 128.4, 123.5, 49.1. LRMS (ESI) *m*/*z* [M+H]^+^ 205.

#### 3.2.10. 1′-[2-(5-Phenyl-2*H*-1,2,3,4-tetrazol-2-yl)acetyl]-3′,4′-dihydro-1′*H*-spiro[cyclopentane-1,2′-quinoxalin]-3′-one (**11**)

To a suspension of 2-(5-phenyl 2*H*-1,2,3,4 tetrazol-yl acetic acid) (**21**) (46.0 mg, 0.225 mmol, 1.0 eq) in dichloromethane (3 mL) was added oxalyl chloride (28.6 mg, 0.225 mmol, 1.1 eq) followed by a drop of DMF and stirred for 15 min at ambient temperature. To this solution, **15** (50 mg, 0.205 mmol, 1.0 eq) and Et_3_N (47 μL, 0.308 mmol, 1.5 eq) were added and stirred for 3 h. This solution was diluted with dichloromethane (20 mL), washed with brine (3 × 20 mL), dried (Na_2_SO_4_), filtered and concentrated in vacuo. The residue was purified by a SiO_2_ column eluting with a gradient of 10 to 60% EtOAc in cyclohexane to afford an off-white solid, which still contains some carboxylic acid. A second chromatographic purification on SiO_2_ eluting with a gradient of 10 to 50% EtOAc in cyclohexane resulted in a colorless solid of **11** (9.3 mg, 0.027 mmol, 13%). Mp 237 °C decomp.; ^1^H-NMR (500 MHz, (CD_3_)_2_CO): *δ* 9.60 (s, 1H), 8.12–8.07 (m, 2H), 7.86–7.83 (m, 1H), 7.56–7.50 (m, 3H), 7.43 (ddd, *J* = 7.8, 7.8, 1.3, 1H), 7.27–7.22 (m, 2H), 5.67 (br s, 2H), 2.25–1.89 (br m, 4H), 1.82–1.60 (br m, 4H). ^13^C-NMR (126 MHz, (CD_3_)_2_SO): *δ* 171.7, 166.7, 164.7, 134.9, 130.3, 129.0, 129.7, 128.0, 127.6, 126.5, 126.3, 122.8 116.2, 70.2, 55.4, 34.5, 23.5. LRMS (ESI) *m*/*z* [M+H]^+^ 389. RP-HPLC: RT 12.78 min; purity: 99.7%.

#### 3.2.11. 3,3-Dimethyl-4-[2-(5-phenyl-2*H*-1,2,3,4-tetrazol-2-yl)acetyl]-1,2,3,4-tetrahydroquinoxalin-2-one (**23**)

The 2-(5-Phenyl 2*H*-1,2,3,4-tetrazol-yl acetic acid) (**21**) (128 mg, 0.625 mmol, 1.1 eq) was suspended in dichloromethane (3 mL). Oxalyl chloride (52.5 µL, 78.8 mg, 0.625 mmol, 1.1 eq) and a drop of DMF were added and stirred for 15 min. Then, **6** (100 mg, 0.568 mmol, 1.0 eq) and Et_3_N (158 µL, 115 mg, 1.141 mmol, 2.0 eq) dissolved in dichloromethane (3 mL) were added over 5 min and stirred at ambient temperature and monitored by UPLC-MS. After 30 min, the reaction was completed. The reaction mixture was diluted with EtOAc (30 mL) and extracted with sat. NaHCO_3_ (2 × 30 mL) and brine (2 × 30 mL), dried (Na_2_SO_4_), filtered and concentrated in vacuo. The residue was purified by a SiO_2_ column eluting with a gradient of 10 to 80% EtOAc in cyclohexane to afford an off-white solid, which still had some impurities. A second purification on a reversed phase C_18_ column eluting with a gradient of 10 to 100% MeCN in H_2_O containing 0.1% AcOH to afford a colorless solid of **23** (14.8 mg, 0.041 mmol, 7%). Mp 252 °C decomp.; ^1^H-NMR (500 MHz, (CD_3_)_2_SO): *δ* 10.76 (s, 1H), 8.07–8.01 (m, 2H), 7.66–7.62 (m, 1H), 7.61–7.53 (m, 3H), 7.28 (ddd, *J* = 7.7, 7.7, 1.0 Hz, 1H), 7.13 (ddd, *J* = 7.7, 7.7, 1.0 Hz, 1H), 7.03 (dd, *J* = 7.7, 1.0 Hz, 1H), 5.80 (s br, 2H), 1.49 (s, 6H). ^13^C-NMR (126 MHz, (CD_3_)_2_SO): *δ* 171.5, 166.5, 164.5, 132.0, 131.1, 129.8, 127.9, 127.2, 126.8, 125.4, 125.2, 123.4, 116.2, 61.8, 56.7, 23.5 ppm. LRMS (ESI) *m*/*z* [M+H]^+^ 363. RP-HPLC: rt 12.39 min; purity: 99.4%.

#### 3.2.12. 4-[2-(5-Phenyl-2*H*-1,2,3,4-tetrazol-2-yl)acetyl]-1,2,3,4-tetrahydroquinoxalin-2-one (**24**)

2-(5-Phenyl 2*H*-1,2,3,4 tetrazol-yl acetic acid) (**21**) (60.3 mg, 0.295 mmol, 1.1 eq) was suspended in dichloromethane (3 mL). Oxalyl chloride (31.8 μL, 47.7 mg, 0.378 mmol, 1.4 eq) and a drop of DMF were added and stirred for 15 min at ambient temperature. The 1,2,3,4-tetrahydroquinoxalin-2-one (40 mg, 0.270 mmol, 1.0 eq) and Et_3_N (75 μL, 0.541 mmol, 2.0 eq) were added and stirred at ambient temperature and monitored by UPLC-MS. After 30 min, the reaction was completed. The reaction mixture was diluted with EtOAc (30 mL) and extracted with sat. NaHCO_3_ (2 × 30 mL), brine (2 × 30 mL), dried (Na_2_SO_4_), filtered and concentrated in vacuo. The residue was purified by a SiO_2_ column chromatography eluting with a gradient of 30 to 100% EtOAc in cyclohexane to afford an off-white solid, which still had some impurities. A second purification on the reversed phase C_18_ column eluting with a gradient of 10 to 100% MeCN in H_2_O containing 0.1% AcOH afforded a white solid of **24** (15.6 mg, 0.047 mmol, 17%). Mp 231 °C decomp.; ^1^H-NMR (500 MHz; 375K; (CD_3_)_2_SO): *δ* 10.61 (s, 1H), 8.08–8.04 (m, 2H), 7.78–7.73 (m, 1H), 7.61–7.52 (m, 3H), 7.29–7.22 (m, 1H), 7.12–7.05 (m, 2H), 6.06 (s, 2H), 4.44 (dd, *J =* 0.9 Hz, 2H). ^13^C-NMR (126 MHz, (CD_3_)_2_SO): *δ* 167.1 (HMBC), 164.7, 164.6, 132.6 (HMBC), 131.1, 129.8, 127.8 (HMBC), 127.2, 126.8, 125.6, 124.2, 122.9 (HSQC), 117.0 (HSQC), 54.7 (HSQC), 47.3 (HSQC). LRMS (ESI) *m*/*z* [M+H]^+^ 335. RP-HPLC: RT 11.46 min; purity: 99.4%.

#### 3.2.13. 3,4-Dihydrospiro [1,4-benzoxazine-2,1′-cyclopentan]-3-one (**26**)

To an ice-cooled solution of methyl 1-hydroxycyclopentan-1-carboxylate (0.55 g, 3.62 mmol, 1 eq) in dry THF (7.5 mL) was added sodium hydride (60% suspension in paraffin oil, 0.185 g, 4.35 mmol, 1.2 eq) in two portions (caution: vigorous gas production). Then, 10 min after the completed addition, 2-fluoronitrobenzene (0.46 mL, 4.39 mmol, 1.2 eq) was added and stirred at 0 °C for another 20 min. The reaction mixture was stirred at ambient temperature overnight. The reaction mixture was quenched with sat. aq. NH_4_Cl solution (25 mL), diluted with water (5 mL) and the organic components of the aq. layer were extracted with EtOAc (3 × 30 mL). The combined organic layers were dried (MgSO_4_), filtered and concentrated in vacuo. The residue was purified by silica column chromatography eluting with a gradient of 0 to 20% EtOAc in cyclohexane to afford the impure ether intermediate, which was directly used in the next step. Zn powder (2.09 g, 32.0 mmol, 8.8 eq) and NH_4_Cl (1.71 g, 32.0 mmol, 8.8 eq) were carefully added to a stirred solution of the ether intermediate in DMF (3 mL). The reaction was stirred for 3 h at ambient temperature. The reaction mixture was filtered and rinsed with EtOAc (100 mL). The filtrate was washed with brine (3 × 30 mL), 0.5 M aq. LiCl solution (20 mL) and brine (20 mL), dried (Na_2_SO_4_), filtered and concentrated in vacuo. The crude was purified by a silica column eluting with a gradient of 0 to 20% EtOAc in cyclohexane to afford an off-white solid **26** (0.13 g, 0.64 mmol, 18% over 2 steps). Mp 157–162 °C; ^1^H-NMR (500 MHz, CDCl_3_): *δ* 7.83 (s, 1H), 6.99–6.92 (m, 3H), 6.79–6.73 (m, 1H), 2.23–2.13 (m, 2H), 2.05–1.97 (m, 2H), 1.95–1.75 (m, 4H). ^13^C-NMR (126 MHz, CDCl_3_): *δ* 170.1, 143.0, 127.3, 124.0, 122.5, 117.9, 115.2, 88.6, 35.7, 24.7. LRMS (ESI) *m*/*z* [M+H]^+^ 204. RP-HPLC: RT 12.99 min; purity: 99.7%.

#### 3.2.14. 3′,4′-Dihydro-1′*H*-spiro[cyclohexane-1,2′-quinoxalin]-3′-one (**27**)

The reaction with 1,2-diaminobenzene (**14**) (1.08 g, 10.0 mmol) and cyclohexanone was performed analogous to the general procedure A. The residue was purified by a silica column eluting with a gradient of 0 to 30% EtOAc in cyclohexane to afford a greenish solid of **27** (0.89 g, 4.12 mmol, 41%). Mp 230–234 °C decomp. [Lit [8]: 239–240 °C]; ^1^H-NMR (500 MHz, (CD_3_)_2_SO): *δ* 10.08 (br s, 1H), 6.89 (dd, *J* = 7.8, 1.1 Hz, 1H), 6.75 (td, *J* = 7.6, 1.5 Hz, 1H), 6.70 (dd, *J* = 7.8, 1.4 Hz, 1H), 6.59 (ddd, *J* = 7.6, 7.6, 1.3 Hz, 1H), 5.92 (br s, 1H), 1.71–1.42 (m, 9H), 1.29–1.16 (m, 1H). ^13^C-NMR (126 MHz, (CD_3_)_2_SO): *δ* 170.1, 133.3, 126.2, 122.5, 117.8, 114.2, 55.7, 31.4, 25.02, 19.9. LRMS (ESI) *m*/*z* [M+H]^+^ 217. RP-HPLC: RT 11.97 min; purity: >99.9%.

#### 3.2.15. 3′,4′-Dihydro-1′*H*-spiro[cyclooctane-1,2′-quinoxalin]-3′-one (**28**)

The reaction with 1,2-diaminobenzene (**14**) (1.08 g, 10.0 mmol) and cyclooctanone was performed analogous to the general procedure A. The residue was purified by a silica column eluting with a gradient of 0 to 40% EtOAc in cyclohexane to afford a reddish/brown solid of **28** (0.07 g, 0.29 mmol, 3%). ^1^H-NMR (500 MHz, CDCl_3_): *δ* 7.90 (br s, 1H), 6.88 (td, *J* = 7.6, 1.1 Hz, 1H), 6.75 (td, *J* = 7.6, 1.2 Hz, 2H), 6.70–6.65 (m, 2H), 3.93 (br s, 1H), 2.28–2.17 (m, 2H), 1.71–1.52 (m, 12H). ^13^C-NMR (126 MHz, CDCl_3_): *δ* 171.3, 132.8, 125.8, 123.8, 119.4, 114.7, 114.5, 60.1, 31.7, 28.4, 25.1, 22.2. LRMS (ESI) *m*/*z* [M+H]^+^ 245. RP-HPLC: RT 11.74 min; purity: 96.1%.

#### 3.2.16. 3′,4’-Dihydro-1′*H*-spiro[cyclobutane-1,2′-quinoxalin]-3′-one (**29**)

The reaction with 1,2-diaminobenzene (**14**) (0.546 g, 5.05 mmol) and cyclobutanone was performed analogous to the general procedure A. The residue was purified by a silica column eluting with a gradient of 0 to 20% EtOAc in cyclohexane followed by a RP column eluting with 10 to 100% B in A (A: 95/5 water/MeCN + 0.2% AcOH, B: 5/95 water/MeCN + 0.2% AcOH) to afford a yellowish solid of **29** (0.07 g, 0.29 mmol, 3%). Mp 189–194 °C decomp.; ^1^H-NMR (500 MHz, CD_3_OD): *δ* 6.82 (ddd, *J* = 7.8, 7.3, 1.5 Hz, 1H), 6.76–6.72 (m, 2H), 6.68 (ddd, *J* = 7.7, 7.3, 1.3 Hz, 1H), 2.62–2.54 (m, 2H), 2.14–2.06 (m, 2H), 1.98–1.81 (m, 2H). ^13^C-NMR (126 MHz, CD_3_OD): *δ* 172.6, 134.7, 127.4, 124.5, 120.1, 115.9, 115.4, 60.0, 34.7, 13.3. LRMS (ESI) *m*/*z* [M+H]^+^ 245. RP-HPLC: RT 10.34 min; purity: 98.1%.

#### 3.2.17. 3′,4′-Dihydro-1′*H*-spiro[oxetane-3,2′-quinoxalin]-3′-one (**30**)

The reaction with 1,2-diaminobenzene (**14**) (1.08 g, 10.0 mmol) and cyclobutanone was performed analogous to the general procedure A. The residue was purified over a silica gel column eluting with a gradient of 0 to 70% EtOAc in cyclohexane. A total of 100 mg of the still impure product was separated by a semi-prep RP chromatography (C_18_ column) eluting with a gradient of 14 to 95% MeCN in water, which afforded a red solid of **30** (0.017 g, 0.089 mmol, 1%). Mp 165–170 °C decomp.; ^1^H-NMR (500 MHz, (CD_3_)_2_SO): *δ* 10.46 (br s, 1H), 7.18 (br s, 1H), 6.81–6.71 (m, 3H), 6.62 (dd, *J* = 7.0, 7.0 Hz, 1H), 4.87 (d, *J* = 6.0 Hz, 2H), 4.47 (d, *J* = 6.0 Hz, 1H). ^13^C-NMR (126 MHz, (CD_3_)_2_SO): *δ* 166.0, 132.3, 125.3, 122.9, 118.3, 114.7, 113.6, 81.0, 58.6. LRMS (ESI) *m*/*z* [M+H]^+^ 191. RP-HPLC: RT 9.04 min; purity: 95.2%.

#### 3.2.18. 3′,4′-Dihydro-1′*H*-spiro[oxane-4,2′-quinoxalin]-3′-one (**31**)

The reaction with 1,2-diaminobenzene (**14**) (1.08 g, 10.0 mmol) and tetrahydro-4*H*-pyran-4-one was performed analogous to the general procedure A. The residue was purified over a silica gel column eluting with a gradient of 0 to 40% EtOAc in cyclohexane. The impurity could be separated by RP chromatography (C_18_ column) eluting with a gradient of 14 to 50% MeCN in water with 0.2% AcOH, which afforded **31** as a brownish-yellow solid (0.185 g, 0.85 mmol, 8%). Mp 211–226 °C decomp.; ^1^H-NMR (500 MHz, CDCl_3_): δ 8.06 (br s, 1H), 6.92 (ddd, *J* = 7.7, 7.7, 1.2 Hz, 1H), 6.81 (ddd, *J* = 7.7, 7.7, 1.2 Hz, 1H), 6.77 (dd, *J* = 7.7, 1.2 Hz, 1H), 6.73 (dd, *J* = 7.7, 1.2 Hz, 1H), 4.06 (br s, 1H), 4.00–3.88 (m, 2H), 3.82–3.67 (m, 2H), 2.28–2.15 (m, 2H), 1.65–1.55 (m, 2H). ^13^C–NMR (126 MHz, CDCl_3_): δ 169.6, 132.0, 125.7, 124.0, 120.2, 115.2, 115.1, 63.6, 54.5, 33.0. LRMS (ESI) *m*/*z* [M+H]+ 219. RP-HPLC: RT 9.69 min; purity: 99.1%.

#### 3.2.19. 3′,4′-Dihydro-1′*H*-spiro[cyclopentane-1,2′-quinazolin]-4′-one (**32**)

To a solution of 2-aminobenzamid (0.255 g, 1.87 mmol, 1 eq) and cyclopentanone (0.17 mL, 1.92 mmol, 1 eq) in EtOH (5.5 mL) was added NH_4_Cl (8.5 mg, 0.159 mmol, 0.08 eq). The reaction was stirred for 24 h at ambient temperature. Water (5 mL) was added and the precipitate was filtered off. The dry crystals were recrystallized in acetone (2 mL) to afford the clean product of **32** (0.15 g, 0.742 mmol, 40%). Mp 233–238 °C decomp. [Lit. 254–256 °C [46]]; ^1^H-NMR (500 MHz, (CD_3_)_2_SO): *δ* 8.07 (br s, 1H), 7.57 (d, *J* = 7.7, 1H), 7.24–7.18 (m, 1H), 6.73 (br s, 1H), 6.69 (d, *J* = 7.8 Hz, 1H), 6.65–6.60 (m, 1H), 1.85–1.73 (m, 4H), 1.71–1.62 (m, 4H). ^13^C-NMR (126 MHz, (CD_3_)_2_SO): *δ* 163.4, 147.5, 133.0, 127.2, 116.5, 114.6, 114.3, 77.1, 39.3, 22.0. LRMS (ESI) *m*/*z* [M+H]^+^ 203. RP-HPLC: RT 10.64 min; purity: > 99.9%.

#### 3.2.20. 3′,4′-Dihydro-1′*H*-spiro[cyclopentane-1,2′-quinoxaline] (**33**)

To an ice cooled solution of **15** (0.20 g, 0.494 mmol, 1.0 eq.) in THF (1 mL) a flame-dried flask was added a solution of LiAlH_4_ (2 M in THF, 0.99 mL, 0.494 mmol, 1 eq.) dropwise. The reaction was heated at reflux overnight. The mixture was cooled with an ice bath, quenched with EtOAc (4 mL) and water (4 mL), filtered over celite and rinsed with EtOAc (3 × 30 mL) and water (3 × 10 mL). The pH of the aqueous phase was set to alkaline with NaOH (2 M, ca. 5 mL), the phase was separated, and the aq. phase was extracted with EtOAc (3 × 50 mL). The combined org. layers were dried (Na_2_SO_4_), filtered and evaporated in vacuo to give a dark oil. The residue was purified by a silica column eluting with a gradient of 0 to 25% EtOAc in cyclohexane to afford a reddish-brownish solid of **33** (0.13 g, 0.690 mmol, 70%). Mp 80–82 °C; ^1^H-NMR (500 MHz, CDCl_3_): *δ* 6.62–6.56 (m, 2H), 6.55–6.51 (m, 1H), 6.50–6.46 (m, 1H), 3.68 (br s, 2H), 3.12 (s, 2H), 1.83–1.64 (m, 6H), 1.62-1.54 (m, 2H). ^13^C-NMR (126 MHz, CDCl_3_): *δ* 133.3, 133.1, 118.8, 118.7, 115.1, 114.5, 59.6, 50.7, 38.3, 24.3. LRMS (ESI) *m*/*z* [M+H]^+^ 189. RP-HPLC: RT 7.11 min; purity: 99.0%. 

#### 3.2.21. 3′,4′-Dihydro-1′*H*-spiro[cyclopentane-1,2′-pyrido [2,3-b]pyrazin]-3′-one (**34**)

The reaction with 6-bromopyridine-2,3-diamine (0.74 g, 3.74 mmol) and cyclopentanone was performed analogous to the general procedure A. The residue was purified by a silica column eluting with a gradient of 0 to 30% EtOAc in cyclohexane to afford a yellowish solid (0.33 g, 1.17 mmol, 31%). Mp 220–226 °C decomp.; ^1^H-NMR (500 MHz, (CD_3_)_2_SO): δ 10.91 (br s, 1H), 6.99–6.94 (m, 2H), 6.52 (br s, 1H), 2.05–1.97 (m, 2H), 1.79–1.70 (m, 2H), 1.66–1.52 (m, 4H). ^13^C-NMR (126 MHz, (CD_3_)_2_SO): δ 170.8, 141.0, 129.3, 125.0, 122.5, 121.4, 64.8, 36.9, 24.4. LRMS (ESI) *m*/*z* [M+H]^+^ 282. RP-HPLC: rt 11.73 min; purity: 96.2%. A suspension of Pd/C (30 mg, 0.014 mmol, 0.06 eq) and (68 mg, 0.23 mmol, 1 eq) in MeOH (3 mL) was degassed with Argon and then H_2_ and stirred for 5 h. The reaction was filtered over celite and the filter cake was rinsed with EtOAc (3 × 10 mL). The solvent was evaporated in vacuo and the crude product was purified by silica column eluting with 0 to 50% EtOAc in cyclohexane to afford an off-white solid 34 (6 mg, 0.030 mmol, 13%). Mp 226–233 decomp.; ^1^H-NMR (500 MHz, CDCl_3_): δ 9.80 (br s, 1H), 7.85 (dd, *J* = 5.0, 1.4 Hz, 1H), 6.92 (dd, *J* = 7.7, 1.4 Hz, 1H), 6.83 (dd, *J* = 7.7, 5.0 Hz, 1H), 3.87 (br s, 1H), 2.37–2.26 (m, 2H), 1.88–1.62 (m, 6H) ^13^C-NMR (126 MHz, CDCl_3_): δ 171.4, 140.9, 138.2, 128.9, 120.5, 119.1, 66.4, 37.9, 24.8. LRMS (ESI) *m*/*z* [M+H]^+^ 204. RP-HPLC: RT 10.09 min; purity: 98.0%. 

#### 3.2.22. 3′,4′-Dihydro-1′*H*-spiro[cyclopentane-1,2′-thieno [3,4-b]pyrazin]-3′-one (**35**) 

The reaction with thiophene-3,4-diamine (0.25 g, 2.19 mmol) and cyclopentanone was performed analogous to the general procedure A. The residue was purified by a silica column eluting with a gradient of 0 to 50% EtOAc in cyclohexane to afford a yellowish solid **35** (0.23 g, 1.10 mmol, 50%). Mp 126–130 °C decomp.; ^1^H-NMR (500 MHz, CDCl_3_): *δ* 8.84 (br s, 1H), 6.38 (d, *J* = 3.1 Hz, 1H), 6.09 (d, *J* = 3.1 Hz, 1H), 3.91 (br s, 1H), 2.26–2.19 (m, 2H), 1.81–1.68 (m, 6H). ^13^C–NMR (126 MHz, CDCl_3_): *δ* 171.7, 134.5, 129.2, 102.0, 99.0, 66.8, 37.5, 24.7. LRMS (ESI) *m*/*z* [M+H]^+^ 209, RP-HPLC: RT 10.89 min; purity: 99.1%.

#### 3.2.23. 3′,4′-Dihydro-1′*H*-spiro[cyclohexane-1,2′-thieno [3,4-b]pyrazin]-3′-one (**36**)

The reaction with thiophene-3,4-diamine (0.25 g, 2.19 mmol) and cyclohexanone was performed analogous to the general procedure A. The residue was purified by a silica column eluting with a gradient of 0 to 30% EtOAc in cyclohexane to afford a yellowish solid **36** (0.055 g, 0.25 mmol, 11%). Mp 170–174 °C decomp.; ^1^H-NMR (500 MHz, CDCl_3_): *δ* 8.28 (br s, 1H), 6.36 (d, *J* = 3.1 Hz, 1H), 6.12 (d, *J* = 3.1 Hz, 1H), 4.27 (br s, 1H), 1.90–1.84 (m, 2H), 1.72–1.67 (m, 5H), 1.46–1.30 (m, 3H). ^13^C–NMR (126 MHz, CDCl_3_): *δ* 171.4, 133.7, 128.5, 101.8, 98.6, 57.8, 32.1, 25.1, 20.8. LRMS (ESI) *m*/*z* [M+H]^+^ 209. RP-HPLC: RT 11.52 min; purity: 95.3%.

#### 3.2.24. 5′-Methoxy-3′,4′-dihydro-1′*H*-spiro[cyclopentane-1,2′-quinoxalin]-3′-one (**37**)

A suspension of 3-fluoro-2-nitroanisol (182 mg, 1.06 mmol, 1.0 eq), methyl 1-aminocyclopentane-1-carboxylate (191 mg, 1.06 mmol, 1.00 eq) and potassium carbonate (305 mg, 2.55 mmol, 2.40 eq) in DMF (1.8 mL) was heated to 80 °C for 11 d. The dark brown reaction mixture was diluted with EtOAc (50 mL), water (5 mL) and brine (30 mL). The organic phase was washed with brine (25 mL), 0.5 M LiCl solution (20 mL) and brine (15 mL), dried (Na_2_SO_4_), filtered and concentrated in vacuo. The residue was purified by silica column (40 g) chromatography (0 to 15% EtOAc in cyclohexane). The impure product fractions were pooled, evaporated and purified again over RP column (C_18_, 250/21 mm HPLC) eluting with 10 to 100% B in A (A: 95/5 water/MeCN, B: 5/95 water MeCN) to afford a yellow solid (27 mg). This yellow solid (27 mg, 0.917 mmol, 1 eq), NH_4_Cl (0.123 g, 2.30 mmol, 25 eq) and Zn (0.155 g, 2.37 mmol, 26 eq) in DMF (1 mL) was stirred overnight. The reaction mixture was diluted with EtOAc (50 mL), filtered and the filter cake was rinsed with EtOAc (2 × 10 mL). The organic layer was washed with water (2 × 20 mL), 0.5 M LiCl solution (10 mL) and brine (10 mL), dried (Na_2_SO_4_), filtered and evaporated in vacuo. The residue was purified by a RP column (C_18_, 250/21 mm HPLC) eluting with a gradient of 10 to 100% B in A (A: 95/5 H_2_O/MeCN, B: 5/95 H_2_O/MeCN) to afford an ochre solid of **37** (16 mg, 0.0689 mmol, 7% over 2 steps). Mp 176–180 °C; ^1^H NMR (500 MHz, CD_3_CN): *δ* 7.95 (br s, 1H), 6.89 (dd, *J* = 8.2, 8.2 Hz, 1H), 6.42 (dd, *J* = 8.2, 1.0 Hz, 1H), 6.38 (br d, *J* = 8.2 Hz, 1H), 4.80 (br s, 1H), 3.82 (s, 3H), 2.12 – 2.05 (m, 2H), 1.81 – 1.57 (m, 6H). ^13^C-NMR (126 MHz, CD_3_CN): *δ* 171.0, 147.2, 135.5, 123.8, 116.0, 108.4, 102.5, 66.4, 56.4, 37.4, 25.3. LRMS (ESI) *m*/*z* [M+H]^+^ 233. RP-HPLC: RT 11.53 min; purity: 98.9%. 

#### 3.2.25. Methyl 3′-oxo-3′,4′-dihydro-1′*H*-spiro[cyclopentane-1,2′-quinoxaline]-5′-carboxylate (**38**)

A suspension of methyl 3-fluoro-2-nitrobenzoate (205 mg g, 1.03 mmol, 1.03 eq), methyl 1-aminocyclopentane-1-carboxylate (180 mg, 1.00 mmol, 1.00 eq) and potassium carbonate (300 mg, 2.51 mmol, 2.51 eq) in DMF (1.8 mL) was heated to 80 °C for 6 d. The dark brown reaction mixture was diluted with EtOAc (50 mL), water (5 mL) and brine (30 mL). The organic phase was washed with brine (25 mL), 0.5 M LiCl solution (20 mL) and brine (15 mL), dried (Na_2_SO_4_), filtered and concentrated in vacuo. The residue was purified by silica column (40 g) eluting with 0–15 % EtOAc in cyclohexane. The impure product fractions were pooled, evaporated and purified over RP column (C_18_, 250/21 mm HPLC) eluting with a gradient of 10 to 100% B in A (A: 95/5 water/MeCN, B: 5/95 water/MeCN) to afford a yellow solid (65 mg). A suspension of the intermediate (0.060 g, 0.186 mmol, 1 eq), NH_4_Cl (0.270 g, 5.05 mmol, 27 eq) and Zn (0.304 g, 4.65 mmol, 25 eq) in DMF (2 mL) was stirred overnight. The reaction mixture was diluted with EtOAc (50 mL), filtered and the filter cake was rinsed with EtOAc (2 × 10 mL). The organic layer was washed with water (2 × 20 mL), 0.5 M LiCl solution (10 mL) and brine (10 mL), dried (Na_2_SO_4_), filtered and evaporated in vacuo. The residue was purified by a RP column (21 × 150, C_18_) eluting with a gradient 10–100% B in A (A: 95/5 H_2_O/MeCN, B: 5/95 H_2_O/MeCN) to afford an orange solid of **38** (13 mg, 0.0499 mmol, 5% over two steps). Mp 147–149 °C; ^1^H NMR (500 MHz, CD_3_CN): *δ* 10.16 (br s, 1H), 7.38 (dd, *J* = 7.9, 1.4 Hz, 1H), 6.98–6.94 (m, 1H), 6.90 (dd, *J* = 7.9, 7.9 Hz, 1H), 5.06 (br s, 1H), 3.87 (s, 3H), 2.15–2.07 (m, 2H), 1.83–1.60 (m, 6H). ^13^C-NMR (126 MHz, CD_3_CN): *δ* 172.0, 169.0, 135.5, 129.7, 122.9, 121.1, 119.5, 113.8, 66.0, 52.8, 37.7, 25.3. LRMS (ESI) *m*/*z* [M+H]^+^ 261. RP-HPLC: RT 11.03 min; purity: 99.9%. 

#### 3.2.26. 7′-Methoxy-3′,4′-dihydro-1′*H*-spiro[cyclopentane-1,2′-quinoxalin]-3′-one (**39**)

A suspension of 3-fluoro-4-nitroanisole (185 mg, 1.08 mmol, 1.0 eq), methyl 1-aminocyclopentane-1-carboxylate hydrochloride (186 mg, 1.04 mmol, 1.0 eq) and potassium carbonate (300 mg, 2.51 mmol, 2.4 eq) in DMF (1.8 mL) was heated to 80 °C for 5 d. The dark brown reaction mixture was diluted with EtOAc (75 mL), water (5 mL) and brine (25 mL). The organic phase was washed with brine (20, 15 mL), 0.5 M LiCl (15 mL) solution and brine (15 mL), dried (Na_2_SO_4_), filtered and concentrated in vacuo. The residue was purified by silica column (80 g) chromatography eluting with 0 to 15% EtOAc in cyclohexane. The impure product fractions were pooled, evaporated and purified over RP column (C_18_, 250/21 mm HPLC) eluting with 10–100 B in A (A: 95/5 water/MeCN, B: 5/95 water/MeCN) to afford a yellow solid (0.127 g). This intermediate (0.127 g, 0.432 mmol, 1.0 eq), NH_4_Cl (0.577 g, 10.8 mmol, 25 eq) and Zn (0.705, 10.8 mmol, 25 eq) in DMF (2 mL) was stirred overnight. The reaction mixture was diluted with EtOAc (50 mL), filtered and the filter cake was rinsed with EtOAc (2 × 10 mL). The organic layer was washed with water (2 × 20 mL), 0.5 M LiCl solution (10 mL) and brine (10 mL), dried (Na_2_SO_4_), filtered and evaporated in vacuo. The residue was purified by a RP column (21 × 150, C18) eluting with a gradient 10–100% B in A (A: 95/5 H_2_O/MeCN, B: 5/95 H_2_O/MeCN). The yellowish solid was suspended in MeCN, filtered, the yellow filtrate was discarded, and an off-white solid of **39** was collected (27 mg, 0.116 mmol, 11%). Mp 224–227 °C decomp.; ^1^H-NMR (500 MHz, (CD_3_)_2_SO): *δ* 9.99 (br s, 1H), 6.62 (d, *J* = 8.5 Hz, 1H), 6.31 (d, *J* = 2.7 Hz, 1H), 6.18 (dd, *J* = 8.5, 2.7 Hz, 1H), 6.05 (br s, 1H), 3.63 (s, 3H), 2.01–1.93 (m, 2H), 1.77–1.68 (m, 2H), 1.64–1.48 (m, 4H). ^13^C-NMR (126 MHz, (CD_3_)_2_SO): *δ* 169.8, 155.2, 135.3, 129.4, 114.8, 102.7, 100.0, 64.6, 54.9, 36.4, 24.3. LRMS (ESI) *m*/*z* [M+H]^+^ 233. RP-HPLC: RT 12.56 min; purity: 99.4%.

#### 3.2.27. Methyl 3′-oxo-3′,4′-dihydro-1′*H*-spiro[cyclopentane-1,2′-quinoxaline]-6′-carboxylate (**40**)

A suspension of methyl 3-fluoro-2-nitrobenzoate (205 mg, 1.03 mmol, 1.0 eq), methyl 1-aminocyclopentane-1-carboxylate (187 mg, 1.01 mmol, 1.0 eq) and potassium carbonate (302 mg, 2.53 mmol, 2.5 eq) in DMF (1.8 mL) was heated to 80 °C for 4 d. The dark brown reaction mixture was diluted with EtOAc (75 mL), water (5 mL) and brine (25 mL). The organic phase was washed with brine (20 and 15 mL), 0.5 M LiCl solution (15 mL) and brine (15 mL), dried (Na_2_SO_4_), filtered and concentrated in vacuo. The residue was purified by silica column (40 g) chromatography eluting with 5 to 20 % EtOAc in cyclohexane to afford the desired compound as a yellow solid (198 mg, 0.614 mmol, 61 %). A suspension of this intermediate (0.190 g, 0.589 mmol, 1 eq), NH_4_Cl (0.813 g, 15.2 mmol, 26 eq) and Zn (1.00 g, 15.3 mmol, 26 eq) in DMF (2 mL) was stirred overnight. The reaction mixture was diluted with EtOAc (70 mL), filtered, and washed with water (2 x 20 mL), LiCl (10 mL) and brine (10 mL). The org. layers were dried (Na_2_SO_4_), filtered and evaporated in vacuo. The residue was purified over silica column (40 g) eluting with 10 to 30% EtOAc in cyclohexane to afford a white solid of **40** (30 mg, 0.115 mmol, 11% over two steps). Mp 246–250 °C dec; ^1^H NMR (500 MHz, (CD_3_)_2_SO): *δ* 10.38 (br s, 1H), 7.41 (dd, *J* = 8.3, 1.8 Hz, 1H), 7.36 (d, *J* = 1.8 Hz, 1H), 6.88 (br s, 1H), 6.72 (d, *J* = 8.3 Hz 1H), 3.75 (s, 3H), 2.09–2.01 (m, 2H), 1.81–1.70 (m, 2H), 1.68–1.53 (m, 6H). ^13^C-NMR (126 MHz, (CD_3_)_2_SO): *δ* 169.7, 166.1, 138.7, 125.6, 124.9, 118.1, 115.2, 112.7, 64.6, 51.5, 37.3, 24.5. LRMS (ESI) *m*/*z* [M+H]^+^ 261. RP-HPLC: RT 11.49 min; purity: 99.9%.

#### 3.2.28. 6′-Nitro-3′,4′-dihydro-1′*H*-spiro[cyclopentane-1,2′-quinoxalin]-3′-one (**41**)

A mixture of 1,2-diamino-4-nitrobenzene (0.92 g, 6.00 mmol, 1.0 eq), cyclopentanone (1.35 mL, 15.2 mmol, 2.6 eq), chloroform (1.5 mL, 18.6 mmol, 3.1 eq) benzyltriethylammonium chloride (71 mg, 0.312 mmol, 0.05 eq) in dichloromethane (4 mL) was cooled to 0 °C. A 50% aqueous NaOH solution (1.8 mL, 30.4 mmol, 5.1 eq) was carefully added over 20 min to the red suspension. The reaction was stirred at 0°C for 45 min and then left to warm to ambient temperature and stirred overnight. EtOAc (100 mL) was added to the reddish suspension and the organic layer was washed with brine (3 × 50 mL), dried (Na_2_SO_4_), filtered and concentrated in vacuo. The residue was purified by a silica column (24 g) eluting with a gradient of 0 to 30% EtOAc in cyclohexane to afford **41** as an orange solid (710 mg, 48%). Mp 275–278 °C.; ^1^H NMR (500 MHz, (CD_3_)_2_SO): *δ* 10.84 (br s, 1H), 7.59 (d, *J* = 2.5 Hz, 1H), 7.56 (dd, *J* = 8.5, 2.5 Hz, 1H), 6.88 (d, *J* = 8.5 Hz, 1H), 6.73 (br s, 1H), 2.07–1.99 (m, 2H), 1.81–1.72 (m, 2H), 1.68–1.55 (m, 4H). ^13^C-NMR (126 MHz, (CD_3_)_2_SO): *δ* 170.4, 142.4, 134.3, 132.7, 114.3, 114.1, 108.0, 64.5, 36.8, 24.3. LRMS (ESI) *m*/*z* [M+H]^+^ 248. RP-HPLC: RT 11.62 min; purity: 99.9%.

#### 3.2.29. 1′-(2-Chloroacetyl)-3′,4′-dihydro-1′H-spiro[cyclopentane-1,2′-quinoxalin]-3′-one (**42**)

A solution of **15** (500 mg, 2.48 mmol, 1.0 eq) in DMF (20 mL) was cooled to 0 °C. The 2-chloroacetyl chloride (422 μL, 584 mg, 3.71 mmol, 1.5 eq) was added over 15 min. The reaction mixture was stirred at 0 °C for 30 min, at which point the TLC indicated complete consumption of the starting material. To the reaction mixture was added EtOAc (100 mL) and the organic layer was washed with brine (3 × 100 mL), 0.5 M aq. LiCl (3 × 50 mL), brine (100 mL), dried (Na_2_SO_4_), filtered and concentrated in vacuo. The residue was purified by a SiO_2_ column eluting with a gradient of 10 to 70% EtOAc in cyclohexane to afford a yellowish solid **42** (679 mg, 2.44 mmol, 98%). ^1^H-NMR (500 MHz, (CDCl_3_): *δ* 8.96 (s, 1H), 7.40 (dd, *J* 7.8, 1.3 Hz, 1H), 7.33 (ddd, *J* 7.8, 7.8, 1.3 Hz, 1H), 7.15 (ddd, *J* 7.8, 7.8, 1.3 Hz, 1H), 7.00 (dd, *J* 7.8, 1.3 Hz, 1H), 4.04 (s, 2H), 2.46–2.03 (m, 4H), 1.88–1.72 (m, 4H). ^13^C-NMR (126 MHz, (CDCl_3_): *δ* 173.7, 168.9, 133.8, 128.9, 128.6, 125.5, 123.5, 116.5, 70.6, 43.5 35.1, 24.3. LRMS (ESI) *m*/*z* [M+H]^+^ 279. 

#### 3.2.30. 1′-(2-Azidoacetyl)-3′,4′-dihydro-1′*H*-spiro[cyclopentane-1,2′-quinoxalin]-3′-one (**43**)

A suspension of **42** (490 mg, 1.76 mmol, 1.0 eq), NaN_3_ (457 mg, 7.03 mmol, 4.0 eq) in DMF (10 mL) was stirred at 80 °C for 30 min, at which point the TLC indicated complete consumption of the starting material. To the reaction mixture was added EtOAc (100 mL) and the organic layer was washed with brine (3 × 100 mL), 0.5 M aq. LiCl (3 × 50 mL), brine (100 mL), dried (Na_2_SO_4_), filtered and concentrated in vacuo. The residue was purified on a SiO_2_ column eluting with a gradient of 10 to 70% EtOAc in cyclohexane to afford a colorless solid of **43** (390 mg, 1.37 mmol, 78%). ^1^H-NMR (500 MHz, CDCl_3_: *δ* 9.28 (s, 1H), 7.32 (ddd, *J* 7.9, 7.9, 1.1 Hz, 1H), 7.25 (dd, *J* 7.9, 1.1 Hz, 1H), 7.10 (ddd, *J* 7.9, 7.9, 1.1 Hz, 1H), 7.04 (dd, *J* 7.9, 1.1 Hz, 1H) 3.79 (s, 2H), 2.49–2.03 (br m, 4H), 1.86–1.49 (br m, 4H). ^13^C-NMR (126 MHz, (CDCl_3_): *δ* 173.9, 170.3, 133.7, 128.5, 128.2, 125.6, 123.2, 116.5, 70.3, 70.3, 52.1, 35.1, 24.1. LRMS (ES) *m*/*z* [M+H]^+^ 286. 

#### 3.2.31. 1′-[2-(4-Phenyl-1*H*-1,2,3-triazol-1-yl)acetyl]-3′,4′-dihydro-1′*H*-spiro[cyclopentane-1,2′-quinoxalin]-3′-one (**44**)

The reaction was performed analogous to the general procedure B. The product was purified on a SiO_2_ column eluting with a gradient of 30 to 100% EtOAc in cyclohexane to afford a colorless solid of **44** (15.3 mg, 0.039 mmol, 23%). Mp 256 °C decomp.; ^1^H-NMR (500 MHz, (CD_3_)_2_SO): *δ* 10.58 (s, 1H), 8.41 (s, 1H), 7.84–7.80 (m, 2H), 7.76 (dd, *J* 7.9, 1.1, 1H), 7.46–7.41 (m, 2H), 7.37 (ddd, *J* = 7.6, 7.6, 1.1 Hz, 1H), 7.33 (tt, *J =* 7.3, 0.9 Hz, 1H), 7.15 (ddd, *J =* 7.6, 7.6 Hz, 1.1, 1H), 7.08 (dd, *J =* 8.1, 1.1 Hz, 1H), 5.32 (br s, 2H), 2.28–1.85 (br m, 4H), 1.75–1.51 (br m, 4H). ^13^C–NMR (126 MHz, (CD_3_)_2_SO): *δ* 172.2, 168.5, 146.5, 134.8, 131.2, 129.4, 128.9, 128.3, 128.1, 126.7, 125.5, 123.6, 123.0, 116.5, 69.6, 52.7, 35.4, 24.1. LRMS (ESI) *m*/*z* [M+H]^+^ 388. RP-HPLC: RT 12.10 min; purity: 97.7%.

#### 3.2.32. 1′-[2-(4-Cyclohexyl-1*H*-1,2,3-triazol-1-yl)acetyl]-3′,4′-dihydro-1′*H*-spiro[cyclopentane-1,2′-quinoxalin]-3′-one (**45**)

The reaction was performed analogous to the general procedure B. The product was purified on a SiO_2_ column eluting with a gradient of 20 to 80% EtOAc in cyclohexane to afford a colorless solid of **45** (60.7 mg, 0.154 mmol, 63%). Mp 205 °C decomp.; ^1^H-NMR (500 MHz, (CD_3_)_2_SO): *δ* 10.54 (s, 1H), 7.69 (dd, *J =* 8.0, 1.0 Hz, 1H), 7.65 (s, 1H), 7.34 (ddd, *J =* 8.0, 8.0, 1.0 Hz, 1H), 7.12 (ddd, *J =* 7.9, 7.9, 1.1 Hz, 1H), 7.06 (dd, *J =* 7.9, 1.1 Hz, 1H), 5.18 (br s, 2H), 2.64 (m, 1H), 2.26–1.80 (br m, 6H), 1.74–1.49 (br m, 8H), 1.40–1.26 (m, 4H). ^13^C-NMR (126 MHz, (CD_3_)_2_SO): *δ* 172.2, 168.7, 152.1, 134.8, 128.8 128.2, 126.6, 122.9, 122.7, 116.4, 69.6, 52.4, 42.1, 35.0, 33.0, 26.1, 26.0, 24.0. LRMS (ESI) *m*/*z* [M+H]^+^ 394. RP-HPLC: RT 12.76 min; purity: 98.9%.

#### 3.2.33. 1′-[2-(4-Benzyl-1*H*-1,2,3-triazol-1-yl)acetyl]-3′,4′-dihydro-1′*H*-spiro[cyclopentane-1,2′-quinoxalin]-3′-one (**46**)

The reaction was performed analogous to the general procedure B. The product was purified on a semi-prep reversed-phase HPLC eluting with a gradient of 10 to 100% MeCN in H_2_O, but there were still some minor impurities. A second purification on a SiO_2_ column eluting with a gradient of 20 to 80% EtOAc in cyclohexane afforded a colorless solid of **46** (38.0 mg, 0.095 mmol, 39%). Mp 203 °C decomp.; ^1^H-NMR (500 MHz, (CD_3_)_2_SO): *δ* 10.53 (s, 1H), 7.70–7.64 (m, 2H), 7.33 (ddd, *J =* 7.8, 7.8, 1.4 Hz, 1H), 7.31–7.26 (m, 2H), 7.25–7.17 (m, 3H), 7.11 (ddd, *J =* 7.9, 7.9, 1.1 Hz, 1H), 7.07 (dd, *J =* 7.9, 1.1 Hz, 1H), 5.20 (br s, 2H), 3.97 (s, 2H), 2.26–1.80 (br m, 4H), 1.74–1.49 (br m, 4H). ^13^C-NMR (126 MHz, (CD_3_)_2_SO): *δ* 172.2, 168.7, 146.1, 140.0, 134.8, 129.0, 128.84, 128.77, 128.2, 124.7, 122.9, 116.4, 69.6, 52.4, 31.7, 24.0. LRMS (ESI) *m*/*z* [M+H]^+^ 402. RP-HPLC: RT 12.23 min; purity: 95.3%.

#### 3.2.34. 3-[1-(2-Oxo-2-{3′-oxo-3′,4′-dihydro-1′*H*-spiro[cyclopentane-1,2′-quinoxalin]-1′-yl}ethyl)-1*H*-1,2,3-triazol-4-yl]benzoic acid (**47**)

The reaction was performed analogous to the general procedure B. The product was purified on a C_18_ column eluting with a gradient of 10 to 100% MeCN in H_2_O containing 0.2% AcOH to afford the colorless solid of **47** (24.3 mg, 0.056 mmol, 23%). Mp 272 °C decomp.; ^1^H-NMR (500 MHz, (CD_3_)_2_SO): *δ* 12.16 (br s, 1H), 10.58 (s, 1H), 8.54 (s, 1H), 8.40 (dd, *J =* 1.6, 1.6 Hz, 1H), 8.04 (ddd, *J =* 7.8, 1.6, 1.6 Hz, 1H), 7.92 (ddd, *J =* 7.8, 1.6, 1.6 Hz, 1H), 7.76 (dd, *J* 8.0, 1.1, 1H), 7.57 (dd, *J =* 7.8, 7.8 Hz, 1H), 7.37 (ddd, *J =* 8.0, 8.0, 1.1 Hz, 1H), 7.16 (ddd, *J =* 8.0, 8.0, 1.1 Hz, 1H), 7.08 (dd, *J =* 8.0, 1.1 Hz, 1H), 5.25 (br s, 2H), 2.16–1.84 (br m, 4H), 1.74–1.52 (br m, 4H); ^13^C-NMR (126 MHz, (CD_3_)_2_SO): *δ* 172.2, 168.4, 167.6, 145.7, 134.8, 131.5, 129.7, 129.0, 128.9, 128.1, 126.6, 126.2, 124.1, 123.0, 116.5, 69.7, 52.8, 43.4, 24.1. LRMS (ESI) *m*/*z* [M+H]^+^ 432. RP-HPLC: RT 11.80 min; purity: 99.2%.

#### 3.2.35. Methyl 4-[1-(2-oxo-2-{3′-oxo-3′,4′-dihydro-1′*H*-spiro[cyclopentane-1,2′-quinoxalin]-1′-yl}ethyl)-1*H*-1,2,3-triazol-4-yl]benzoate (**48**)

The reaction was performed analogous to the general procedure B. The product was purified on a SiO_2_ column eluting with a gradient of 20 to 90% EtOAc in cyclohexane to afford a colorless solid of **48** (60.7 mg, 0.136 mmol, 56%). Mp 263 °C decomp.; ^1^H-NMR (500 MHz, (CD_3_)_2_SO): *δ* 10.59 (s, 1H), 8.57 (s, 1H), 8.05–8.01 (m, 2H), 8.01–7.96 (m, 2H) 7.77 (dd, *J =* 8.0, 1.0 Hz, 1H), 7.37 (ddd, *J =* 8.0, 8.0, 1.0 Hz, 1H), 7.15 (ddd, *J =* 8.0, 8.0, 1.0 Hz, 1H), 7.08 (dd, *J =* 8.0, 1.0 Hz, 1H), 5.36 (br s, 2H), 3.87 (s, 3H), 2.26–1.80 (br m, 4H), 1.74–1.49 (br m, 4H); ^13^C-NMR (126 MHz, (CD_3_)_2_SO): *δ* 172.2, 168.4, 166.4, 145.4, 135.7, 134.8, 130.4, 129.1, 128.9, 128.1, 126.5, 125.6, 124.9, 123.0, 116.5, 69.7, 52.9, 52.6, 44.0, 23.8. LRMS (ESI) *m*/*z* [M+H]^+^ 446. RP-HPLC: RT 12.22 min; purity: 95.2%. 

#### 3.2.36. 1′-{2-[4-(4-Methoxyphenyl)-1*H*-1,2,3-triazol-1-yl]acetyl}-3′,4′-dihydro-1′*H*-spiro[cyclopentane-1,2′-quinoxalin]-3′-one (**49**)

The reaction was performed analogous to the general procedure B. The product was purified on a SiO_2_ column eluting with a gradient of 30 to 100% EtOAc in cyclohexane to afford a colorless solid of **49** (34.0 mg, 0.082 mmol, 47%). Mp 249 °C decomp.; ^1^H-NMR (500 MHz, (CD_3_)_2_SO): *δ* 10.58 (s, 1H), 8.29 (s, 1H), 7.78–7.72 (m, 3H), 7.37 (ddd, *J =* 7.9, 7.9, 1.1 Hz, 1H), 7.15 (ddd, *J =* 7.9, 7.9, 1.1 Hz, 1H), 7.08 (dd, *J =* 7.9, 1.1 Hz, 1H), 7.03–6.98 (m, 2H), 5.31 (br s, 2H), 3.79 (s, 3H), 2.28–1.86 (br m, 4H), 1.75–1.52 (br m, 4H). ^13^C-NMR (126 MHz, (CD_3_)_2_SO): *δ* 172.2, 168.6, 159.4, 146.4, 134.8, 128.9, 128.1, 126.9, 126.7, 123.8, 123.0, 122.6, 116.5, 114.8, 69.6, 55.6, 52.7. LRMS (ESI) *m*/*z* [M+H]^+^ 418. RP-HPLC: RT 12.09 min; purity: 98.0%.

#### 3.2.37. 1′-{2-[4-(Pyridin-2-yl)-1*H*-1,2,3-triazol-1-yl]acetyl}-3′,4′-dihydro-1′*H*-spiro[cyclopentane-1,2′-quinoxalin]-3′-one (**50**)

The reaction was performed analogous to the general procedure B. The residue was purified on a reversed phase C_18_ column eluting with a gradient of 10 to 100% MeCN in H_2_O to afford a colorless solid of **50** (10.2 mg, 0.026 mmol, 15%). Mp 218 °C decomp.; ^1^H-NMR (500 MHz, (CD_3_)_2_CO): *δ* 9.53 (s, 1H), 8.58–8.55 (m, 1H), 8.35 (s, 1H), 8.10 (dt, *J =* 7.6, 1.1, 1.1 Hz, 1H), 7.86 (ddd, *J =* 7.6, 7.6, 1.8 Hz, 1H) 7.78 (dd, *J =* 7.8, 1.2 Hz, 1H), 7.41 (ddd, *J =* 7.9, 7.9, 1.1 Hz, 1H), 7.29 (ddd, *J =* 7.7, 4.7, 1.2 Hz, 1H), 7.26–7.20 (m, 2H), 5.40 (br s, 2H), 3.65–3.34 (br m, 4H), 1.81–1.60 (br m, 4H). ^13^C-NMR (126 MHz, (CD_3_)_2_CO): *δ* 171.9, 167.9, 150.8, 149.6, 147.9, 136.7, 135.2, 128.5, 128.2, 126.4, 124.5, 122.6, 122.5, 119.3, 116.1, 69.9, 52.5, 34.8, 23.7. LRMS (ESI) *m*/*z* [M+H]^+^ 389. RP-HPLC: RT 11.49 min; purity: 99.3%.

#### 3.2.38. 1′-[2-(4-Cyclopropyl-1*H*-1,2,3-triazol-1-yl)acetyl]-3′,4′-dihydro-1′*H*-spiro[cyclopentane-1,2′-quinoxalin]-3′-one (**51**)

The reaction was performed analogous to the general procedure B. The residue was purified on a reversed phase C_18_ column eluting with a gradient of 10 to 100% MeCN in H_2_O to afford a colorless oil of **51** (14.2 mg, 0.040 mmol, 23%). ^1^H-NMR (500 MHz, (CD_3_)_2_CO): *δ* 9.49 (s, 1H), 7.72 (dd, *J =* 7.8, 1.2 Hz, 1H), 7.53 (s, 1H), 7.40 (ddd, *J =* 7.9, 7.9, 1.1 Hz, 1H), 7.23–7.16 (m, 2H), 5.20 (br s, 2H), 2.24–1.95 (br m, 4H), 1.96–1.85 (m, 1H), 1.79–1.60 (br m, 4H), 0.91–0.85 (m, 2H), 0.77–0.73 (m, 2H). ^13^C-NMR (126 MHz, (CD_3_)_2_CO): *δ* 171.8, 168.2, 149.0, 134.9, 128.4, 126.4, 122.6, 121.9, 116.0, 116.0, 69.8, 52.2, 34.6, 23.7, 7.0, 6.4. LRMS (ESI) *m*/*z* [M+H]^+^ 352. RP-HPLC: RT 11.45 min; purity: 97.1%.

#### 3.2.39. 2-[1-(2-Oxo-2-{3′-oxo-3′,4′-dihydro-1′*H*-spiro[cyclopentane-1,2′-quinoxalin]-1′-yl}ethyl)-1*H*-1,2,3-triazol-4-yl]acetic acid (**52**)

The reaction was performed analogous to the general procedure B. The product was purified on a semi-prep reversed phase HPLC eluting with a gradient of 10 to 100% MeCN in H_2_O to afford a colorless solid of **52** (13.6 mg, 0.035 mmol, 20%). Mp 230 °C decomp.; ^1^H-NMR (500 MHz, (CD_3_)_2_SO): *δ* 10.58 (s, 1H), 8.62 (s, 1H), 7.73 (dd, *J =* 7.9, 1.1 Hz, 1H), 7.36 (ddd, *J =* 7.9, 7.9, 1.1 Hz, 1H), 7.14 (ddd, *J =* 7.9, 7.9, 1.1 Hz, 1H), 7.07 (dd, *J =* 7.9, 1.1 Hz, 1H), 5.35 (br s, 2H), 3.82 (s, 2H), 2.26–1.80 (br m, 4H), 1.74–1.50 (br m, 4H). ^13^C-NMR (126 MHz, (CD_3_)_2_SO): *δ* 172.1, 168.0, 161.1, 138.7, 134.9, 131.4, 128.9, 127.9, 126.5, 123.0, 116.5, 69.7, 53.0, 52.3, 35.1, 23.7. LRMS (ESI) *m*/*z* [M+H]^+^ 370. RP-HPLC: RT 10.98 min; purity: 94.1%.

#### 3.2.40. 1′-{2-[4-(2-Methoxyethyl)-1*H*-1,2,3-triazol-1-yl]acetyl}-3′,4′-dihydro-1′*H*-spiro[cyclopentane-1,2′-quinoxalin]-3′-one (**53**)

The reaction was performed analogous to the general procedure B. The residue was purified on a reversed phase C_18_ column eluting with a gradient of 10 to 100% MeCN in H_2_O to afford a colorless solid of **53** (24.3 mg, 0.066 mmol, 38%). Mp 153 °C decomp.; ^1^H-NMR (500 MHz, (CD_3_)_2_CO): *δ* 9.50 (s, 1H), 7.80 (s, 1H), 7.76–7.72 (m, 1H), 7.41 (ddd, *J =* 7.9, 7.9, 1.1 Hz, 1H), 7.23–7.17 (m, 2H), 5.29 (br s, 2H), 4.48–4.46 (m, 2H), 3.30 (s, 3H), 2.81–2.77 (m, 2H), 2.20–1.90 (br m, 4H), 1.78–1.61 (br m, 4H). ^13^C–NMR (126 MHz, (CD_3_)_2_CO): *δ* 171.8, 168.1, 144.2, 134.9, 128.5, 128.2, 126.4, 124.9, 122.6, 116.1, 116.0, 69.8, 65.4, 56.9, 52.2, 34.9, 23.6. LRMS (ESI) *m*/*z* [M+H]^+^. RP-HPLC: RT 10.88 min; purity: 99.1%.

#### 3.2.41. 1′-[2-(5-Phenyl-1*H*-1,2,3,4-tetrazol-1-yl)acetyl]-3′,4′-dihydro-1′*H*-spiro[cyclopentane-1,2′-quinoxalin]-3′-one (**54**)

**22** (74.4 mg, 0.364 mmol, 1.2 eq) was dissolved in dichloromethane (0.1 mL) and excess SOCl_2_ (1 mL) was added. The SOCl_2_ solution was stirred at 45 °C for 1 h before the solvent and the SOCl_2_ was evaporated in vacuo. The residue was dissolved in dichloromethane (1 mL), **5** (61.7 mg, 0.304 mmol, 1.0 eq), and Et_3_N (0.05 mL, 0.361 mmol, 1.2 eq) was added and the reaction was stirred at ambient temperature for 2 h. MeOH (0.5 mL) was added and the reaction mixture was evaporated. The residue was purified by a silica column eluting with a gradient of 0 to 50% EtOAc in cyclohexane to afford a colorless solid of **54** (16.5 mg, 0.042 mmol, 14%). Mp 197–202 °C decomp.; ^1^H-NMR (500 MHz, CDCl_3_): *δ* 8.92 (br s, 1H), 7.48–7.42 (m, 3H), 7.41–7.36 (m, 2H), 7.31–7.28 (m, 2H), 7.05 (ddd, *J* = 7.8, 7.8, 1.2 Hz, 1H), 6.97 (dd, *J* = 7.8, 1.2 Hz, 1H), 5.03 (br s, 2H), 2.05 (m, 2H), 1.70 (m, 4H), 1.31–1.15 (m, 2H). ^13^C-NMR (126 MHz, CDCl_3_): *δ* 173.4, 166.9, 155.3, 134.0, 131.6, 129.34, 129.32, 128.7, 127.8, 125.9, 123.7, 123.6, 116.8, 70.9, 50.5, 29.7, 24.1. RP-HPLC: RT 11.58 min; purity: 99.9%.

#### 3.2.42. 1′-[2-(5-Phenyl-2*H*-1,2,3,4-tetrazol-2-yl)acetyl]-3′,4′-dihydro-1′*H*-spiro[cyclopentane-1,2′-thieno [3,4-b]pyrazin]-3′-one (**55**)

To a solution of **21** (125 mg, 0.623 mmol, 1.0 eq) in dichloromethane (1.5 mL) was added oxalyl chloride (0.06 mL, 0.699 mmol, 1.1 eq) followed by a drop of DMF. When the gas evolution stopped, the acid chloride solution was added to a solution of **35** (152 mg, 0.735 mmol, 1.2 eq) in dichloromethane (1.5 mL) and the reaction stirred at ambient temperature overnight. The reaction mixture was diluted with EtOAc (100 mL) and sat. aq. NaHCO_3_ solution (40 mL). The layers were separated, and the org. layer was washed with and brine (20 mL), dried (Na_2_SO_4_), filtered and evaporated in vacuo. The residue was purified by silica column eluting with 10 to 35% EtOAc in cyclohexane followed by semi-prep RP chromatography (C_18_ column) eluting with a gradient of 10 to 100% B in A (A: 95/5 water/MeCN, B: 5/95 water/MeCN) to afford **55** as an off-white solid (16 mg, 0.041 mmol, 7%). Mp 231–233°°C dec; ^1^H-NMR (500 MHz, (CD_3_)CO): *δ* 9.63 (br s, 1H), 8.13–8.07 (m, 2H), 7.82 (d, *J* = 3.2 Hz, 1H), 7.58–7.49 (m, 3H), 6.85 (d, *J* = 3.2 Hz, 1H), 5.88 (s, 2H, 6), 2.29–2.14 (m, 4H), 1.81–1.63 (m, 4H). ^13^C-NMR (126 MHz, (CD_3_)CO): *δ* 170.4, 166.8, 165.6, 134.1,131.2, 130.3, 129.9, 128.5, 127.4, 118.2, 103.8, 72.7, 56.4, 36.0, 24.4. LRMS (ESI) *m*/*z* [M+H]^+^ 395. RP-HPLC: RT 12.49 min; purity: 99.5%.

#### 3.2.43. 1′-{3-[3-(But-3-yn-1-yl)-3*H*-diazirin-3-yl]propanoyl}-3′,4′-dihydro-1′*H*-spiro[cyclopentane-1,2′-quinoxalin]-3′-one (**56**)

**S15** (40 mg, 0.241 mmol, 1.0 eq) was dissolved in thionyl chloride (2 mL) and stirred for 15 min at 40 °C. Thionyl chloride was removed in vacuo. The residue was taken up in dichloromethane (2 mL) and added dropwise to a solution of **5** (58.4 mg, 0.289 mmol, 1.2 eq), Et_3_N (73 mg, 0.722 mmol, 3.0 eq) and dichloromethane (2 mL) and stirred for 1 h. The reaction mixture was concentrated in vacuo and purified by semi-prep HPLC on a C_18_ column eluting with a gradient of 10 to 100% MeCN in H_2_O containing 0.2% AcOH to afford a pale yellow oil of **56** (20.7 mg, 0.059 mmol, 25%). ^1^H-NMR (500 MHz, CDCl_3_): *δ* 8.81 (s, 1H), 7.31–7.25 (m, 2H), 7.12–7.08 (m, 1H), 6.99–6.95 (m, 1H), 2.20–1.81 (m, 8H), 1.79–1.65 (m, 7H), 1.59–1.53 (m, 2H). ^13^C-NMR (126 MHz, CDCl_3_): *δ* 174.5, 174.2, 133.9, 129.8, 128.0, 126.6, 123.0, 116.1, 82.7, 69.5, 69.1, 34.9, 32.4, 29.8, 27.9, 27.7, 23.7, 13.2. LRMS (ESI) *m*/*z* [M+H]^+^ 351. RP-HPLC: RT 13.34 min; purity: 92.6%.

#### 3.2.44. 1′-Octanoyl-3′,4′-dihydro-1′*H*-spiro[cyclopentane-1,2′-quinoxalin]-3′-one (**57**)

To a solution of octanoic acid (0.09 mL, 0.566 mmol, 1.2 eq) and oxalyl chloride (0.05 mL, 0.582 mmol, 1.2 eq) in dichloromethane (2 mL) was added one drop of DMF (approximately 5 μL, 0.130 mmol, 0.13 eq), resulting in a strong gas evolution. After 5 min, gas formation was complete and **5** (97 mg, 0.480 mmol, 1 eq) and Et_3_N (0.1 mL, 0.717 mmol, 1.5 eq) were added. The reaction was stirred at ambient temperature for 10 min and then quenched with brine (5 mL). The layers were separated, and the aq. layer was extracted with dichloromethane (2 × 5 mL). The org. layers were dried (Na_2_SO_4_), filtered and evaporated in vacuo. The crude product was purified by silica column eluting with a gradient of 0 to 10% MeOH in dichloromethane and semi-prep RP chromatography (C_18_ column) eluting with a gradient of 40 to 100% B in A (A: 95/5 water/MeCN + 0.2% AcOH, B: 5/95 water/MeCN + 0.2% AcOH) to afford an off-white solid of **57** (24 mg, 0.073 mmol, 15%). Mp 67–70 °C; ^1^H-NMR (500 MHz, CDCl_3_): *δ* 9.23 (br s, 1H), 7.29 (d, *J* = 7.8 Hz, 1H), 7.23 (ddd, *J* = 7.8, 7.8, 1.3 Hz, 1H), 7.05 (ddd, *J* = 7.8, 7.8, 1.3 Hz, 1H), 6.97 (d, *J* = 7.8 Hz, 1H) 2.72–1.79 (m, 6H), 1.79–1.61 (m, 4H), 1.49–1.39 (m, 2H), 1.27–1.08 (m, 8H), 0.82 (t, *J* = 7.1 Hz, 3H).^13^C–NMR (126 MHz, CDCl_3_): *δ* 176.9, 174.9, 134.1, 130.4, 127.7, 126.7, 122.8, 116.2, 69.4, 36.0, 35.4, 31.7, 29.2, 29.1, 25.2, 24.4, 22.7, 14.2.LRMS (ESI) *m*/*z* [M+H]^+^ 329. RP-HPLC: RT 14.46 min; purity: 99.9%.

### 3.3. Biological Assays

#### 3.3.1. Fluorogenic SENP1 Assay

IC_50_ measurements were performed with at least two replications using eight concentrations starting at 200 μM and for the fragments at 500 μM with three-fold dilution. Human recombinant, His6-SENP1 Catalytic Domain (Boston Biochem Cat# E-700, Bio-Techne AG, Zug, Switzerland) was prepared in reaction buffer (50 mM HEPES, 150 mM NaCl, 0.5 mM EDTA, 1 mM DTT, pH 7.5, 0.01% CHAPS). To this solution, a 20 mM or 50 mM inhibitor stock solution in DMSO was added and the mixture was incubated for 15 min at ambient temperature. The substrate SUMO1/2 or 3-AMC (SUMO1-AMC; Boston Biochem Cat# UL-551; SUMO2-AMC; Boston Biochem Cat# UL-758; SUMO3-AMC; Boston Biochem Cat# UL-768; Bio-Techne AG, Zug, Switzerland) was delivered to a 96 well-plate to initiate the reaction. The enzyme activities were monitored (Ex/Em = 355/460 nm) as a time-course measurement of the increase in fluorescence signal from SUMO1/2 or 3-AMC for 60 min at 25 °C. The data were analyzed by taking the slope (signal/time) of the linear portion of measurement. The slope was calculated using Excel and the curve fits were performed using GraphPad Prism7 with a four-parameter least-squares fit. The final reaction conditions contained 0.02 nM SENP1 and 300 nM SUMO1/2 or 3-AMC).

#### 3.3.2. Fluorogenic SENP2 Assay

The IC_50_ measurements were conducted at Reaction Biology Corporation, Malvern, PA, USA, with two replications using 10 concentrations starting at 200 μM with a three-fold dilution. Curve fits were performed where the enzyme activities at the highest concentration of compounds were less than 65%. UCH-L3 I was used as a control inhibitor. To SENP2 (Enzo Cat# BML-UW9765, Human recombinant catalytic domain expressed in *E. coli.*) in reaction buffer (50 mM HEPES, 150 mM NaCl, 0.5 mM EDTA, 1 mM DTT, pH 7.5, 0.01% CHAPS) was delivered the compound stock solution by acoustic technology (Echo550; nanoliter range) and subsequently incubated for 15 min at ambient temperature. The substrate SUMO1-AMC (R&D Systems Cat# UL-551-050, Lot# 07613314A, Fluorescence labeled full-length SUMO1 protein) was delivered to the well-plate to initiate the reaction. The enzyme activities were monitored (Ex/Em = 355/460 nm) as a time-course measurement of the increase in fluorescence signal from SUMO1-AMC for 90 min at ambient temperature. The data were analyzed by taking the slope (signal/time) of linear portion of measurement. The slope was calculated using Excel and the curve fits were performed using GraphPad Prism7 with a four-parameter least-squares fit. The final reaction conditions contained 0.015 nM SENP2 and 300 nM SUMO1/2 or 3-AMC.

#### 3.3.3. UCHL1 Activity Assay

IC_50_ measurements were conducted at Reaction Biology Corporation, Malvern, PA, USA, with two replications using 10 concentrations starting at 200 μM with three-fold dilution. Curve fits were performed where the enzyme activities at the highest concentration of compounds were less than 65%. UCHL1 II was used as a control inhibitor. The procedure was performed analogous to the fluorogenic SENP2 assay described in 3.3.2. using the UCHL1 enzyme (SignalChem Cat# U22-30H) and Ubiquitin-AMC substrate (Enzo Cat# BML-SE211-0025). The final reaction conditions contained 0.2 nM UCHL1 and 100 nM Ubiquitin-AMC.

#### 3.3.4. Ataxin-3 Activity Assay

IC50 measurements were conducted at Reaction Biology Corporation, Malvern, PA, USA, with two replications using 10 concentrations starting at 200 μM with three-fold dilution. Curve fits were performed where the enzyme activities at the highest concentration of compounds were less than 65%. UCHL1 II was used as a control inhibitor. The procedure was performed analogous to the fluorogenic SENP2 assay described in 3.3.2. using the Ataxin-3 enzyme (BPS Cat# 80399) and Ubiquitin-AMC substrate (Enzo Cat# BML-SE211-0025). The final reaction conditions contained 500 nM Ataxin-3 and 100 nM Ubiquitin-AMC.

#### 3.3.5. SENP1 Endopeptidase Activity with proSUMO3

Measurements were performed using six different inhibitor concentrations starting at 200 μM of **11** with three-fold dilution. Human recombinant, His6-SENP1 Catalytic Domain (Boston Biochem Cat# E-700, Bio-Techne AG, Zug, Switzerland) was prepared in reaction buffer (50 mM HEPES, 150 mM NaCl 0.5 mM EDTA, 1 mM DTT, pH 7.5, 0.01% CHAPS) as 555 nM solution. A 20 mM compound DMSO stock-solution (1.1 µL) was added to the freshly prepared 555 nM enzyme solution (98.9 µL). Five sequential three-fold dilutions resulted in a final inhibitor concentration of 0.8 µM and the enzyme inhibitor complex was incubated for 15 min at 0 °C. Then, 50 µM proSUMO3 (5 µL) was added to the freshly prepared enzyme inhibitor solution (45 µL) and incubated for 60 min at 37 °C and subsequently the reaction was stopped by adding 4 × Laemmli-Buffer (16.7 µL) and denaturing the protein at 100 °C for 30 min. Those solutions (10 µL) were loaded on a casted 15% sodium dodecyl sulfate polyacrylamide gel and separated on a Mini-PROTEAN Tetra Cell (Bio-Rad, Hercules, CA, USA). After SDS-PAGE gel electrophoresis separation, the gels were stained with staining solution Bio-Safe Coomassie Brilliant Blue G-250 (BioRad) for 60 min. De-staining was performed with 40% (*v*/*v*) methanol, 10% (*v*/*v*) glacial acetic acid, 50% (*v*/*v*) Milli-Q water until protein bands became visible.

#### 3.3.6. Crosslinking Experiments

A buffer exchange of the commercially available 6 × His-SENP1 Protein (Boston Biochem Cat# E-700) to a 50 mM HEPES, 150 mM NaCl; 0.5 mM EDTA, 1 mM DTT, pH 7.5 was performed. A total of 200 μM of the photo affinity probe **56** with or without competitor molecule **11** (400 μM) was incubated in 10 uM SENP1 for 30 min at 0 °C. Subsequently, the mixtures were irradiated in a Proma UV exposure unit equipped with four 8 Watt lamps (Hitachi FL8BL-8, ~350 nm) for 30 min at 0 °C. Then, 50 µL of the crosslinked proteins were mixed with freshly prepared click mix solution (50 μL; 1 mM CuSO_4_, 1 mM TCEP, 100 uM THPTA from a 5 mM DMSO stock, dissolved in Milli-Q Water) and a 10 mM stock solution of azide fluor 545 (1 μL) and incubated at 0 °C for 14 h. A pre-casted gradient sodium dodecyl sulfate polyacrylamide gel (4–20% Mini-PROTEAN^®^ TGX™ Precast Protein Gel, 10-well, 50 µL #4561094) was used. Sodium dodecyl sulfate polyacrylamide gel electrophoresis (SDS-PAGE) was performed using Mini-PROTEAN Tetra Cell (Bio-Rad) using a Tris-Glycine discontinuous buffer system. Protein samples for SDS-PAGE were first prepared in 1 × Laemmli sample buffer (diluted from 4 × Laemmli sample buffer (ROTI^®^Load), and subsequently denatured at 100 °C for 15 min. After SDS-PAGE gel electrophoresis separation, the fluorescence was visualized (Ex. 520 nm, Em. 575 nm) on the SDS PAGE gel using ImageQuant^TM^ 4000 LAS (GE Healthcare, Chicago, IL, USA). Secondly, the gels were stained with Bio-Safe Coomassie Brilliant Blue G-250 (BioRad) for 60 min. De-staining was performed with 40% (*v*/*v*) methanol, 10% (*v*/*v*) glacial acetic acid, 50% (*v*/*v*) Milli-Q water until protein bands became visible.

## 4. Conclusions

Building on a virtual screening campaign, a series of novel SENP inhibitors could be established by fragment-based and synthetic modifications of the screening hit. It was also shown that our inhibitor **11** (ZHAWOC8697) is not only able to inhibit the cleavage of the SUMO1-AMC test substrate, but more importantly the maturation of the native proSUMO protein. The SENP1-inhibitor interaction was further validated with the photo affinity probe **56**. Under our experimental conditions, compound **11** is equipotent compared to the most potent SENP1 inhibitors known to us, published by Chen et al. [21] and Lindemann et al. [25]. Moreover, the selectivity profile of the developed inhibitor toward other DUB proteins is comparable to that of the aforementioned inhibitors. In addition, **11** also inhibits SENP2 very effectively. Thus, this compound represents a valuable small molecule tool to study SENP1/2-SUMO interactions in a biological context and to develop small molecule drugs for the treatment of SENP1/2-associated diseases.

## Figures and Tables

**Table 1:** IC_50_ values of hit compound **11** and the corresponding modifications of the spiro DHQ scaffold **23** and **24**; ^(a)^ n = 5; ^(b)^ n = 2.

Compound	Structure		IC_50_ SENP1 (µM)
**11**	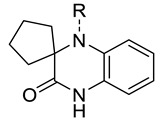	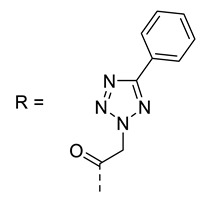	8.6 (6.3–12) ^(a)^
**23**	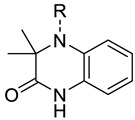		12.3 (9.2–16.5) ^(b)^
**24**	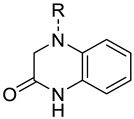		77.6 (57.8–104) ^(b)^

**Table 2:** List of the synthesized fragments (**15**, **16**, **25**–**41**). LE = ligand efficiency (calculated: 1.4(-*log*IC_50_)/N) [30]; ^(a)^ n = 4, ^(b)^ n = 2. * Compound is fluorescent at c > 10 μM; NA = not applicable.

Compound	Structure	IC_50_ SENP1 [µM]	LE		Compound	Structure	IC_50_ SENP1 [µM]	LE
**15**	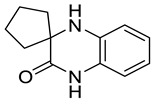	116 (79–170) ^[a]^	0.37		**33**	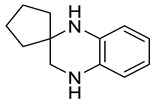	>500 ^[b]^	NA
**16**	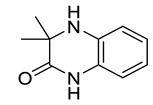	>500 ^[b]^	NA		**34**	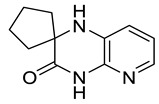	>500 ^[b]^	NA
**25**	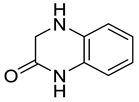	>500 ^[b]^	NA		**35**	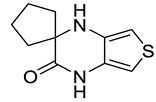	37 ^[b]^ (23–61)	0.44
**26**	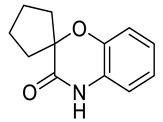	78 ^[b]^ (47–130)	0.38		**36**	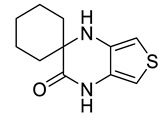	61 ^[b]^ (38–98)	0.39
**27**	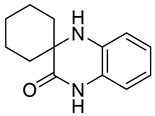	261 ^[b]^ (199–344)	0.31		**37**	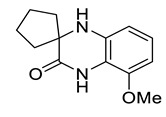	110 ^[b]^ (92–133)	0.34
**28**	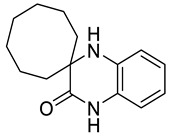	51 ^[b]^ (32–79)	0.33		**38**	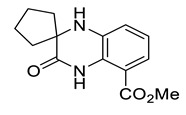	61 ^[b]^ (47–79)	0.33
**29**	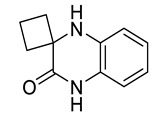	66 ^[b]^ (57–77)	0.42		**39**	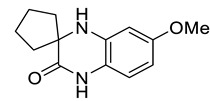	198 ^[b]^ (141–288)	0.32
**30**	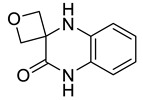	81 ^[b]^ (66–99)	0.41		**40**	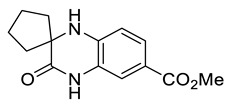	202 ^[b]^ (110–373)	0.28
**31**	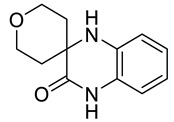	233 ^[b]^ (182–298)	0.32		**41**	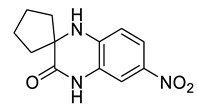	67 ^[b]^ (48–94)	0.34
**32**	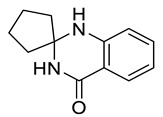	NA * ^[b]^	NA					

**Table 3:** IC_50_ values of **11** and its analogues; ^(a)^ n = 5.

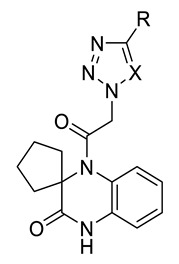			
Compound	X	R	IC_50_ SENP1 (µM) n = 2
**11**	N	Ph	8.6 ^(a)^ (6.3–12)
**44**	CH	Ph	23 (19–27)
**45**	CH	cyclohexyl	19 (17–21)
**46**	CH	benzyl	38 (28–52)
**47**	CH	3-benzoic acid	29 (24–36)
**48**	CH	4-benzoic acid methyl ester	7.5 (6.3–8.9)
**49**	CH	4-methoxyphenyl	13 (9–18)
**50**	CH	2-pyridine	>200
**51**	CH	cyclopropyl	>200
**52**	CH	CH_2_CO_2_H	>100
**53**	CH	(CH_2_)_2_OCH_3_	>100
**54**	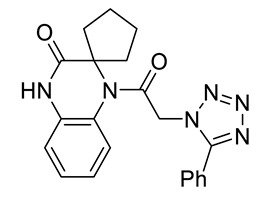		50 (43–57)
**55**	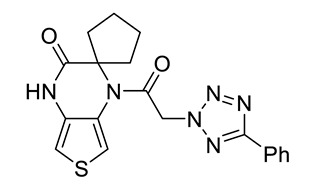		10 (8–13)
**56**	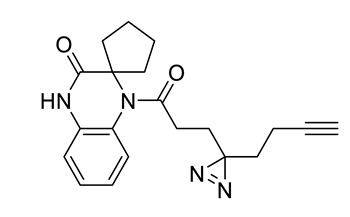		24 (20–29)
**57**	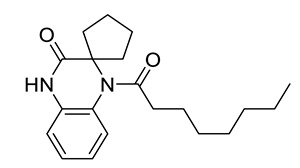		6.6 (5.7–7.7)

**Table 4:** IC_50_ values of **11** (ZHAWOC8697), **55** and published compound **7 [21]**; n = 2.

Compound	SENP1-SUMO1-AMC (µM)	SENP1-SUMO2-AMC (µM)	SENP1-SUMO3-AMC (µM)	SENP2 SUMO1-AMC (µM)	UCHL1 (µM)	Ataxin 3 (µM)
**11**	8.6 (6.3–12)	43 (31–59)	59 (41–86)	2.3 (1.6–3.5)	77 (57–105)	68 (53–89)
**55**	10 (8–13)	18 (15–22)	23 (15–38)	5.8 (3.2–10)	132 (107–162)	>100
**7**	9 (8–11)	4.3 (3.8–5.0)	8.5 (6.4–11)	9.3 (7.2–12)	64 (51–81)	>100

## Data Availability

Data is contained within the article or supplementary material.

## Supplementary Materials

The following supporting information can be downloaded at https://www.mdpi.com/article/10.3390/ijms232012085/s1.
