# Supplementary material for: Discovery of a Dual SENP1 and SENP2 Inhibitor
Source: Int J Mol Sci. 2022 Oct 11;23(20):12085. doi: 10.3390/ijms232012085 (PMC9602571; doi:10.3390/ijms232012085)
Supplement: Supplementary file 1 [file ijms-23-12085-s001.zip › ijms-1936843-supplementary.pdf]

Discovery of a dual SENP1 and SENP2 inhibitor

Michael Brand [1], Elias Benjamin Bommeli [1], Marc Rütimann [1], Urs Lindenmann [1], Rainer Riedl [1,*]

[1] Institute of Chemistry and Biotechnology, Competence Center for Drug Discovery, Zurich University of Applied Sciences (ZHAW), Einsiedlerstrasse 31, 8820 Wädenswil, Switzerland.

* Correspondence: rainer.riedl@zhaw.ch

Table of Contents

1. SUPPLEMENTARY DATA 2

1.1. FIGURES 2
1.2. TABLES 3
1.3. SCHEMES 4

2. EXPERIMENTAL SECTION 5

2.1. CHEMISTRY 5
2.1.1. ORGANIC SYNTHESES OF SI COMPOUNDS 5

3. SUPPLEMENTARY DATA 10

3.1. NMR AND HPLC SPECTRA 10
3.2. BIOLOGICAL ASSAY RESULTS 108
3.2.1. SENP1-SUMO1-AMC 108
3.2.2. SENP1-SUMO2-AMC 124
3.2.3. SENP1-SUMO3-AMC 125
3.2.4. UCHL1 126
3.2.5. ATAXIN-3 127

1. Supplementary Data

1.1. Figures

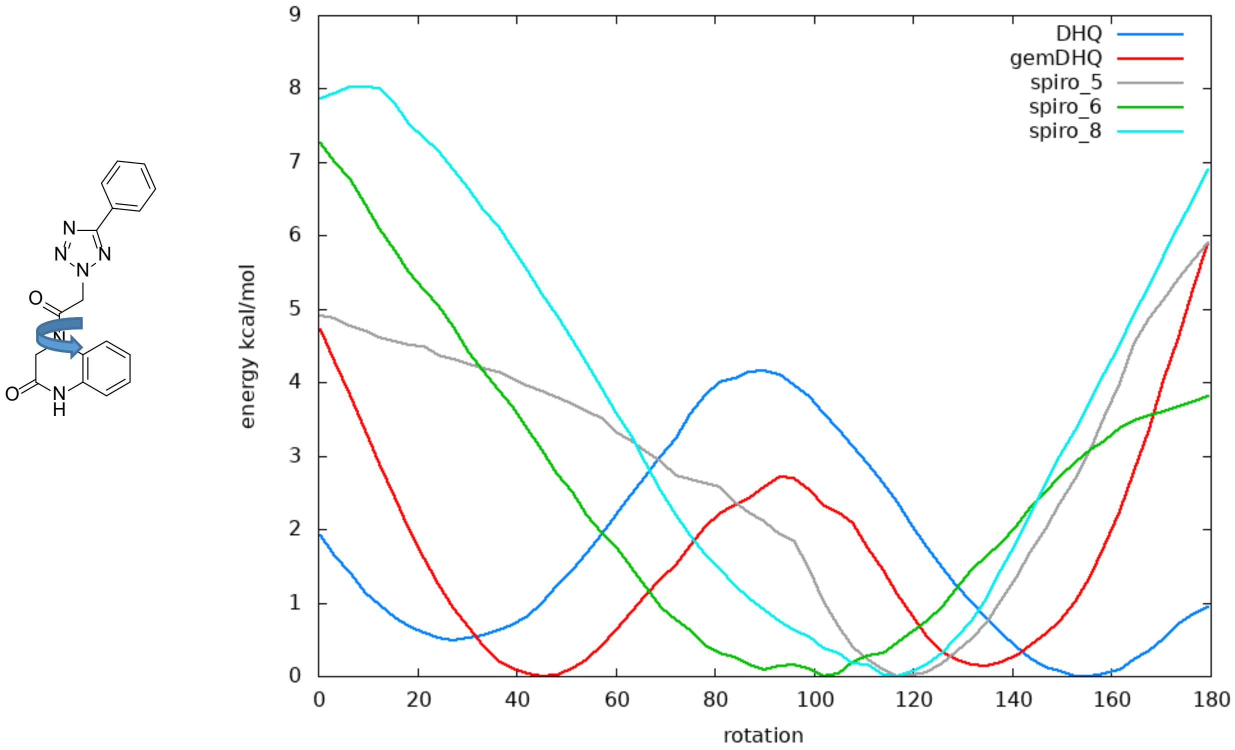

Figure S1 Calculation of the amide bond rotational barrier of **11** (spiro 5), **23** (gem-DHQ) and **24** (DHQ). Additionally the rotational barrier of the six-membered and eight membered ring DHQ is shown.

1.2. Tables

Compound	Structure	IC_{50} SENP1-SUMO1-AMC [μM] n = 2
**S1**	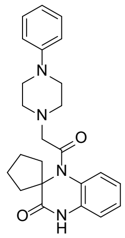	152 (74-312)[a]
**S2**	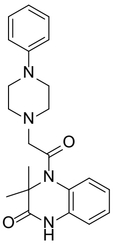	101 (70-146)
**S3**	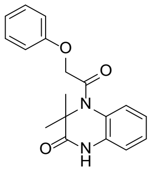	> 200
**S4**	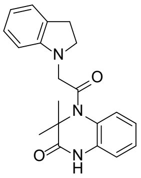	153 (132-177)
**S5**	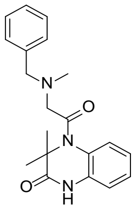	> 200
**S6**	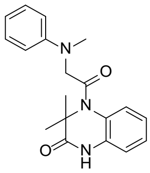	> 200
**S7**	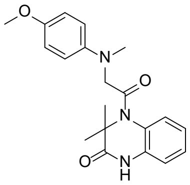	> 200

1.3. Schemes

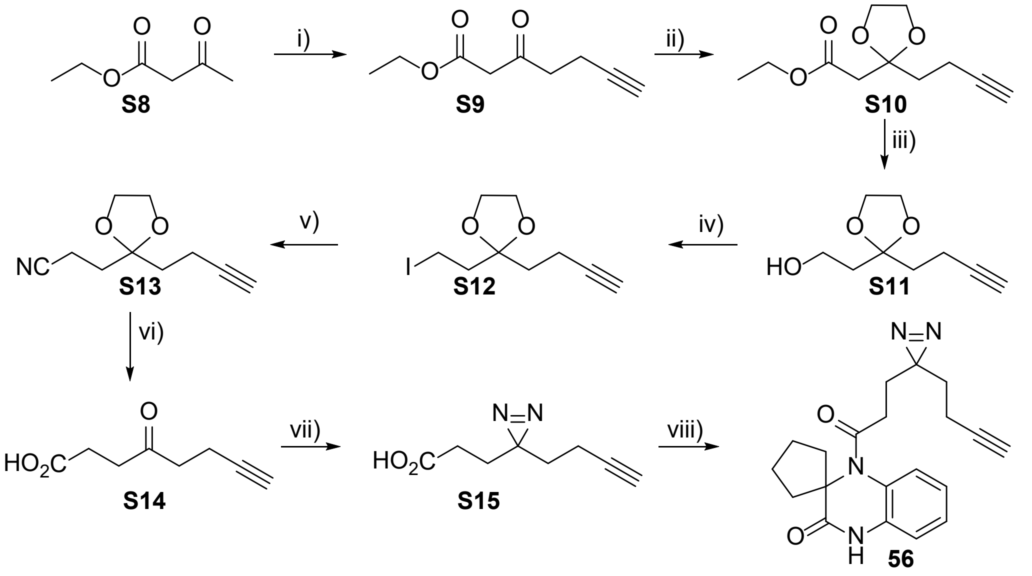

Scheme S1: Synthetic scheme of the photo affinity probe **56**. *Reagents & conditions*: i) 1: 2 M LDA in THF, THF, -78 °C – 0 °C, 15 minutes, 2: propargyl bromide, 1.5 h, 0 °C, 57%; ii) p-TsOH, ethylene glycol, Dean-Stark apparatus, 120 °C overnight, 85%; iii) $LiAlH_4$, THF, rt, 2 h, 90%; iv) PPh_3, I_2, imidazole, CH_2Cl_2, reflux, 1 h, v) KCN, DMF, 70 °C, 5 h, 45% over two steps; vi) 1: 5 M NaOH (aq.), reflux, 100 °C; 2: adjust pH to 1 with 5 M HCl (aq.), rt, 17 h, 89%; vii) 1: NH_3 (liq.), MeOH, -40 °C, 5 h, 2: NH_2-OSO_3H, MeOH, -40 °C – rt, overnight, 3: I_2, Et_3N, Et_2O, 0 °C – rt, 1 h, 28%; viii) 1: $SOCl_2$, CH_2Cl_2, 40 °C, 1 h, 2: **15**, CH_2Cl_2, Et_3N, rt, 1 h; 25%.

2. Experimental Section

2.1. Chemistry

2.1.1. Organic syntheses of SI compounds

1'-[2-(4-Phenylpiperazin-1-yl)acetyl]-3',4'-dihydro-1'*H*-spiro[cyclopentane-1,2'-quinoxalin]-3'-one (**S1**)

15 (55.0 mg, 0.197 mmol, 1.0 eq), 1-phenylpiperazine (37.3 µL, 38.4 mg, 0.237 mmol, 1.2 eq) and pyridine (31.9 uL, 31.2 mg, 0.395 mmol, 2.0 eq) was dissolved in DMF (3 mL) and stirred at 60 °C overnight. The reaction mixture was diluted with EtOAc (20 mL) and the organic layer was washed with brine (3 × 20 mL), 0.5 M aq. LiCl (3 × 10 mL), brine (10 mL), dried (Na_2SO_4), filtered and concentrated *in vacuo*. The residue was purifed by a SiO_2 column chromatography eluting with a gradient of 10 to 70% EtOAc in cyclohexan to afford a still impure product. A second purification by semi-prep reversed phase HPLC eluting with a gradient of 10 to 100% MeCN in H_2O with 0.2% AcOH afforded a colourless oil of **S1** (7.50 mg, 0.019 mmol, 9%). ^{1}H-NMR (500 MHz, $(CD_3)_2CO$: δ 9.38 (s, 1H), 7.67-7.63 (m, 1H), 7.29 (ddd, *J* 7.7, 7.7 1.4, 1H), 7.23-7.18 (m, 2H), 7.12-7.06 (m, 2H), 6.92-6.87 (m, 2H), 6.78 (tt, *J* 7.3, 1.1, 1H), 3.12 (s, 2H), 3.06-3.01 (m, 4H), 2.48-2.42 (m, 4H), 1.88-1.72 (m, 4H). ^{13}C-NMR (126 MHz, $(CDCl_3)$: δ 172.6, 172.3, 151.6, 134.9, 130.4, 128.8, 127.4, 126.1, 122.1, 119.0, 115.7, 115.34, 115.28 54.2, 69.4, 61.3, 54.2, 48.7 35.1, 23.8. LRMS (ESI) m/z $[M+H]^+$ 405. RP-HPLC: RT 9.68 min purity: 99.6%.

4-(2-Chloroacetyl)-3,3-dimethyl-1,2,3,4-tetrahydroquinoxalin-2-one (S**16**)

16 (500 mg, 2.84 mmol, 1.0 eq), in DMF (20 mL) was cooled to 0 °C. 2-chloroacetyl chloride (462 µL, 670 mg, 4.26 mmol, 1.5 eq) was added over 15 minutes. The reaction mixture was stirred at 0 °C for 120 minutes, at which point TLC indicated complete consumption of the starting material. To the reaction mixture was added EtOAc (100 mL) and the organic layer was washed with brine (3 × 100 mL), 0.5 M aq. LiCl (3 × 50 mL), brine (100 mL), dried (Na_2SO_4), filtered and concentrated *in vacuo*. The residue was purified by a SiO_2 column eluting with a gradient of 10 to 70% EtOAc in cyclohexan to afford a yellowish solid of **S16** (665 mg, 2.63 mmol, 93%). ^{1}H NMR (500 MHz, $(CDCl_3)$: δ 8.86 (s, 1H), 7.29-7.24 (m, 1H), 7.14-7.07 (m, 2H), 6.97 (dd, *J* 7.8, 1.4, 1H), 4.16 (s, 2H), 1.68 (s, 6H). ^{13}C-NMR (126 MHz, $(CDCl_3)$: δ 173.0, 168.6, 129.9, 127.0, 126.5, 123.8, 123.4, 116.0, 62.6, 43.9, 22.8. LRMS (ESI) m/z $[M+H]^+$ 253.

3,3-Dimethyl-4-[2-(4-phenylpiperazin-1-yl)acetyl]-1,2,3,4-tetrahydroquinoxalin-2-one (**S2**)

S16 (50 mg, 0.198 mmol, 1.0 eq), 1-phenylpiperazine (64.2 mg, 0.396 mmol, 2.0 eq) and K_2CO_3 (54.7 mg, 0.396 mmol, 2.0 eq) was suspended in DMF (2 mL) and stirred at 120 °C in the microwave for 1 hour. The reaction mixture was diluted with EtOAc (20 mL) and the organic layer was washed with brine (3 × 20 mL), 0.5 M aq. LiCl (3 × 10 mL), brine (10 mL), dried (Na_2SO_4), filtered and concentrated *in vacuo*. The residue was purified by semi-prep reversed phase HPLC eluting with a gradient of 10 to 100% MeCN in H_2O with 0.2% AcOH to afford a colourless oil (19.2 mg, 0.051 mmol, 20%). %). MP: 160 °C dec.; ^{1}H NMR (500 MHz, $(CD_3)_2CO$: δ 9.58 (s, 1H), 7.37 (dd, *J* 7.9, 0.9, 1H), 7.23-7.18 (m, 3H), 7.11-7.04 (m, 2H), 6.92-6.86 (m, 2H), 6.78 (tt, *J* 7.4, 1.2, 1H), 3.31 (s, 2H), 3.02-2.99 (m, 4H), 2.57-2.52 (m, 1.56 (s, 6H). ^{13}C-NMR (126 MHz, $(CDCl_3)$: δ 172.7, 171.7, 151.6, 131.3, 128.8, 128.1, 125.8, 123.8, 123.3, 122.3, 119.0, 115.6, 115.2, 62.2, 61.2, 52.6, 48.7, 22.7. LRMS (ESI) m/z $[M+H]^+$ 379. RP-HPLC: RT 8.79 min purity: 99.4%.

3,3-Dimethyl-4-(2-phenoxyacetyl)-1,2,3,4-tetrahydroquinoxalin-2-one (**S3**)

S16 (50 mg, 0.198 mmol, 1.0 eq), phenol (37.2 mg, 0.396 mmol, 2.0 eq), K_2CO_3 (54.7 mg, 0.396 mmol, 2.0 eq) and DMF (2 mL) was stirred at 120 °C for 1 hour under microwave irradiation, at which time the reaction showed complete consumption of the starting material by HPLC. The reaction mixture was diluted with EtOAc (20 mL) and the organic layer was washed with brine (3 × 20 mL), 0.5 M aq. LiCl (3 × 10 mL), brine (10 mL), dried (Na_2SO_4), filtered and concentrated *in vacuo*. The residue was purified by a SiO_2 column chromatography eluting with a gradient of 10 to 50% EtOAc in cyclohexan to afford a colourless solid of **S3** (47.3 mg, 0.152 mmol, 77%). Mp 140 °C dec.; ^{1}H NMR (500 MHz, $(CD_3)_2CO$: δ 9.53 (s, 1H), 7.35 (dd, *J* 8.0, 1.4 Hz, 1H), 7.27-7.20 (m, 3H), 7.12 (dd, *J* 8.0, 1.4 Hz, 1H), 7.05 (ddd, *J* 8.0, 8.0 1.4 Hz, 1H), 6.92 (tt, *J* 7.2, 1.1 Hz, 1H), 6.79-6.74 (m, 2H), 6.78 (tt, *J* 7.3, 1.1 Hz, 1H), 7.00 (dd, *J* 7.8, 1.3 Hz, 1H), 4.80 (s, 2H), 1.45 (s, 6H). ^{13}C-NMR (126 MHz, ($CDCl_3$): δ 171.3, 170.0, 158.1, 131.2, 129.3, 126.4, 123.5, 122.7, 121.1, 115.5, 114.5, 68.1, 61.4, 22.4. LRMS (ESI) m/z $[M+H]^+$ 311. RP-HPLC: RT: 12.1 min purity: 97.8%.

4-[2-(2,3-Dihydro-1*H*-indol-1-yl)acetyl]-3,3-dimethyl-1,2,3,4-tetrahydroquinoxalin-2-one (**S4**)

S16 (50 mg, 0.198 mmol, 1.0 eq), indoline (47.17 mg, 0.396 mmol, 1.2 eq), K_2CO_3 (54.7 mg, 0.396 mmol, 2.0 eq) and DMF (3 mL) and stirred at 60 °C overnight, at which point the reaction showed complete consumption of the starting material by UPLCMS. The reaction mixture was diluted with EtOAc (20 mL) and the organic layer was washed with brine (3 × 20 mL), 0.5 M aq. LiCl (3 × 10 mL), brine (10 mL), dried (Na_2SO_4), filtered and concentrated *in vacuo*. The residue was purified by a SiO_2 column chromatography eluting with a gradient of 10 to 60% EtOAc in cyclohexan to afford a colourless oil of **S4** (13.7 mg, 0.041 mmol, 21%). ^{1}H NMR (500 MHz, $(CD_3)_2CO$: δ 9.56 (s, 1H), 7.35 (dd, *J* 8.1, 1.2 Hz, 1H), 7.25 (ddd, *J* 7.9, 7.9, 1.2 Hz, 1H), 7.14-7.08 (m, 2H), 7.00-6.96 (m, 1H), 6.92-6.87 (m, 1H), 6.54 (ddd, *J* 7.6, 7.6, 0.9 Hz, 1H), 6.25 (d, *J* 7.6 Hz, 1H), 4.05 (s, 2H), 3.42 (t, *J* 8.7, 2H), 2.87 (t, *J* 8.7, 2H), 1.53 (s, 6H). ^{13}C-NMR (126 MHz, ($CDCl_3$): δ 171.73, 171.66, 151.5, 131.4, 129.3, 127.0, 126.8, 126.2, 124.1, 122.7, 117.3, 115.5, 115.4, 106.2, 61.4, 53.2, 53.1, 26.6, 22.6. LRMS (ESI) m/z $[M+H]^+$ 336. RP-HPLC: RT: 12.8 min purity: 95.4%

4-{2-[Benzyl(methyl)amino]acetyl}-3,3-dimethyl-1,2,3,4-tetrahydroquinoxalin-2-one (**S5**)

S16 (50 mg, 0.198 mmol, 1.0 eq), *N*-methylbenzylamin (48.0 mg, 0.396 mmol, 2.0 eq) and K_2CO_3 was suspended in DMF (3 mL) and heated under microwave irradiation for 1 hour at 120 °C. The reaction mixture was taken up in EtOAc (20 mL) and the organic layer was washed with sat. aq. K_2CO_3 (3 × 20 mL), brine (10 mL), dried (Na_2SO_4), filtered and concentrated *in vacuo*. The residue was purified by reversed phase column chromatography eluting with a gradient of 10 to 100% MeCN in H_2O +0.2% AcOH to afford a colourless oil of **S5** (14.5 mg, 0.043 mmol, 22%). ^{1}H NMR (500 MHz, $(CD_3)_2CO$: δ 9.58 (s, 1H), 7.29-7.15 (m, 7H), 7.10 (dd, *J* 8.0, 1.4 Hz, 1H) 6.99 (ddd, *J* 8.0, 8.0, 1.4 Hz, 1H), 3.57 (s, 2H), 3.40 (s, 2H), 2.21 (s, 3H), 1.57 (s, 6H). ^{13}C-NMR (126 MHz, ($CDCl_3$): δ 173.1, 171.8, 139.0, 131.0, 128.7, 128.0, 127.5, 126.8, 125.7, 123.7, 122.5, 115.32, 115.25, 61.2, 60.93, 60.92, 41.2, 22.6. LRMS (ESI) m/z $[M+H]^+$ 338. RP-HPLC: RT 7.16 min purity: 95.0%.

3,3-Dimethyl-4-{2-[methyl(phenyl)amino]acetyl}-1,2,3,4-tetrahydroquinoxalin-2-one (**S6**)

S16 (50 mg, 0.198 mmol, 1.0 eq), *N*-Methylanilin (42.2 mg, 0.396 mmol, 2.0 eq) and K_2CO_3 (54.7 mg, 0.396 mmol, 2.0 eq) was suspended in DMF (2 mL) and stirred at 120 °C under microwave irradiation for 5 hours. To the reaction mixture was added EtOAc (20 mL) and the organic layer was washed with brine (3 × 20 mL), 0.5 M aq. LiCl (3 × 10 mL), brine (10 mL), dried (Na_2SO_4), filtered and concentrated *in vacuo*. The residue was purified by reversed phase column chromatography eluting with a gradient of 10 to 100% MeCN in H_2O +0.2% AcOH afforded a colourless solid of **S6** (12.4 mg, 0.033 mmol, 17%). Mp 158 °C dec.; ^{1}H NMR (500 MHz, $(CD_3)_2CO$: δ 9.54 (s, 1H), 7.39 (dd, *J* 7.8, 1.3 Hz, 1H), 7.28 (ddd, *J* 7.8, 7.8, 1.3 Hz, 1H), 7.17-7.07 (m, 4H), 6.61 (tt, *J* 7.3, 0.9 Hz, 1H), 6.58-6.54 (m, 2H), 4.26 (s, 2H), 2.92 (s, 3H), 1.48 (s, 6H). ^{13}C-NMR (126 MHz, ($CDCl_3$): δ 172.3, 171.8, 149.0, 131.8, 128.7, 126.6, 124.5, 122.7, 116.4, 115.6, 112.0, 61.3, 56.8, 39.0, 22.7.. LRMS (ESI) m/z $[M+H]^+$ 324. RP-HPLC: RT 12.43 min purity: 98.3%.

4-{2-[(4-Methoxyphenyl)(methyl)amino]acetyl}-3,3-dimethyl-1,2,3,4-tetrahydroquinoxalin-2-one (**S7**)

S16 (50 mg, 0.198 mmol, 1.0 eq), 4-Methoxy-*N*-methylanilin (54.3 mg, 0.396 mmol, 2.0 eq) and K_2CO_3 (54.7 mg, 0.396 mmol, 2.0 eq) was suspended in DMF (3 mL) and heated under microwave irradiation for 2 hours at 120 °C. The reaction mixture was taken up in EtOAc (20 mL) and the organic layer was washed with brine (3 × 20 mL), 0.5 M aq. LiCl (3 × 10 mL), brine (10 mL), dried (Na_2SO_4), filtered and concentrated *in vacuo*. The residue was purified on reversed phase C_{18} column eluting with a gradient of 10 to 100% MeCN in H_2O +0.2% AcOH to afford a colourless solid (8.4 mg, 0.024 mmol, 12%). Mp 131 °C dec.; ^{1}H NMR (500 MHz, $(CD_3)_2CO$): δ 9.52 (s, 1H), 7.33 (dd, *J* 7.9, 1.2, 1H), 7.26 (ddd, *J* 7.9, 7.9, 1.2 Hz, 1H), 7.14 (dd, *J* 7.9, 1.2 Hz, 1H), 6.74-6.69 (m, 2H), 6.55-6.50 (m, 2H), 4.19 (s, 2H), 3.68 (s, 3H), 2.87 (s, 2H), 1.46 (s, 6H). ^{13}C-NMR (126 MHz, ($CDCl_3$): δ 172.5, 171.7, 151.8, 143.5, 131.6, 126.8, 126.4, 124.3, 122.6, 115.5, 114.3, 113.7, 61.2, 57.5, 54.8, 39.4, 22.6. LRMS (ESI) m/z $[M+H]^+$ 354. RP-HPLC: RT 12.18 min purity: 97.6%

Ethyl 3-oxohept-6-ynoate (**S9**)

Ethyl aceto acetate (**S8**) (6.00 g, 46.1 mmol, 1.0 eq) was dissolved in THF (100 mL) and cooled to -78 °C. 2 M LDA in THF (46.1 mL, 92.2 mmol, 2.0 eq) was added over 15 minutes and the solution was subsequently allowed to warm up to 0 °C and stirred for 15 minutes . The solution was cooled again to -78 °C. 80% propargyl bromide in toluene (6.86 g, 46.1 mmol, 1.0 eq) was added over 15 minutes and the reaction mixture was allowed to warm up to 0 °C and stirred for 1.5 hours at this temperature. Glacial acetic acid (5.9 mL) was carefully added to quench the reaction mixture. The suspension was concentrated *in vacuo*, and the residue was dissolved in EtOAc (150 mL) and H_2O (100 mL). The organic extracts were washed with brine (2 × 100 mL), dried (Na_2SO_4), filtered, and concentrated *in vacuo* to afford a 3 to 1 mixture of product and starting material. The residual oil was purified by a Kugelrohr vacuum distillation to afford a pale yellow oil of **S9** (4.42 g, 26.3 mmol, 57%). ^{1}H NMR (500 MHz, $CDCl_3$): δ 4.22 (q, *J* 7.2 Hz, 2H), 3.49 (s, 2H), 2.83 (t, *J* 7.2 Hz, 2H), 2.50 (td, *J* 7.2, 2.5 Hz, 2H), 1.98 (t, *J* 2.5 Hz, 1H), 1.30 (t, *J* 7.2 Hz, 3H). ^{13}C-NMR (126 MHz, $CDCl_3$): δ 200.6, 166.9, 82.5, 69.0, 61.5, 49.2, 41.6, 14.1, 12.8.

Ethyl 2-[2-(but-3-yn-1-yl)-1,3-dioxolan-2-yl]acetate (**S10**)

Ethyl 3-oxohept-6-ynoate (**S9**) (4.30 g, 25.6 mmol, 1.0 eq) was dissolved in toluene (150 mL). To this solution ethylene glycol (1.75 g, 28.1 mmol, 1.1 eq) and 4-toluenesulfonic acid monohydrate (0.24 g, 1.3 mmol, 0.05 eq) was added and stirred at reflux in a Dean-Stark apparatus overnight. After cooling to ambient temperature the organic layer were washed with 1 M aq. Na_2CO_3 (3 × 100 mL), brine (100 mL), dried (Na_2SO_4), filtered and concentrated *in vacuo* to afford a pale yellow oil of **S10** (4.62 g, 21.8 mmol, 85%), which was used without further purification. ^{1}H NMR (500 MHz, $CDCl_3$): δ 4.18 (q, *J* 7.3 Hz, 2H), 4.05-3.97 (m, 4H), 2.67 (s, 2H), 2.36-2.30 (m, 2H), 2.19-2.11 (m, 2H), 1.95 (t, *J* 2.5, 1H), 1.29 (t, *J* 7.3 Hz, 3H).

2-[2-(But-3-yn-1-yl)-1,3-dioxolan-2-yl]ethan-1-ol (**S11**)

S10 (6.80 g, 32.0 mmol, 1.0 eq) was dissolved in THF and cooled to 0 °C. 2 M $LiAlH_4$ in THF (17.0 mL, 34.0 mmol, 1.05 eq) was carefully added over 15 minutes. The yellow solution was warmed up to ambient temperature and stirred for 2 hours, at which point the reduction was completed. The yellow solution was cooled to 0 °C. EtOAc (5 mL) was slowly added followed by careful addition of H_2O (10 mL). The THF was evaporated *in vacuo*. To the residue EtOAc (150 mL) and sat. rochelle solution (100 mL) was added and dissolved. The organic layer was washed with brine (2 × 100 mL), dried (Na_2SO_4), filtered and concentrated *in vacuo* to afford a yellow oil of **S11** (4.90 g, 28.8 mmol, 90%), which was used without further purification. ^{1}H-NMR (500 MHz, CDCl3): δ 4.06-3.98 (m, 4H), 3.78 (q, *J* = 7.7 Hz, 2H), 2.70-2.63 (m, 1H), 2.33-2.27 (m, 2H), 2.01-1.92 (m, 5H). ^{13}C-NMR (126 MHz, $CDCl_3$): δ 111.1, 83.9, 68.3, 65.0, 58.7, 38.2, 35.9, 13.2.

3-[2-(But-3-yn-1-yl)-1,3-dioxolan-2-yl]propanenitrile (**S13**)

PPh_3 (8.31 g, 31.7 mmol, 1.1 eq) and 1*H*-imidazole (5.88 g, 86.3 mmol, 3.0 eq) were dissolved in CH_2Cl_2 (300 mL). Iodine (8.77 g, 34.5 mmol, 1.2 eq) was added portion wise and stirred for 15 min at ambient temperature. MIB-4-274 (4.90 g, 28.8 mmol, 1.0 eq) in CH_2Cl_2 (50 mL) was added over 15 minutes. The reaction mixture was heated at reflux for 1 hour, at which point the reaction was completed. The brown solution was cooled to ambient temperature and quenched with 10% w/v aq. $Na_2S_2O_3$ (150 mL).The organic layer was washed with brine (2 × 150 mL), dried (Na_2SO_4), filtered and concentrated *in vacuo*. The residual solid was stirred in cyclohexan (170 mL) and TBME (20 mL) for 5 minutes, filtered and concentrated *in vacuo* to afford a yellow oil (**S12**, 6.90 g), which contains around 30% PPh_3, which was used without further purification. This crude mixture was taken up in DMF (25 mL) and KCN (0.85 g, 13.0 mmol, 1.1 eq) was added. This reaction mixture was stirred for 5 hours at 70 °C. Upon cooling to ambient temperature the suspension was dissolved with 10% aq. $NaHCO_3$ (50 mL) and EtOAc (100 mL). The organic layer was washed with with 0.5 M aq. LiCl (2 × 10 mL), brine (10 mL), dried (Na_2SO_4), filtered, and concentrated *in vacuo*. The residue was purified by a SiO_2 column eluting with a gradient of 0% to 20% EtOAc in cyclohexane to afford a colourless oil of **S13** (2.20 g, 12.9 mmol, 45% over two steps). ^{1}H NMR (500 MHz, $CDCl_3$): δ 4.01 (s, 4H),2.43 (t, *J* 7.7 Hz, 2H), 2.32-2.27 (m, 2H), 2.07 (t, *J* 7.5 Hz, 2H), 1.98 (t, *J* 2.7 Hz, 1H), 1.90 (t, *J* 7.7 Hz, 2H); ^{13}C-NMR (126 MHz, $CDCl_3$): δ 119.7, 108.9, 83.6, 68.6, 65.3, 35.9, 32.5, 13.2, 11.6.

4-Oxooct-7-ynoic acid (**S14**)

S13 (2.15 g, 12.0 mmol, 1.0 eq) and 10% aq. NaOH (10 mL) was stirred at reflux for 5 hours. Upon cooling the pH of the aq. layer was adjusted with 5 M aq. HCl to 1 and stirred at ambient temperature overnight. The organic extracts of aq. layer were extracted with EtOAc (5 × 50 mL). The combined organic layers were dried (Na_2SO_4), filtered and concentrated *in vacuo*. The residue was purified by a SiO_2 column eluting with a gradient of 50% to 100% of EtOAc in cyclohexane containing 2% of AcOH to afford a pale yellow solid (1.64 g, 10.6 mmol, 89%). ^{1}H NMR (500 MHz, $CDCl_3$): δ 2.80-2.71 (m, 4H), 2.71-2.66 (m, 2H), 2.52-2.46 (m, 2H), 1.97 (t, *J* 7.5 Hz, 1H). ^{13}C-NMR (126 MHz, $CDCl_3$): δ 206.4, 178.3, 82.9, 68.8, 41.3, 36.8, 27.7, 12.9.

3-[3-(But-3-yn-1-yl)-3*H*-diazirin-3-yl]propanoic acid (**S15**)

S14 (1000 mg, 6.49 mmol, 1.0 eq) was dissolved in dry MeOH (5 mL) and liquid ammonia (20 mL) was condensed into the apparatus. This solution was stirred at -40 °C for 5 h. A solution of hydroxylamine O-sulfonic acid (676 mg, 10.4 mmol, 1.6 eq) in dry MeOH (8 mL) was added dropwise. After one hour at −40 °C the suspension was allowed to warm up to ambient temperature overnight. The suspension was diluted with MeOH (5 mL), filtered, rinsed with MeOH (10 mL), and concentrated *in vacuo*. The residual oil was dissolved in Et_2O (10 mL) and triethylamine (2 mL), and cooled to 0 °C. I_2 (2.5 g) was added in 5 portions until the brown colour persist. After 1 hour at ambient temperature the solution was diluted with EtOAc (50 mL). The pH was adjusted to 2 with 1 M aq. HCl (10 mL) and the organic layer was washed with 10% aq. $Na_2S_2O_3$ (50 mL), 1 M aq. HCl (50 mL), dried (Na_2SO_4), filtered and concentrated *in vacuo*. The residue was purified by a SiO_2 column eluting with a gradient of 10% to 50% petroleum ether in EtOAc containing 1% AcOH, to afford a yellow oil (304 mg, 1.83 mmol, 28%). ^{1}H NMR (500 MHz, $CDCl_3$): δ 2.18 (t, *J* 7.6 Hz, 2H), 2.05-1.98 (m, 3H), 1.82 (t, *J* 7.5 Hz, 2H), 1.67 (t, *J* 7.5 Hz, 2H). ^{13}C-NMR (126 MHz, $CDCl_3$): δ 178.0, 82.5, 69.3, 32.2, 28.1, 27.8, 27.5, 13.2.

3. Supplementary Data

3.1. NMR and HPLC spectra

Compound 15

^{1}H-NMR

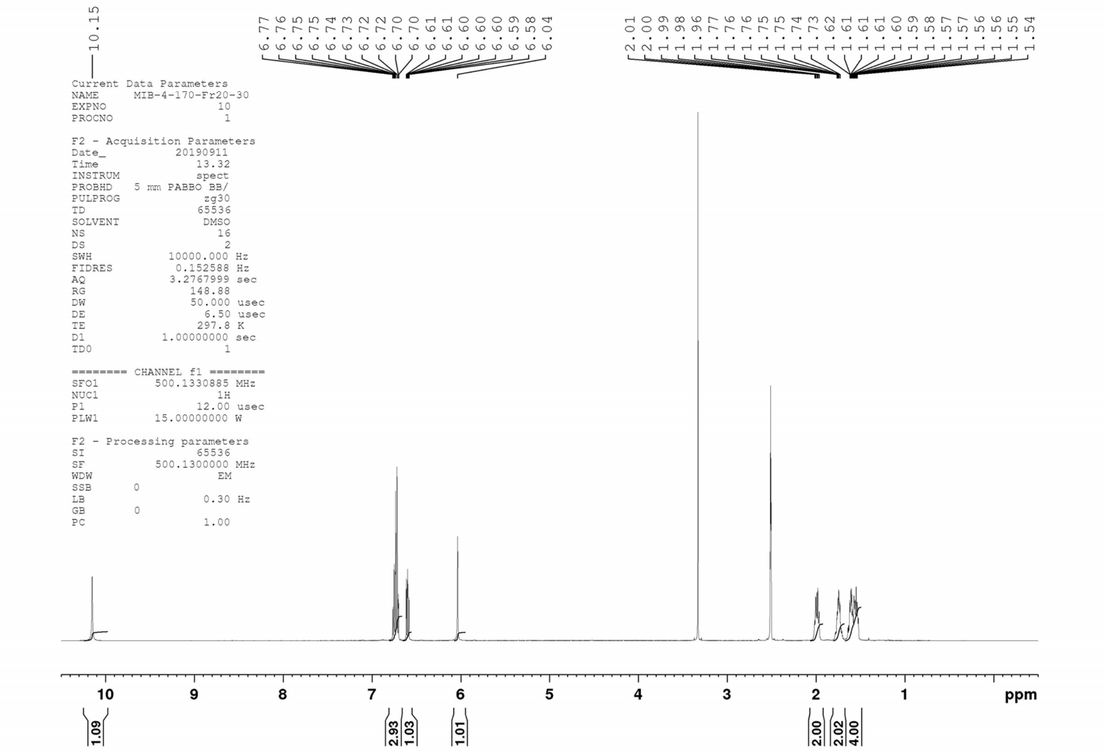

^{13}C-NMR

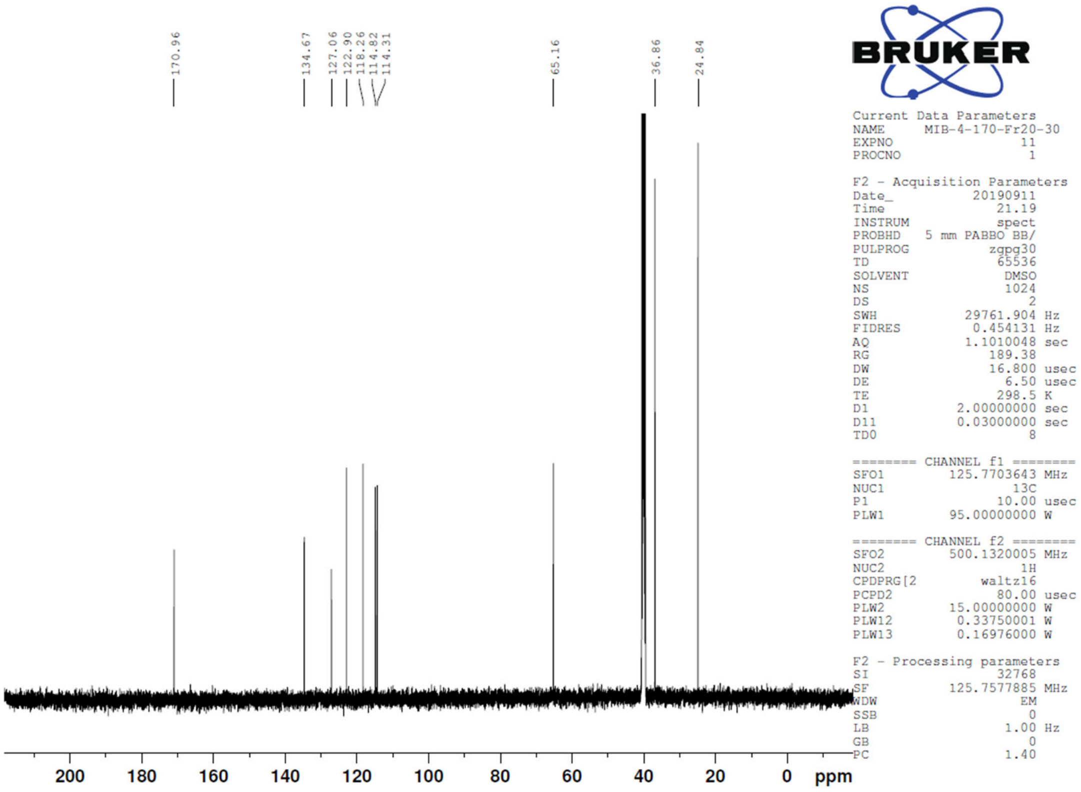

LC

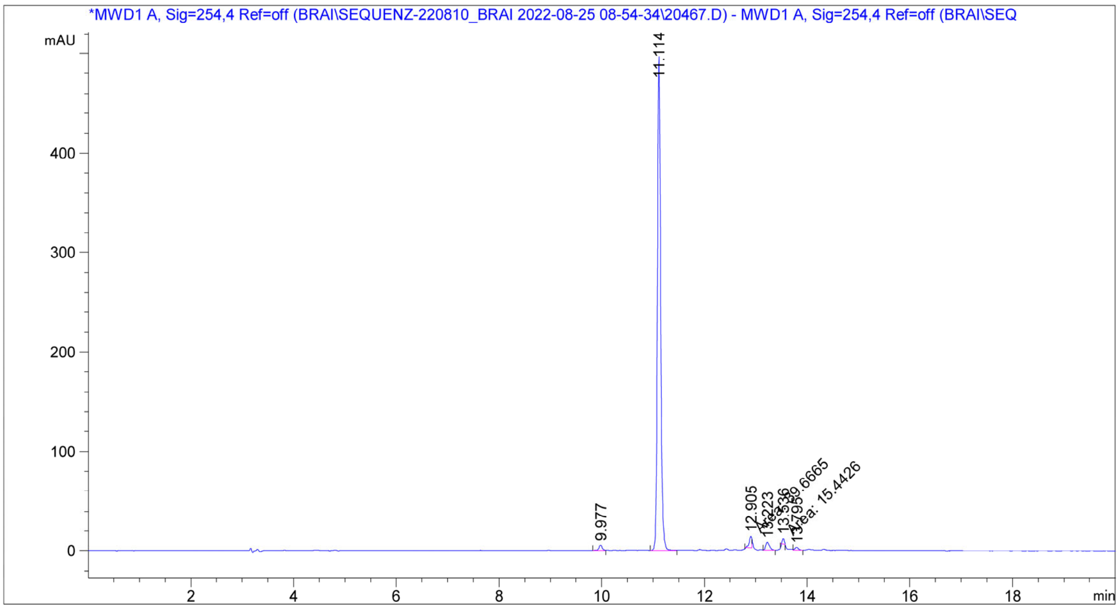


```
=====================================================================
                          Area Percent Report
=====================================================================

Sorted By             :      Signal
Multiplier            :      1.0000
Dilution              :      1.0000
Use Multiplier & Dilution Factor with ISTDs


Signal 1: MWD1 A, Sig=254,4 Ref=off
 Signal has been modified after loading from rawdata file!

Peak RetTime Type  Width     Area      Height     Area
  #   [min]        [min]   [mAU*s]     [mAU]        %
----|-------|----|-------|----------|----------|--------|
   1   9.977 VV    0.0722   26.12964    5.42852   1.1171
   2  11.114 BV    0.0676 2202.04565  497.41626  94.1402
   3  12.905 MM    0.0550   39.66650   12.02836   1.6958
   4  13.223 VB    0.0783   41.06660    7.94498   1.7556
   5  13.536 MM    0.0433   15.44265    5.94494   0.6602
   6  13.795 VB    0.0740   14.76199    3.07795   0.6311
```

Compound 16

^{1}H-NMR

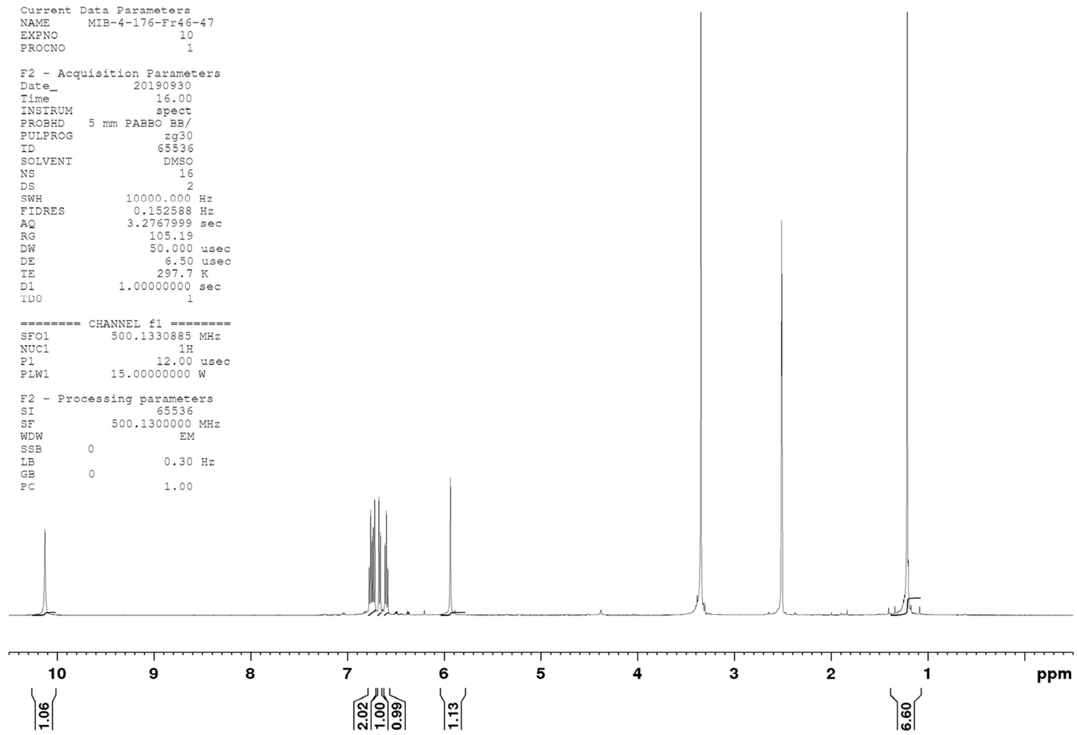

^{13}C-NMR

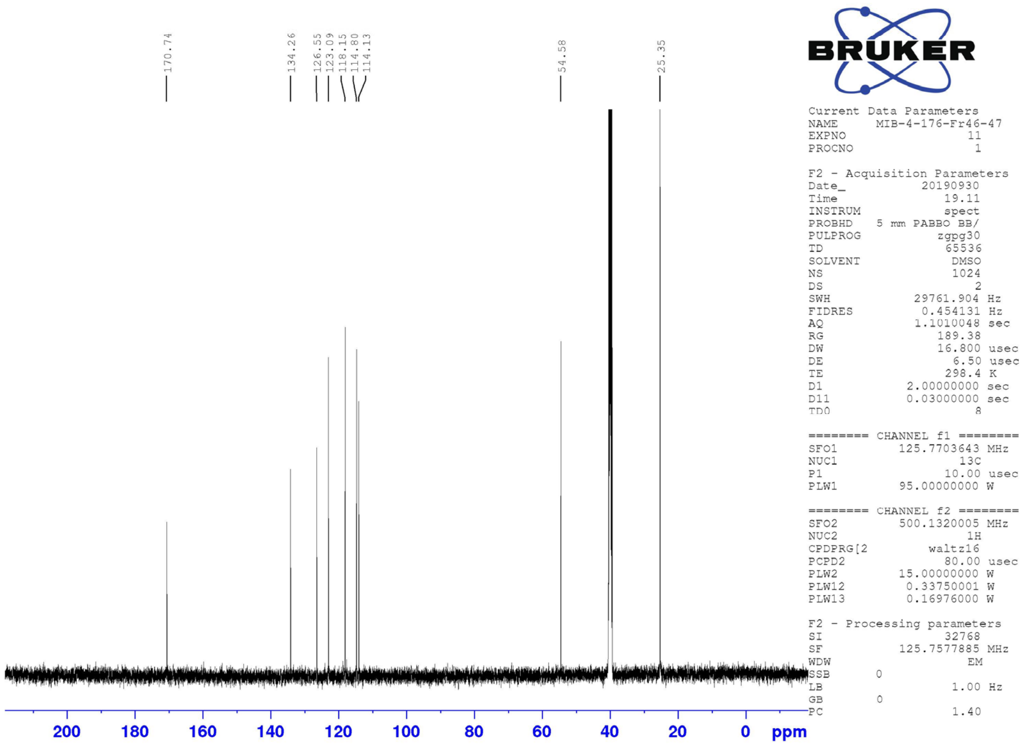

LC

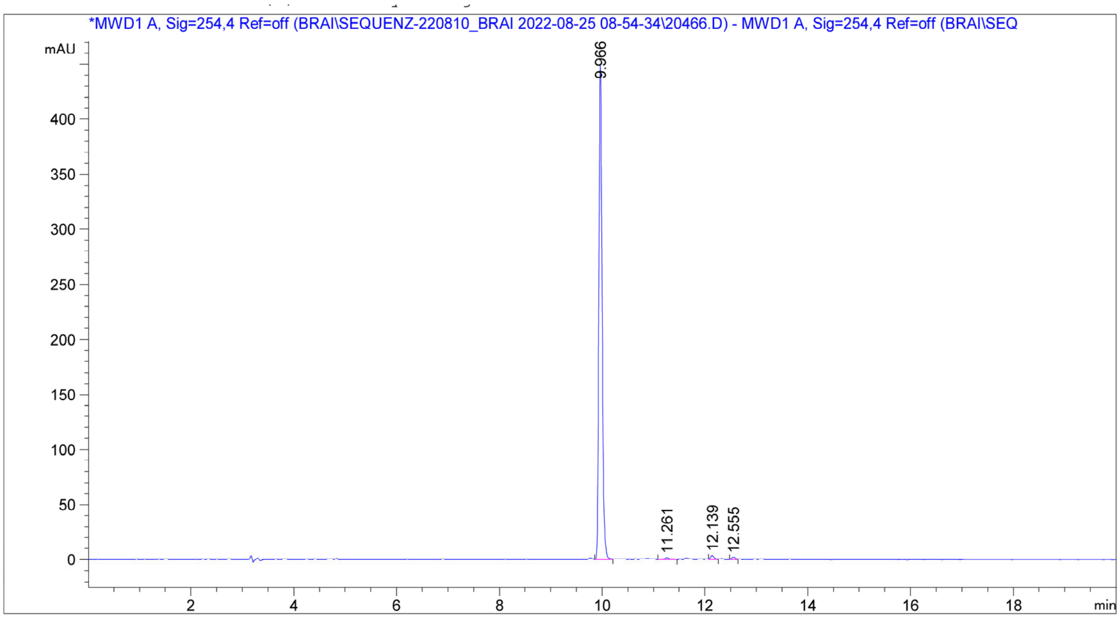

===

Area Percent Report

===

Sorted By : Signal
Multiplier : 1.0000
Dilution : 1.0000
Use Multiplier & Dilution Factor with ISTDs

Signal 1: MWD1 A, Sig=254,4 Ref=off
Signal has been modified after loading from rawdata file!

Peak #	RetTime [min]	Type	Width [min]	Area [mAU*s]	Height [mAU]	Area %
1	9.966	BV	0.0686	1945.47693	448.92221	98.1594
2	11.261	BV	0.0803	9.72409	1.71239	0.4906
3	12.139	VV	0.0711	16.78909	3.55318	0.8471
4	12.555	VV	0.0680	9.96659	2.23754	0.5029

Totals : 1981.95670 456.42532

Compound 18

^{1}H-NMR

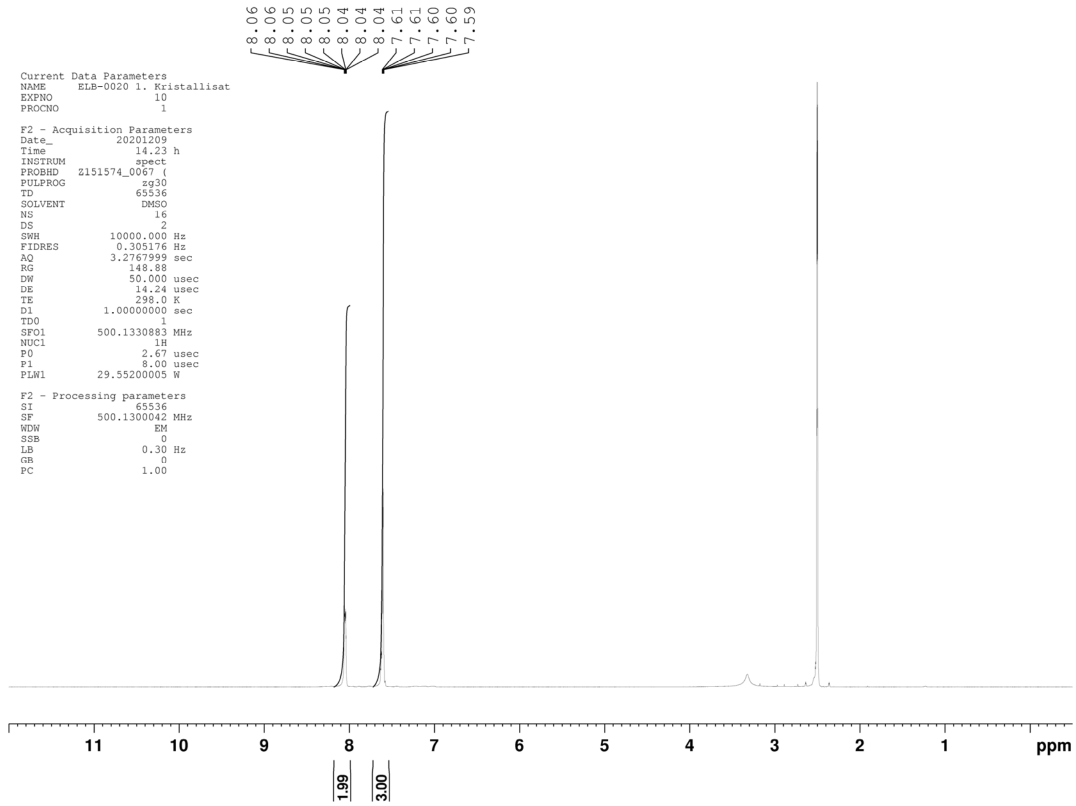

^{13}C-NMR

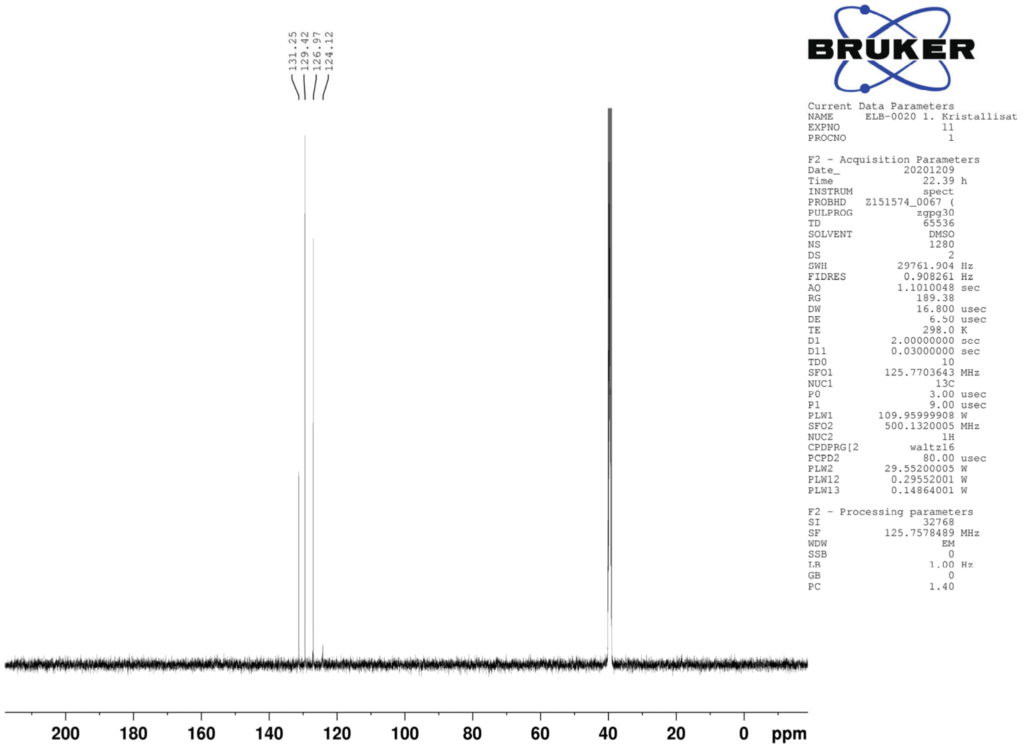

Compound 19

^{1}H-NMR

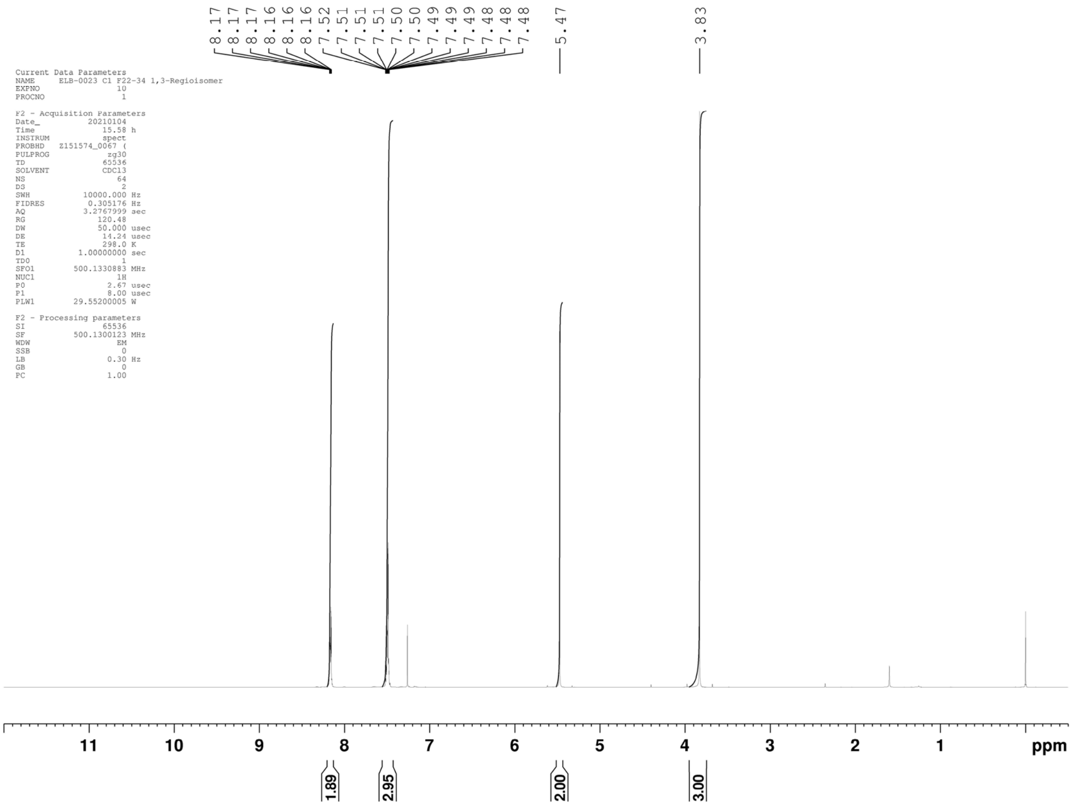

^{13}C-NMR

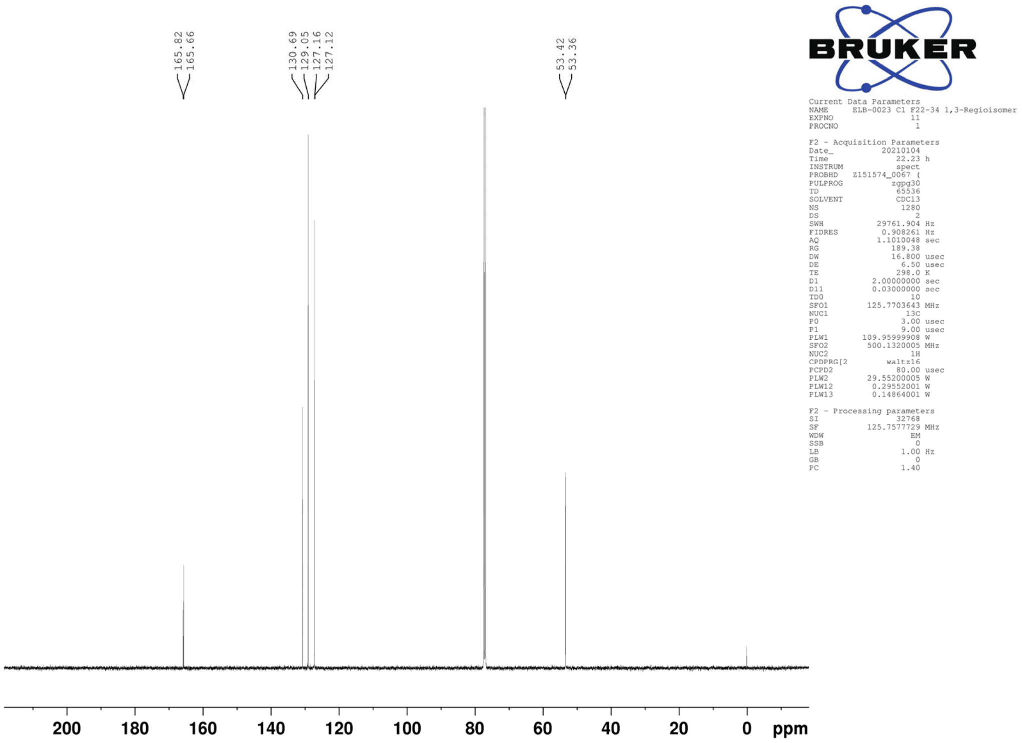

Compound 20

^{1}H-NMR

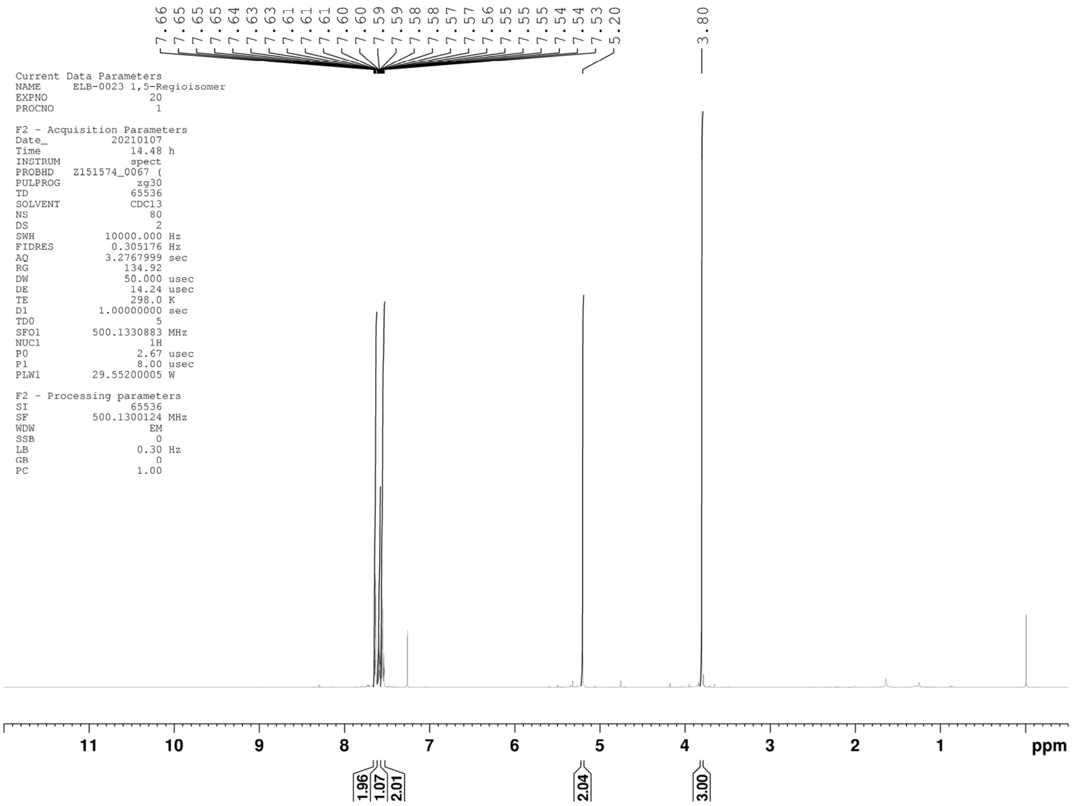

^{13}C-NMR

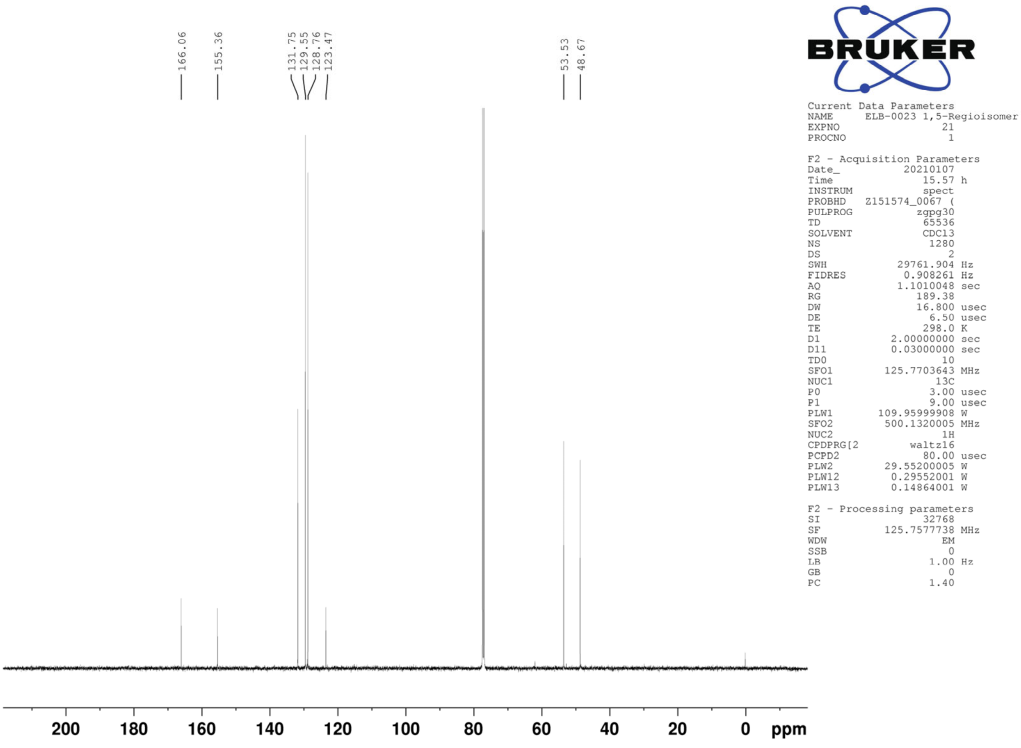

Compound 21

^{1}H-NMR

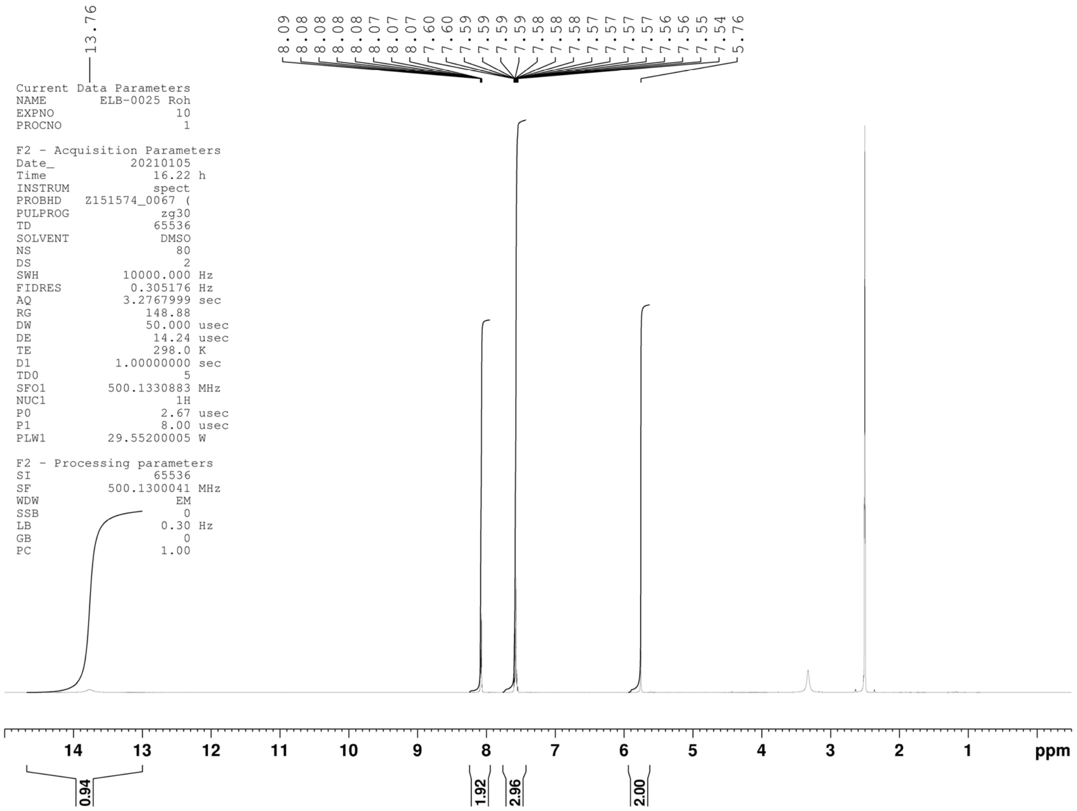

^{13}C-NMR

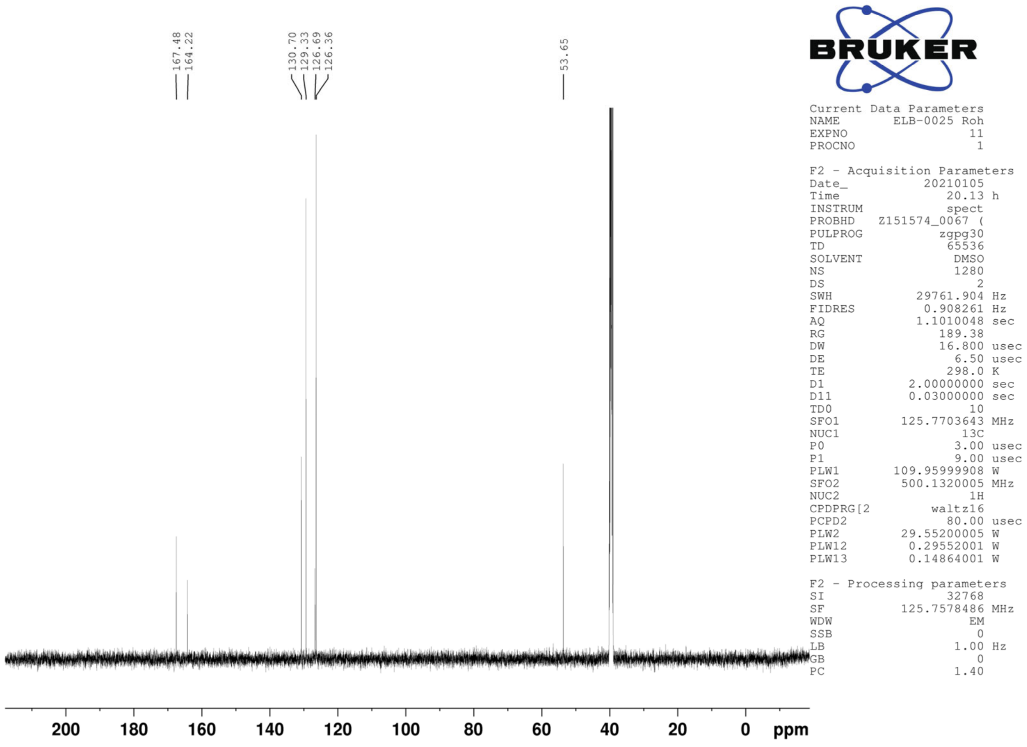

Compound 22

^{1}H-NMR

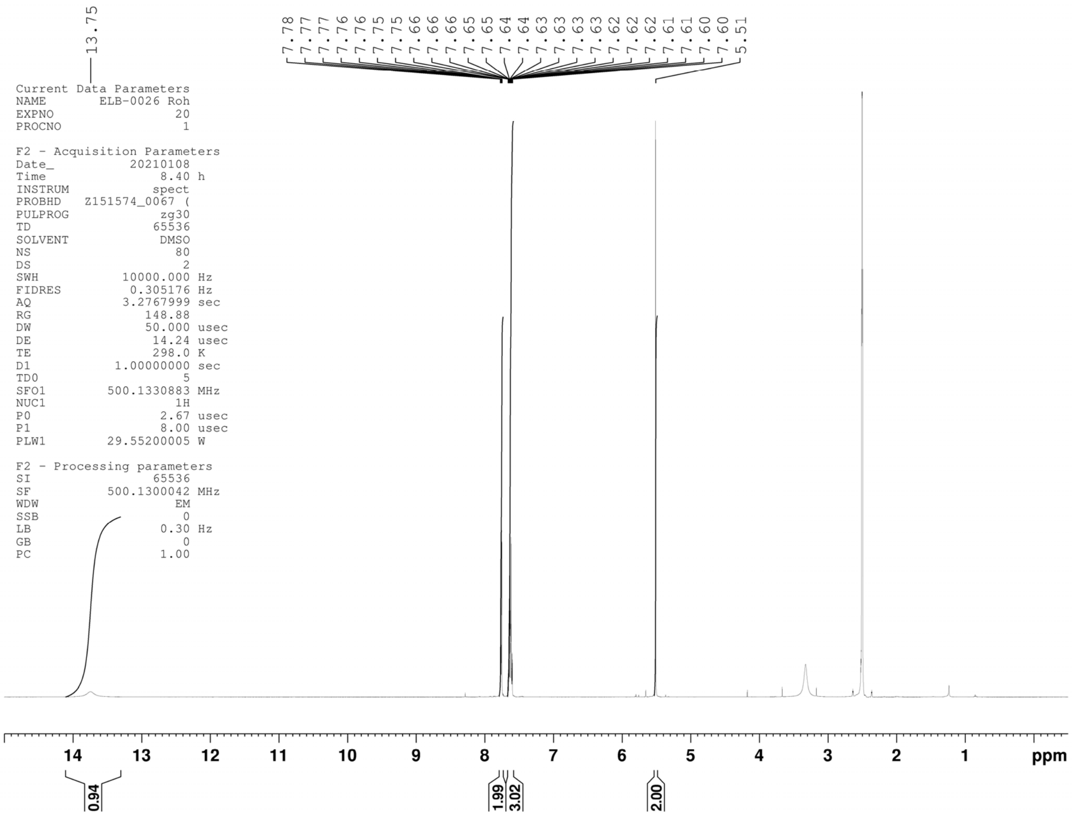

^{13}C-NMR

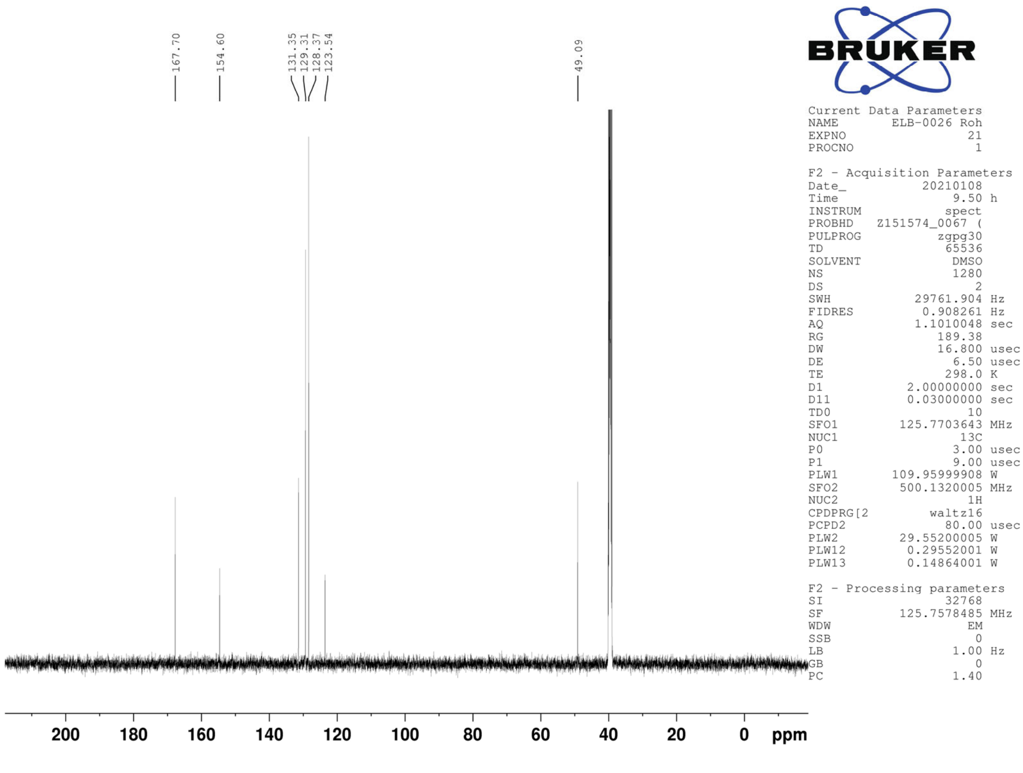

Compound 11

^{1}H-NMR

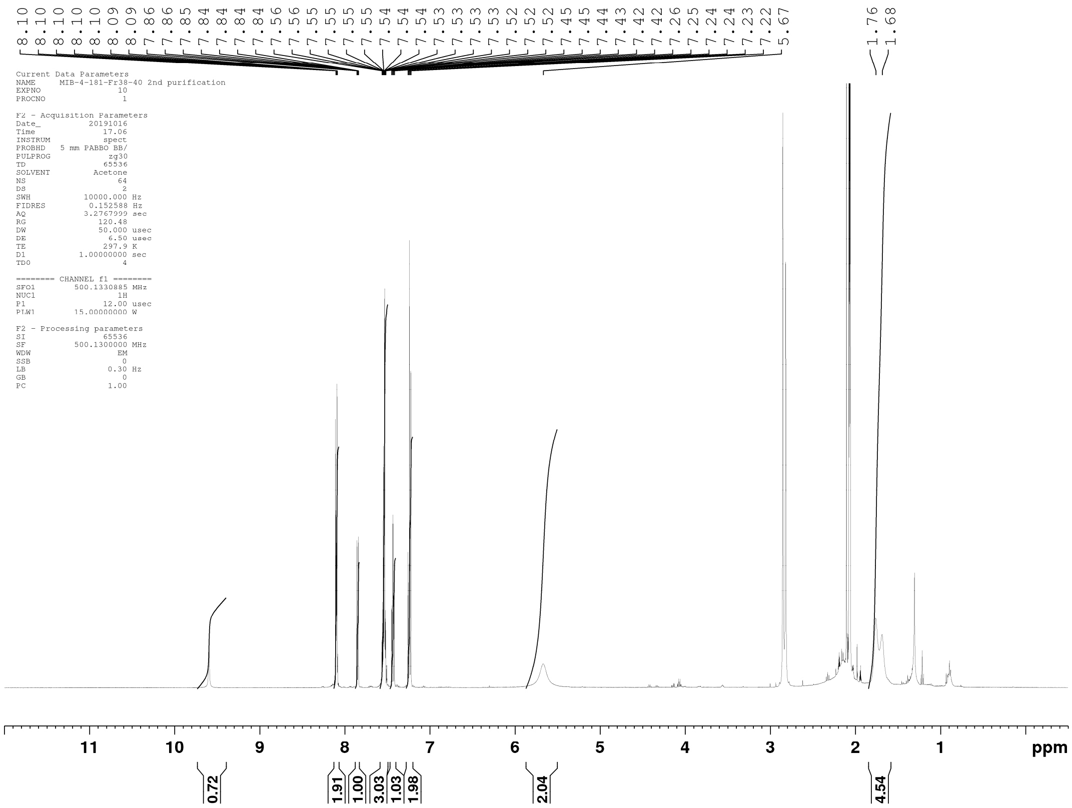

^{13}C-NMR

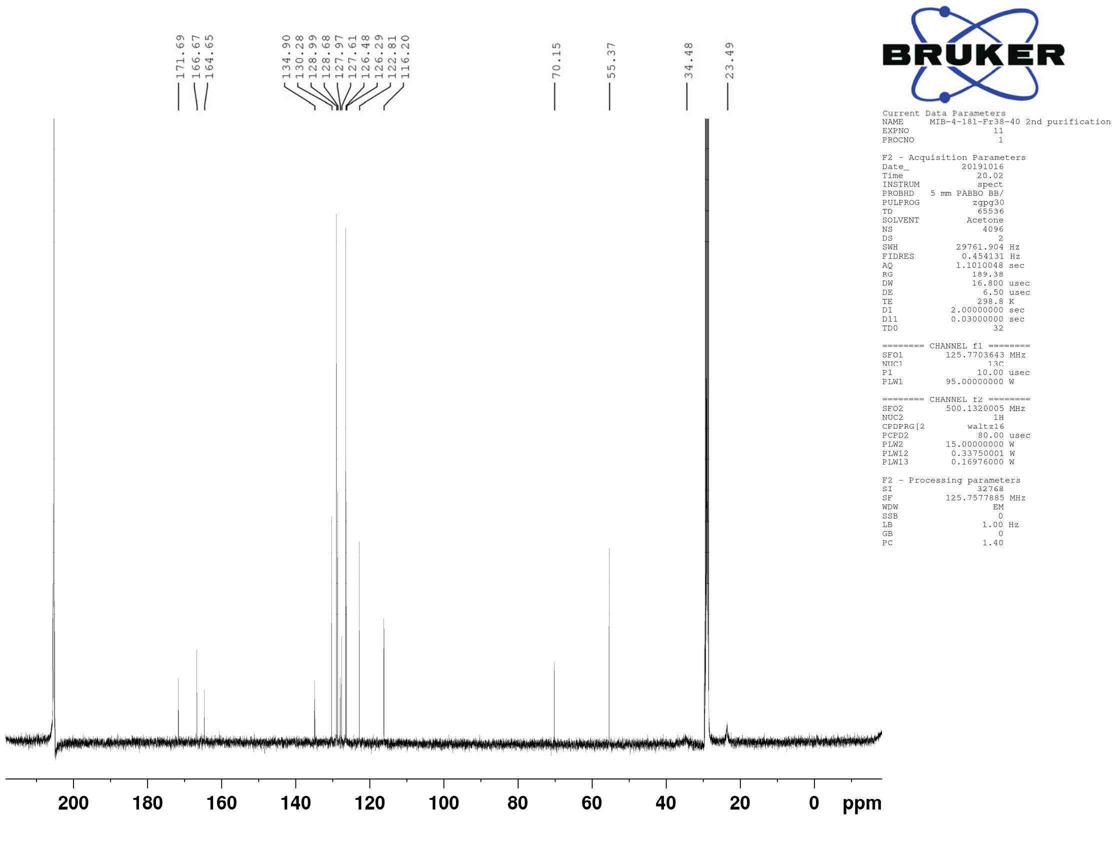

LC

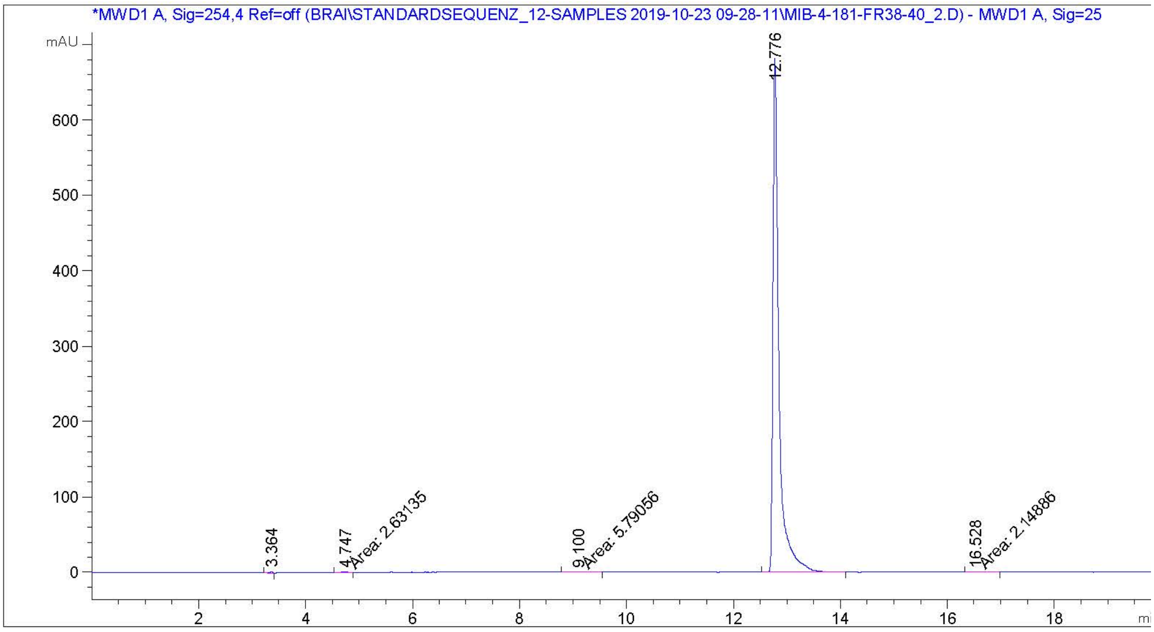

===

Area Percent Report

===

Sorted By : Signal
Multiplier : 1.0000
Dilution : 1.0000
Use Multiplier & Dilution Factor with ISTDs

Signal 1: MWD1 A, Sig=254,4 Ref=off
Signal has been modified after loading from rawdata file!

Peak #	RetTime [min]	Type	Width [min]	Area [mAU*s]	Height [mAU]	Area %
1	3.364	BB	0.0581	7.81879	1.97929	0.1455
2	4.747	MM	0.1337	2.63135	3.28028e-1	0.0490
3	9.100	MM	0.4023	5.79056	2.39877e-1	0.1078
4	12.776	BB	0.1135	5353.58447	682.41895	99.6577
5	16.528	MM	0.3350	2.14886	1.06912e-1	0.0400

Compound 23

^{1}H-NMR

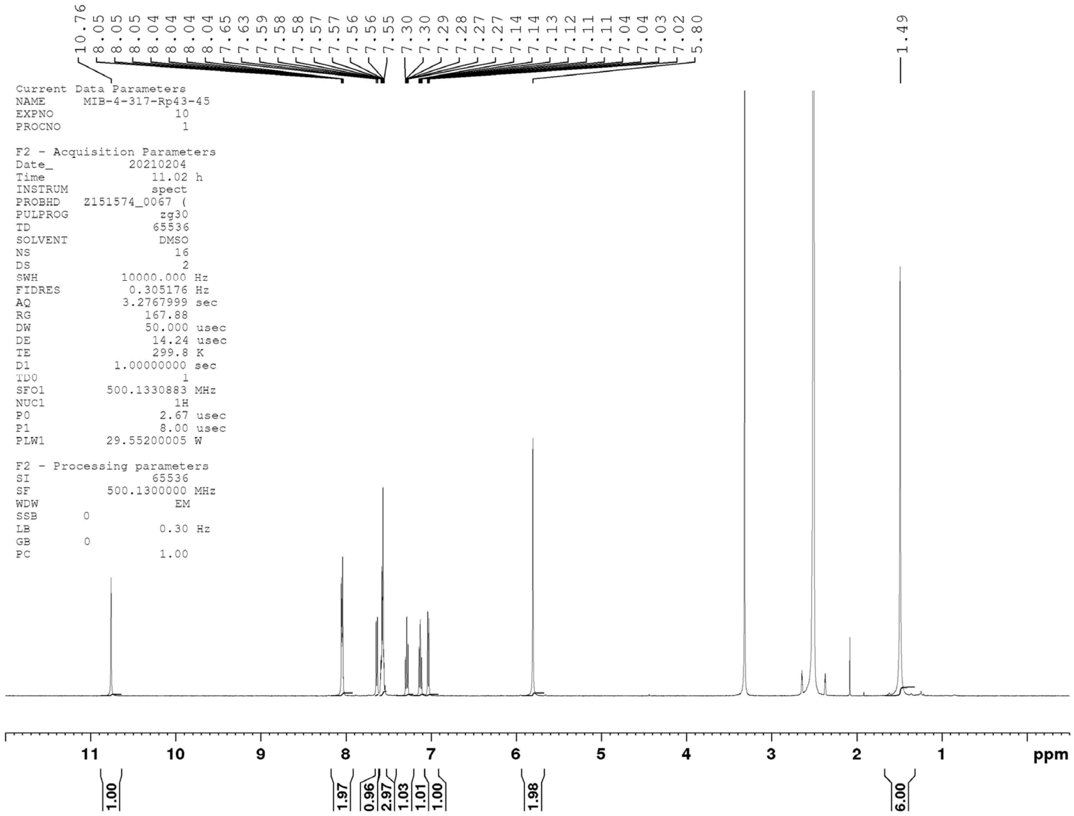

^{13}C-NMR

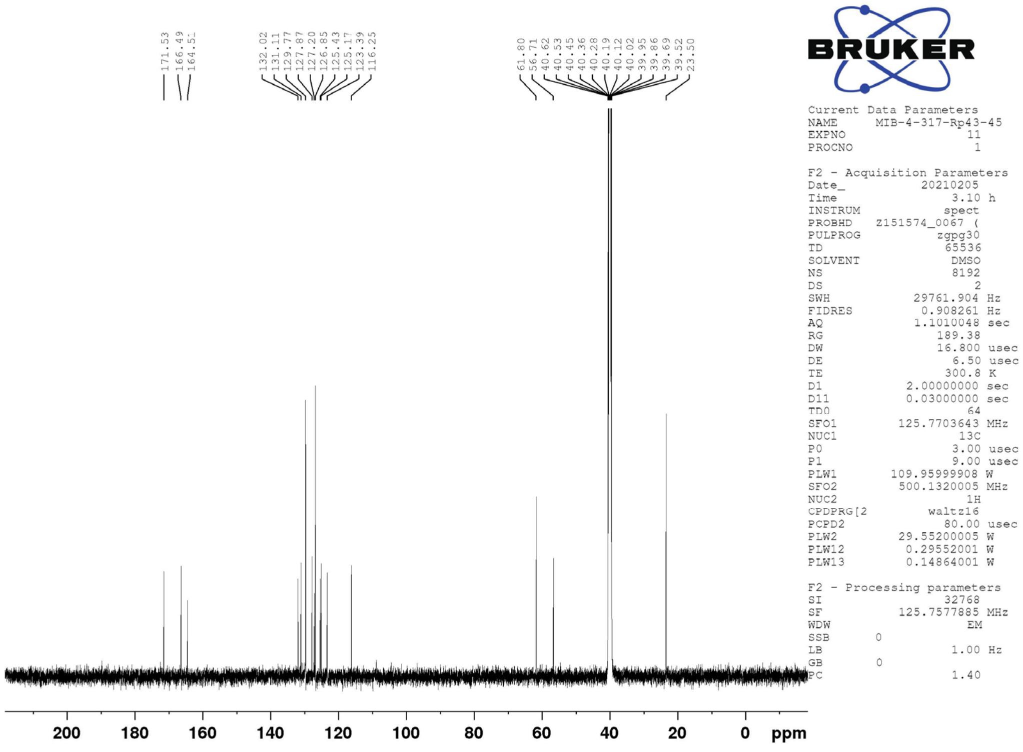

LC

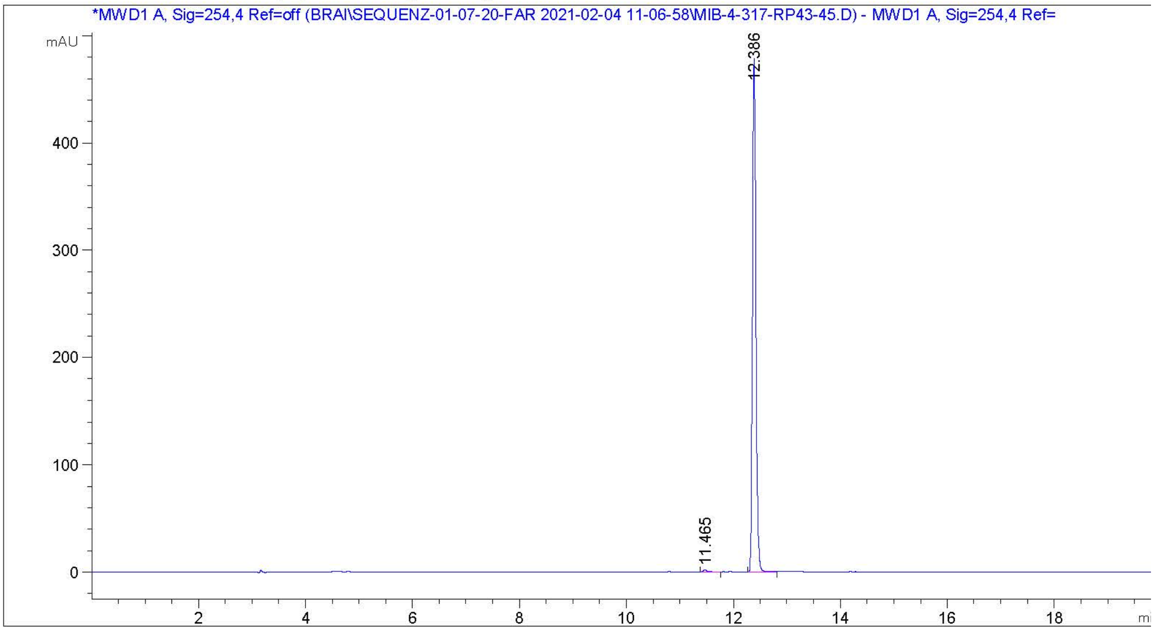


```
=====================================================================
                        Area Percent Report
=====================================================================

Sorted By             :      Signal
Multiplier            :      1.0000
Dilution              :      1.0000
Use Multiplier & Dilution Factor with ISTDs


Signal 1: MWD1 A, Sig=254,4 Ref=off
 Signal has been modified after loading from rawdata file!

Peak RetTime Type  Width     Area      Height     Area
  #   [min]        [min]   [mAU*s]     [mAU]        %
----|-------|----|-------|----------|----------|--------|
   1  11.465 BB    0.0839   12.89997    2.21576   0.6039
   2  12.386 BV    0.0675 2123.19580  480.99524  99.3961
```

Compound 24

^{1}H-NMR (25 °C)

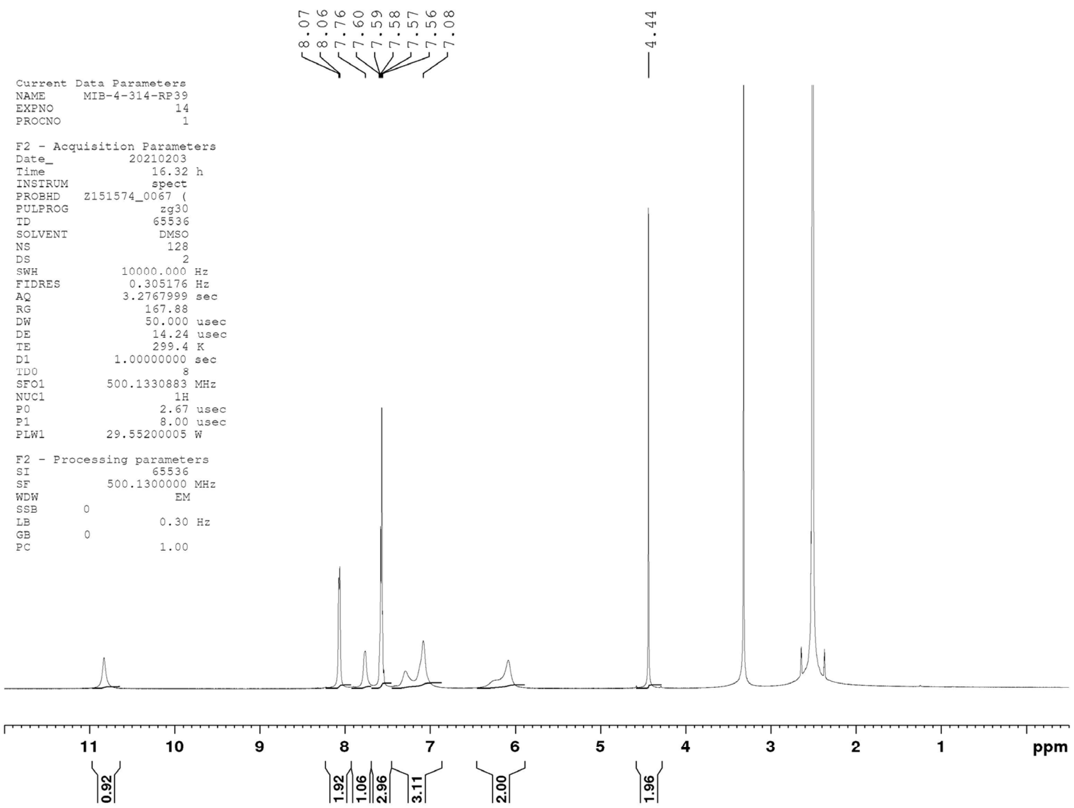

^{1}H-NMR (75 °C)

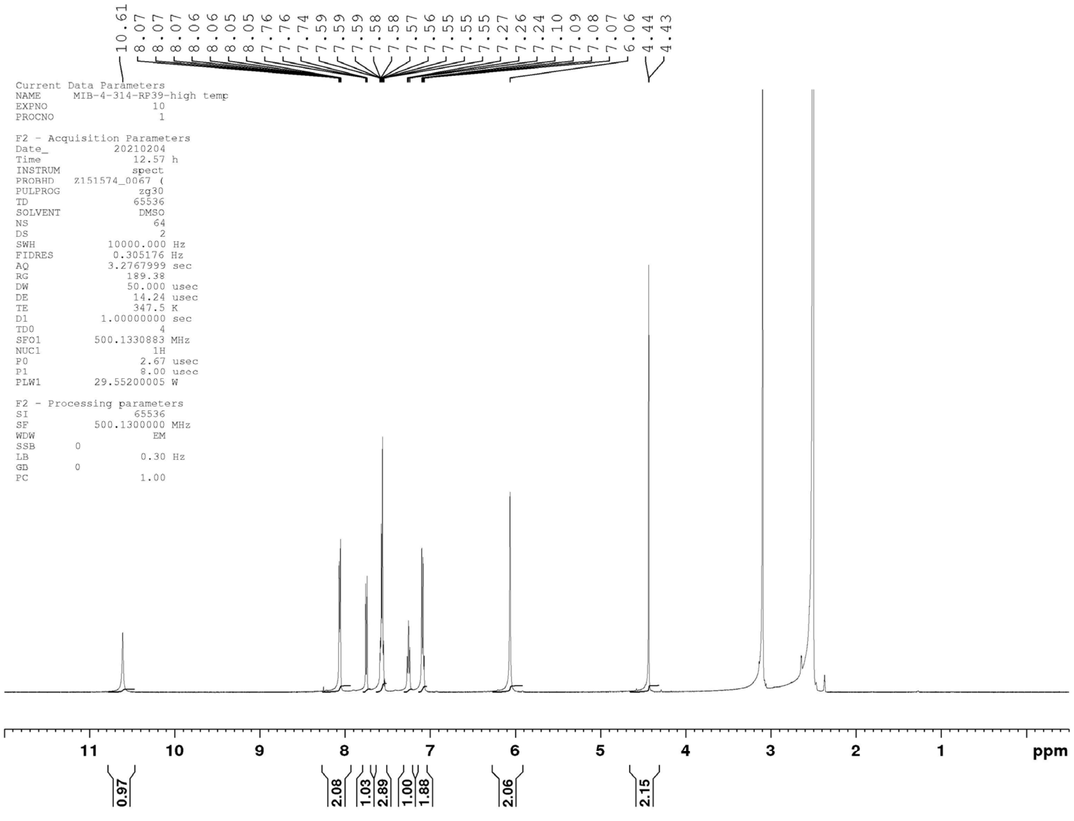

^{13}C-NMR

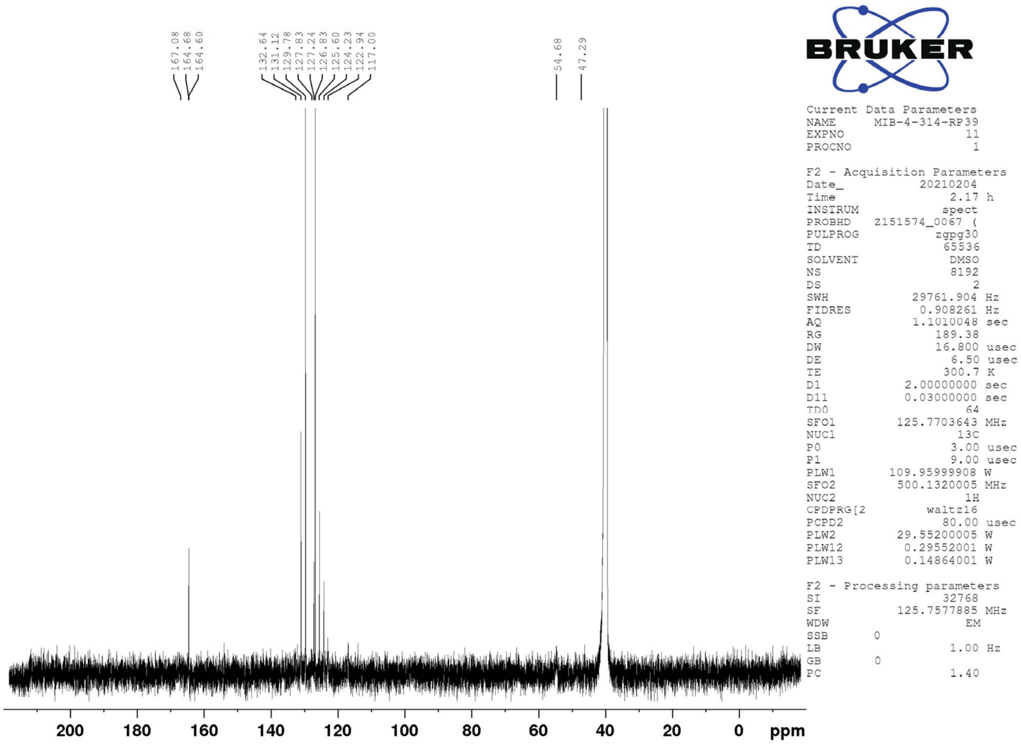
167.08
164.68
164.60
132.64
131.12
129.78
127.83
127.24
126.83
125.60
124.23
122.94
117.00
54.68
47.29
BRUKER
Current Data Parameters
NAME MIB-4-314-RP39
EXPNO 11
PROCNO 1
F2 - Acquisition Parameters
Date_ 20210204
Time 2.17 h
INSTRUM spect
PROBHD Z151574_0067 (
PULPROG zgpg30
TD 65536
SOLVENT DMSO
NS 8192
DS 2
SWH 29761.904 Hz
FIDRES 0.908261 Hz
AQ 1.1010048 sec
RG 189.38
DW 16.800 usec
DE 6.50 usec
TE 300.7 K
D1 2.00000000 sec
D11 0.03000000 sec
TD0 64
SFO1 125.7703643 MHz
NUC1 13C
P0 3.00 usec
P1 9.00 usec
PLW1 109.95999908 W
SFO2 500.1320005 MHz
NUC2 1H
CPDPRG[2 waltz16
PCPD2 80.00 usec
PLW2 29.55200005 W
PLW12 0.29552001 W
PLW13 0.14864001 W
F2 - Processing parameters
SI 32768
SF 125.7577885 MHz
WDW EM
SSB 0
LB 1.00 Hz
GB 0
PC 1.40
200
180
160
140
120
100
80
60
40
20
0
ppm

LC

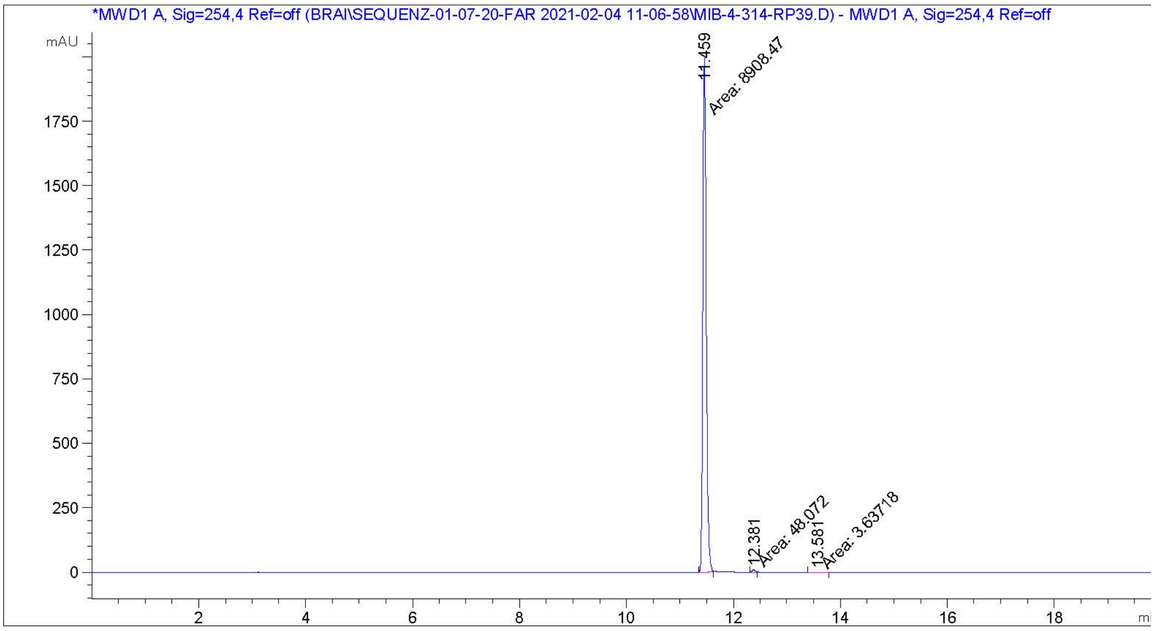


```
=====================================================================
                          Area Percent Report
=====================================================================

Sorted By             :      Signal
Multiplier            :      1.0000
Dilution              :      1.0000
Use Multiplier & Dilution Factor with ISTDs


Signal 1: MWD1 A, Sig=254,4 Ref=off
 Signal has been modified after loading from rawdata file!

Peak RetTime Type  Width     Area      Height     Area
  #   [min]        [min]   [mAU*s]     [mAU]        %
----|-------|----|-------|----------|----------|--------|
   1  11.459 MM    0.0740 8908.46582 2005.36218 99.4229
   2  12.381 MM    0.0762   48.07204   10.52061  0.5365
   3  13.581 MM    0.1841    3.63718 3.29313e-1  0.0406
```

Compound 26

^{1}H-NMR

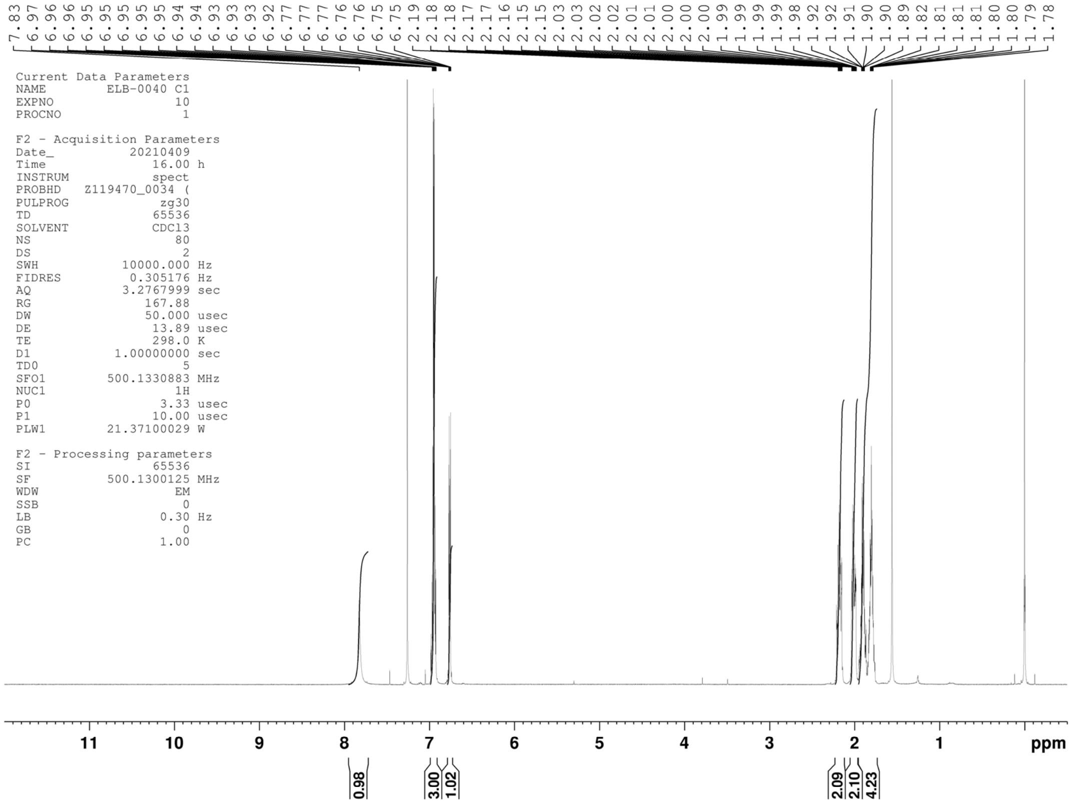

^{13}C-NMR

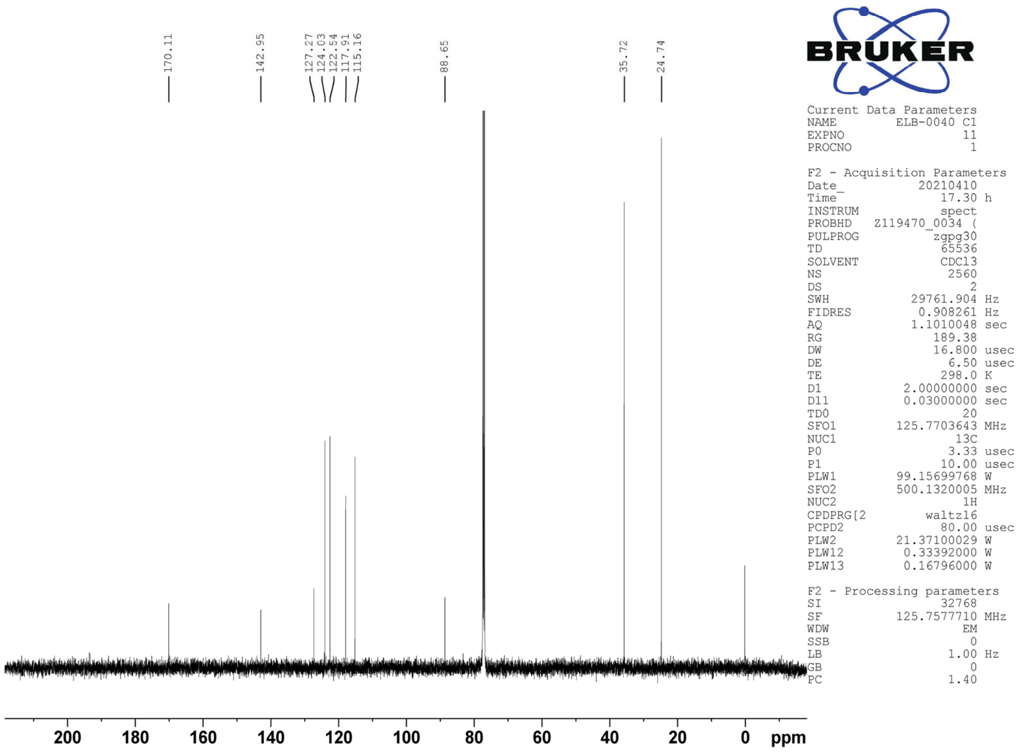

LC

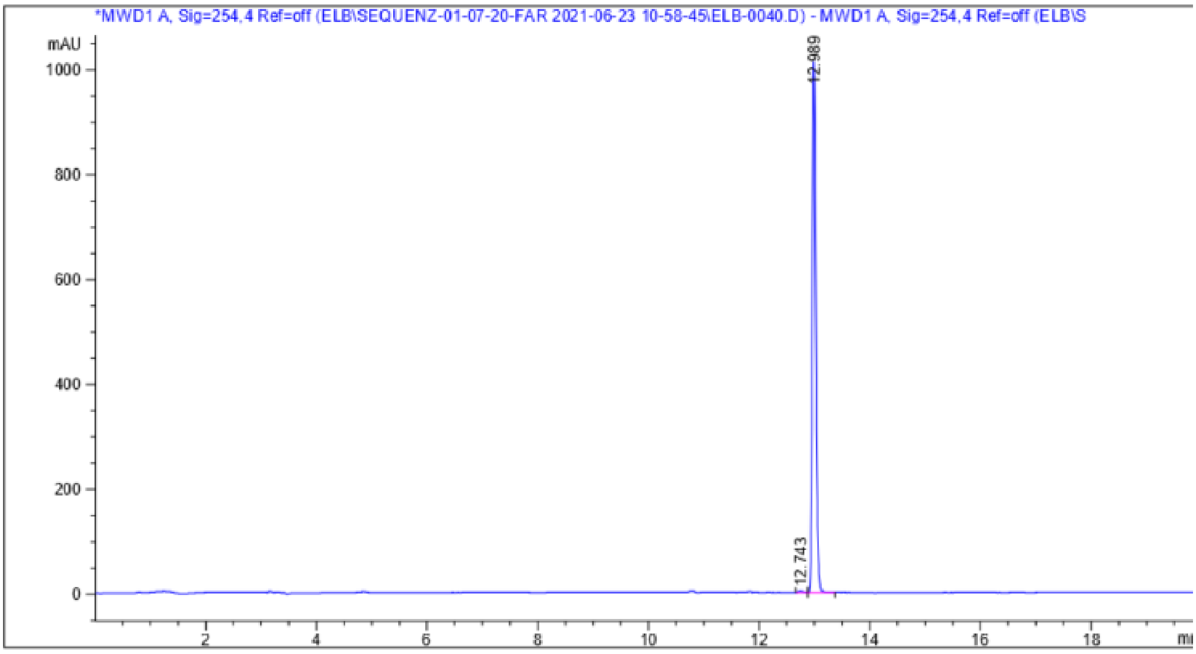

Area Percent Report

Sorted By : Signal
Multiplier : 1.0000
Dilution : 1.0000
Use Multiplier & Dilution Factor with ISTDs

Signal 1: MWD1 A, Sig-254,4 Ref-off
Signal has been modified after loading from rawdata file!

Peak #	RetTime [min]	Type	Width [min]	Area [mAU*s]	Height [mAU]	Area %
1	12.743	BV	0.1010	15.37414	2.32849	0.3485
2	12.989	VV	0.0666	4395.62646	1013.22925	99.6515

Compound 27

^{1}H-NMR

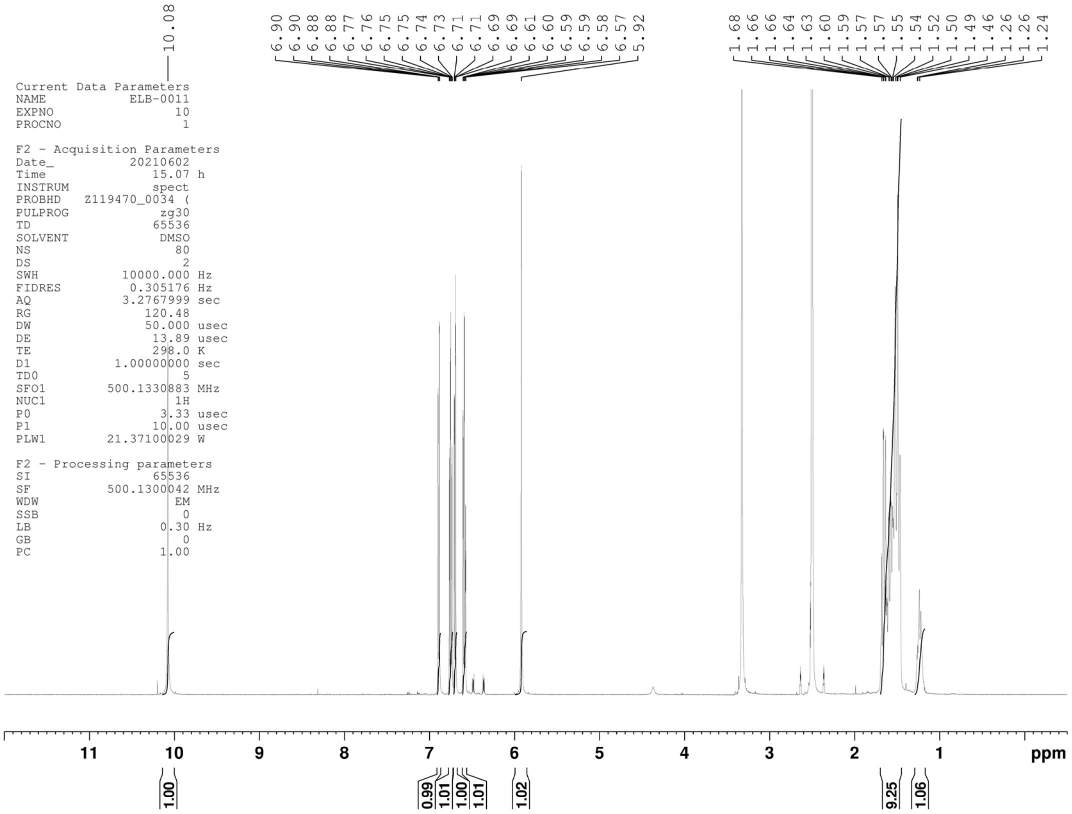

^{13}C-NMR

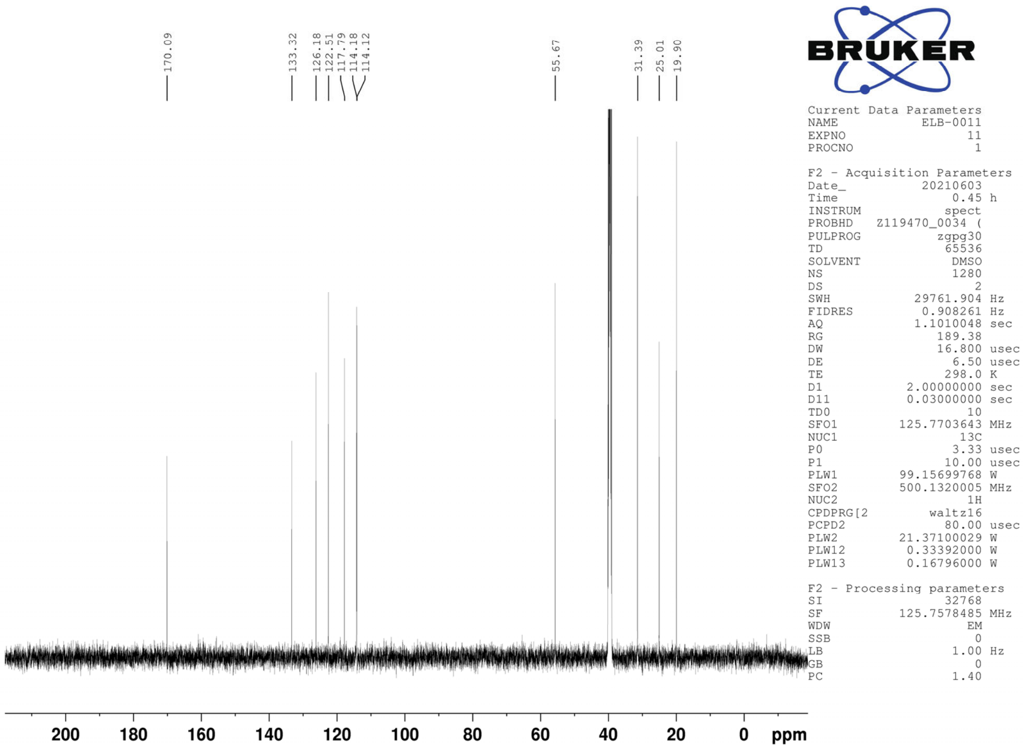

LC

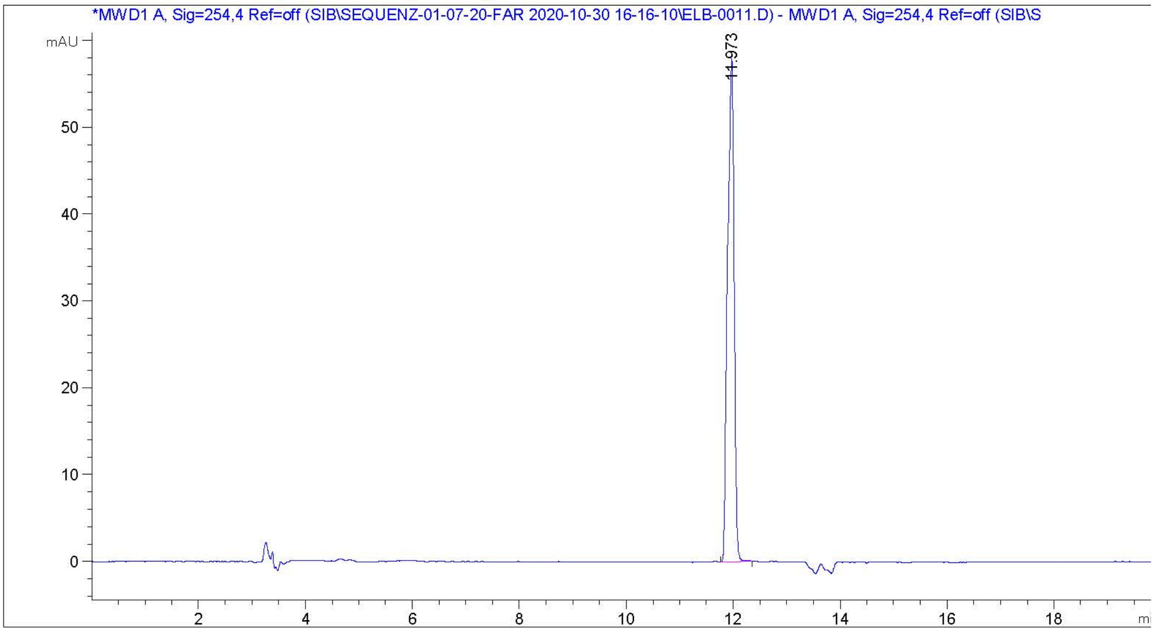


```
=====================================================================
                         Area Percent Report
=====================================================================

Sorted By             :      Signal
Multiplier            :      1.0000
Dilution              :      1.0000
Use Multiplier & Dilution Factor with ISTDs


Signal 1: MWD1 A, Sig=254,4 Ref=off
 Signal has been modified after loading from rawdata file!

Peak RetTime Type  Width     Area      Height     Area
  #   [min]        [min]   [mAU*s]     [mAU]        %
----|-------|----|-------|----------|----------|--------|
   1  11.973 BB    0.1243  498.50531   57.97225 100.0000

Totals :                   498.50531   57.97225
```

Compound 28

^{1}H-NMR

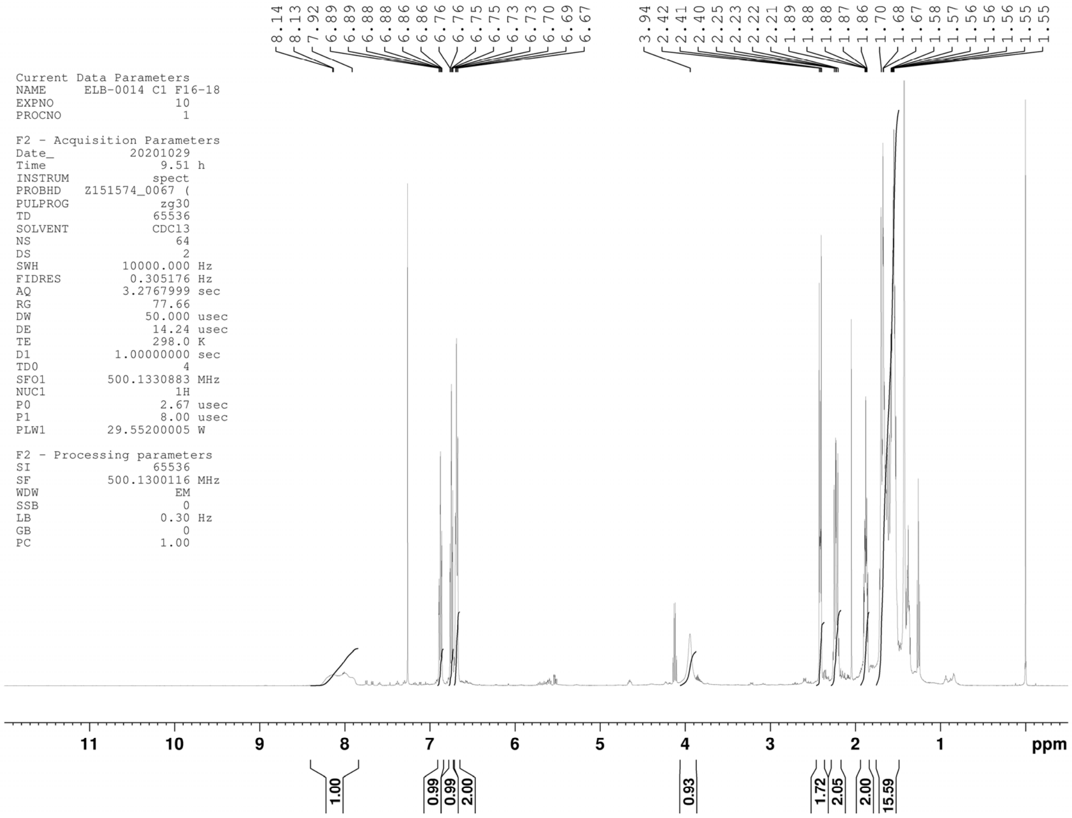

^{13}C-NMR

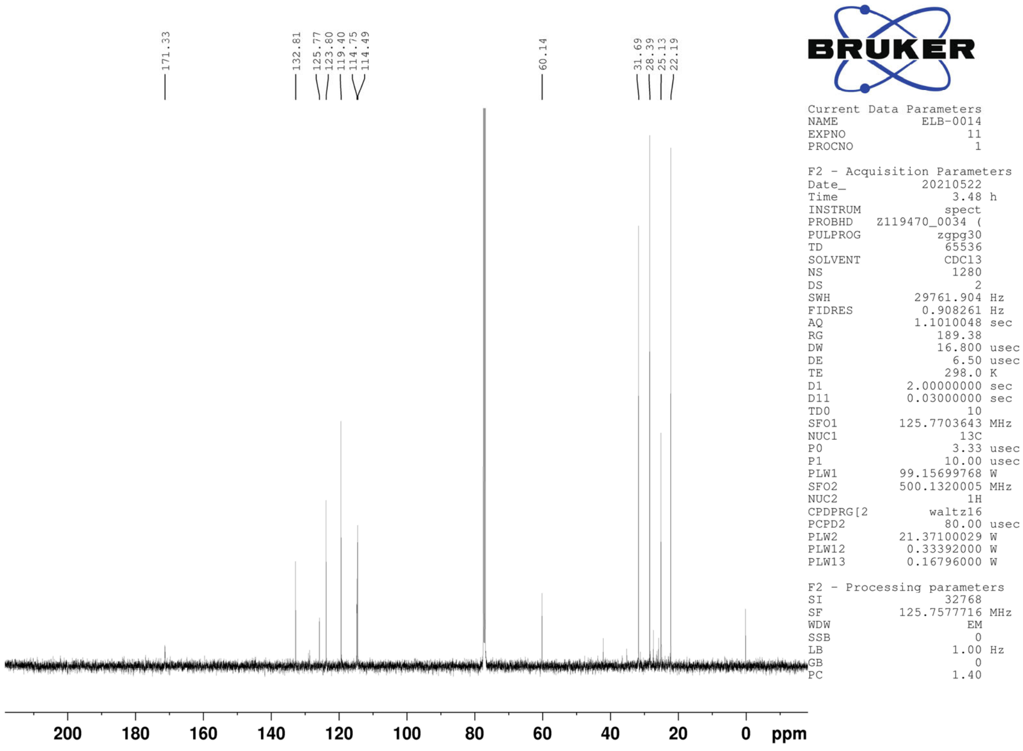

LC

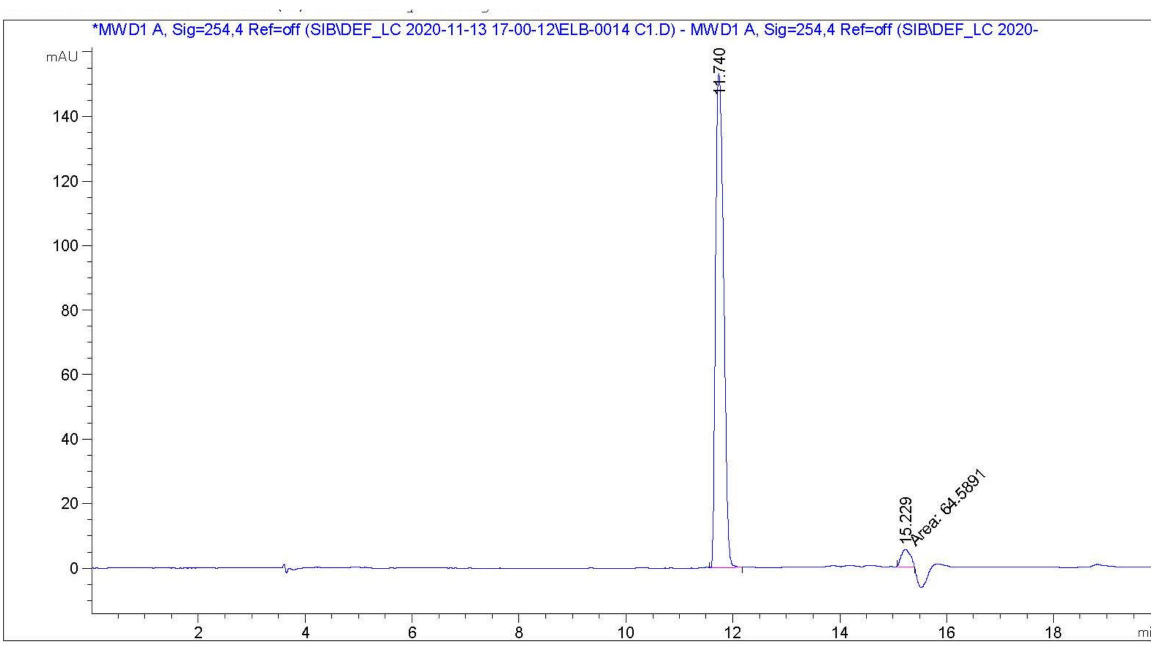


```
=====================================================================
                         Area Percent Report
=====================================================================

Sorted By             :      Signal
Multiplier            :      1.0000
Dilution              :      1.0000
Use Multiplier & Dilution Factor with ISTDs


Signal 1: MWD1 A, Sig=254,4 Ref=off
 Signal has been modified after loading from rawdata file!

Peak RetTime Type  Width     Area      Height     Area
  #   [min]        [min]   [mAU*s]     [mAU]        %
----|-------|----|-------|----------|----------|--------|
   1  11.740 VB    0.1715 1595.30127  153.24013  96.1088
   2  15.229 MM    0.1993   64.58913    5.40105   3.8912

Totals :                  1659.89040  158.64118
```

Compound 29

^{1}H-NMR

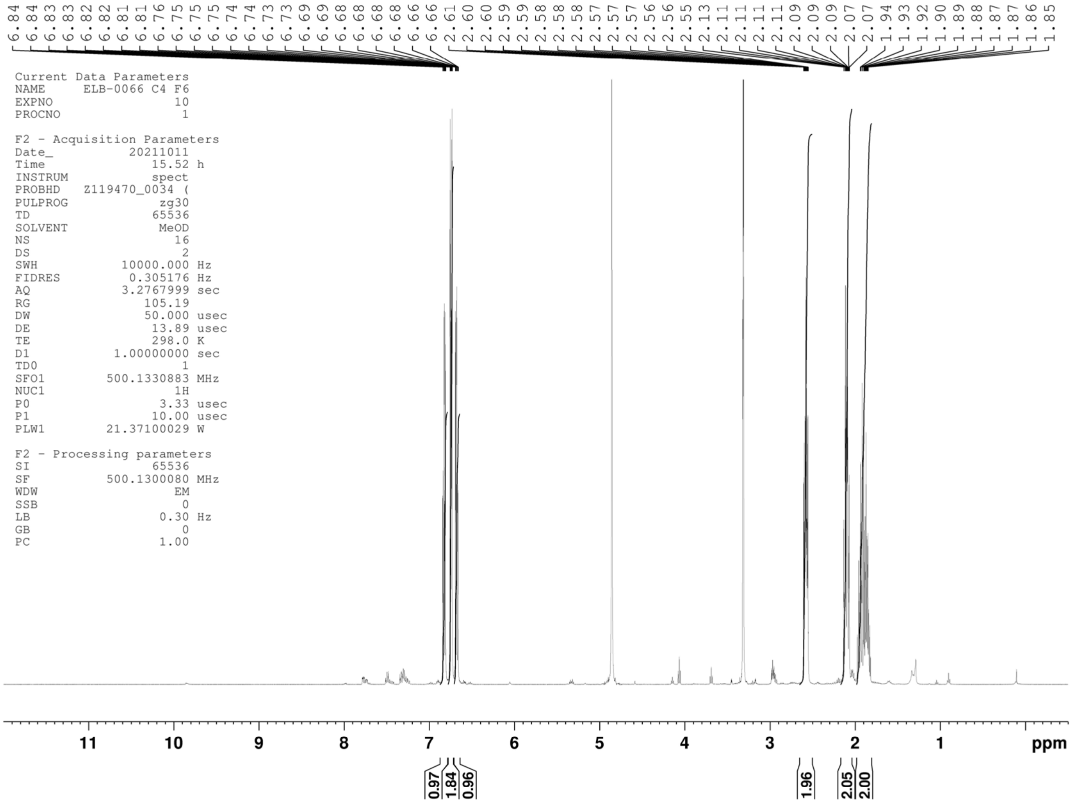

^{13}C-NMR

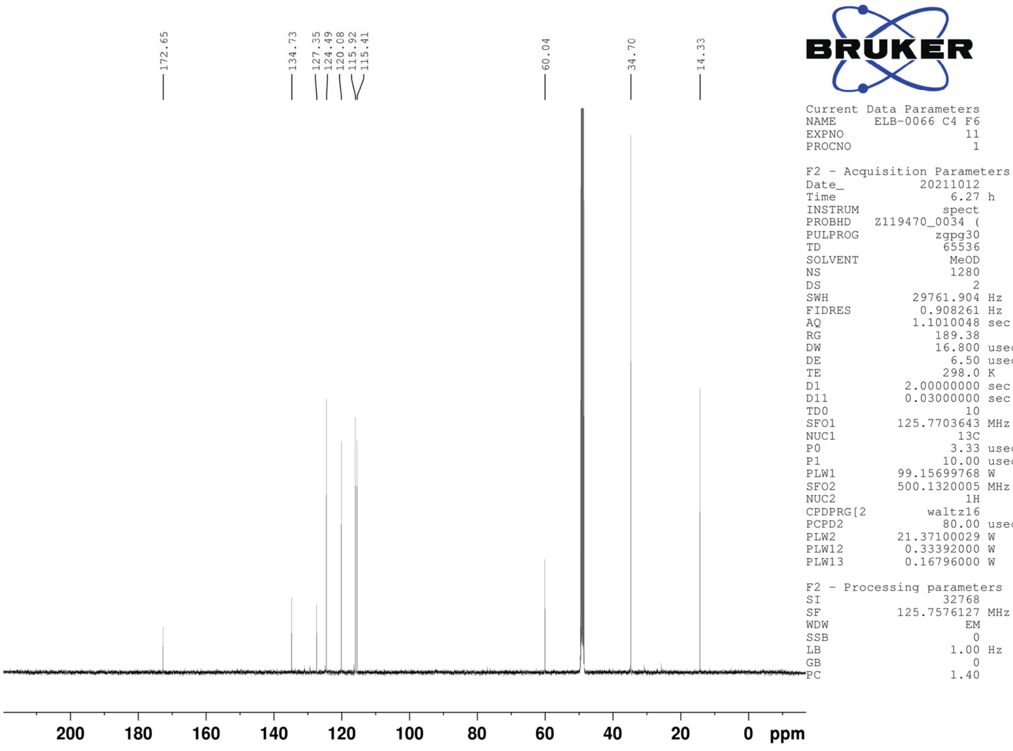

LC

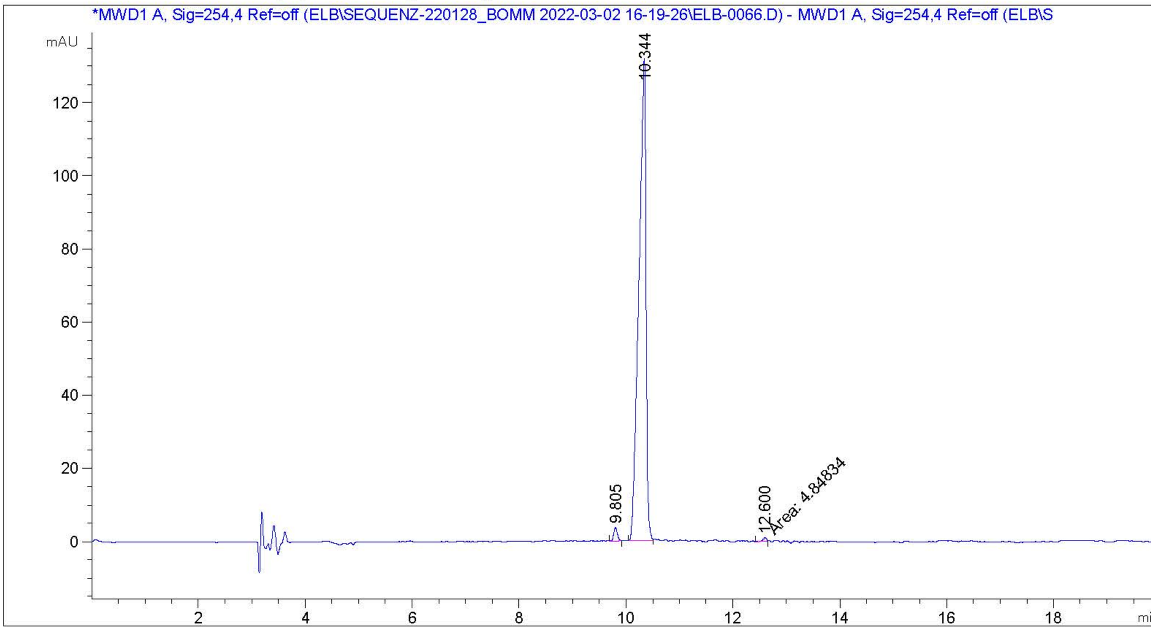


```
=====================================================================
                         Area Percent Report
=====================================================================

Sorted By             :      Signal
Multiplier            :      1.0000
Dilution              :      1.0000
Use Multiplier & Dilution Factor with ISTDs


Signal 1: MWD1 A, Sig=254,4 Ref=off
 Signal has been modified after loading from rawdata file!

Peak RetTime Type  Width     Area      Height     Area
  #   [min]        [min]   [mAU*s]     [mAU]        %
----|-------|----|-------|----------|----------|--------|
   1   9.805 VV    0.0733   18.23354    3.71084   1.4620
   2  10.344 VV    0.1241 1224.05200  132.16112  98.1492
   3  12.600 MM    0.0820    4.84834 9.85984e-1   0.3888

Totals :                  1247.13388  136.85794
```

Compound 30

^{1}H-NMR

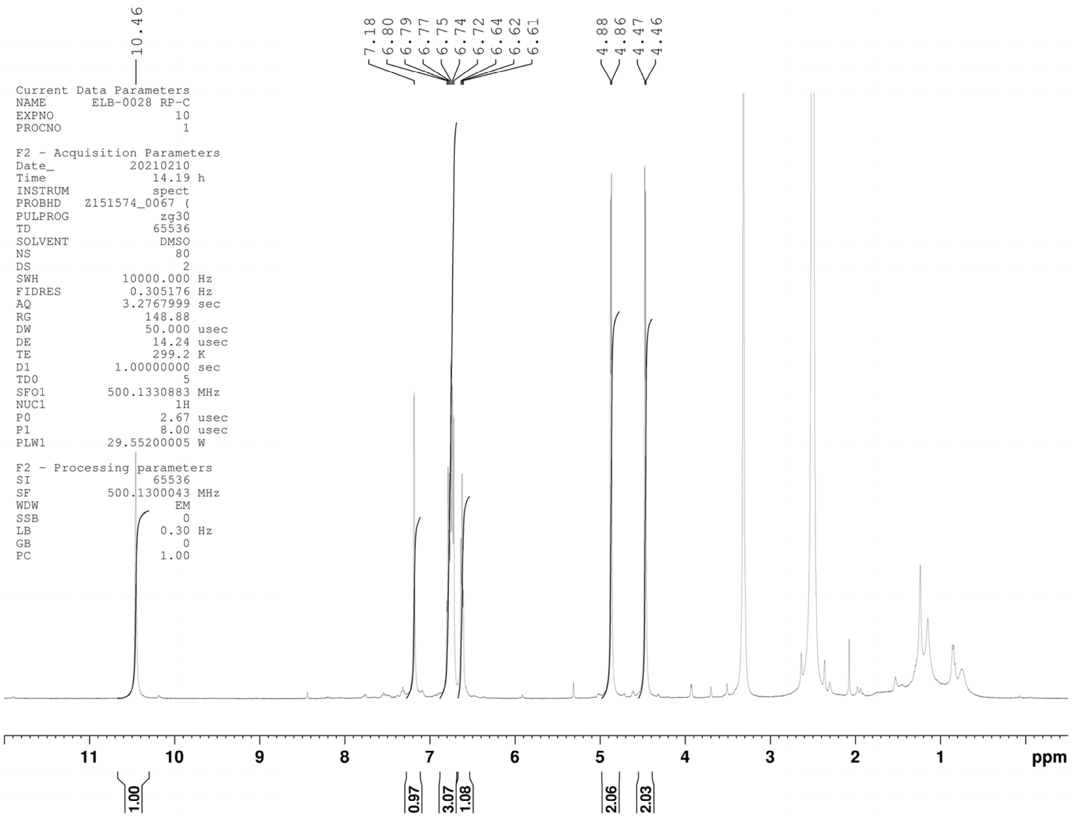

^{13}C-NMR

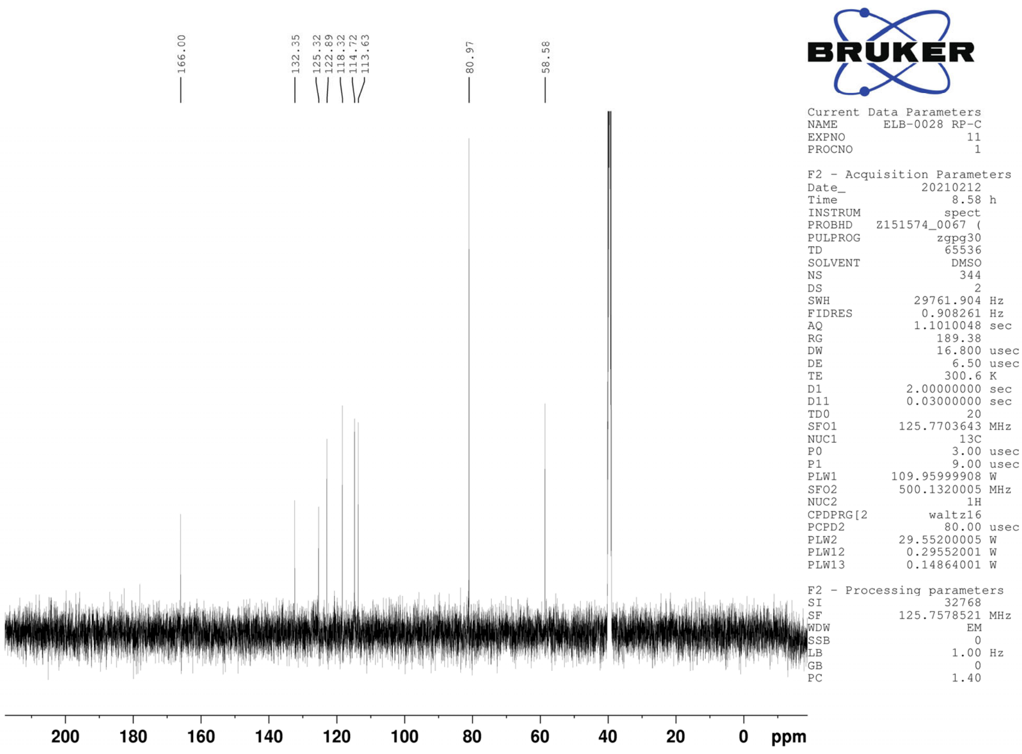

LC

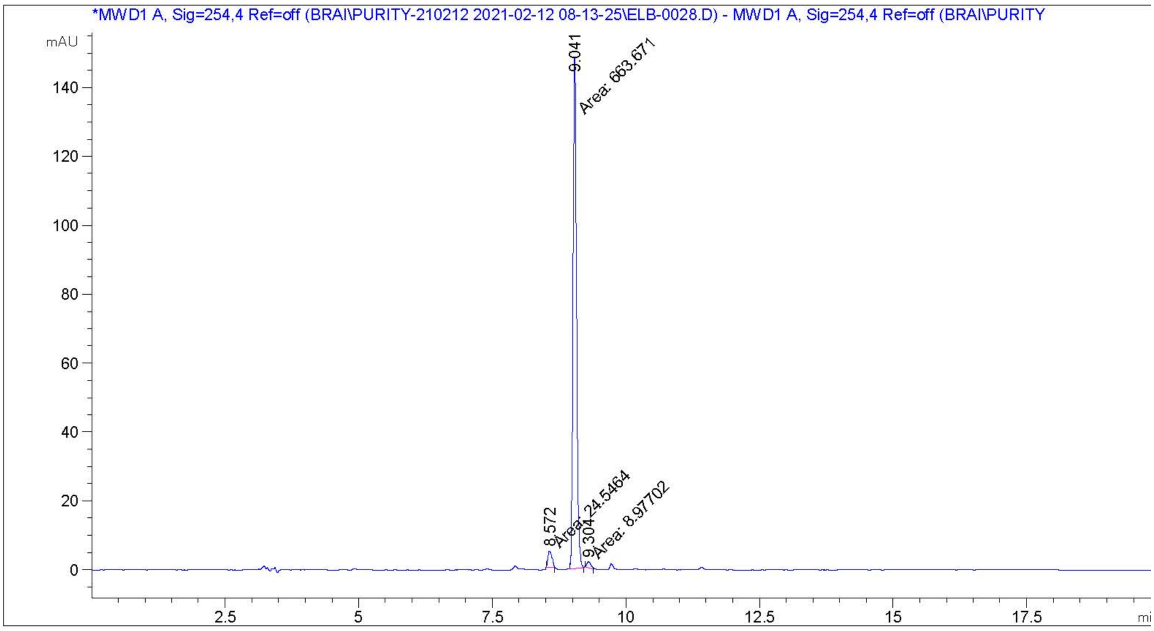

===

Area Percent Report

===

Sorted By : Signal
Multiplier : 1.0000
Dilution : 1.0000
Use Multiplier & Dilution Factor with ISTDs

Signal 1: MWD1 A, Sig=254,4 Ref=off
Signal has been modified after loading from rawdata file!

Peak #	RetTime [min]	Type	Width [min]	Area [mAU*s]	Height [mAU]	Area %
1	8.572	MM	0.0860	24.54641	4.75823	3.5207
2	9.041	MM	0.0741	663.67084	149.22447	95.1917
3	9.304	MM	0.0904	8.97702	1.65500	1.2876

[illegible]

Compound 31

^{1}H-NMR

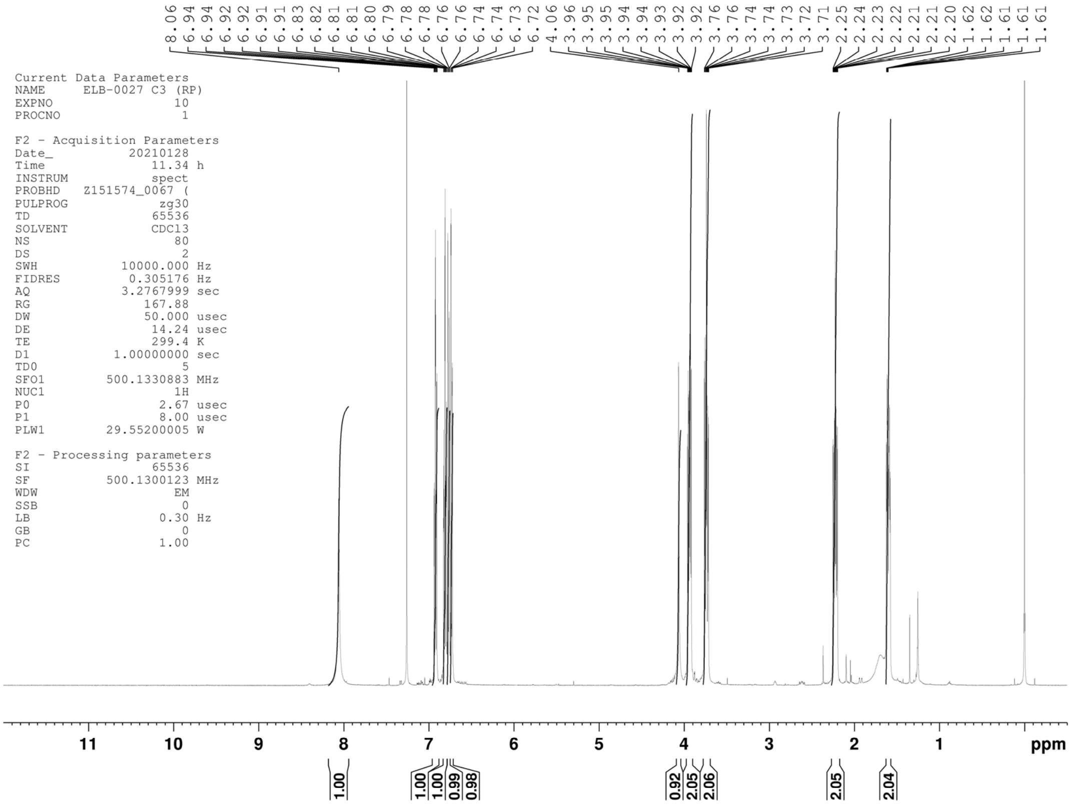

^{13}C-NMR

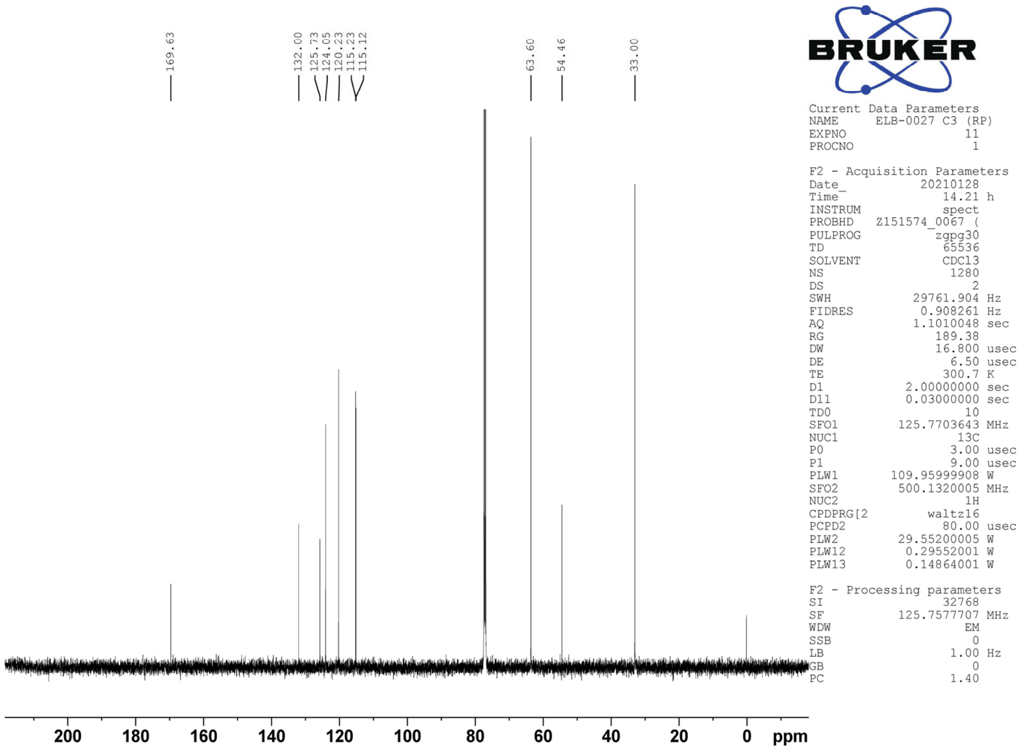

LC

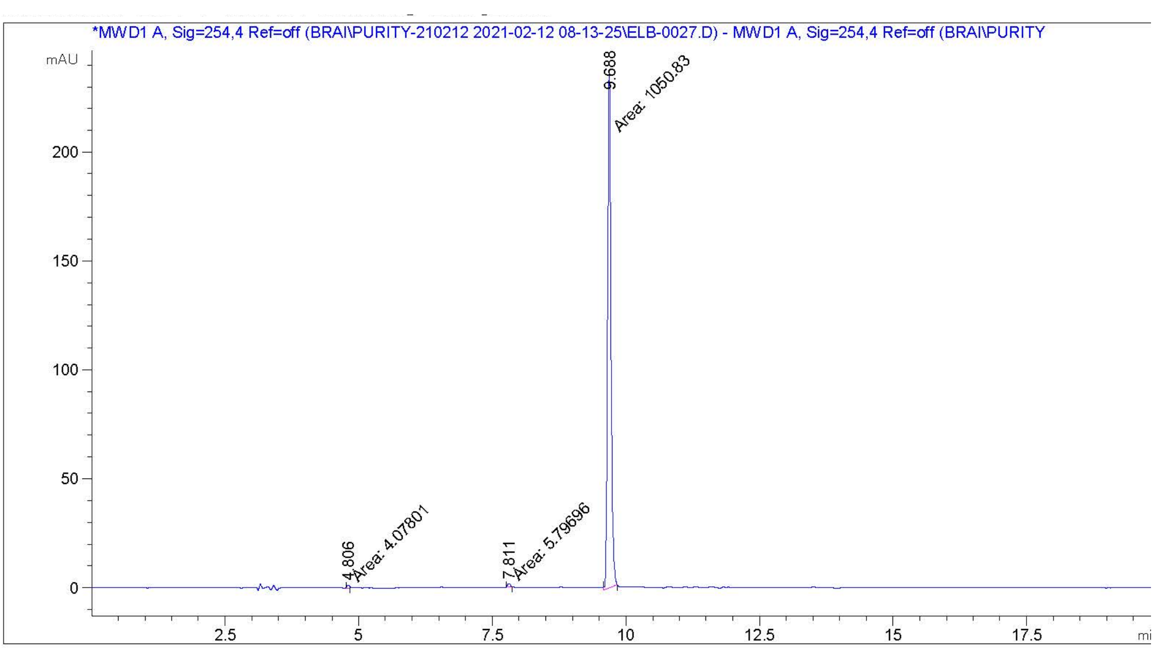


```
=====================================================================
                         Area Percent Report
=====================================================================

Sorted By             :      Signal
Multiplier            :      1.0000
Dilution              :      1.0000
Use Multiplier & Dilution Factor with ISTDs


Signal 1: MWD1 A, Sig=254,4 Ref=off
 Signal has been modified after loading from rawdata file!

Peak RetTime Type  Width     Area      Height     Area
  #   [min]        [min]   [mAU*s]     [mAU]        %
----|-------|----|-------|----------|----------|--------|
   1   4.806 MM    0.0548    4.07801    1.24037   0.3845
   2   7.811 MM    0.0585    5.79696    1.65210   0.5465
   3   9.688 MM    0.0741 1050.83008  236.41370  99.0690

Totals :                  1060.70505  239.30617
```

Compound 32

^{1}H-NMR

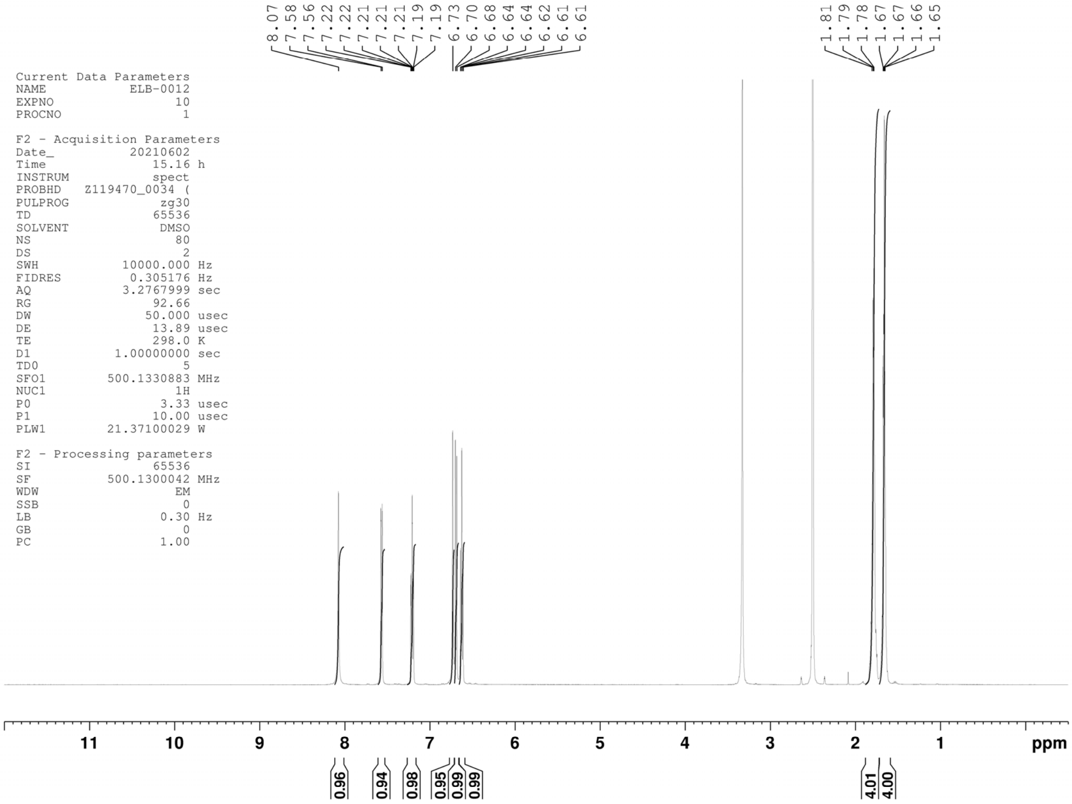

^{13}C-NMR

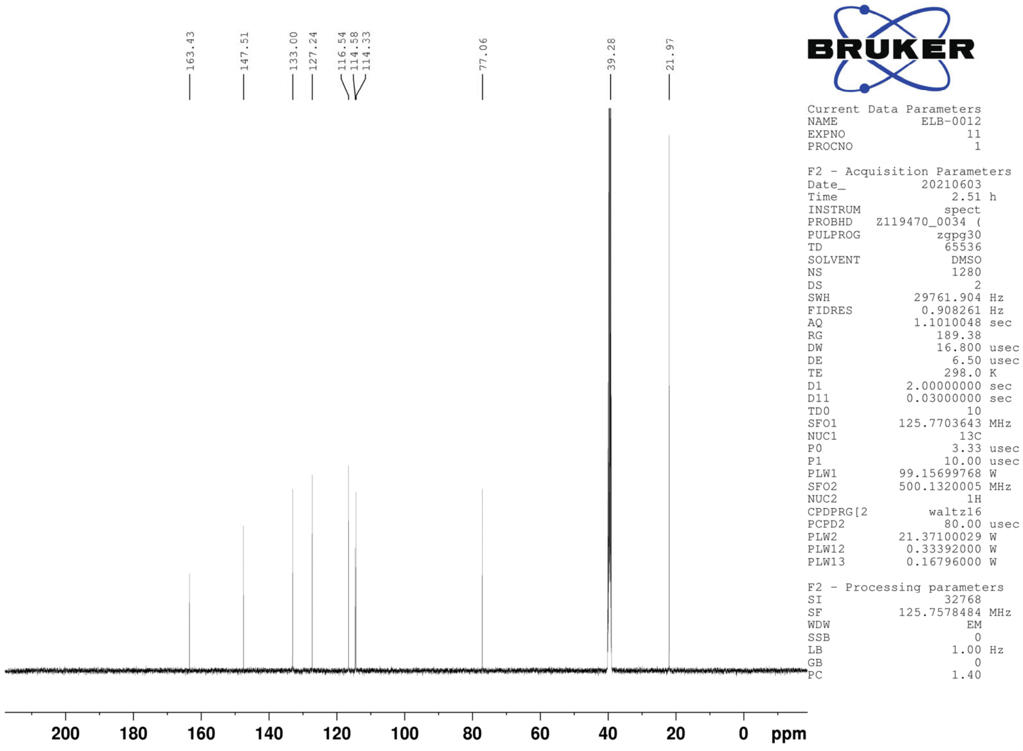

LC

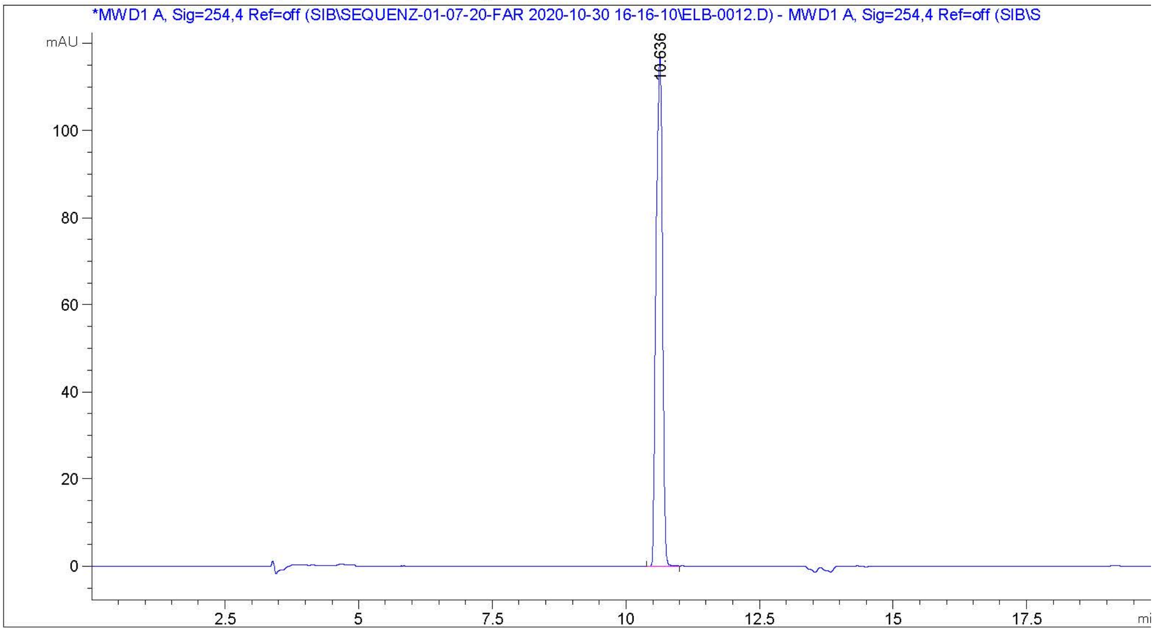


```
=====================================================================
                         Area Percent Report
=====================================================================

Sorted By             :      Signal
Multiplier            :      1.0000
Dilution              :      1.0000
Use Multiplier & Dilution Factor with ISTDs


Signal 1: MWD1 A, Sig=254,4 Ref=off
 Signal has been modified after loading from rawdata file!

Peak RetTime Type  Width     Area      Height     Area
  #   [min]        [min]   [mAU*s]     [mAU]        %
----|-------|----|-------|----------|----------|--------|
   1  10.636 BB    0.1378  957.72064  116.57840 100.0000

Totals :                   957.72064  116.57840
```

Compound 33

^{1}H-NMR

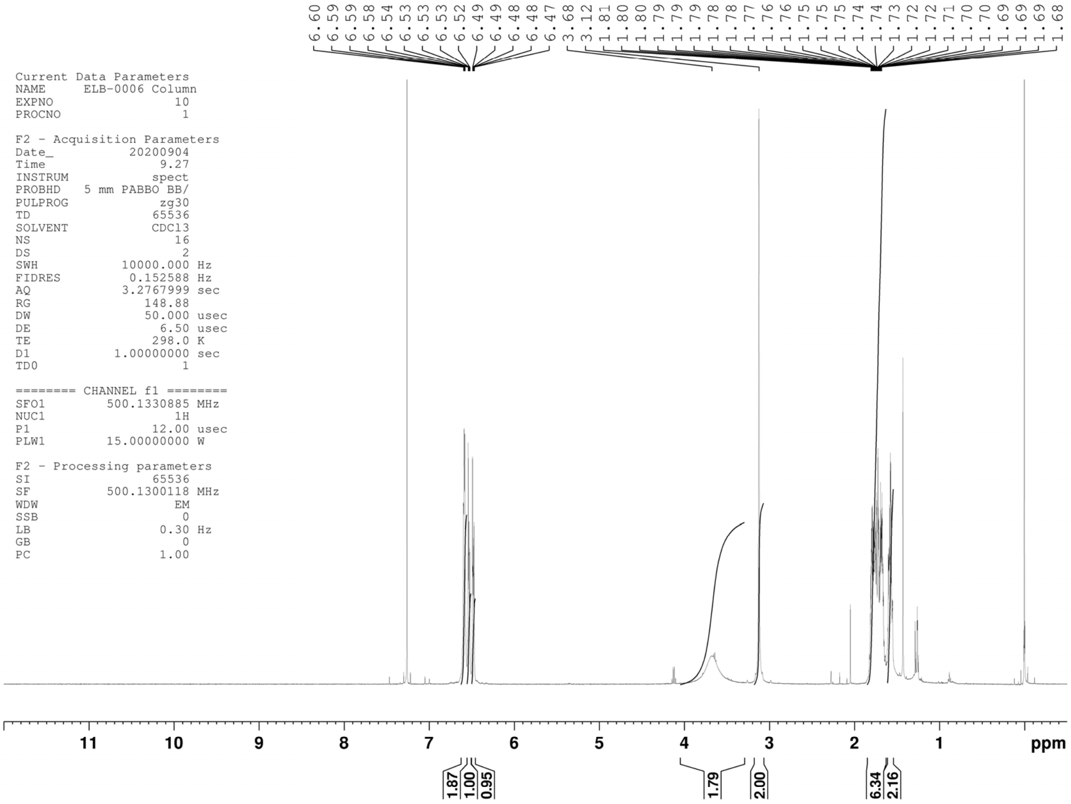

^{13}C-NMR

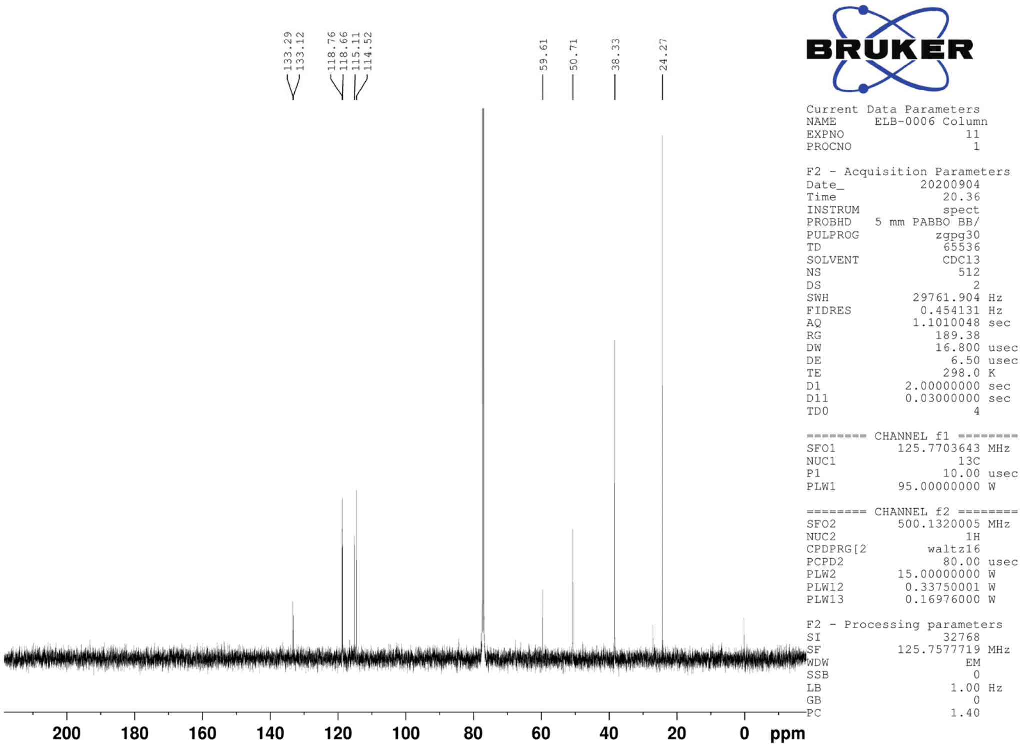

LC

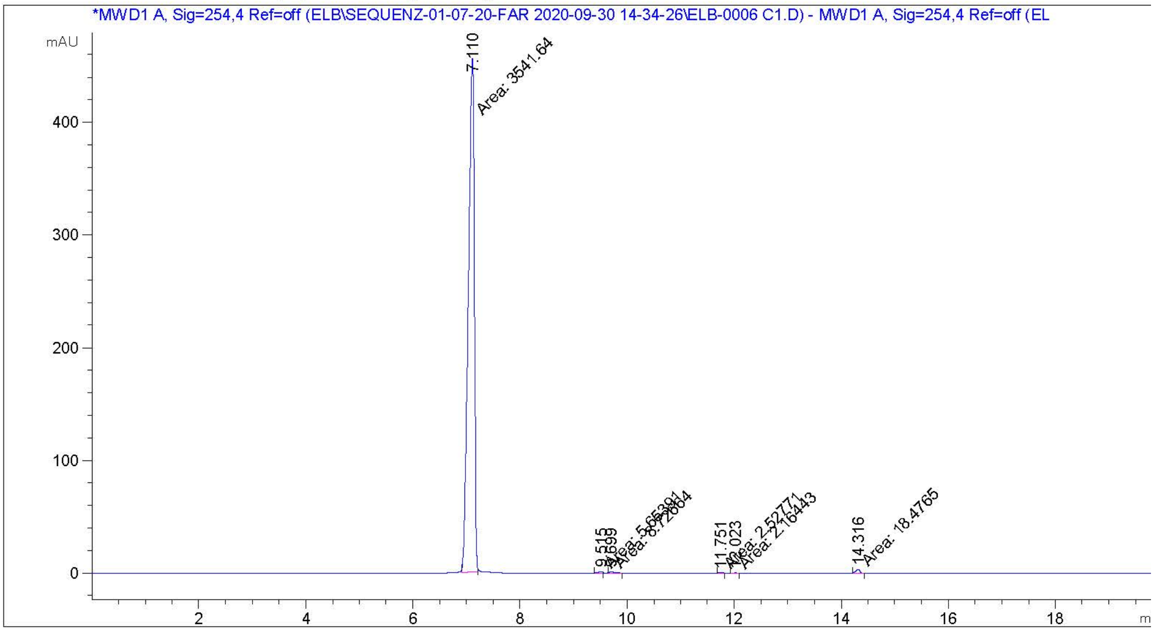


```
=====================================================================
                         Area Percent Report
=====================================================================

Sorted By             :      Signal
Multiplier            :      1.0000
Dilution              :      1.0000
Use Multiplier & Dilution Factor with ISTDs


Signal 1: MWD1 A, Sig=254,4 Ref=off
 Signal has been modified after loading from rawdata file!
```

Peak #	RetTime [min]	Type	Width [min]	Area [mAU*s]	Height [mAU]	Area %
1	7.110	MM T	0.1294	3541.63672	456.25479	98.9509
2	9.515	MM T	0.0947	5.65391	9.95205e-1	0.1580
3	9.699	MM T	0.1257	8.72664	1.15742	0.2438
4	11.751	MM T	0.0934	2.52771	4.51045e-1	0.0706
5	12.023	MM T	0.1099	2.16443	3.28346e-1	0.0605
6	14.316	MM T	0.0921	18.47653	3.34177	0.5162

Compound 34

^{1}H-NMR

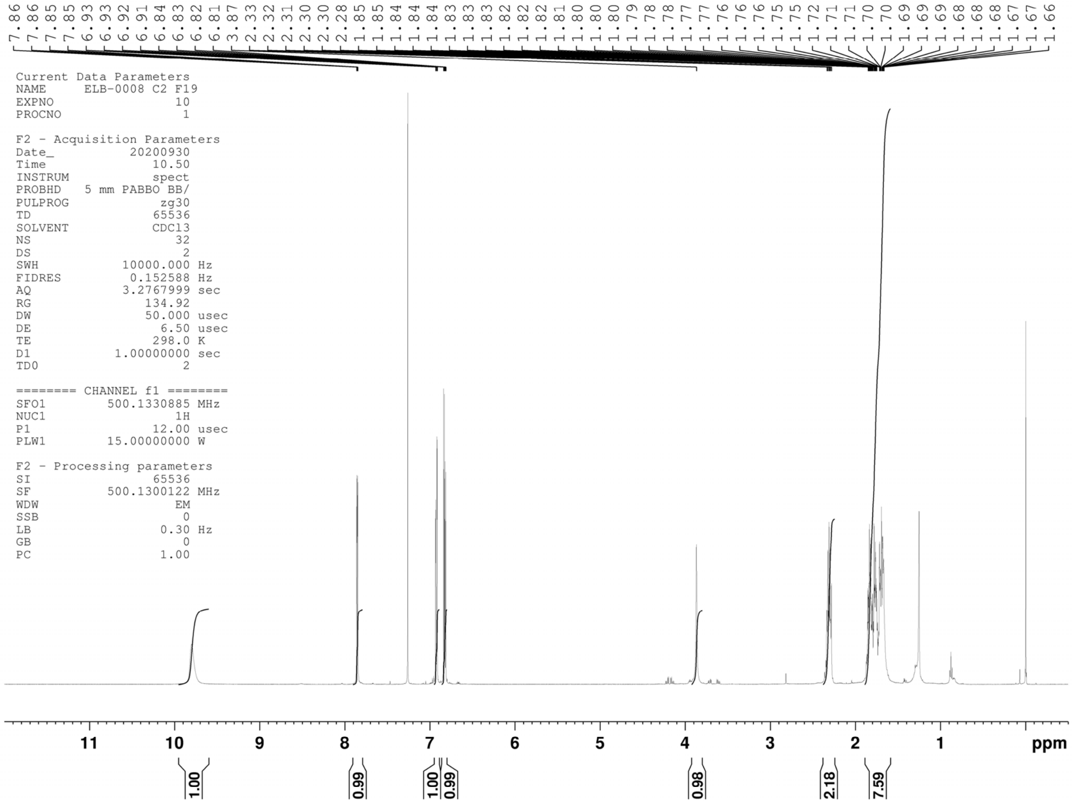

^{13}C-NMR

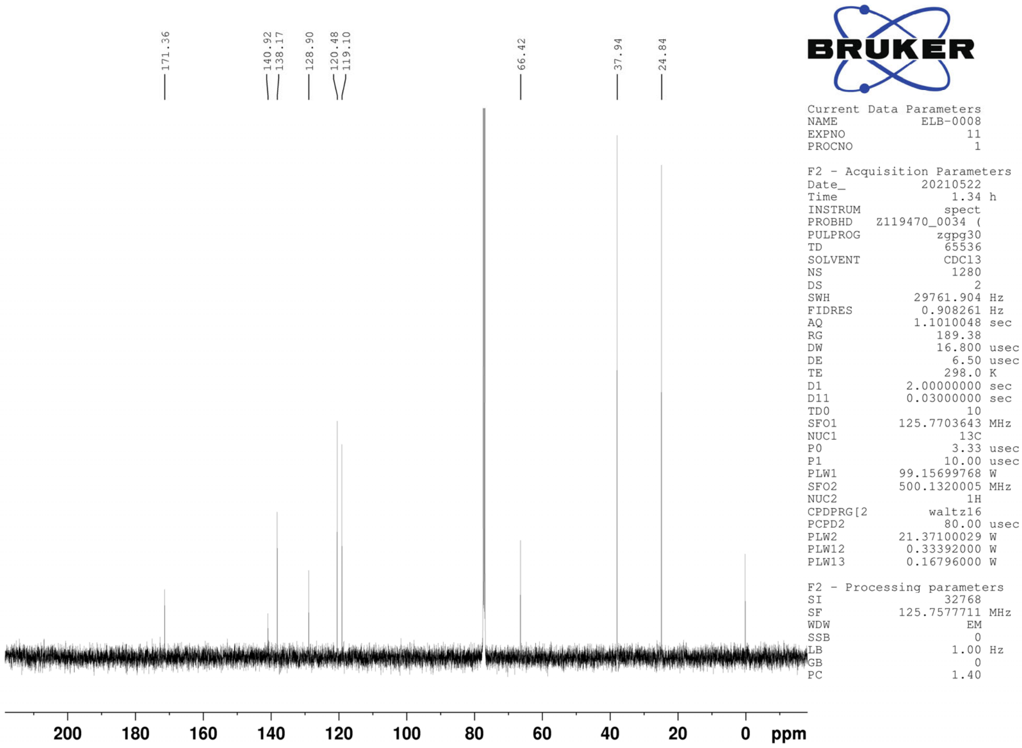

LC

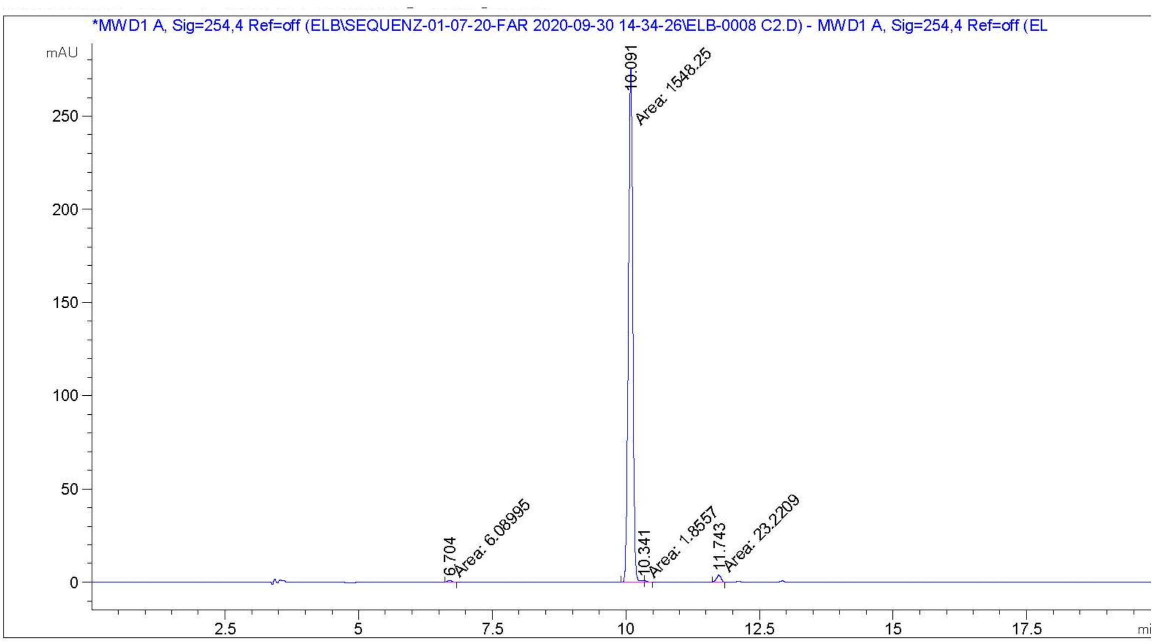

===

Area Percent Report

===

Sorted By : Signal
Multiplier : 1.0000
Dilution : 1.0000
Use Multiplier & Dilution Factor with ISTDs

Signal 1: MWD1 A, Sig=254,4 Ref=off
Signal has been modified after loading from rawdata file!

Peak #	RetTime [min]	Type	Width [min]	Area [mAU*s]	Height [mAU]	Area %
1	6.704	MM T	0.0994	6.08995	1.02136	0.3856
2	10.091	MF T	0.0936	1548.24792	275.67526	98.0267
3	10.341	FM T	0.0590	1.85570	5.24229e-1	0.1175
4	11.743	MM T	0.1006	23.22087	3.84733	1.4702

Totals : 1579.41445 281.06818

Compound 35

^{1}H-NMR

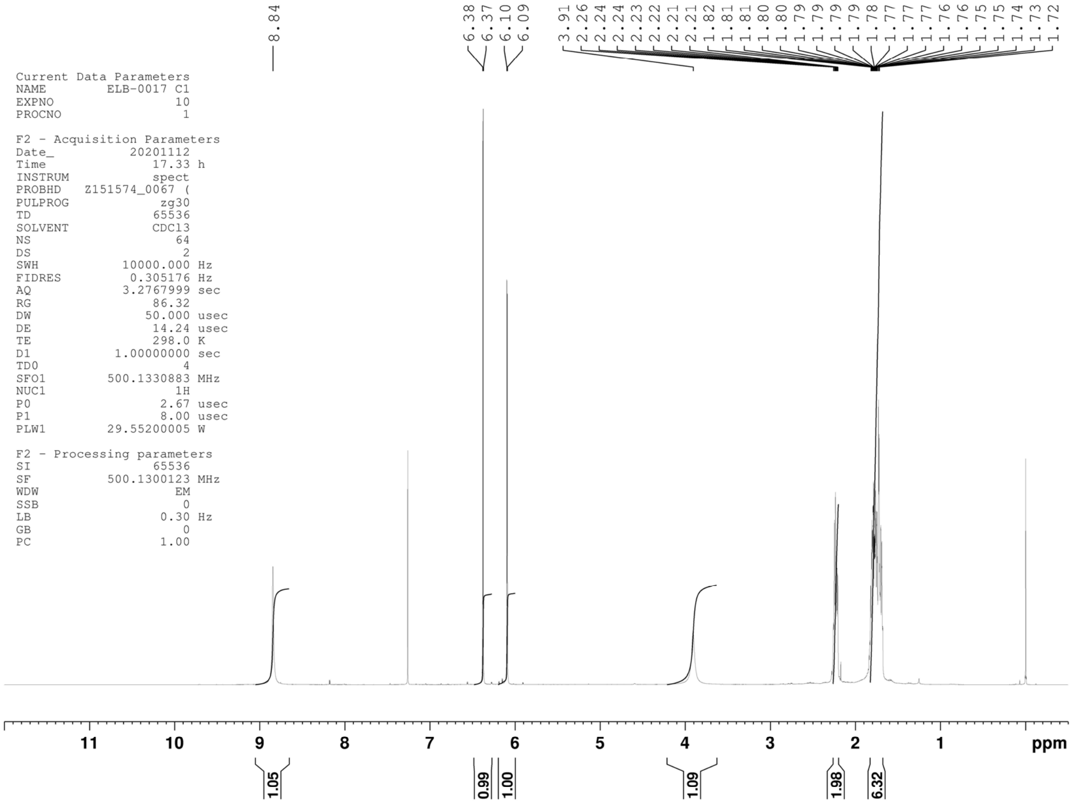

^{13}C-NMR

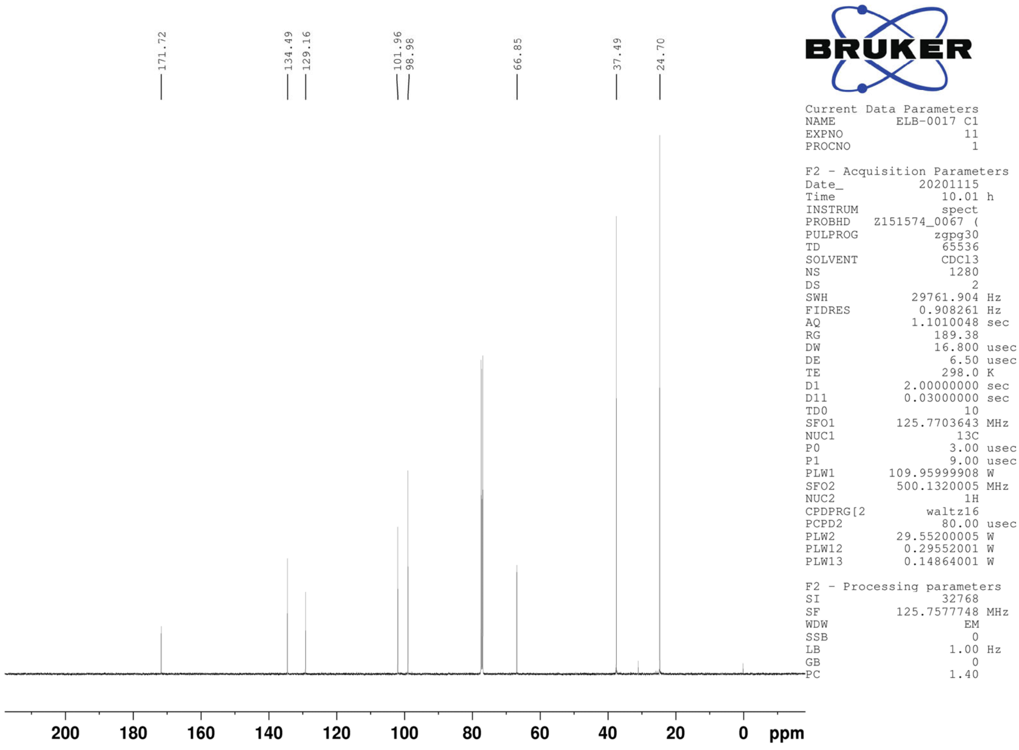

LC

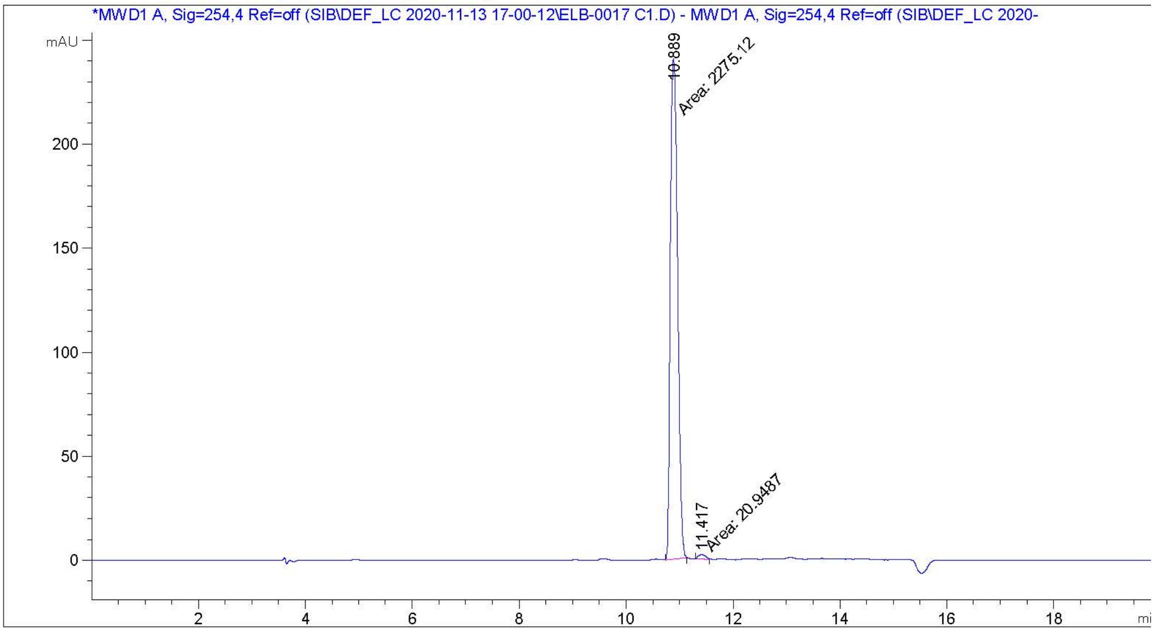


```
=====================================================================
                         Area Percent Report
=====================================================================

Sorted By             :      Signal
Multiplier            :      1.0000
Dilution              :      1.0000
Use Multiplier & Dilution Factor with ISTDs


Signal 1: MWD1 A, Sig=254,4 Ref=off
 Signal has been modified after loading from rawdata file!

Peak RetTime Type  Width     Area      Height     Area
  #   [min]        [min]   [mAU*s]     [mAU]        %
----|-------|----|-------|----------|----------|--------|
   1  10.889 MM    0.1576 2275.11841  240.67653  99.0876
   2  11.417 MM    0.1658   20.94868    2.10626   0.9124

Totals :                  2296.06709  242.78279
```

Compound 36

^{1}H-NMR

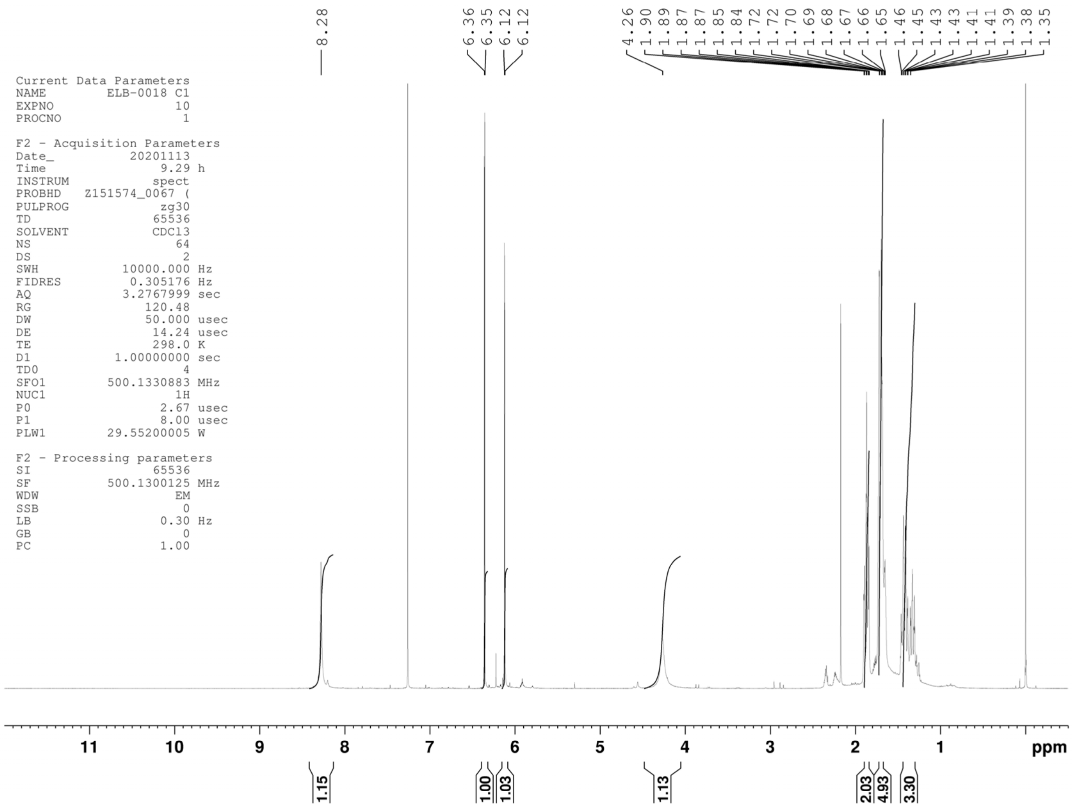

^{13}C-NMR

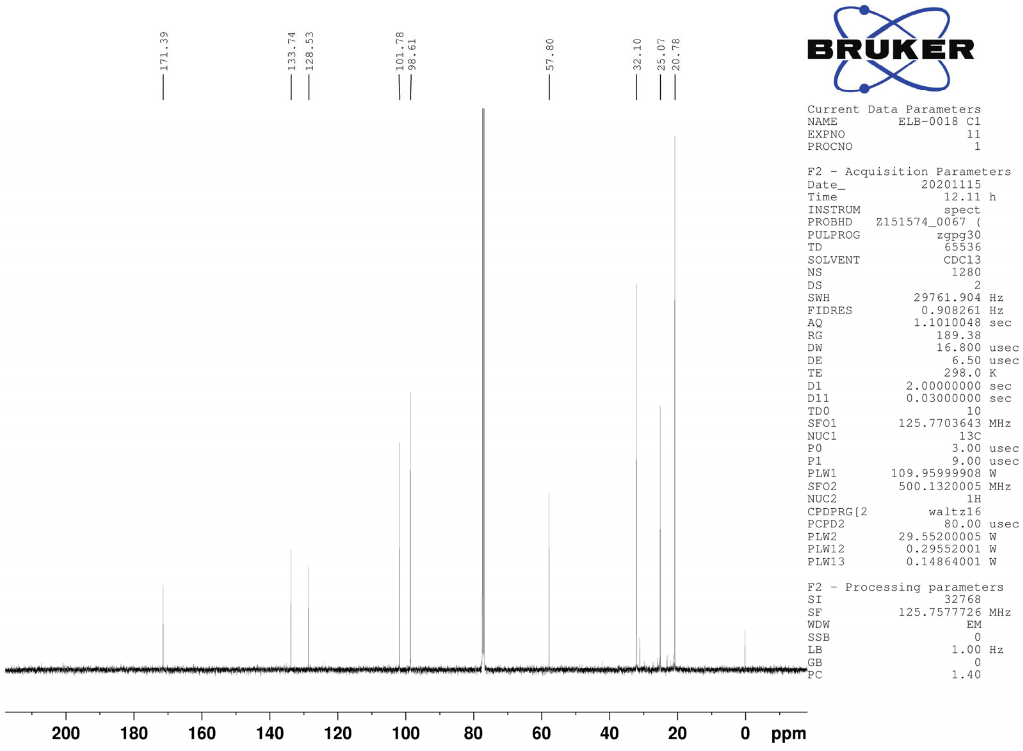

LC

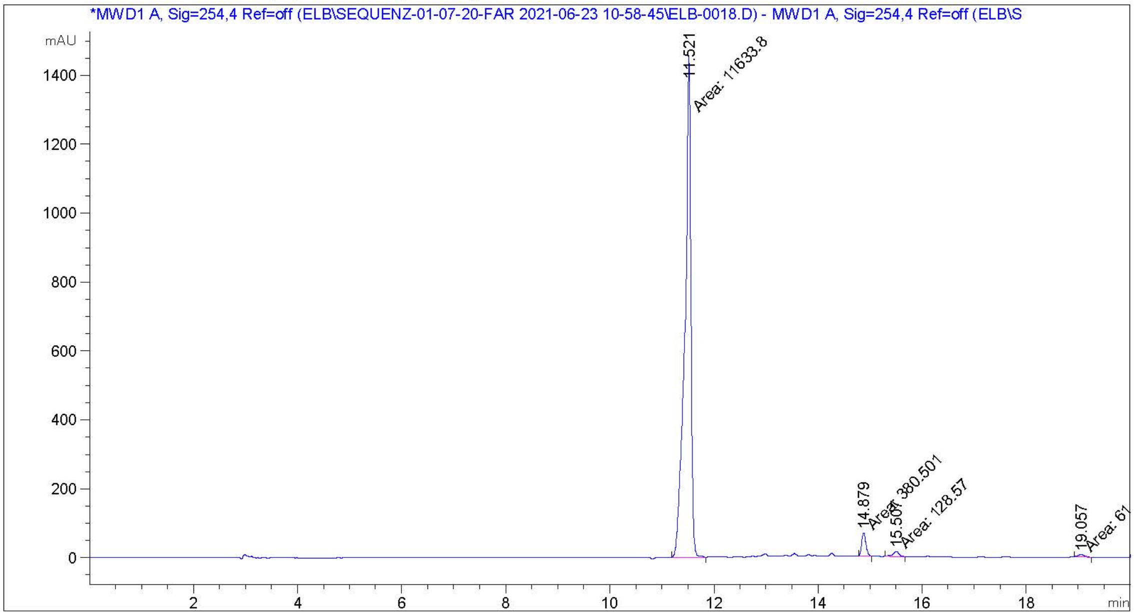


```
=====================================================================
                         Area Percent Report
=====================================================================

Sorted By             :      Signal
Multiplier            :      1.0000
Dilution              :      1.0000
Use Multiplier & Dilution Factor with ISTDs


Signal 1: MWD1 A, Sig=254,4 Ref=off
 Signal has been modified after loading from rawdata file!

Peak RetTime Type  Width     Area      Height     Area
  #   [min]        [min]   [mAU*s]     [mAU]        %
----|-------|----|-------|----------|----------|--------|
   1  11.521 MM    0.1329 1.16338e4 1459.25366 95.3272
   2  14.879 MM    0.0937  380.50146   67.65564  3.1178
   3  15.507 MM    0.1446  128.57019   14.82338  1.0535
   4  19.057 MM    0.1630   61.19766    6.25837  0.5015

Totals :                  1.22041e4 1547.99105
```

Compound 37

^{1}H-NMR

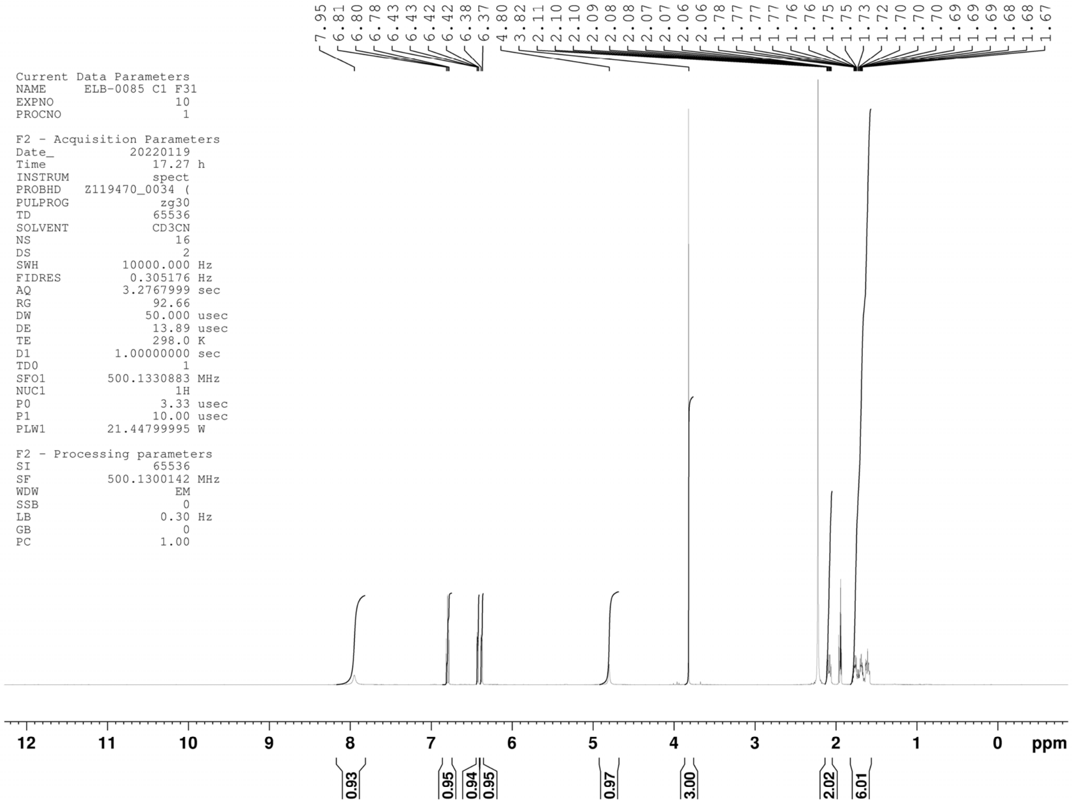

^{13}C-NMR

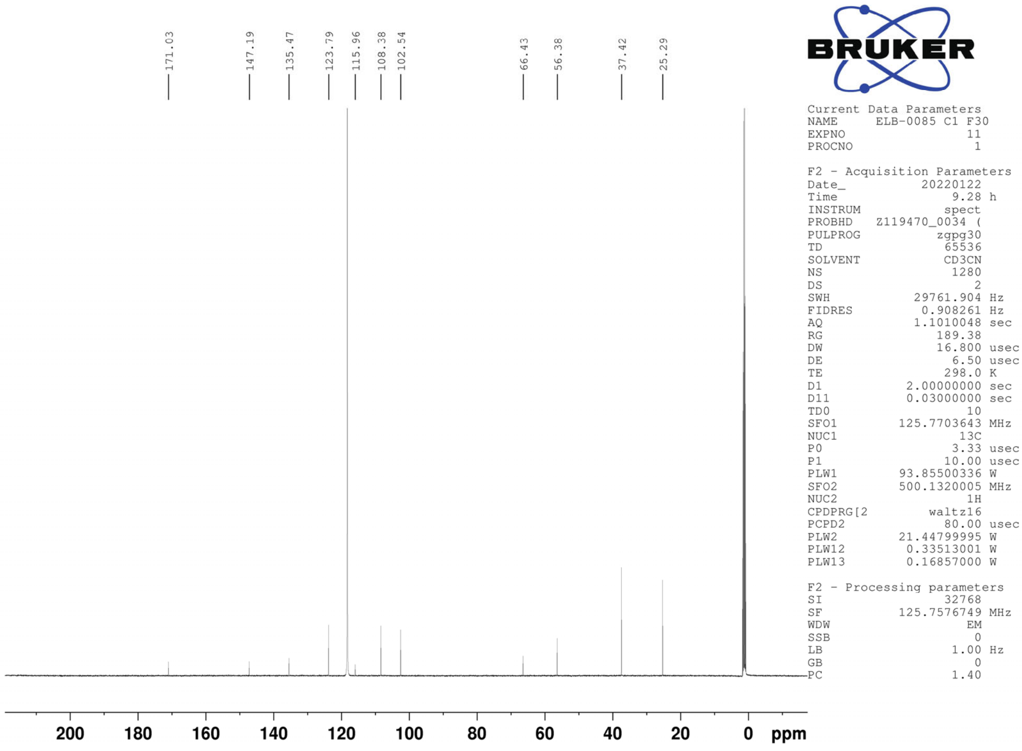

LC

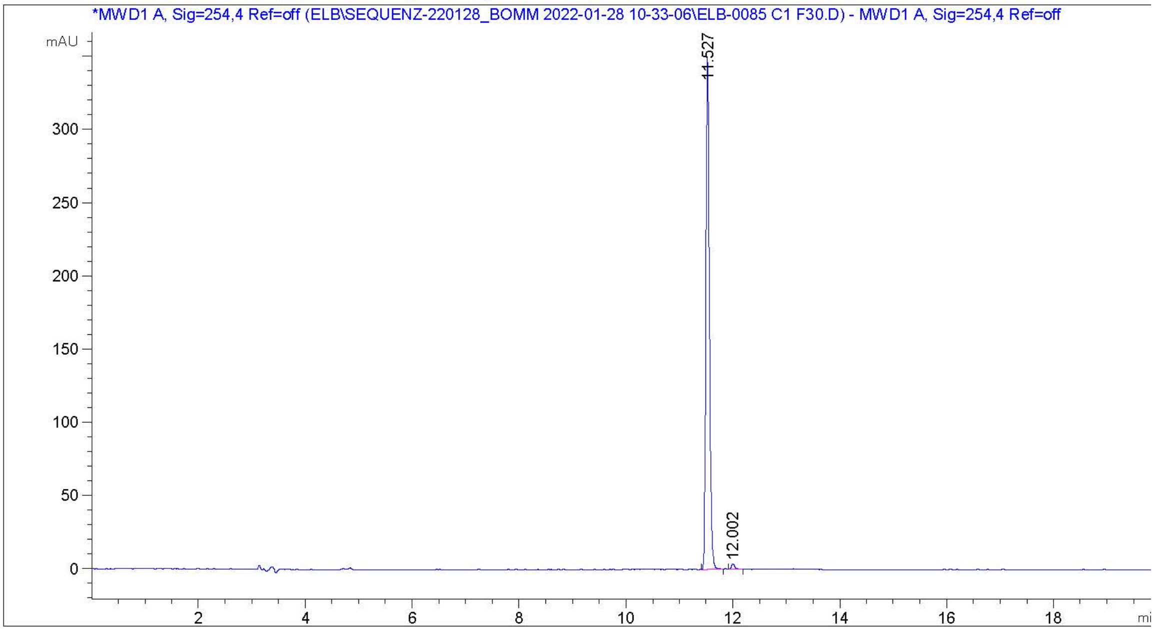


```
=====================================================================
                         Area Percent Report
=====================================================================

Sorted By             :      Signal
Multiplier            :      1.0000
Dilution              :      1.0000
Use Multiplier & Dilution Factor with ISTDs


Signal 1: MWD1 A, Sig=254,4 Ref=off
 Signal has been modified after loading from rawdata file!

Peak RetTime Type  Width     Area      Height     Area
  #   [min]        [min]   [mAU*s]     [mAU]        %
----|-------|----|-------|----------|----------|--------|
   1  11.527 VV    0.0701 1559.25256  349.38745  98.9104
   2  12.002 VB    0.0718   17.17689    3.58976   1.0896

Totals :                  1576.42945  352.97722
```

Compound 38

^{1}H-NMR

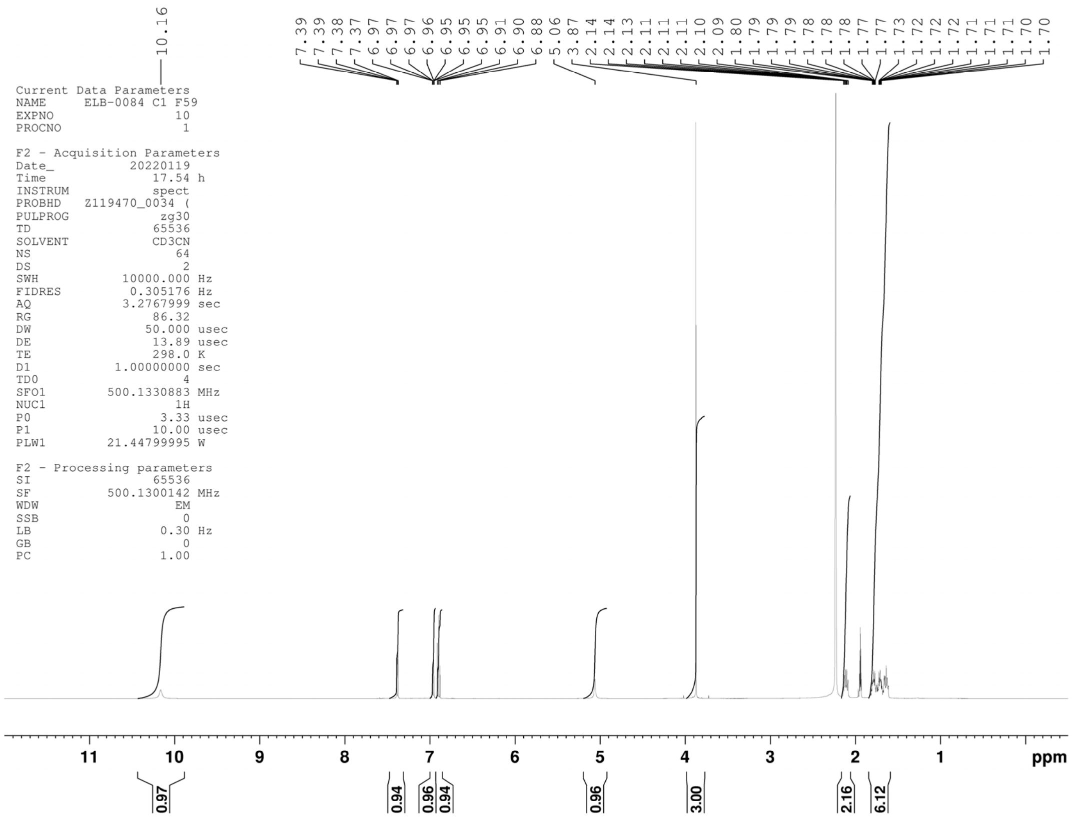

^{13}C-NMR

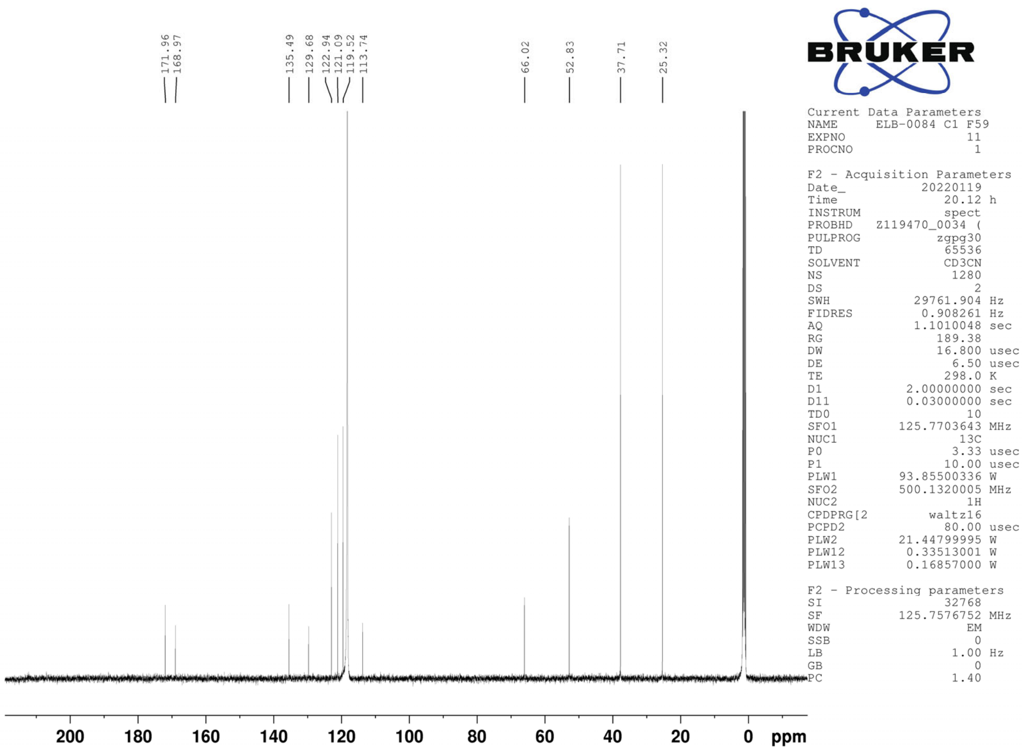

LC

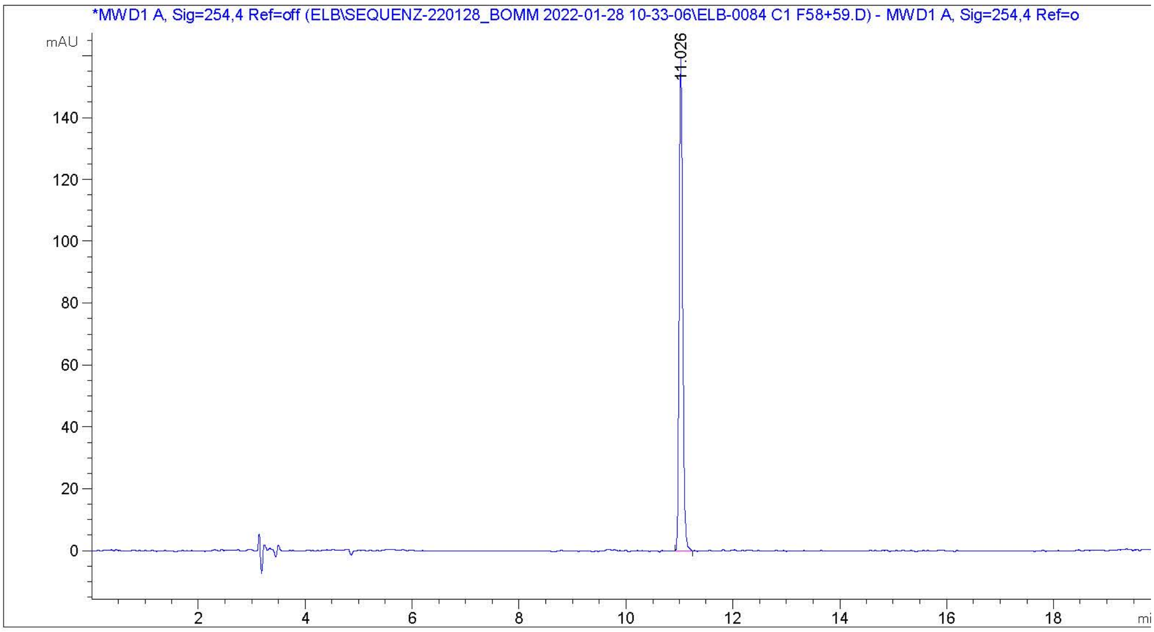


```
=====================================================================
                         Area Percent Report
=====================================================================

Sorted By             :      Signal
Multiplier            :      1.0000
Dilution              :      1.0000
Use Multiplier & Dilution Factor with ISTDs


Signal 1: MWD1 A, Sig=254,4 Ref=off
 Signal has been modified after loading from rawdata file!

Peak RetTime Type  Width     Area      Height     Area
  #    [min]        [min]   [mAU*s]     [mAU]        %
----|-------|----|-------|----------|----------|--------|
   1  11.026 VV    0.0705  717.39008  159.36082 100.0000

Totals :                   717.39008  159.36082
```

Compound 39

^{1}H-NMR

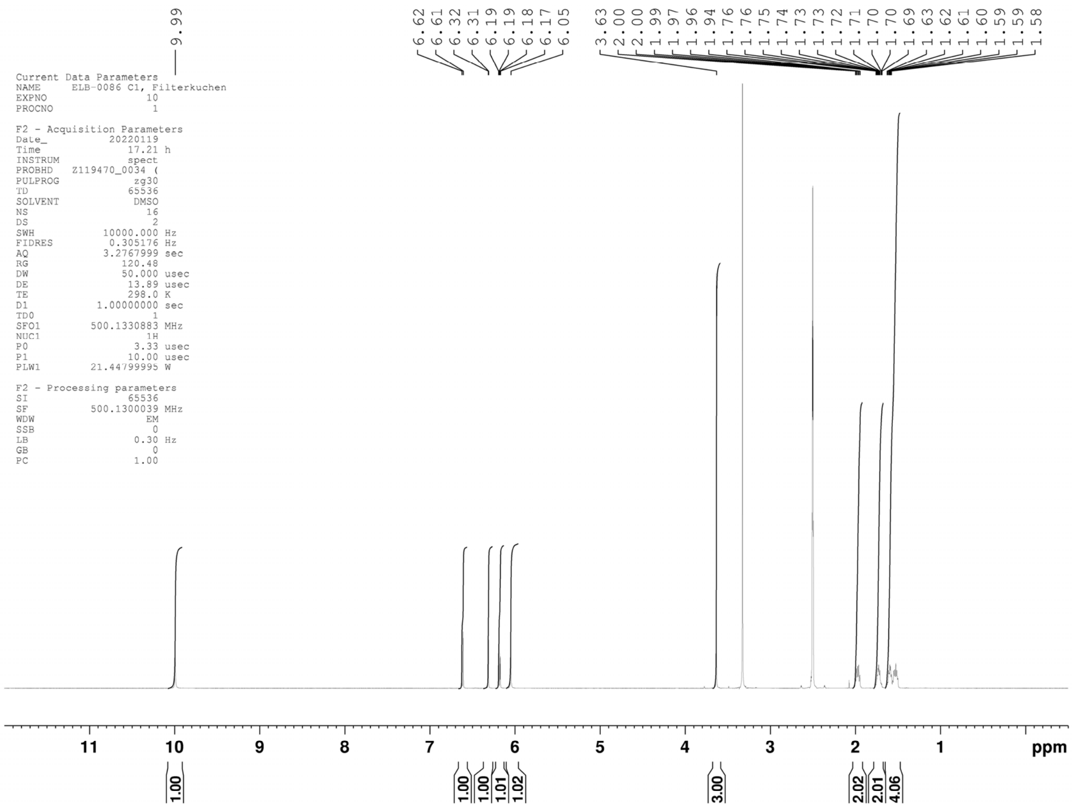

^{13}C-NMR

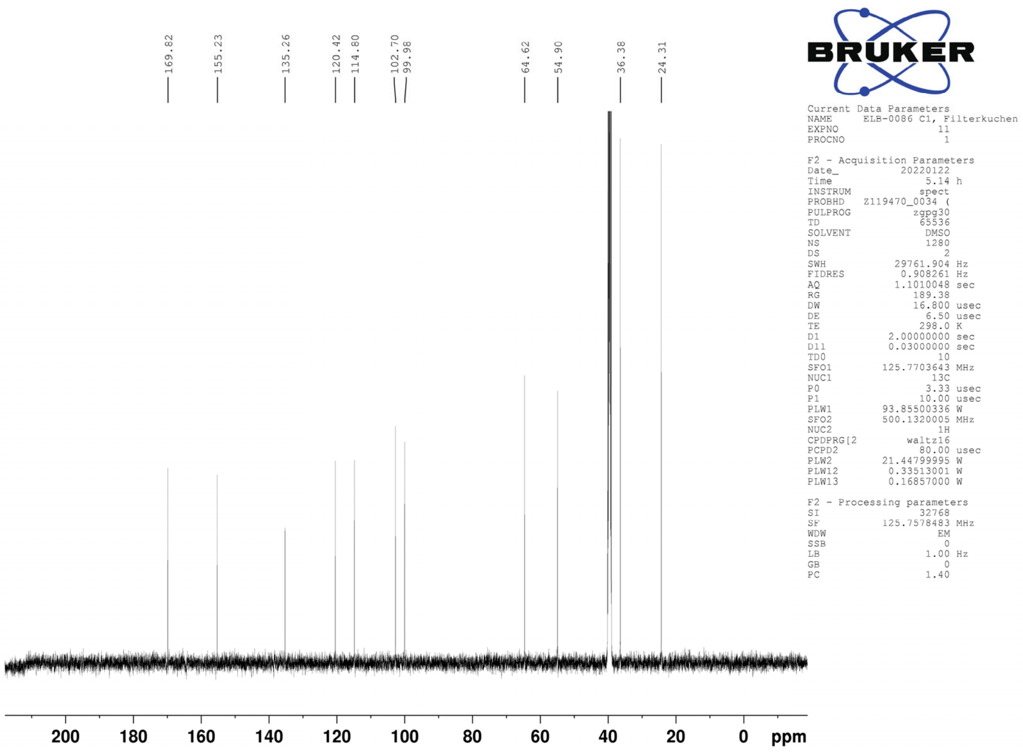

LC

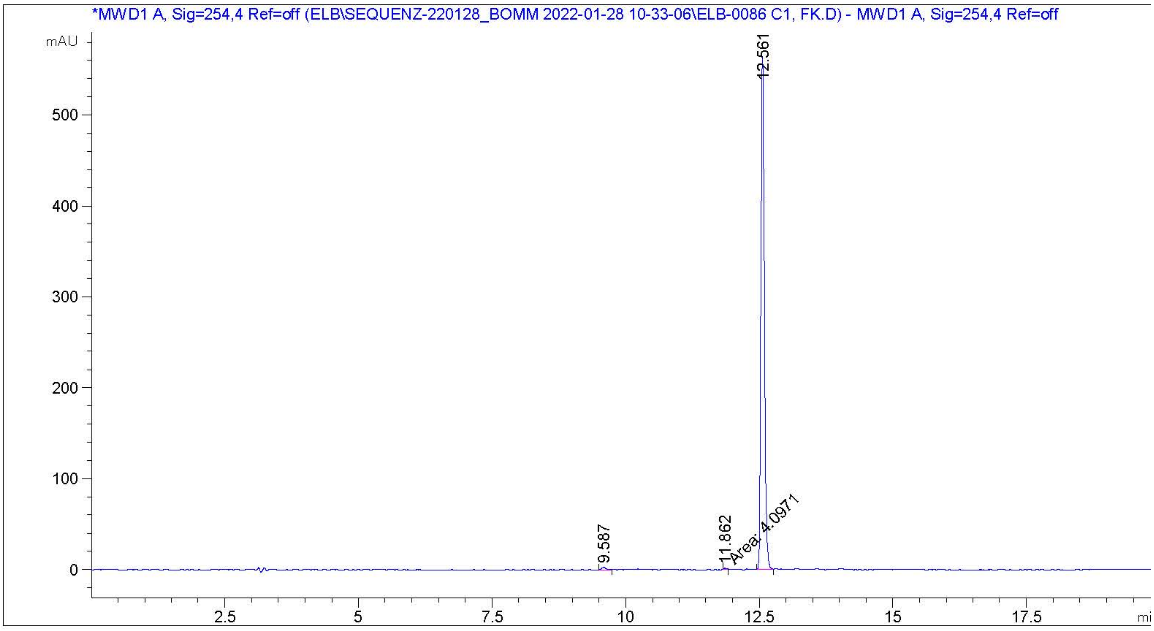


```
=====================================================================
                        Area Percent Report
=====================================================================

Sorted By             :      Signal
Multiplier            :      1.0000
Dilution              :      1.0000
Use Multiplier & Dilution Factor with ISTDs


Signal 1: MWD1 A, Sig=254,4 Ref=off
 Signal has been modified after loading from rawdata file!

Peak RetTime Type  Width     Area      Height     Area
  #   [min]        [min]   [mAU*s]     [mAU]        %
----|-------|----|-------|----------|----------|--------|
   1   9.587 BV    0.0752   12.16766    2.39638   0.4755
   2  11.862 MM    0.0569    4.09710    1.19958   0.1601
   3  12.561 BV    0.0686 2542.89893  564.26318  99.3645

Totals :                  2559.16368  567.85914
```

Compound 40

^{1}H-NMR

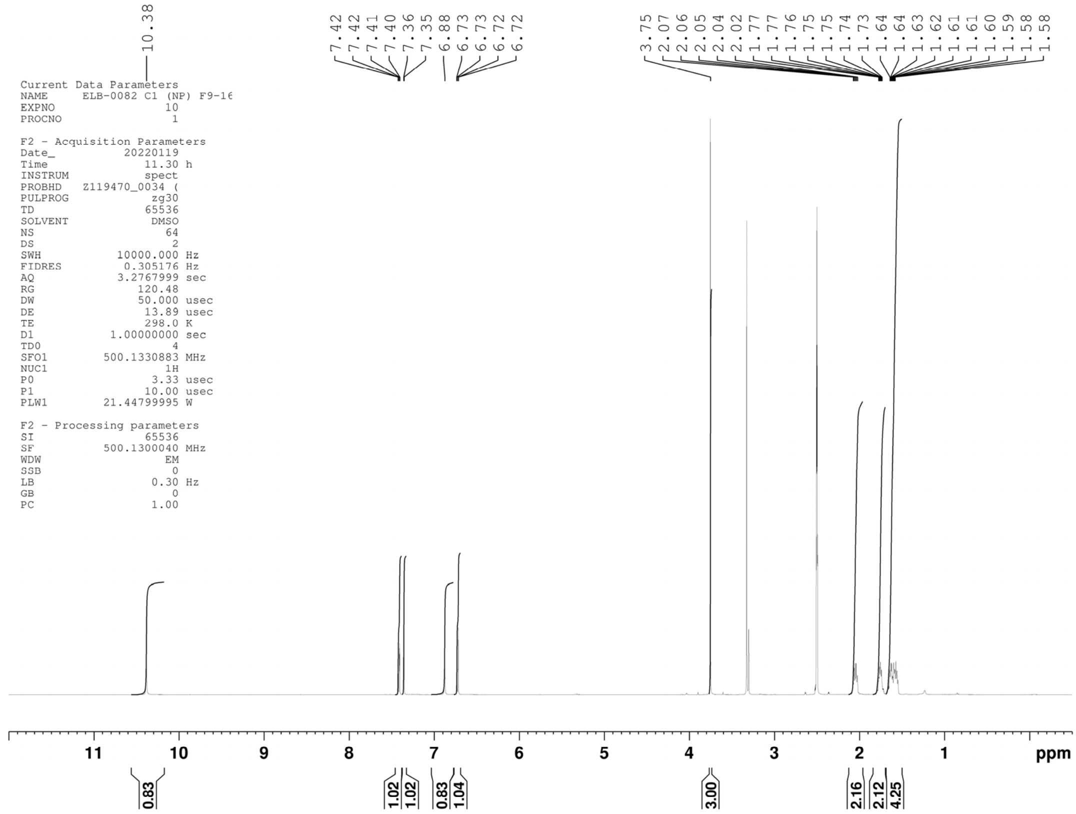

^{13}C-NMR

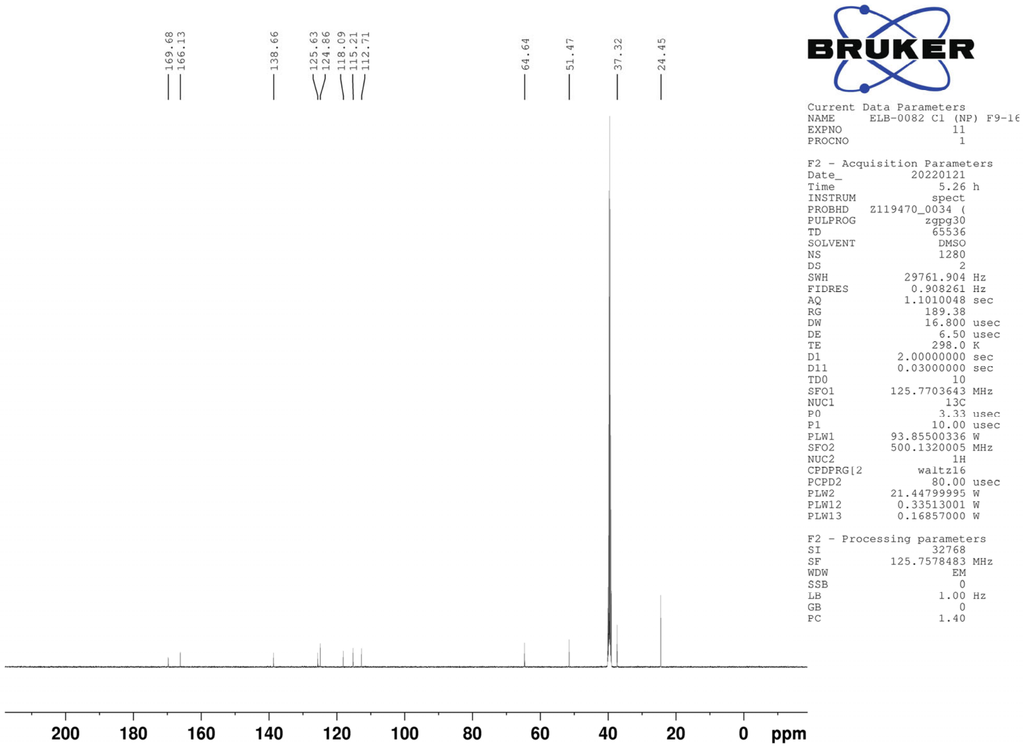

LC

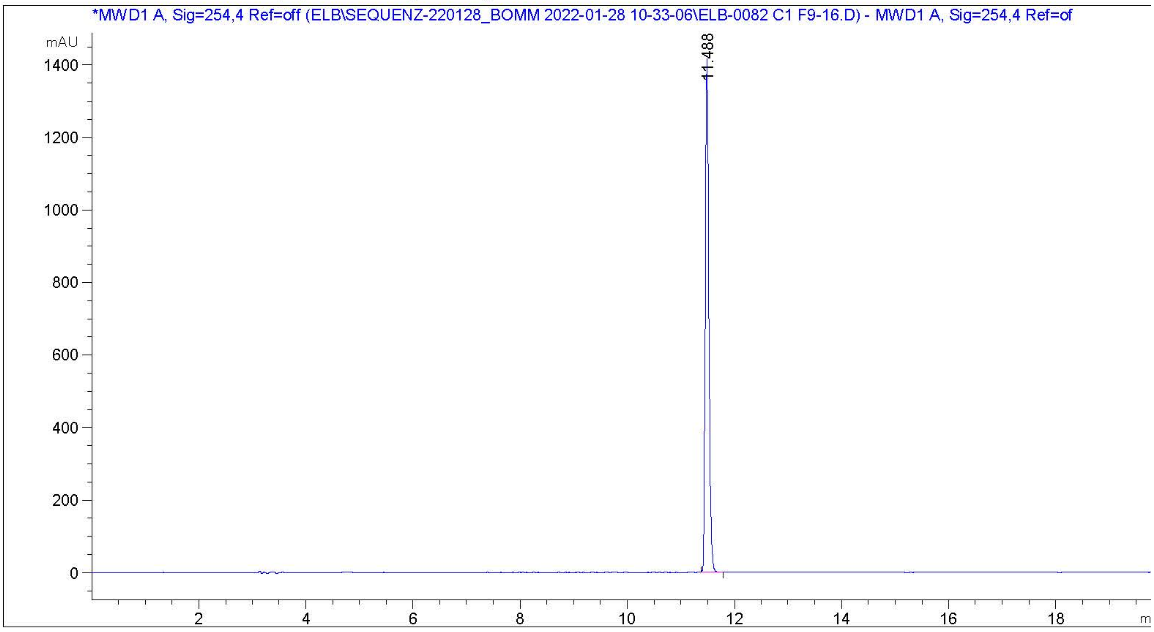


```
=====================================================================
                         Area Percent Report
=====================================================================

Sorted By             :      Signal
Multiplier            :      1.0000
Dilution              :      1.0000
Use Multiplier & Dilution Factor with ISTDs


Signal 1: MWD1 A, Sig=254,4 Ref=off
 Signal has been modified after loading from rawdata file!

Peak RetTime Type  Width     Area      Height     Area
  #   [min]        [min]   [mAU*s]     [mAU]        %
----|-------|----|-------|----------|----------|--------|
   1  11.488 BV    0.0705 6371.96045 1417.23743 100.0000

Totals :                  6371.96045 1417.23743
```

Compound 41

^{1}H-NMR

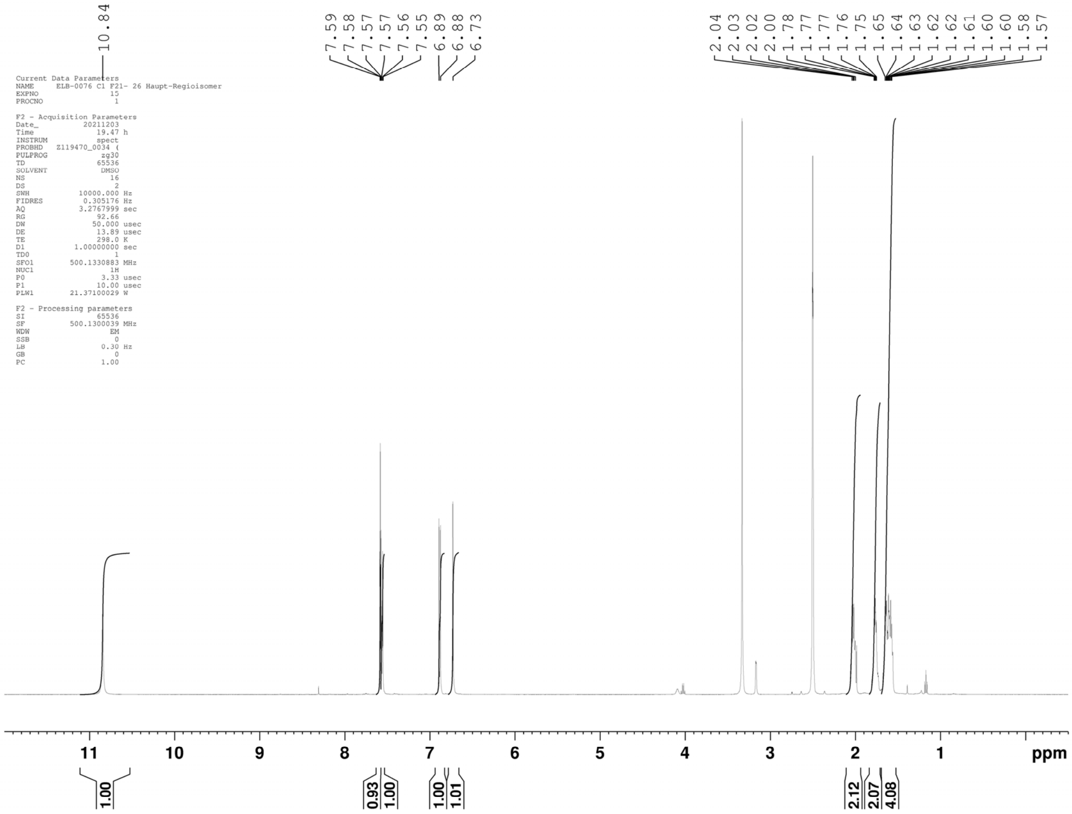

^{13}C-NMR

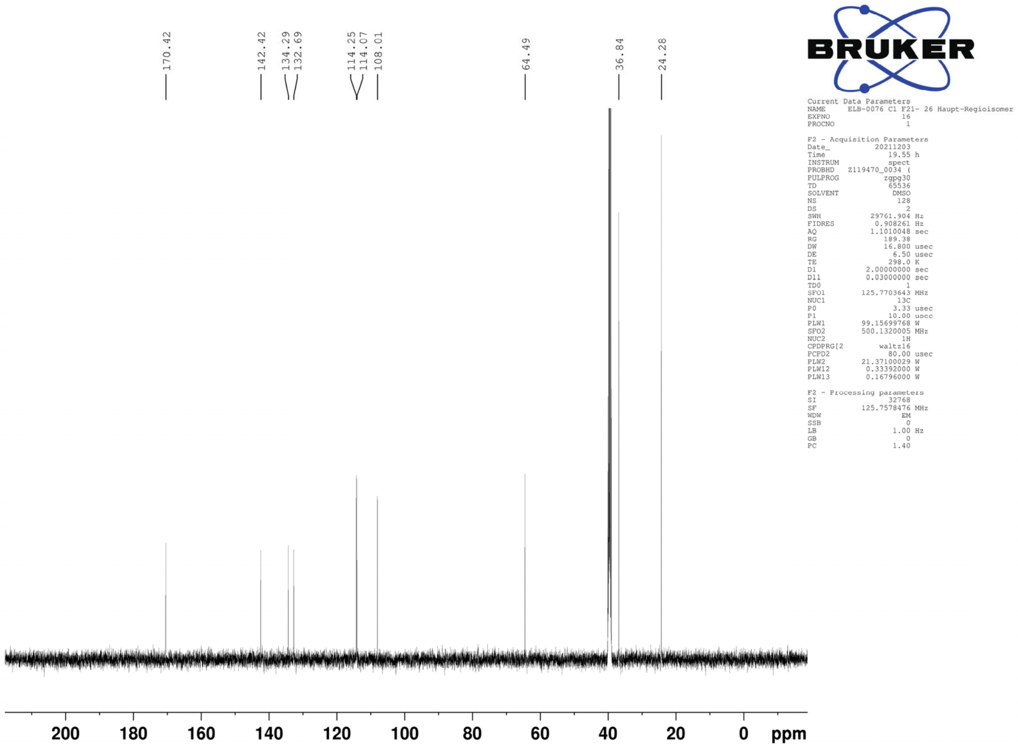

LC

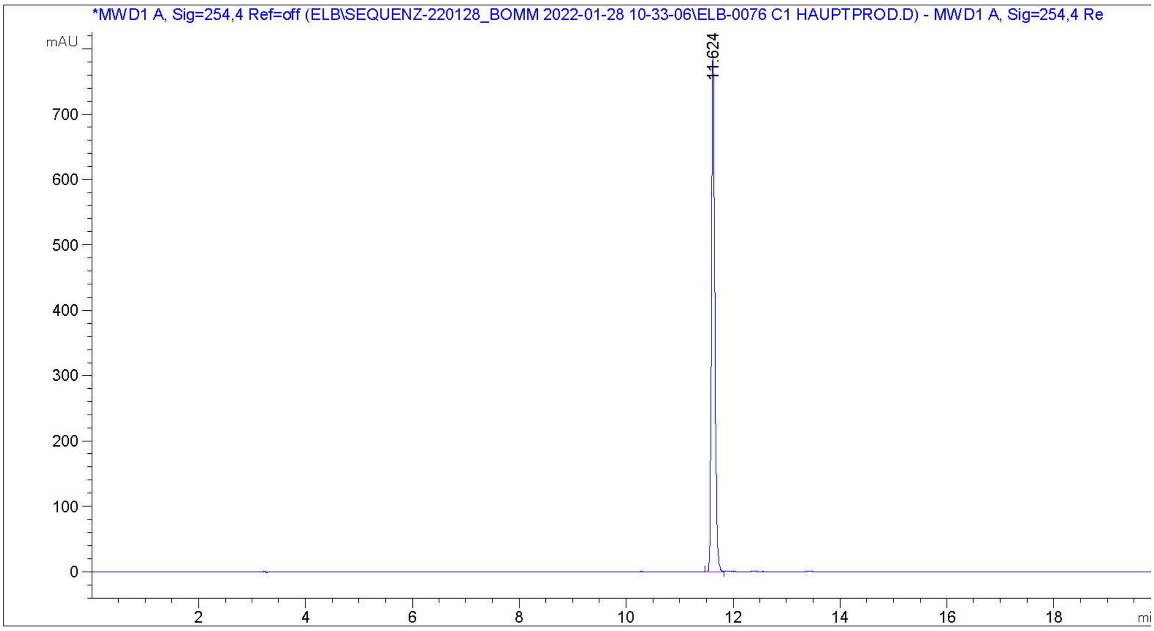


```
=====================================================================
                         Area Percent Report
=====================================================================

Sorted By             :      Signal
Multiplier            :      1.0000
Dilution              :      1.0000
Use Multiplier & Dilution Factor with ISTDs


Signal 1: MWD1 A, Sig=254,4 Ref=off
 Signal has been modified after loading from rawdata file!

Peak RetTime Type  Width     Area      Height     Area
  #   [min]        [min]   [mAU*s]     [mAU]        %
----|-------|----|-------|----------|----------|--------|
   1  11.624 BV    0.0679 3507.93848  788.64679 100.0000

Totals :                  3507.93848  788.64679
```

Compound 42

^{1}H-NMR

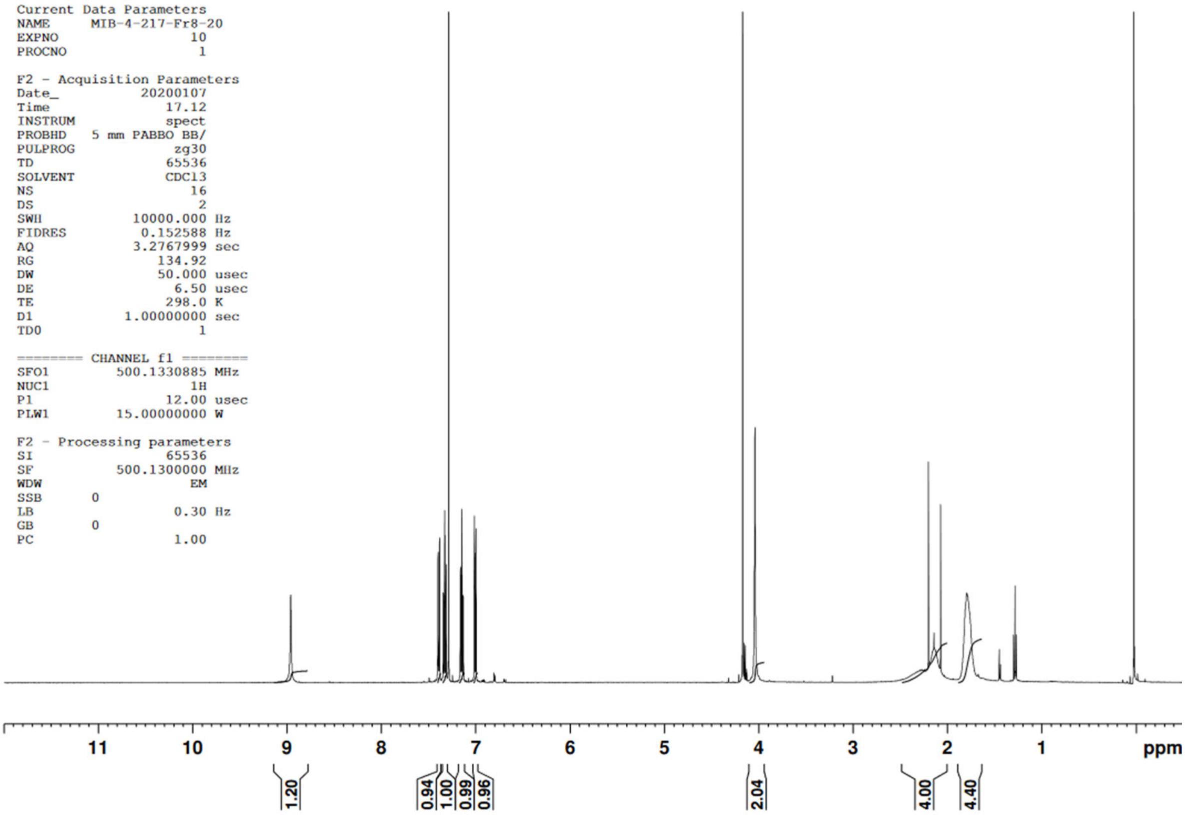

^{13}C-NMR

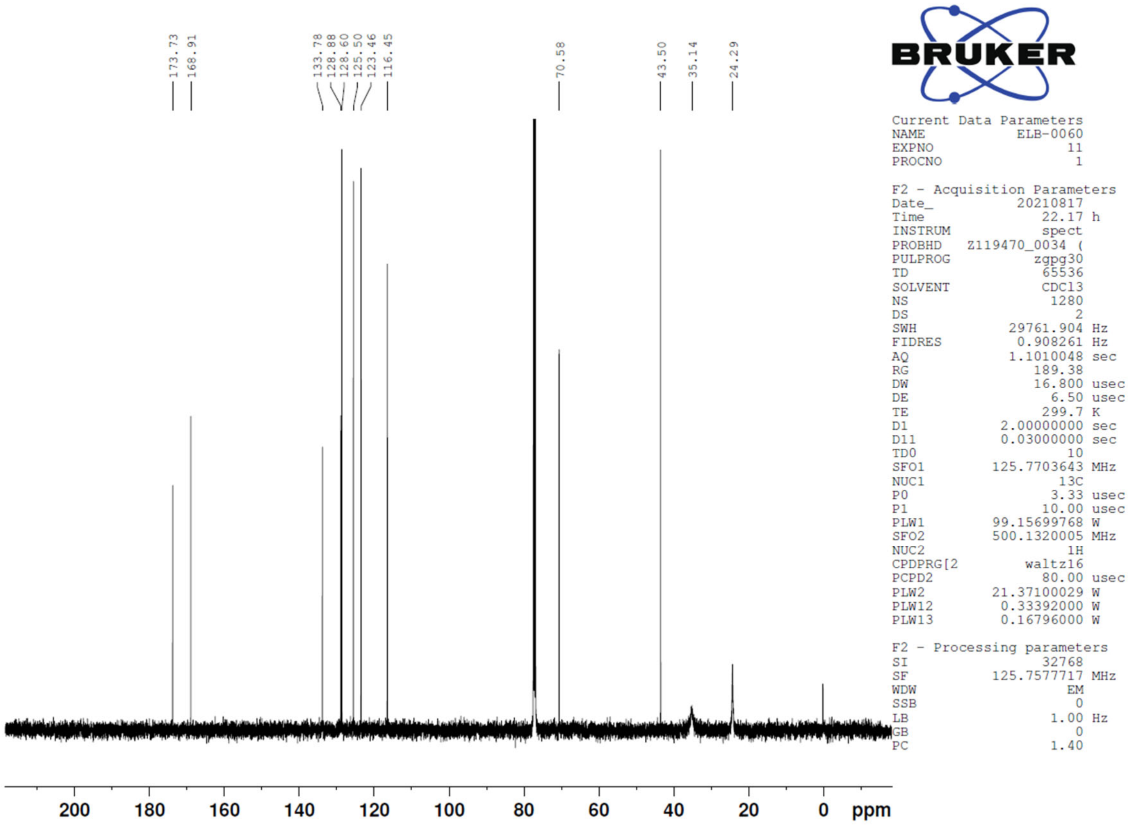

Compound 43

^{1}H-NMR

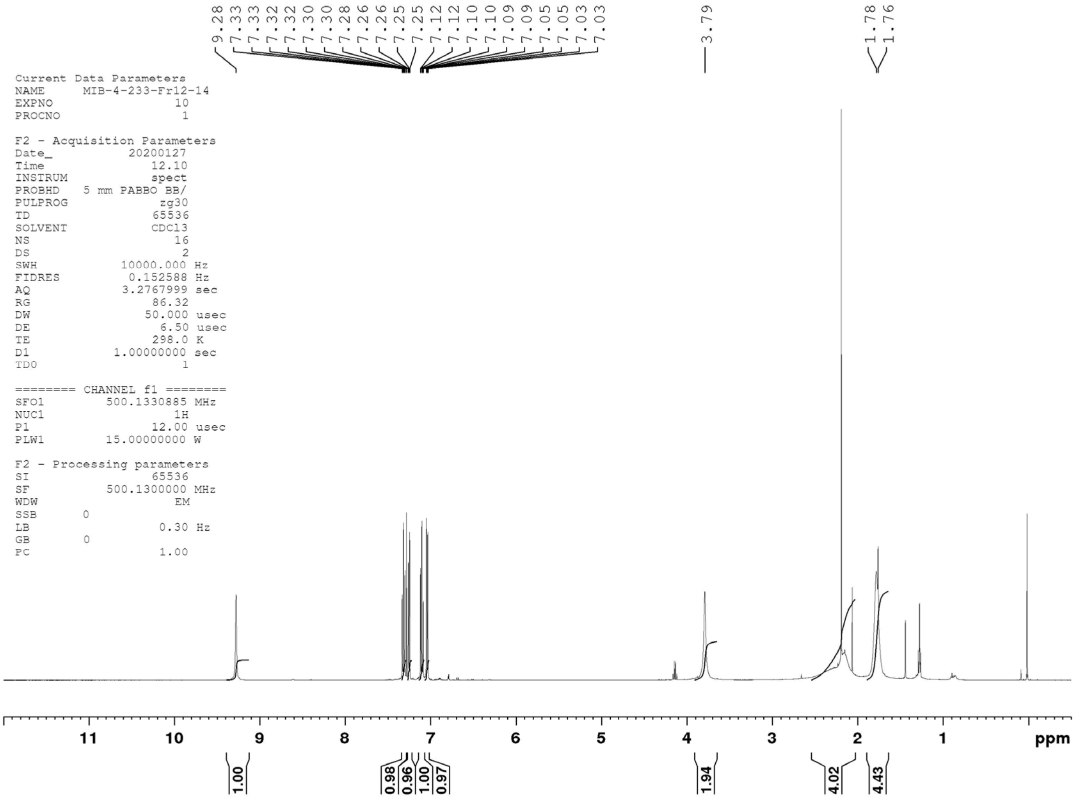

^{13}C-NMR

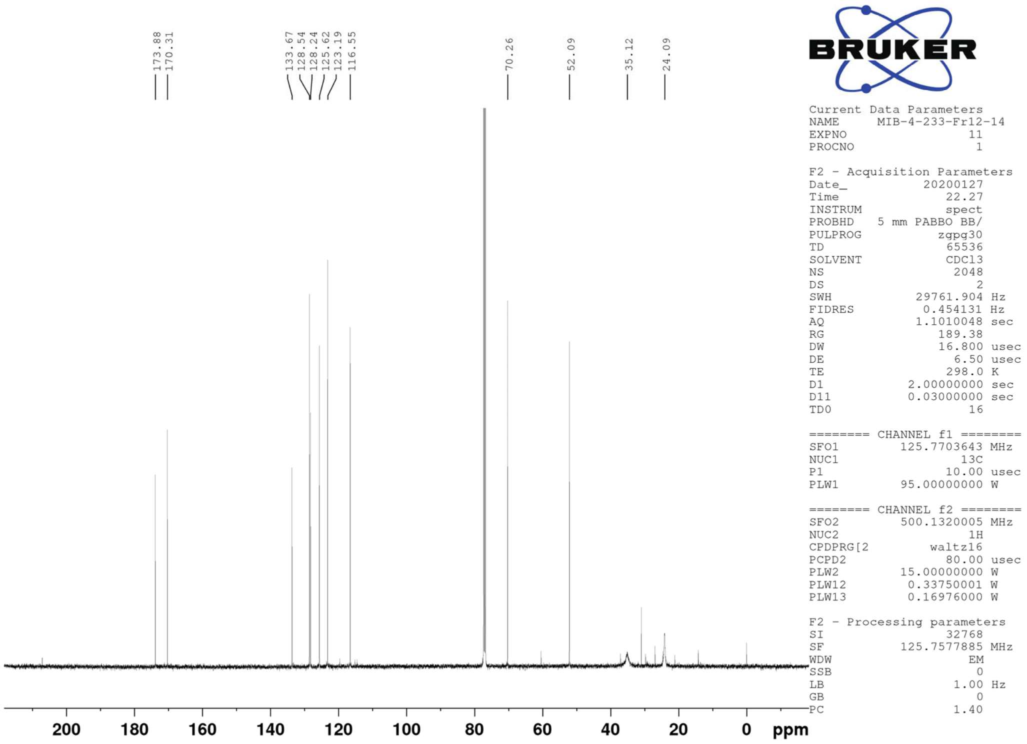

Compound 44

^{1}H-NMR

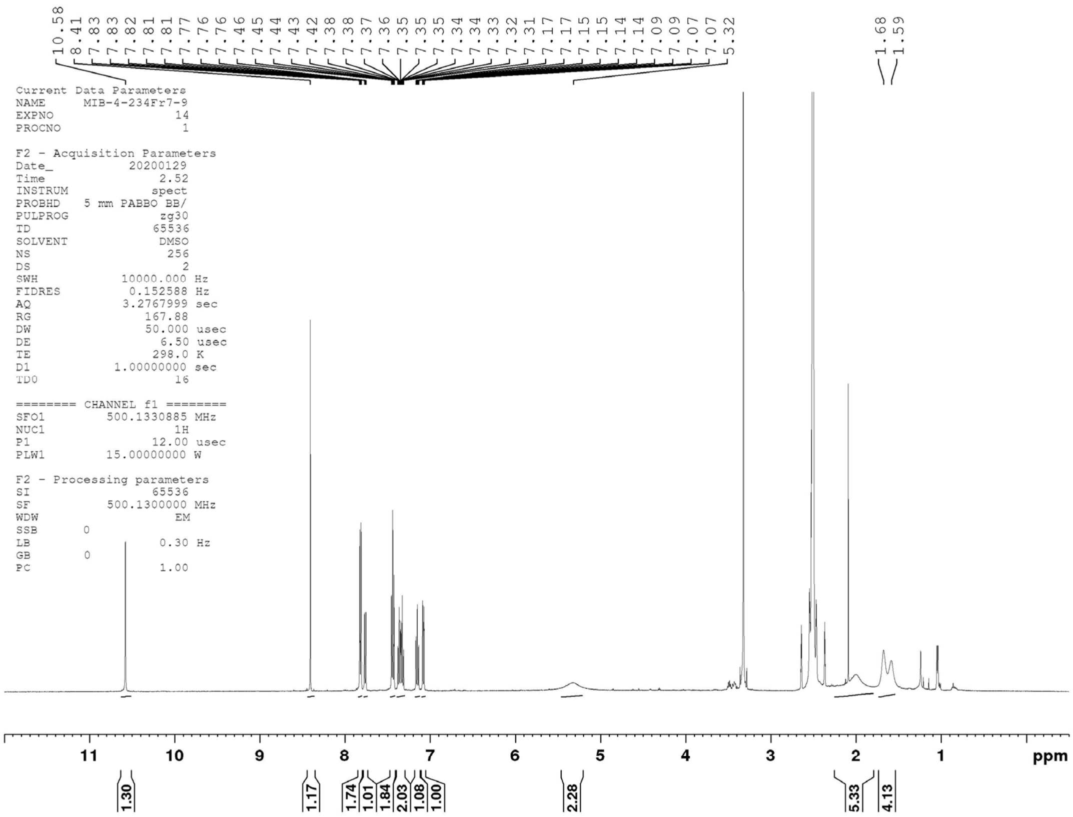

^{13}C-NMR

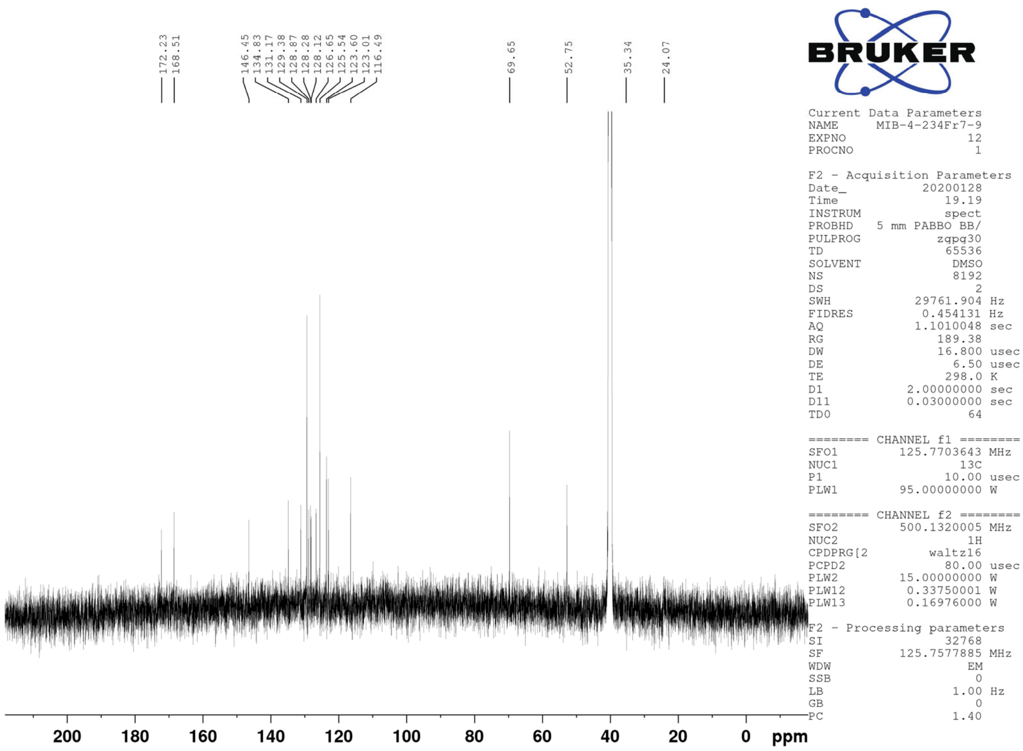

LC

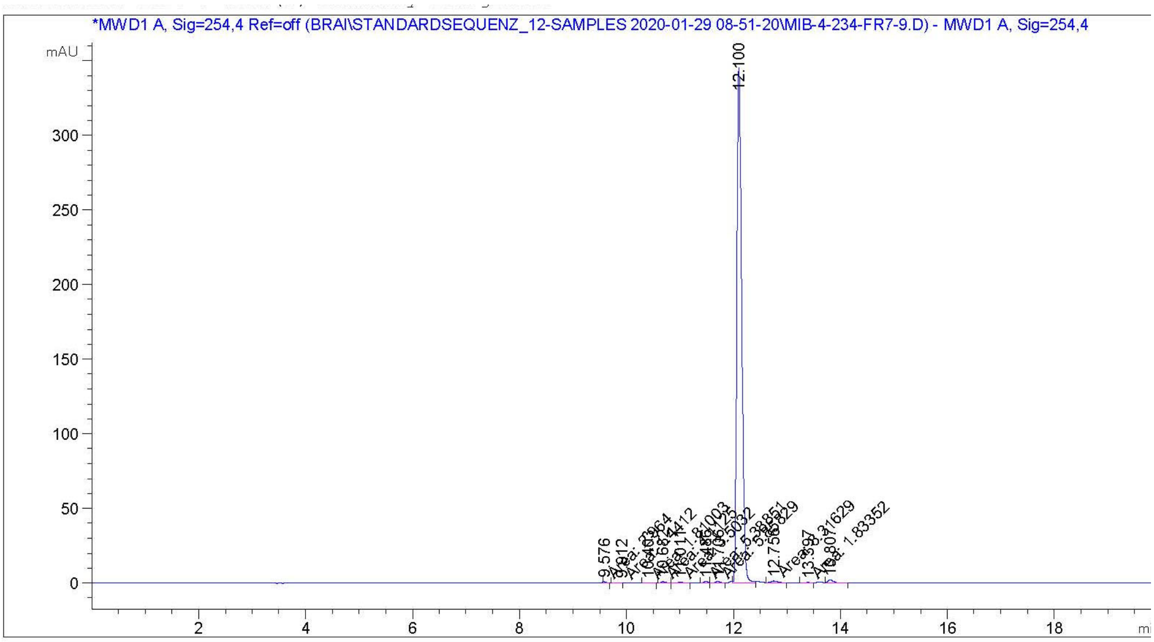


```
=====================================================================
                         Area Percent Report
=====================================================================

Sorted By             :      Signal
Multiplier            :      1.0000
Dilution              :      1.0000
Use Multiplier & Dilution Factor with ISTDs


Signal 1: MWD1 A, Sig=254,4 Ref=off
 Signal has been modified after loading from rawdata file!
```

Peak #	RetTime [min]	Type	Width [min]	Area [mAU*s]	Height [mAU]	Area %
1	9.576	MM	0.0652	2.96400	7.57965e-1	0.1343
2	9.912	MM	0.0793	1.44120	3.03079e-1	0.0653
3	10.403	MM	0.1256	1.81003	2.40131e-1	0.0820
4	10.682	MM	0.1006	4.11250	6.81531e-1	0.1863
5	11.011	MM	0.1182	3.50320	4.94089e-1	0.1587
6	11.486	MM	0.0972	5.38851	9.23937e-1	0.2441
7	11.706	MM	0.1071	5.85829	9.11943e-1	0.2654
8	12.100	VV	0.0970	2157.63428	344.96829	97.7415
9	12.756	MM	0.1277	8.31629	1.08515	0.3767
10	13.397	MM	0.1309	1.83352	2.33445e-1	0.0831
11	13.807	VV	0.1230	14.62966	1.83569	0.6627

Compound 45

^{1}H-NMR

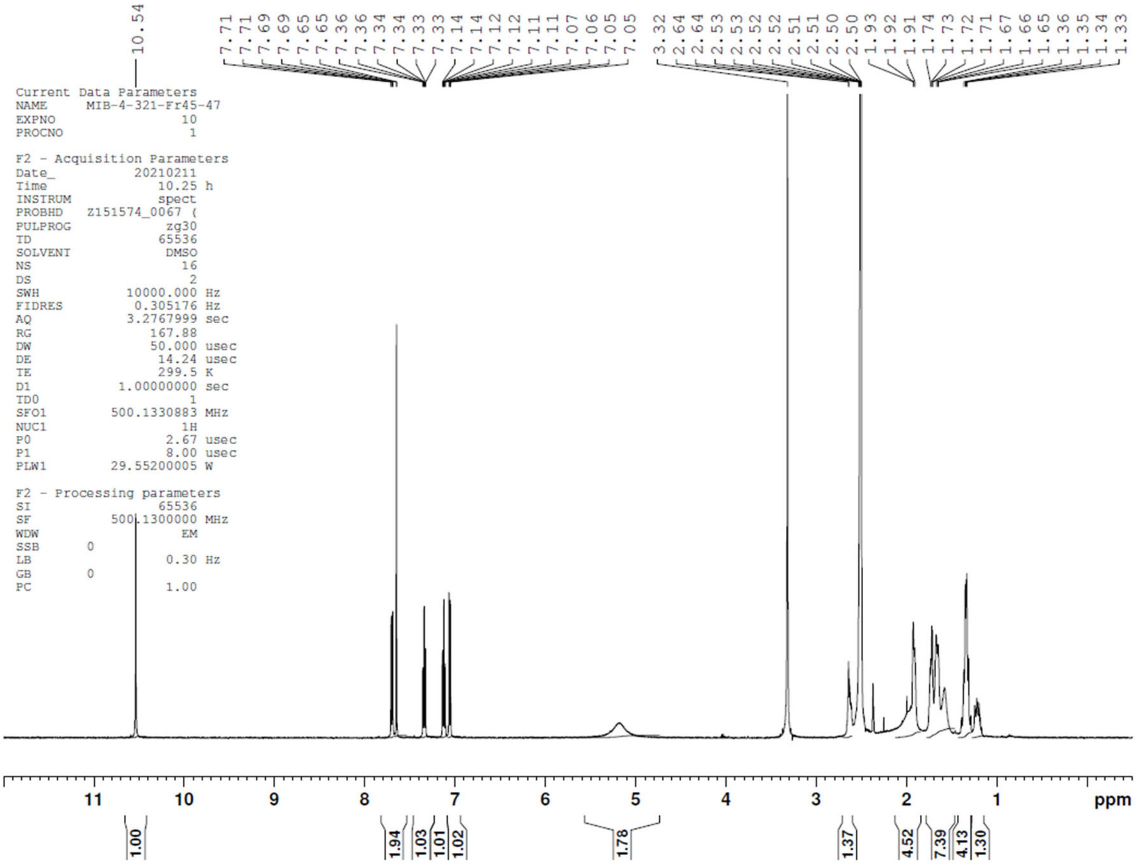

^{13}C-NMR

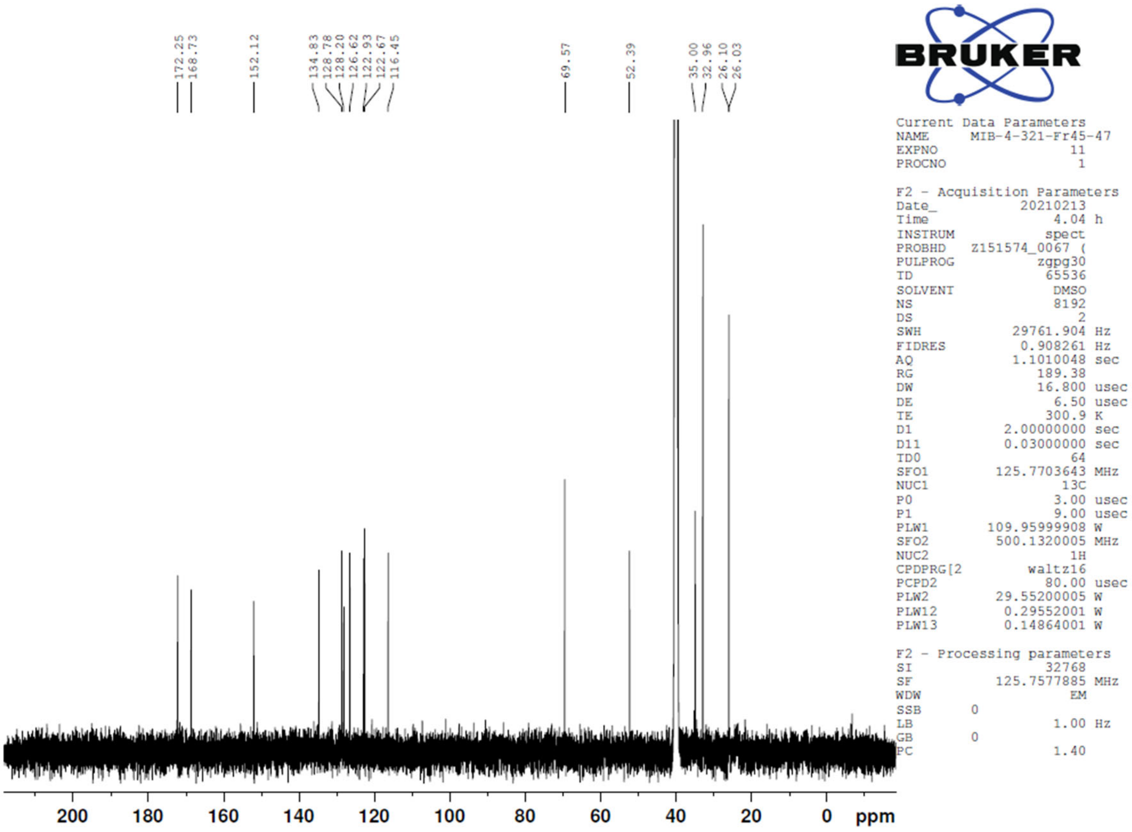

LC

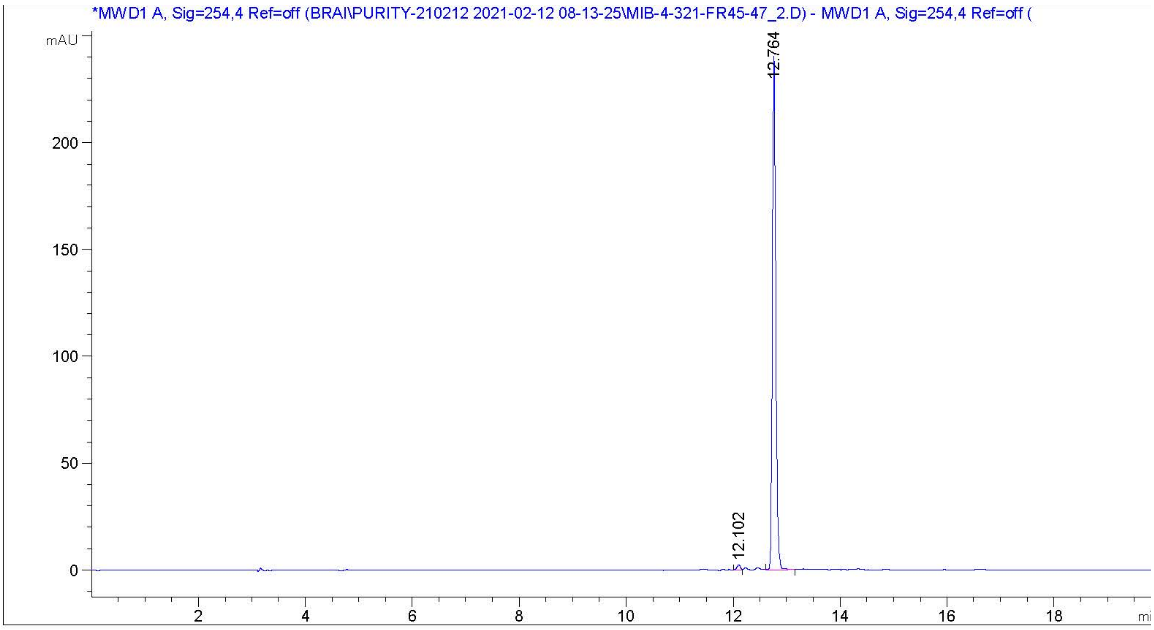


```
=====================================================================
                         Area Percent Report
=====================================================================

Sorted By             :      Signal
Multiplier            :      1.0000
Dilution              :      1.0000
Use Multiplier & Dilution Factor with ISTDs


Signal 1: MWD1 A, Sig=254,4 Ref=off
 Signal has been modified after loading from rawdata file!

Peak RetTime Type  Width     Area      Height     Area
  #    [min]        [min]   [mAU*s]     [mAU]        %
----|-------|----|-------|----------|----------|--------|
   1  12.102 VV     0.0742   12.02480    2.49525    1.1109
   2  12.764 VB     0.0679 1070.39270  240.64124   98.8891
```

Compound 46

^{1}H-NMR

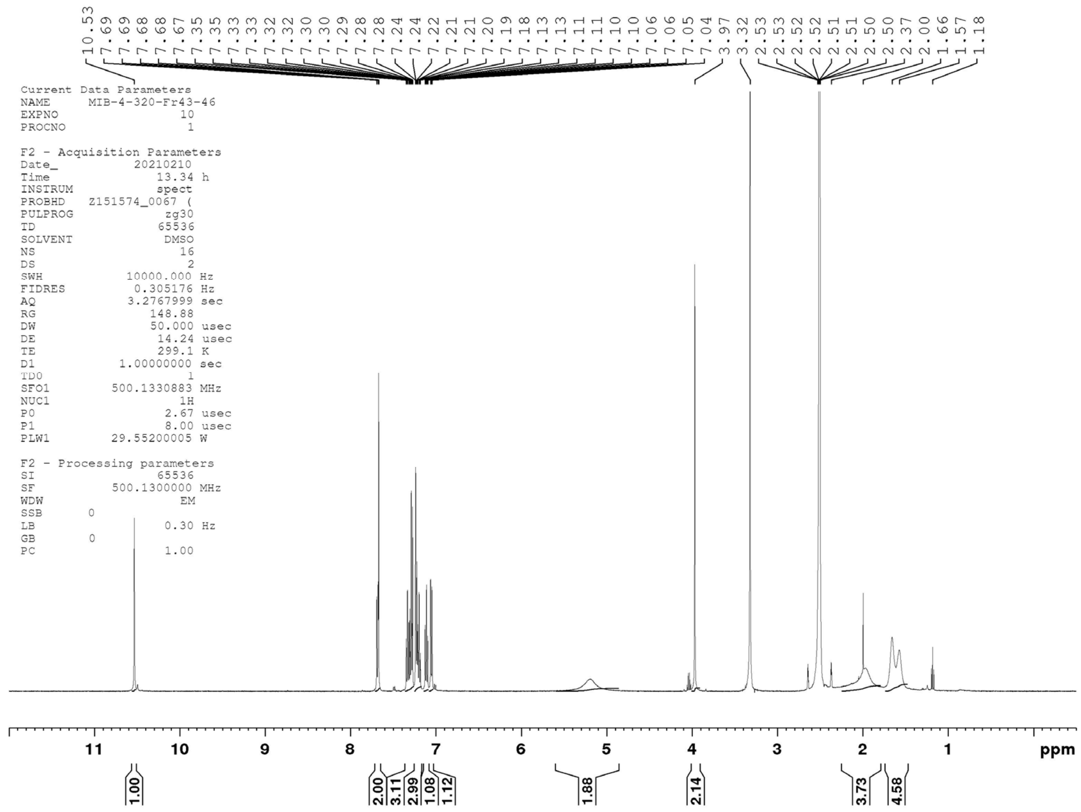

^{13}C-NMR

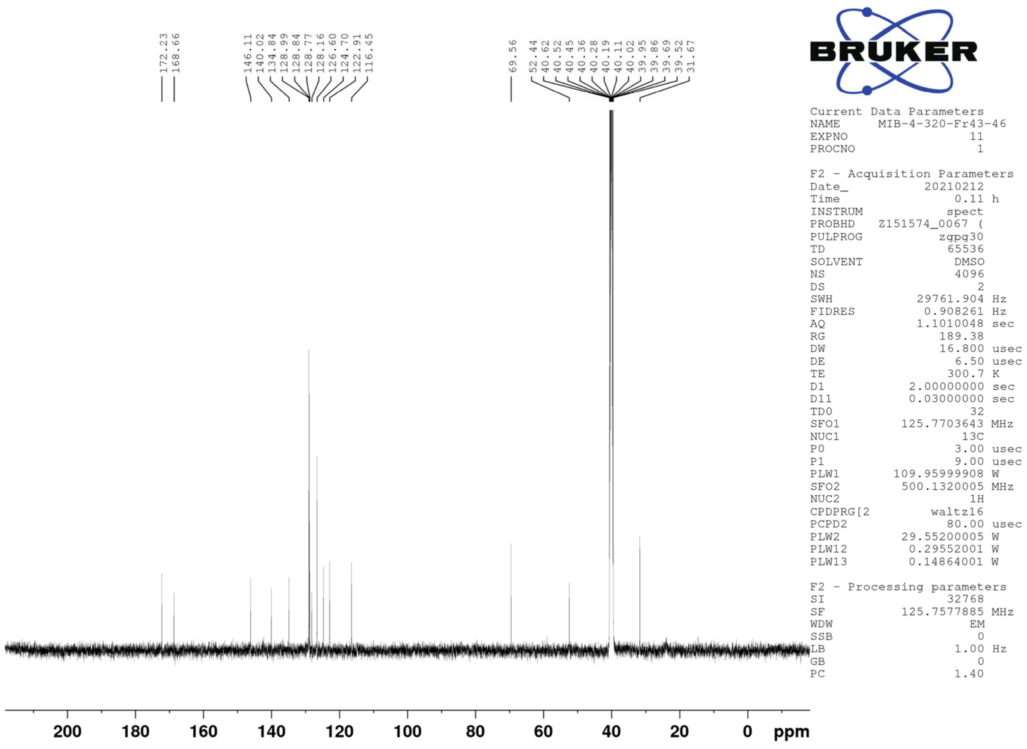

LC

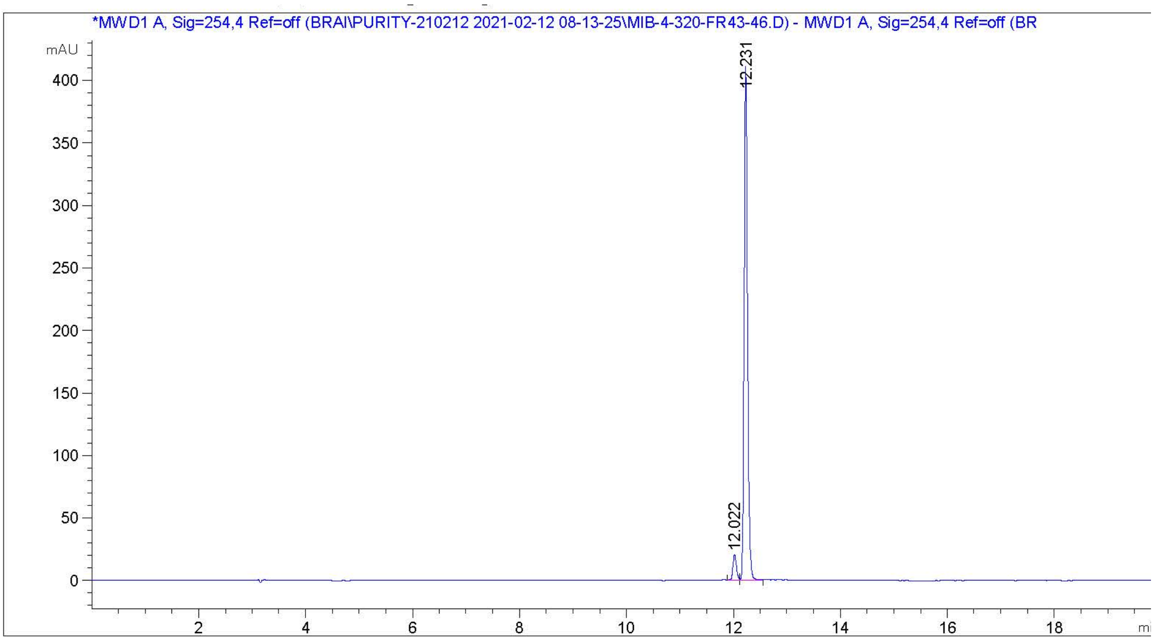


```
=====================================================================
                         Area Percent Report
=====================================================================

Sorted By             :      Signal
Multiplier            :      1.0000
Dilution              :      1.0000
Use Multiplier & Dilution Factor with ISTDs


Signal 1: MWD1 A, Sig=254,4 Ref=off
 Signal has been modified after loading from rawdata file!

Peak RetTime Type  Width     Area      Height     Area
  #   [min]        [min]   [mAU*s]     [mAU]        %
----|-------|----|-------|----------|----------|--------|
   1  12.022 BV    0.0683   91.06959   20.31276    4.7026
   2  12.231 VV    0.0681 1845.51868  413.59122   95.2974
```

Compound 47

^{1}H-NMR

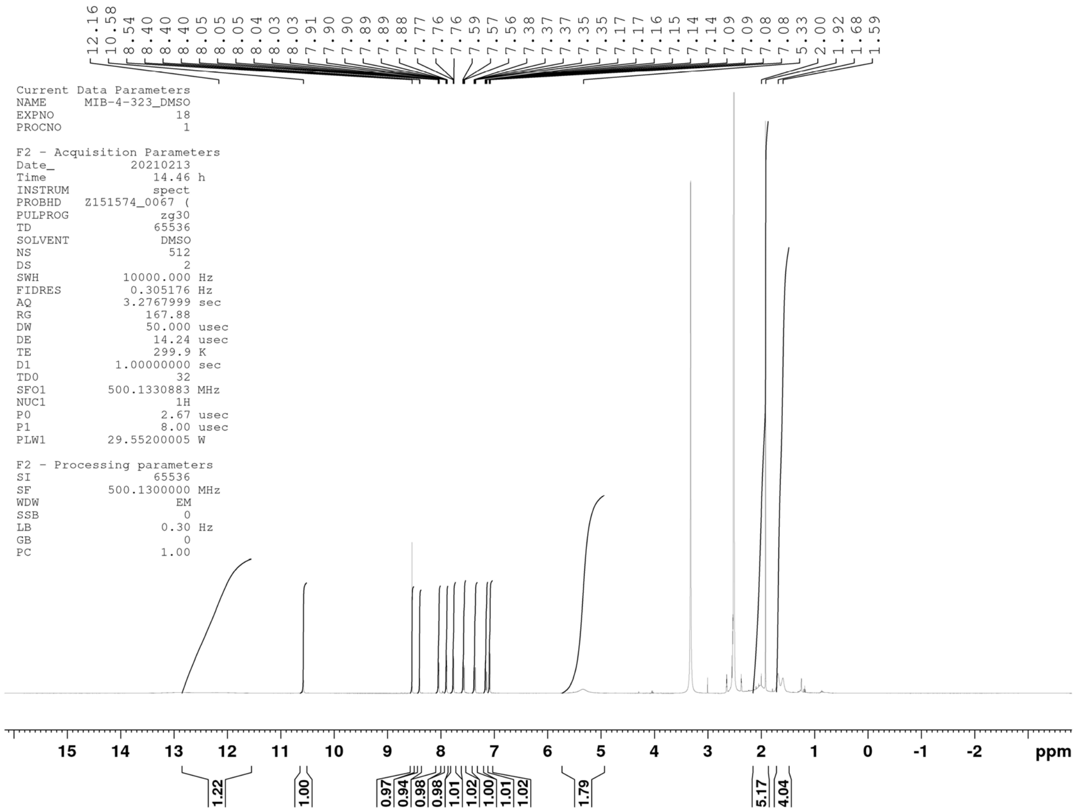

^{13}C-NMR

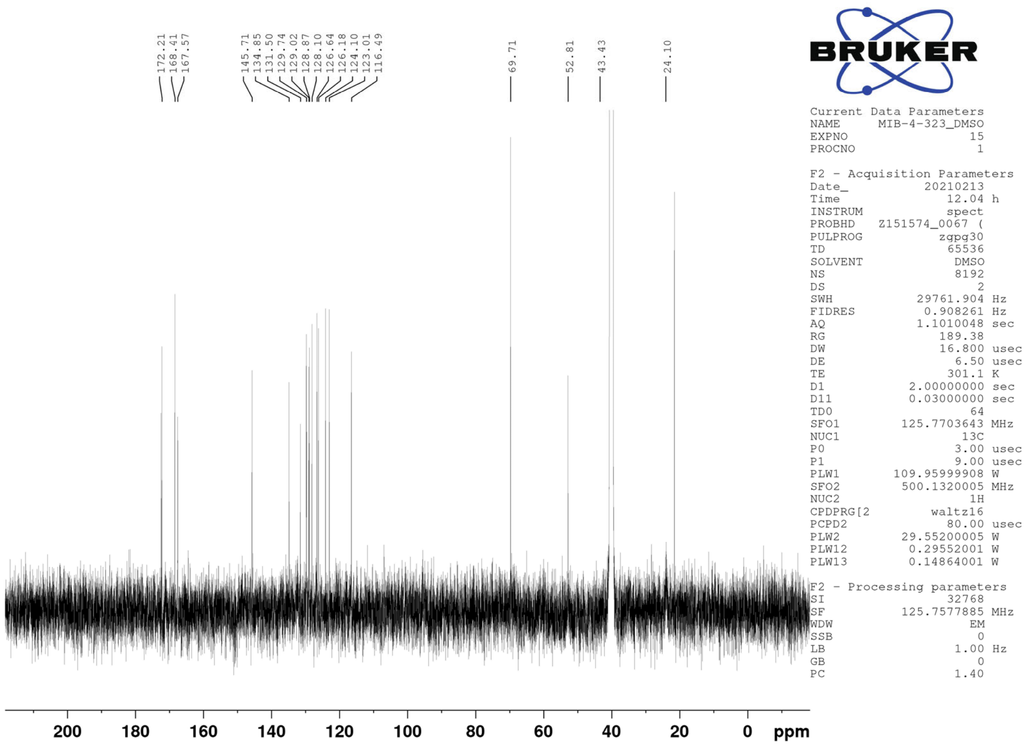

LC

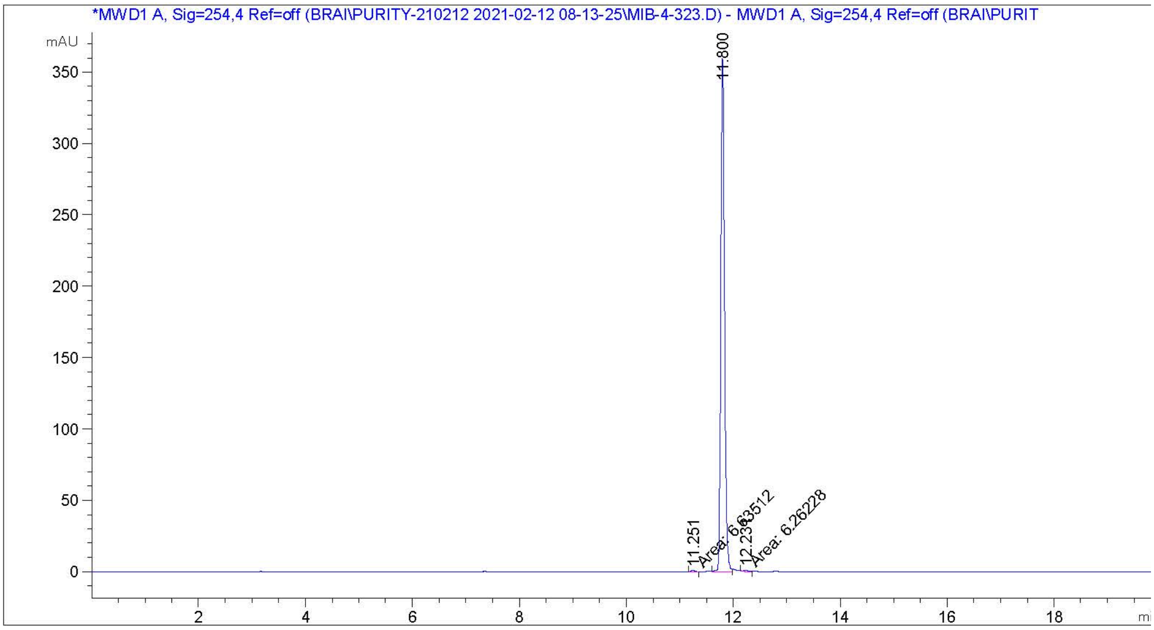


```
=====================================================================
                         Area Percent Report
=====================================================================

Sorted By             :      Signal
Multiplier            :      1.0000
Dilution              :      1.0000
Use Multiplier & Dilution Factor with ISTDs


Signal 1: MWD1 A, Sig=254,4 Ref=off
 Signal has been modified after loading from rawdata file!

Peak RetTime Type  Width     Area      Height     Area
  #   [min]        [min]   [mAU*s]     [mAU]        %
----|-------|----|-------|----------|----------|--------|
   1  11.251 MM    0.1223    6.63512 9.04162e-1   0.3895
   2  11.800 VV    0.0727 1690.51233  360.55911  99.2428
   3  12.231 MM    0.1285    6.26228 8.11981e-1   0.3676
```

Compound 48

^{1}H-NMR

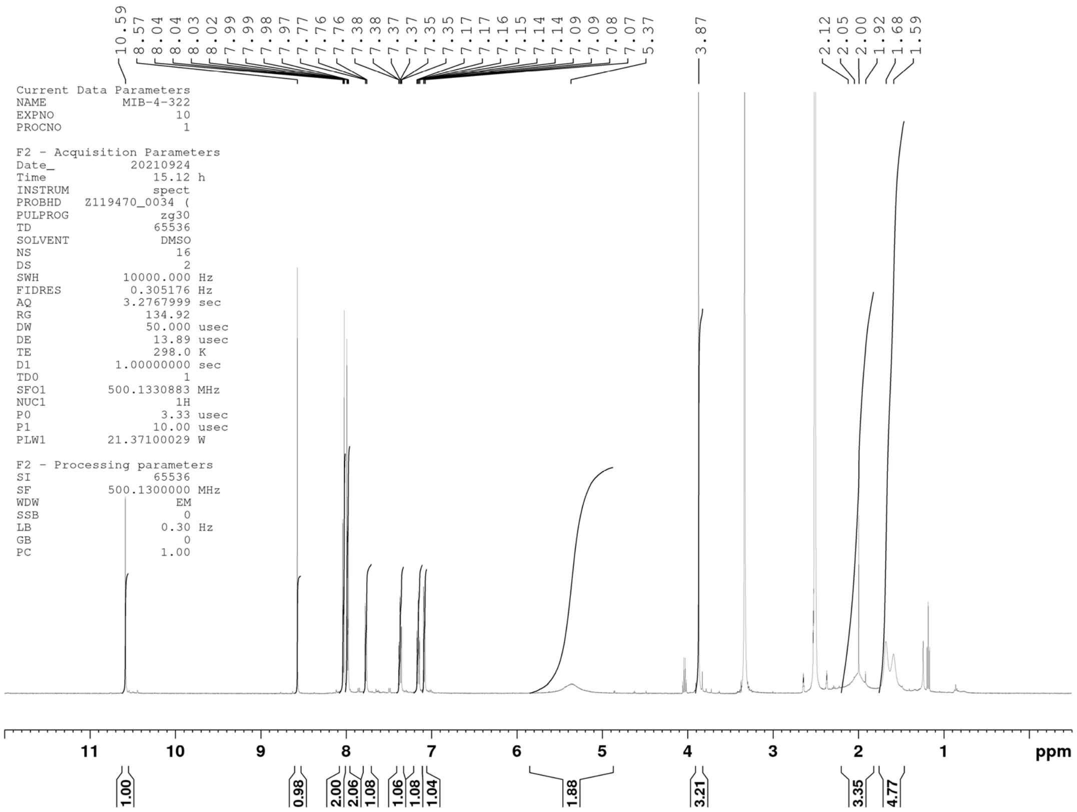

^{13}C-NMR

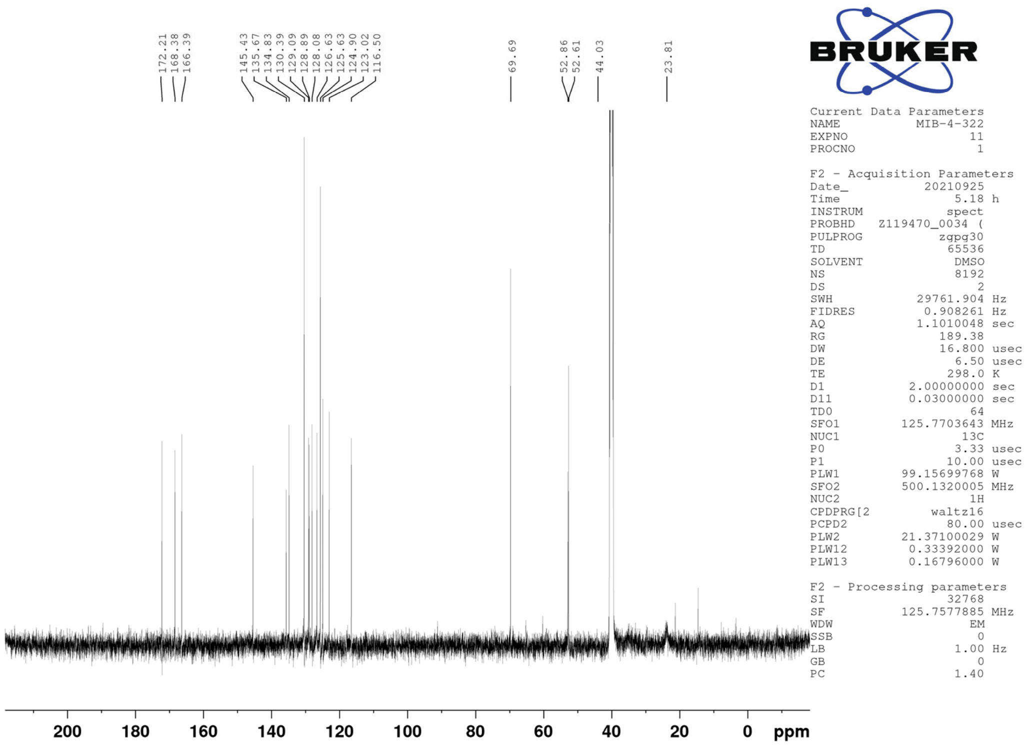

LC

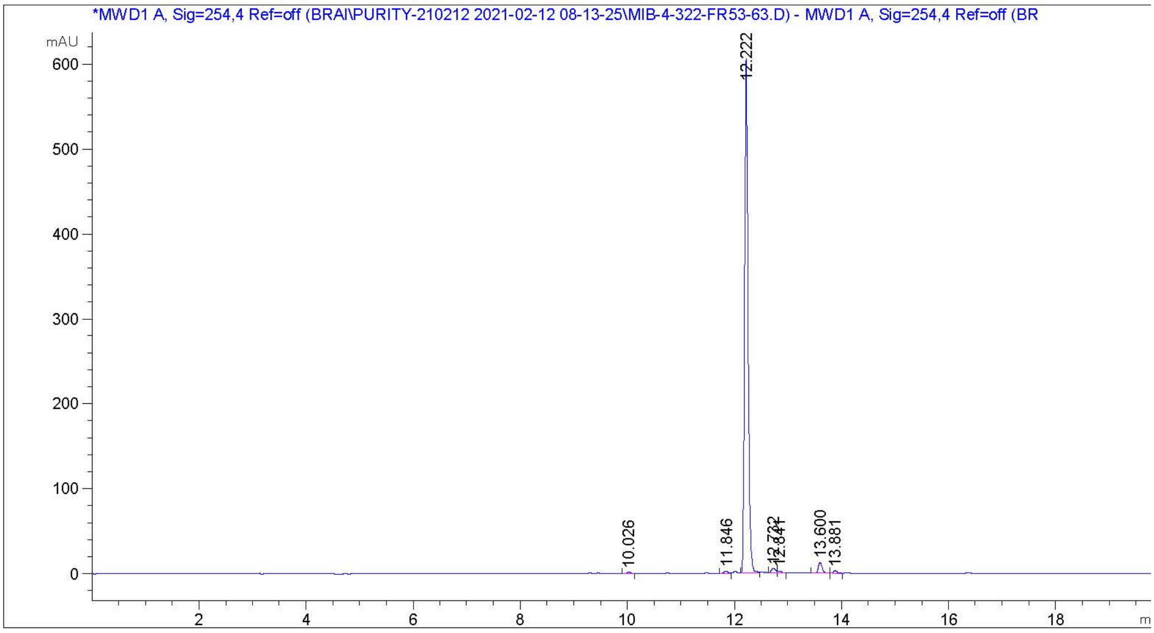

===

Area Percent Report

===

```
Sorted By             :      Signal
Multiplier            :      1.0000
Dilution              :      1.0000
Use Multiplier & Dilution Factor with ISTDs
```

Signal 1: MWD1 A, Sig=254,4 Ref=off
Signal has been modified after loading from rawdata file!

Peak #	RetTime [min]	Type	Width [min]	Area [mAU*s]	Height [mAU]	Area %
1	10.026	BV	0.0675	8.21501	1.86211	0.2862
2	11.846	BV	0.0720	14.66877	3.05846	0.5110
3	12.222	BV	0.0706	2731.29468	606.05725	95.1508
4	12.732	BV	0.0938	27.99298	4.81930	0.9752
5	12.841	VB	0.0663	10.42537	2.32458	0.3632
6	13.600	BV	0.0736	62.51662	13.10691	2.1779
7	13.881	VB	0.0762	15.37740	3.08325	0.5357

Compound 49

^{1}H-NMR

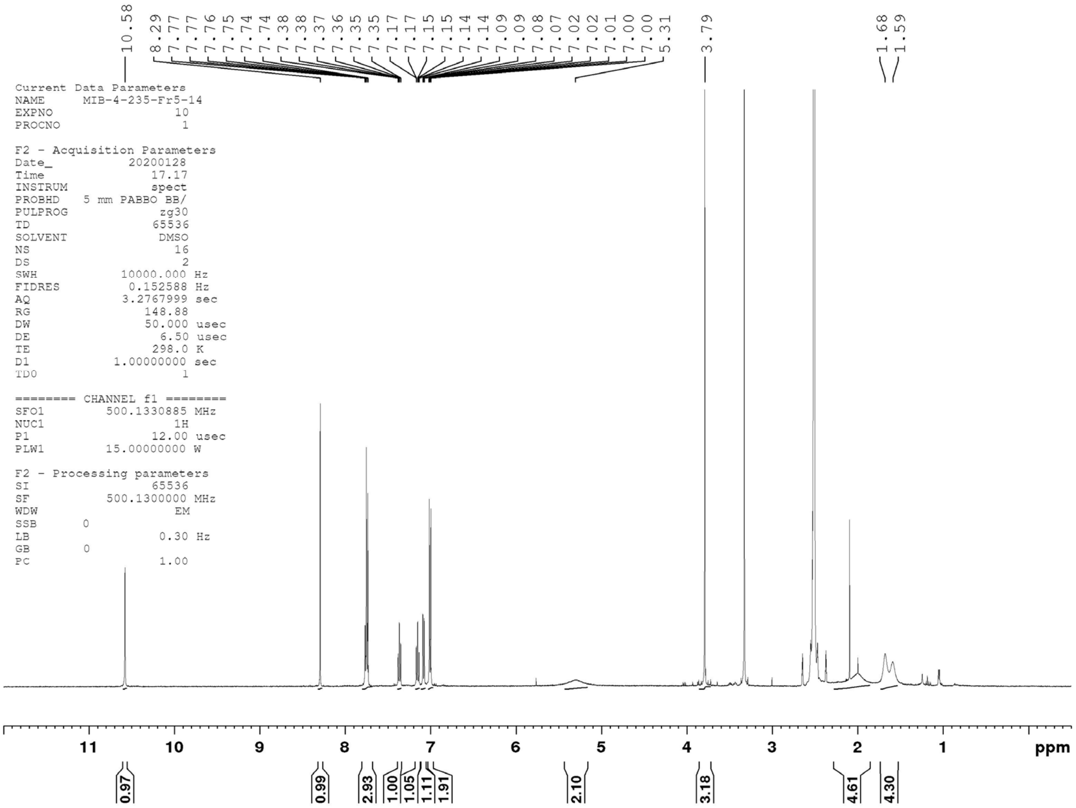

^{13}C-NMR

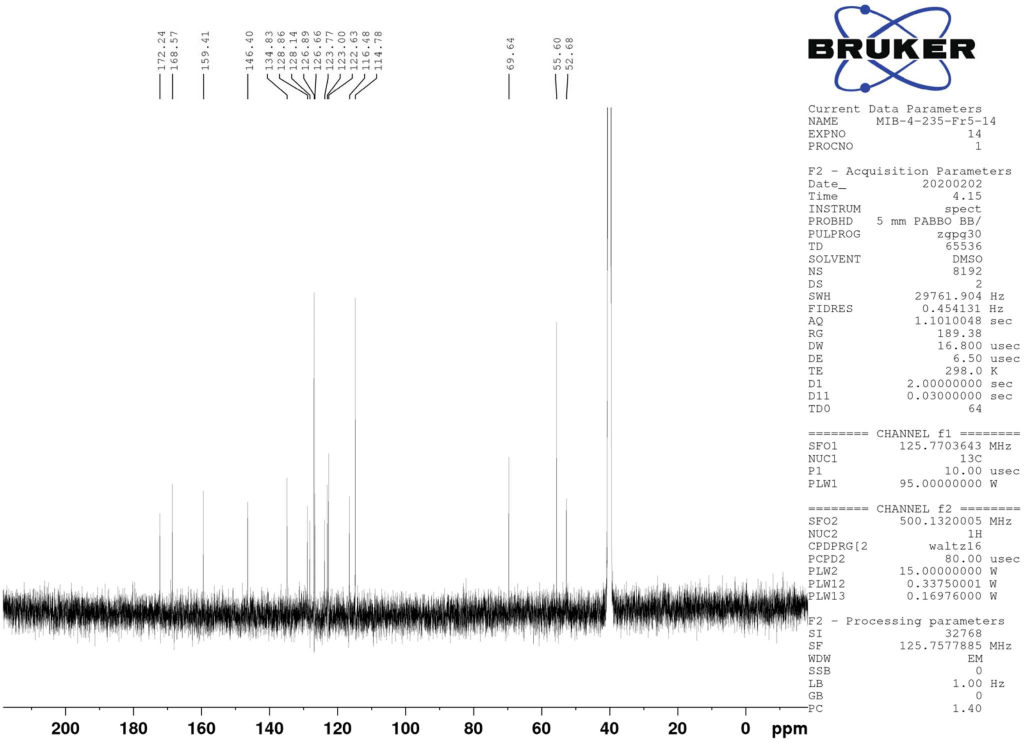

LC

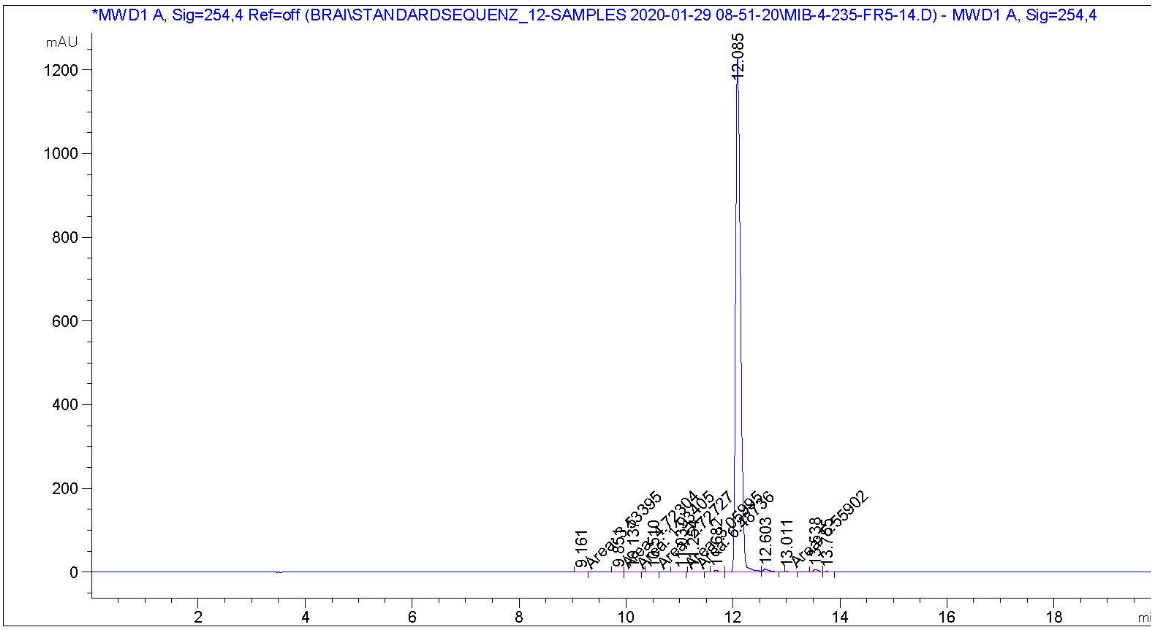

===

Area Percent Report

===

Sorted By : Signal
Multiplier : 1.0000
Dilution : 1.0000
Use Multiplier & Dilution Factor with ISTDs

Signal 1: MWD1 A, Sig=254,4 Ref=off
Signal has been modified after loading from rawdata file!

Peak #	RetTime [min]	Type	Width [min]	Area [mAU*s]	Height [mAU]	Area %
1	9.161	MM	0.1302	3.53395	4.52261e-1	0.0447
2	9.851	MM	0.0864	4.72304	9.10863e-1	0.0597
3	10.137	MM	0.1061	1.93405	3.03688e-1	0.0244
4	10.510	MM	0.0853	2.72727	5.32622e-1	0.0345
5	11.037	MM	0.1377	3.05995	3.70439e-1	0.0387
6	11.254	MM	0.1800	6.48736	6.00588e-1	0.0820
7	11.682	BB	0.1015	24.65386	3.91296	0.3116
8	12.085	BV	0.0977	7755.65039	1226.99341	98.0098
9	12.603	VB	0.1208	49.41617	6.07977	0.6245
10	13.011	MM	0.0971	5.55902	9.54264e-1	0.0703
11	13.538	BV	0.1221	41.98141	5.56161	0.5305
12	13.755	VV	0.1135	13.41397	1.82965	0.1695

Compound 50

^{1}H-NMR

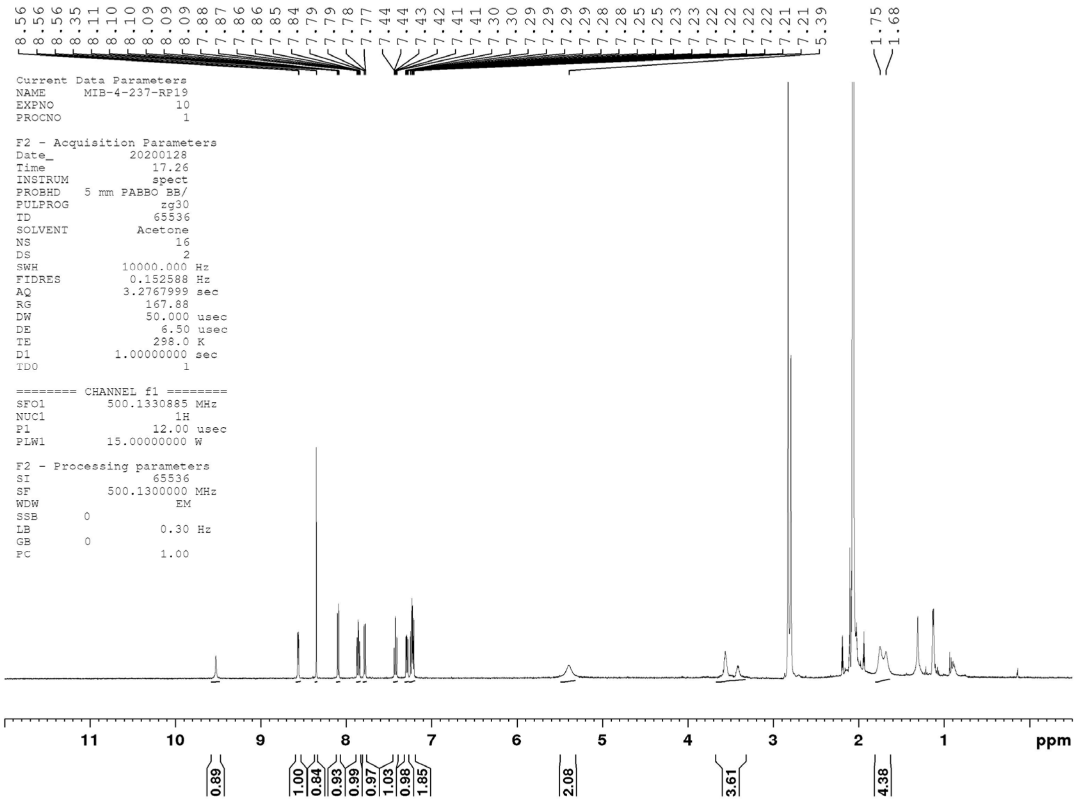

^{13}C-NMR

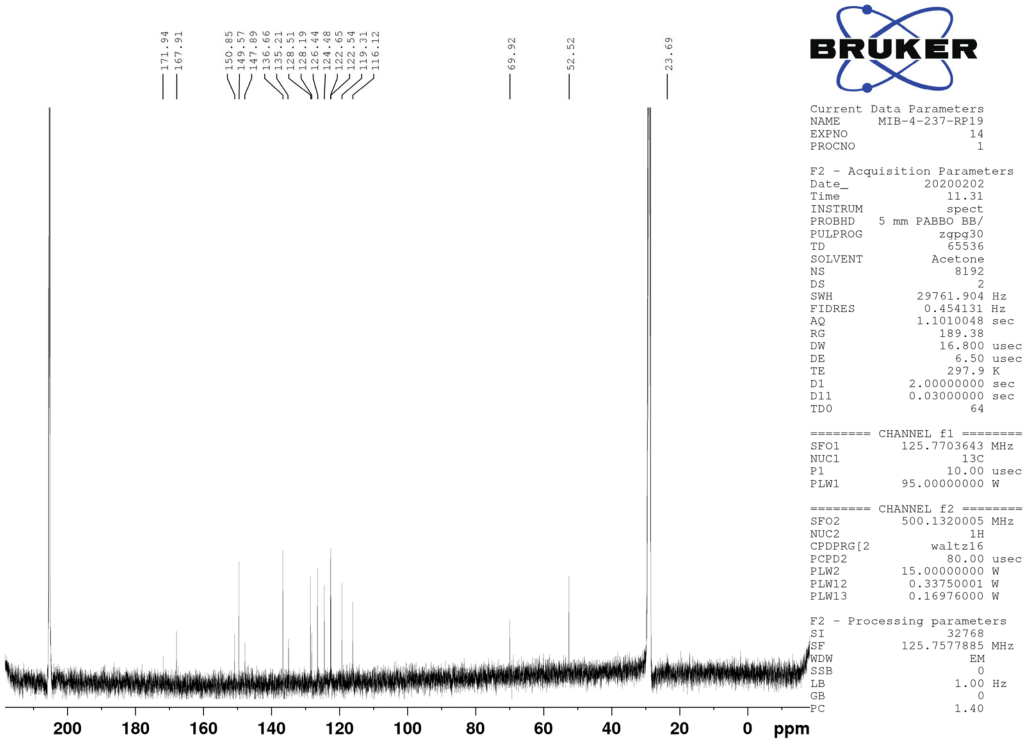

LC

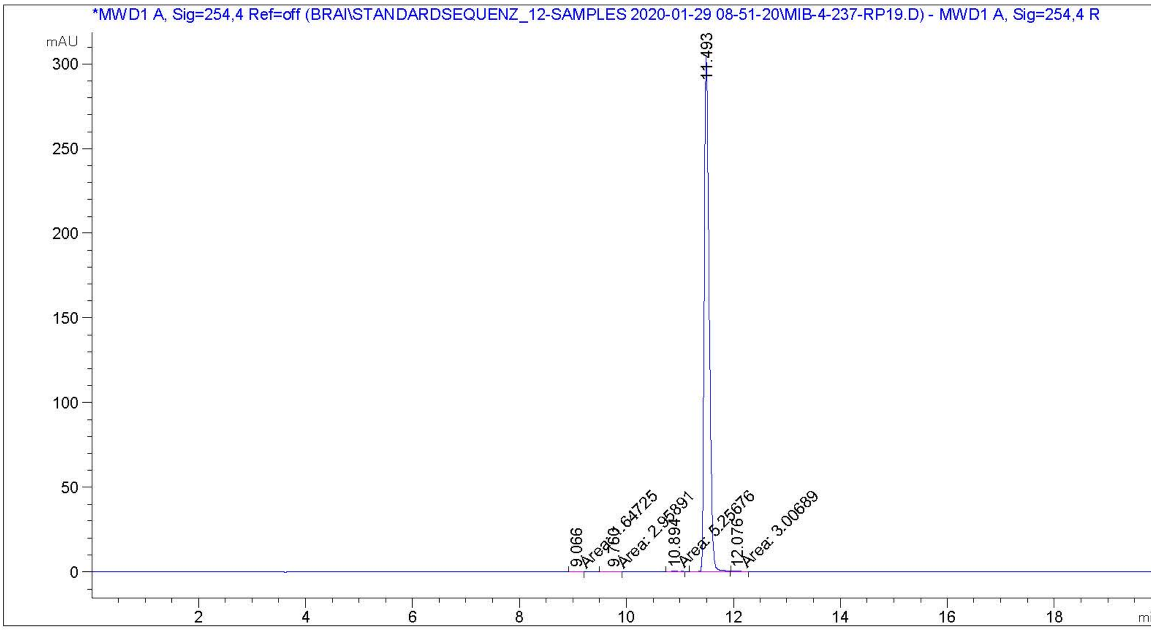


```
=====================================================================
                         Area Percent Report
=====================================================================

Sorted By             :      Signal
Multiplier            :      1.0000
Dilution              :      1.0000
Use Multiplier & Dilution Factor with ISTDs


Signal 1: MWD1 A, Sig=254,4 Ref=off
 Signal has been modified after loading from rawdata file!
```

Peak #	RetTime [min]	Type	Width [min]	Area [mAU*s]	Height [mAU]	Area %
1	9.066	MM	0.1325	1.64725	2.07139e-1	0.0858
2	9.760	MM	0.2369	2.95891	2.08200e-1	0.1541
3	10.894	MM	0.1739	5.25676	5.03742e-1	0.2738
4	11.493	BV	0.0952	1907.04333	304.07443	99.3297
5	12.076	MM	0.2205	3.00689	2.27270e-1	0.1566

Compound 51

^{1}H-NMR

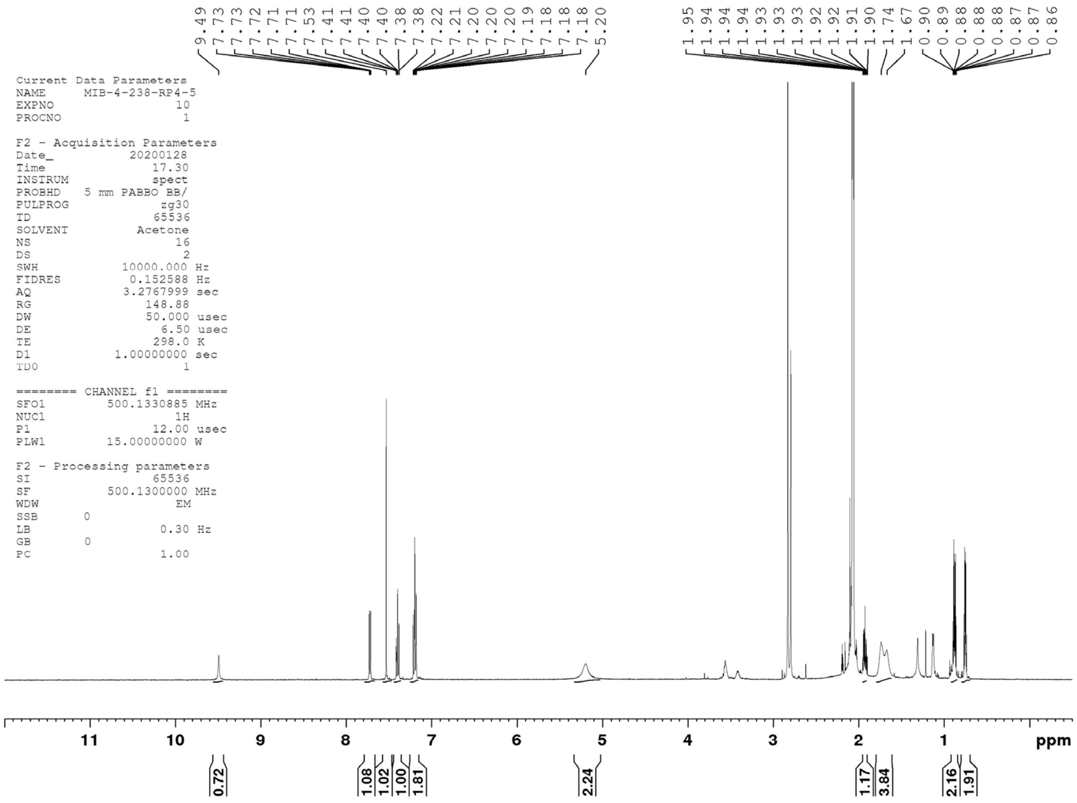

^{13}C-NMR

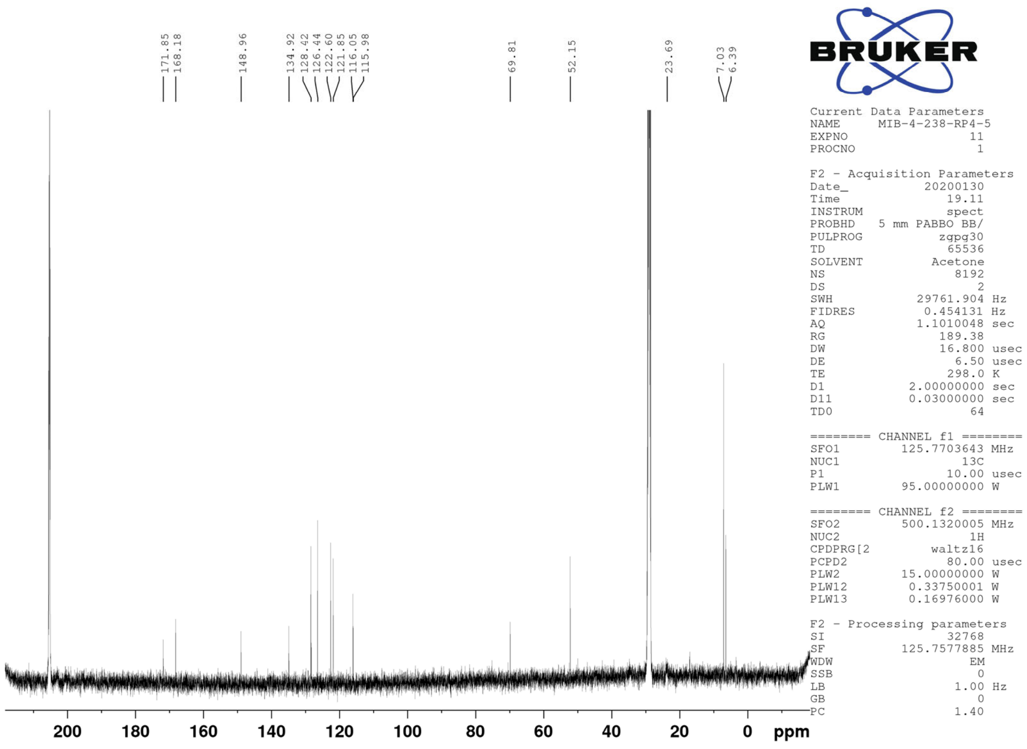

LC

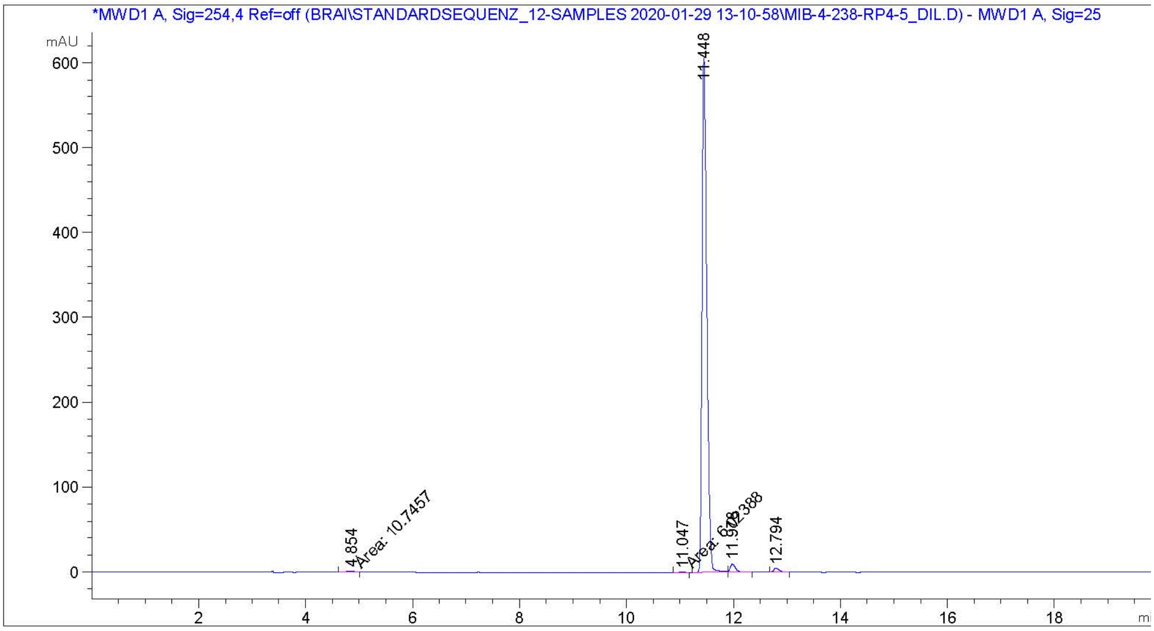


```
=====================================================================
                         Area Percent Report
=====================================================================

Sorted By             :      Signal
Multiplier            :      1.0000
Dilution              :      1.0000
Use Multiplier & Dilution Factor with ISTDs


Signal 1: MWD1 A, Sig=254,4 Ref=off
 Signal has been modified after loading from rawdata file!

Peak RetTime Type  Width     Area      Height     Area
  #   [min]        [min]   [mAU*s]     [mAU]        %
----|-------|----|-------|----------|----------|--------|
   1   4.854 MM    0.1895   10.74570 9.44862e-1   0.2774
   2  11.047 MM    0.1669    6.02388 6.01699e-1   0.1555
   3  11.448 BV    0.0964 3761.81274  606.09668  97.1119
   4  11.978 VB    0.1020   64.92233    9.71219   1.6760
   5  12.794 BB    0.1133   30.18341    4.33205   0.7792
```

Compound 52

^{1}H-NMR

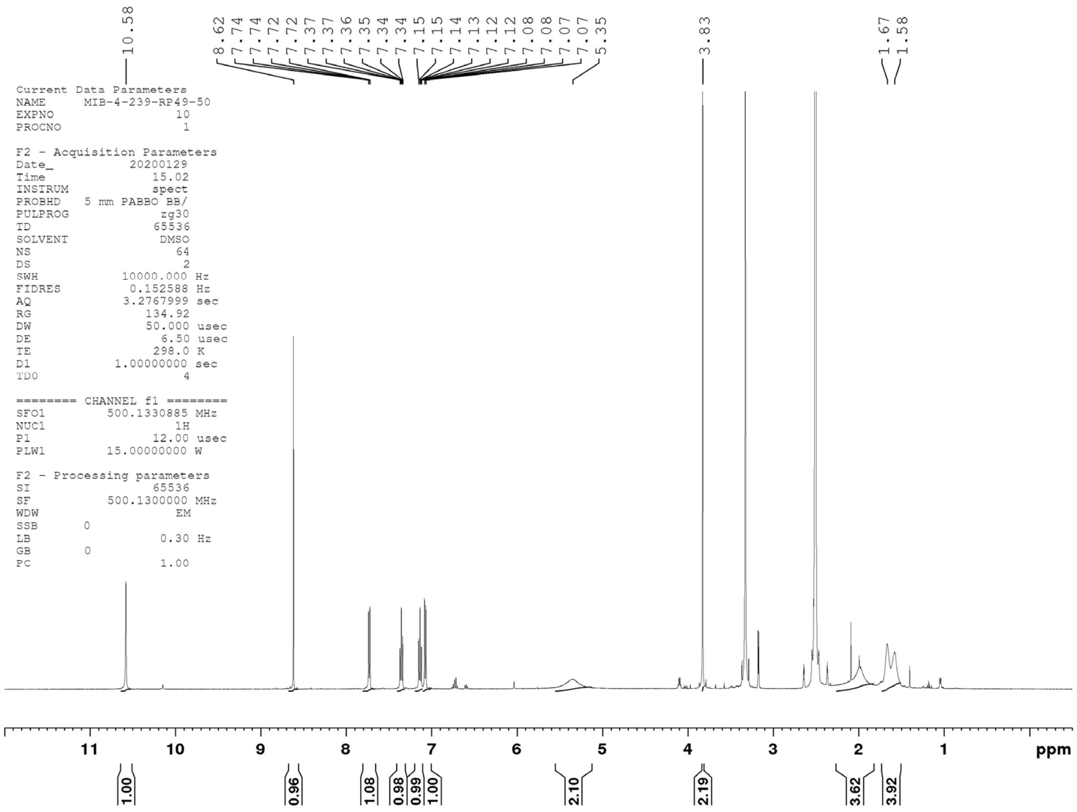

^{13}C-NMR

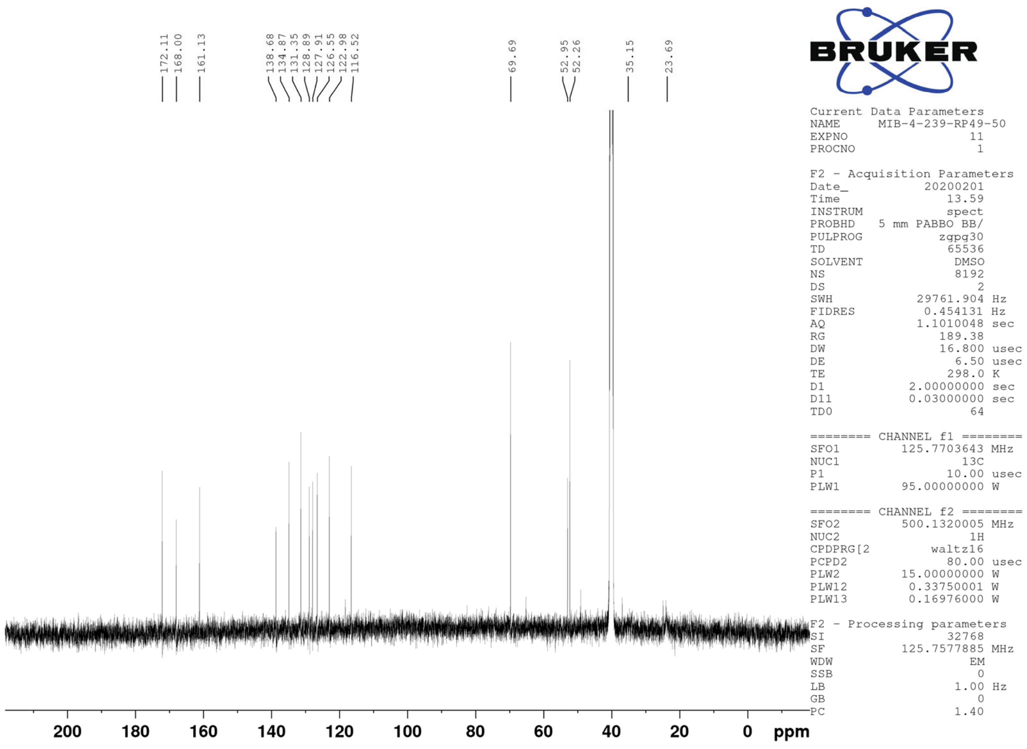

LC

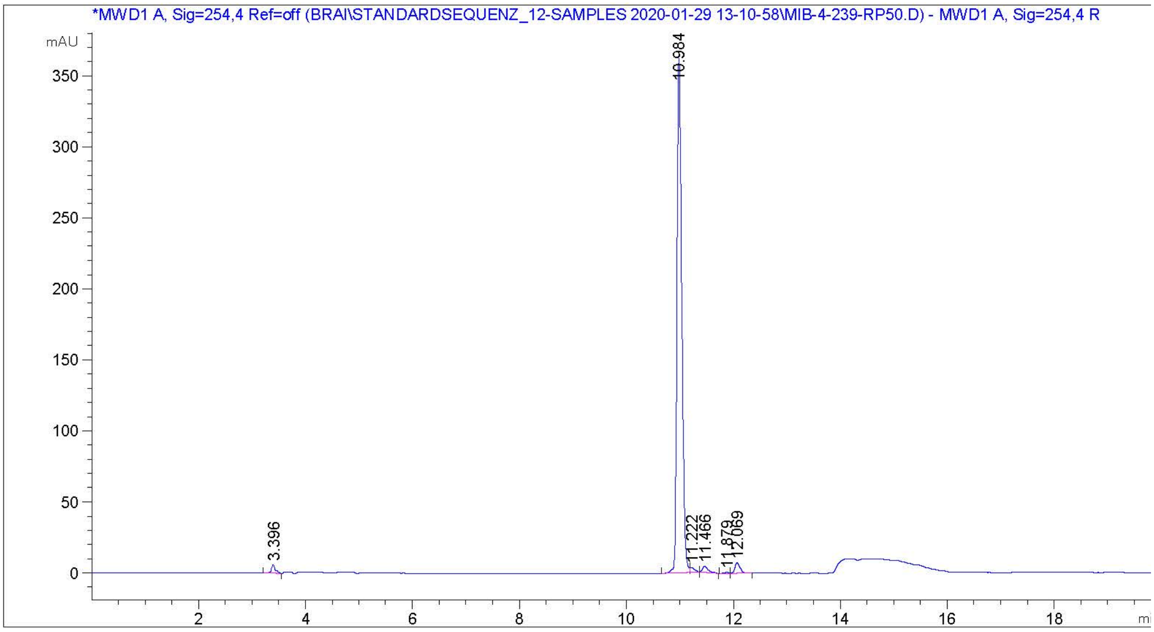

===

Area Percent Report

===

Sorted By : Signal
Multiplier : 1.0000
Dilution : 1.0000
Use Multiplier & Dilution Factor with ISTDs

Signal 1: MWD1 A, Sig=254,4 Ref=off
Signal has been modified after loading from rawdata file!

Peak #	RetTime [min]	Type	Width [min]	Area [mAU*s]	Height [mAU]	Area %
1	3.396	BB	0.0783	31.21430	5.84831	1.3368
2	10.984	BV	0.0928	2197.71777	362.15585	94.1194
3	11.222	VB	0.0881	19.79609	3.39032	0.8478
4	11.466	BB	0.1102	31.28835	4.23284	1.3400
5	11.879	BV	0.1114	4.71324	5.88426e-1	0.2018
6	12.069	VB	0.1019	50.30114	7.53028	2.1542

Compound 53

^{1}H-NMR

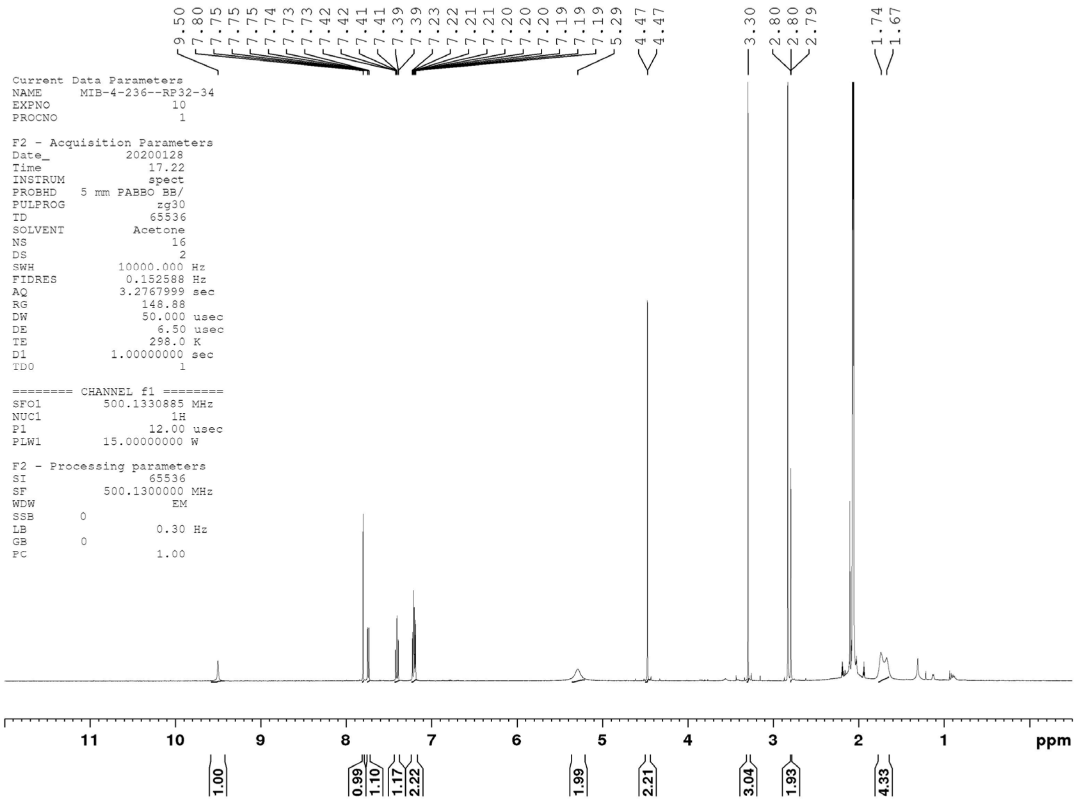

^{13}C-NMR

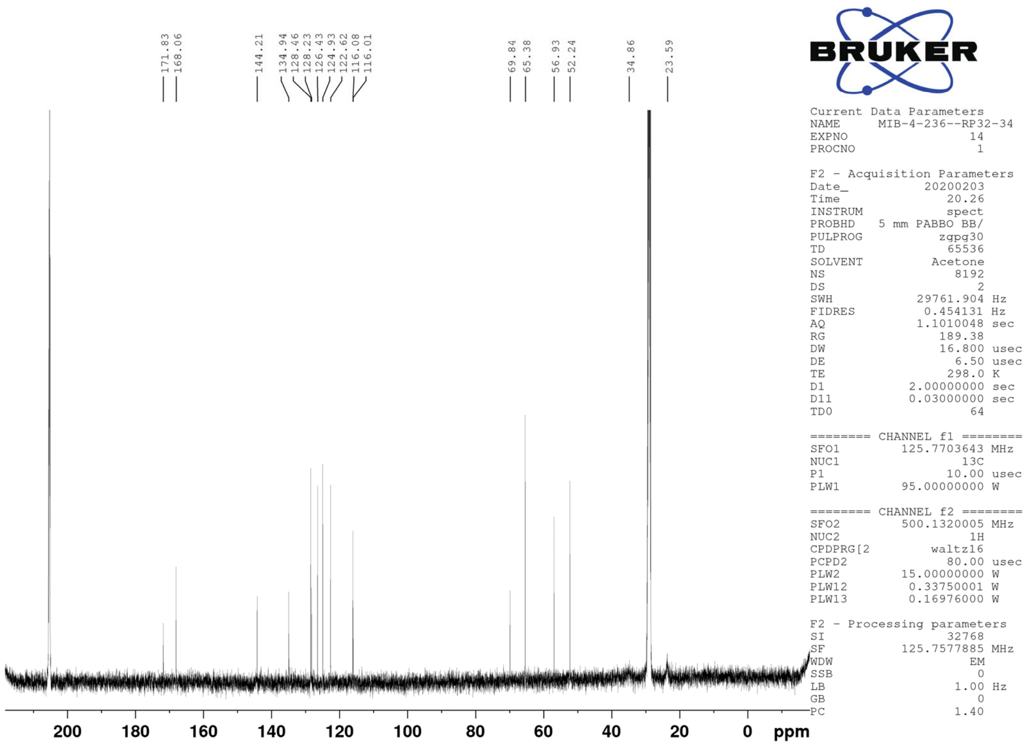

LC

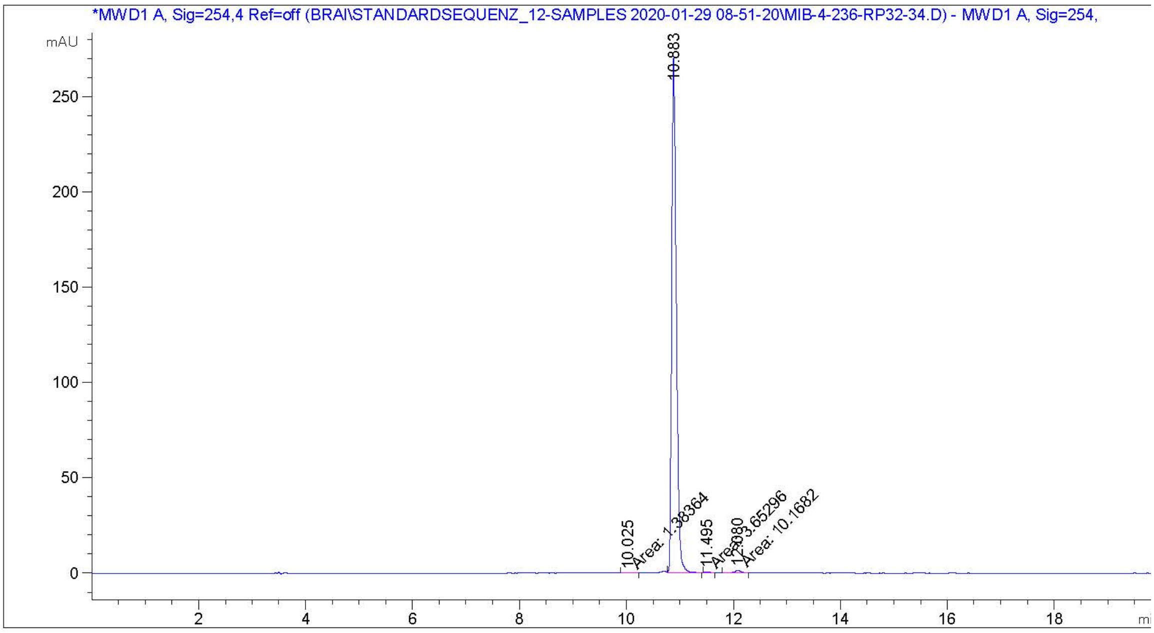


```
=====================================================================
                         Area Percent Report
=====================================================================

Sorted By             :      Signal
Multiplier            :      1.0000
Dilution              :      1.0000
Use Multiplier & Dilution Factor with ISTDs


Signal 1: MWD1 A, Sig=254,4 Ref=off
 Signal has been modified after loading from rawdata file!
```

Peak #	RetTime [min]	Type	Width [min]	Area [mAU*s]	Height [mAU]	Area %
1	10.025	MM	0.1318	1.38364	1.74931e-1	0.0835
2	10.883	VV	0.0929	1642.53210	270.19199	99.0828
3	11.495	MM	0.1076	3.65296	5.65791e-1	0.2204
4	12.080	MM	0.1180	10.16818	1.43630	0.6134

Compound 54

^{1}H-NMR

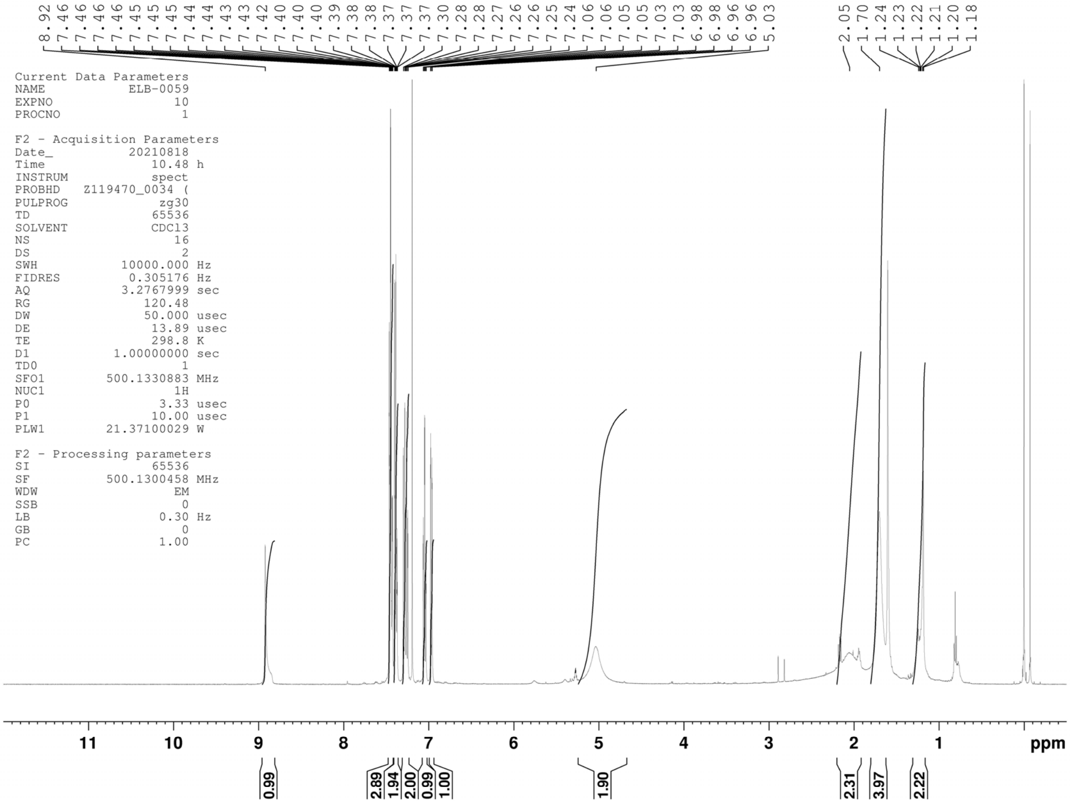

^{13}C-NMR

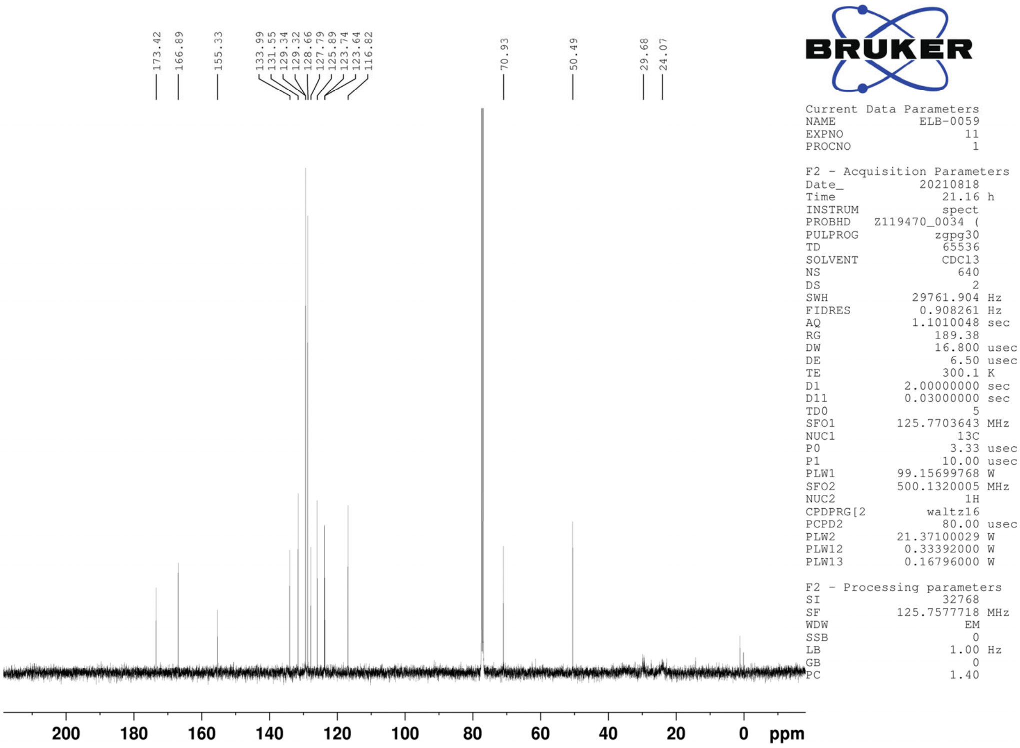

LC

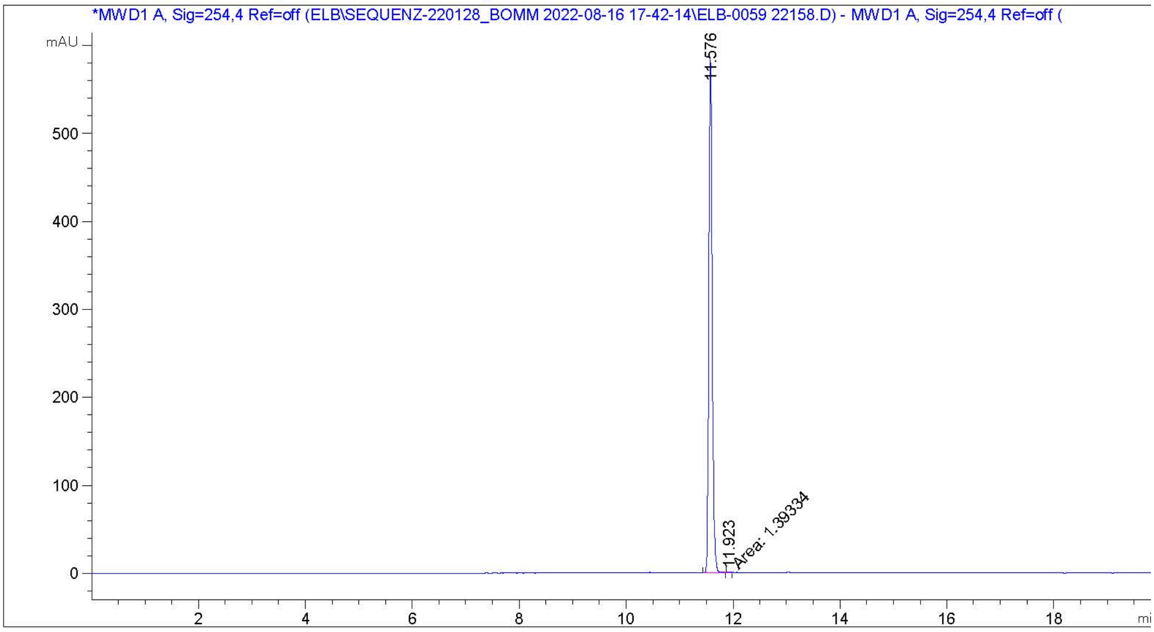


```
=====================================================================
                         Area Percent Report
=====================================================================

Sorted By             :      Signal
Multiplier            :      1.0000
Dilution              :      1.0000
Use Multiplier & Dilution Factor with ISTDs


Signal 1: MWD1 A, Sig=254,4 Ref=off
 Signal has been modified after loading from rawdata file!

Peak RetTime Type  Width     Area      Height     Area
  #   [min]        [min]   [mAU*s]     [mAU]        %
----|-------|----|-------|----------|----------|--------|
   1  11.576 BV    0.0693 2570.32397  584.90930 99.9458
   2  11.923 MM    0.0672    1.39334 3.45459e-1  0.0542

Totals :                  2571.71732  585.25476
```

Compound 55

^{1}H-NMR

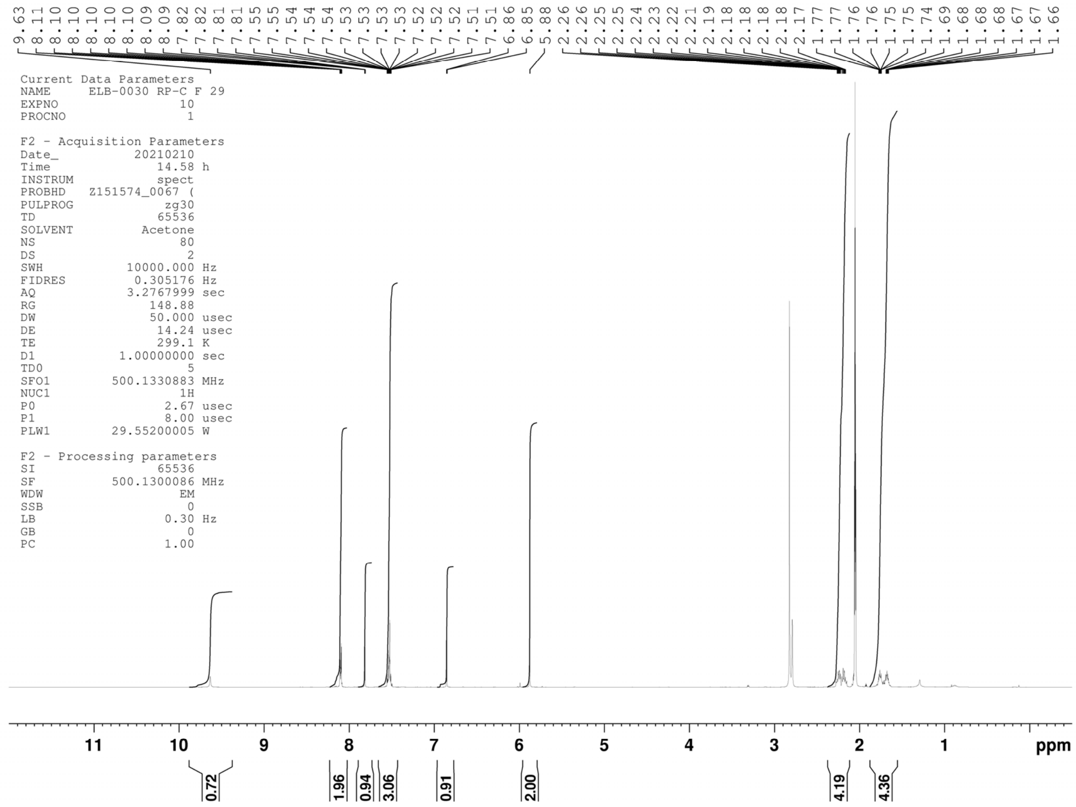

^{13}C-NMR

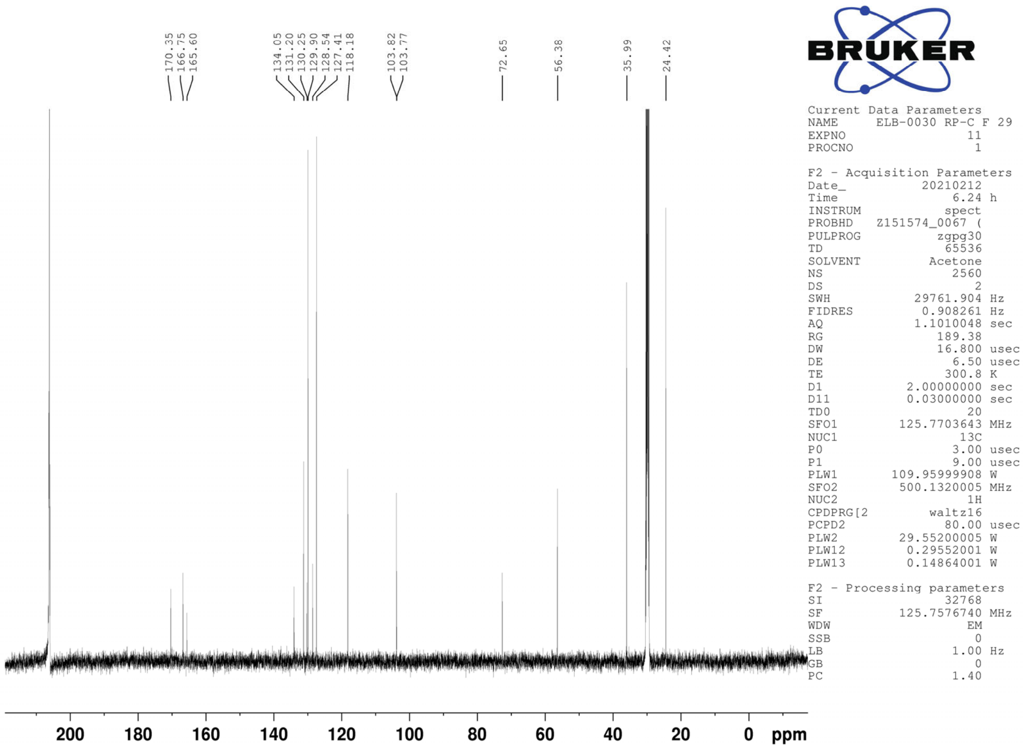

LC

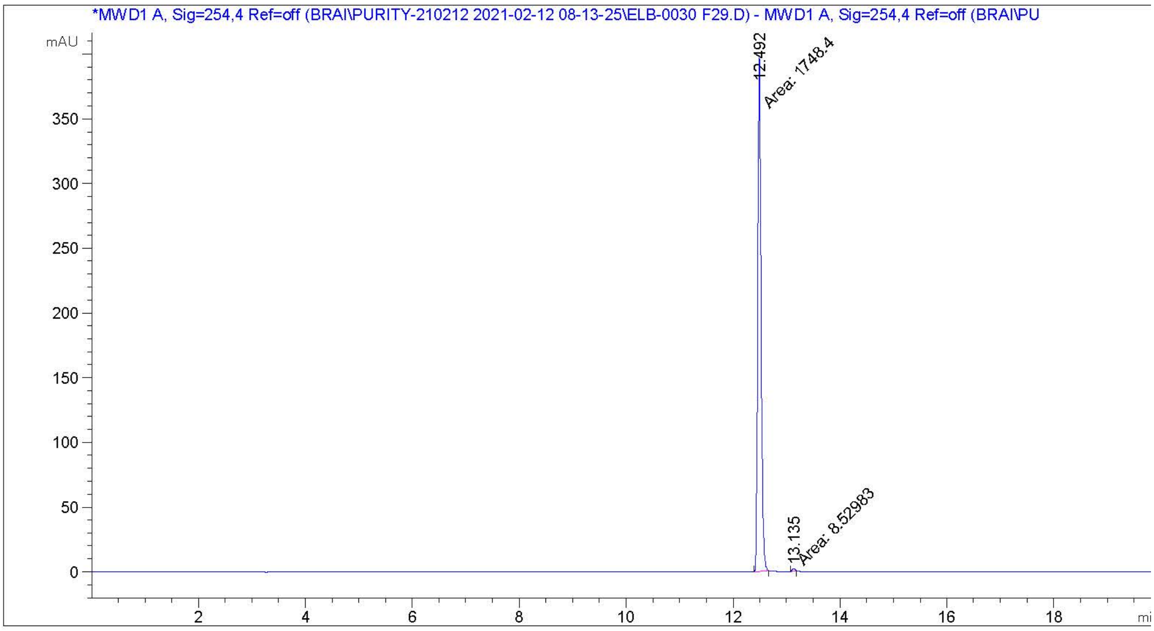


```
=====================================================================
                         Area Percent Report
=====================================================================

Sorted By             :      Signal
Multiplier            :      1.0000
Dilution              :      1.0000
Use Multiplier & Dilution Factor with ISTDs


Signal 1: MWD1 A, Sig=254,4 Ref=off
 Signal has been modified after loading from rawdata file!

Peak RetTime Type  Width     Area      Height     Area
  #   [min]        [min]   [mAU*s]     [mAU]        %
----|-------|----|-------|----------|----------|--------|
   1  12.492 MM    0.0729 1748.40356  399.80600  99.5145
   2  13.135 MM    0.0650    8.52983    2.18628   0.4855

Totals :                  1756.93340  401.99228
```

Compound 56

^{1}H-NMR

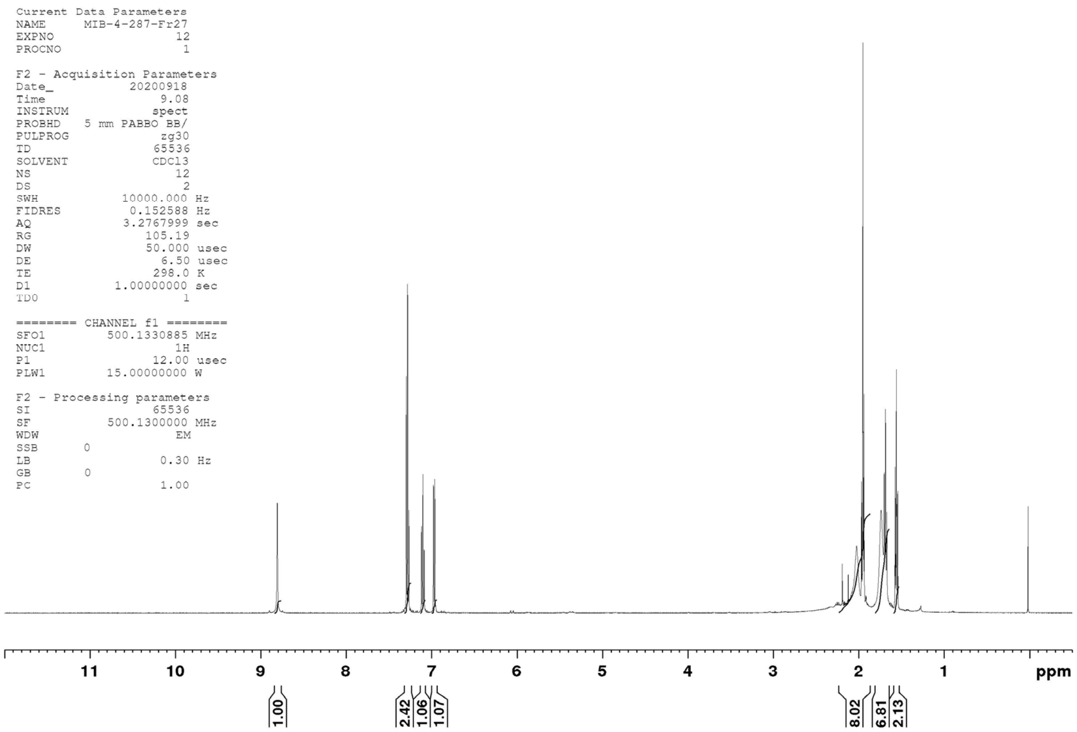

^{13}C-NMR

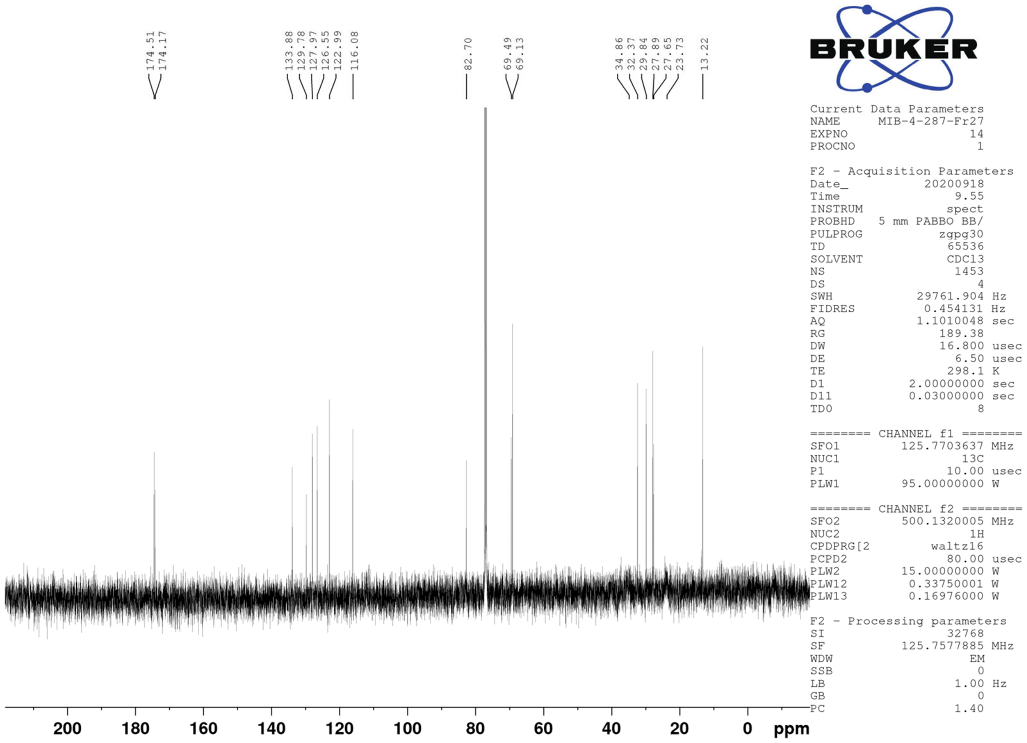

LC

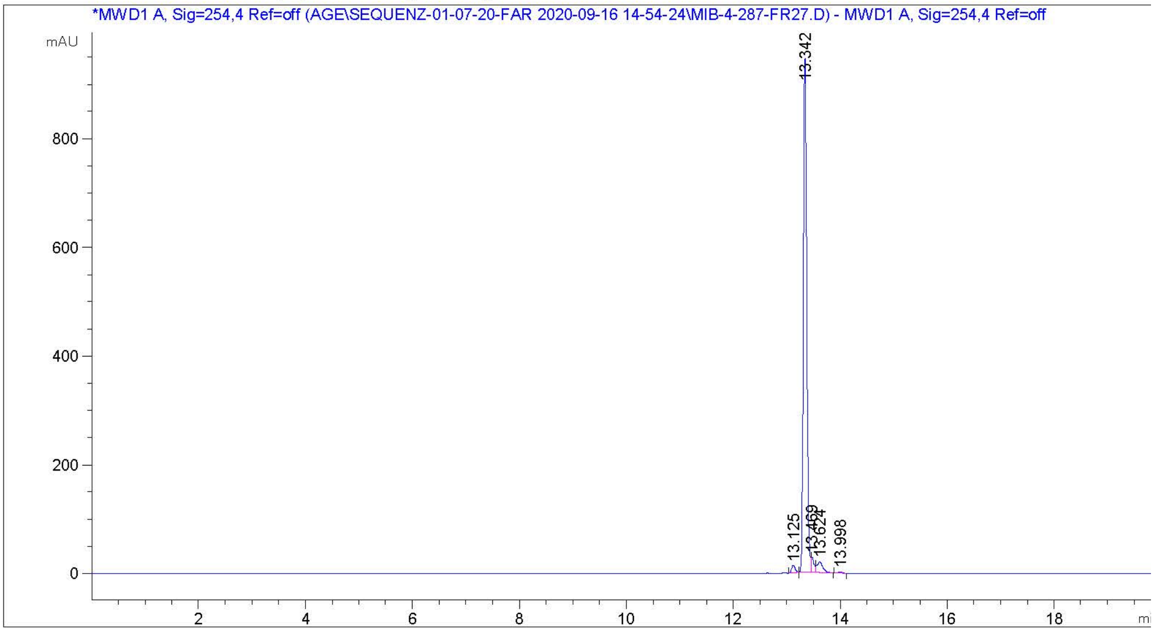

===

Area Percent Report

===

Sorted By : Signal
Multiplier : 1.0000
Dilution : 1.0000
Use Multiplier & Dilution Factor with ISTDs

Signal 1: MWD1 A, Sig=254,4 Ref=off
Signal has been modified after loading from rawdata file!

Peak #	RetTime [min]	Type	Width [min]	Area [mAU*s]	Height [mAU]	Area %
1	13.125	BB	0.0705	59.63300	13.24950	1.3170
2	13.342	BV	0.0675	4192.39502	949.02539	92.5895
3	13.469	VV	0.0543	108.75386	28.63091	2.4018
4	13.624	VB	0.1054	156.64952	20.43171	3.4596
5	13.998	BB	0.0777	10.50593	2.05454	0.2320

Compound 57

^1H-NMR

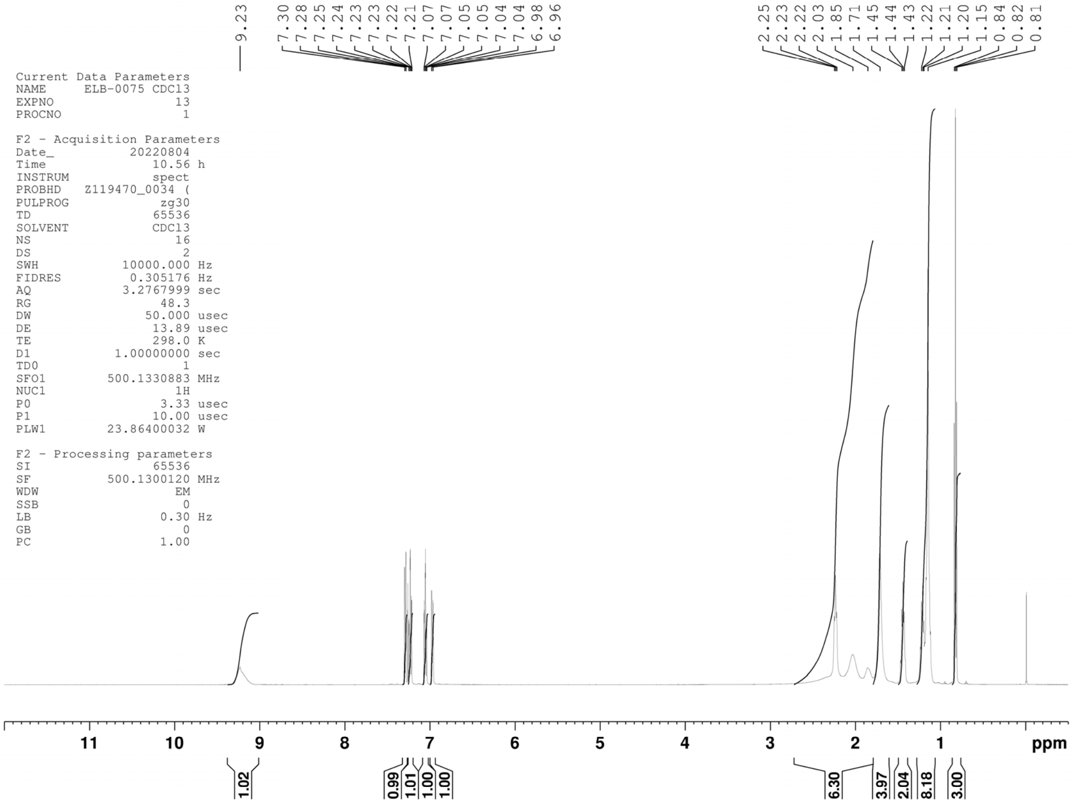

^13C-NMR

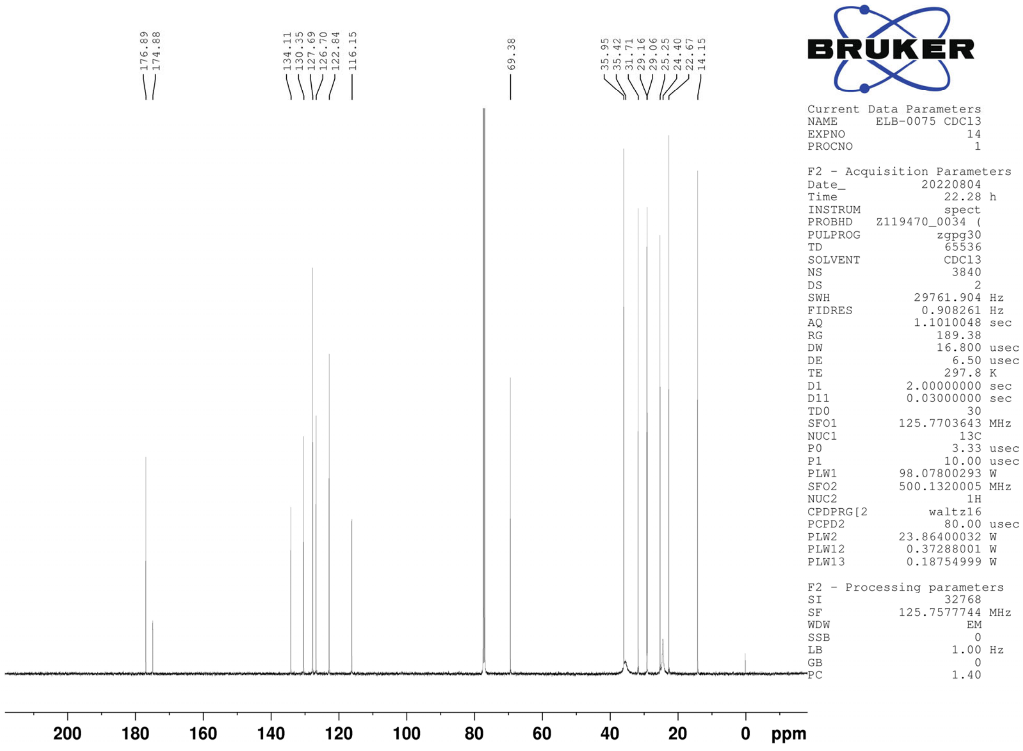

LC

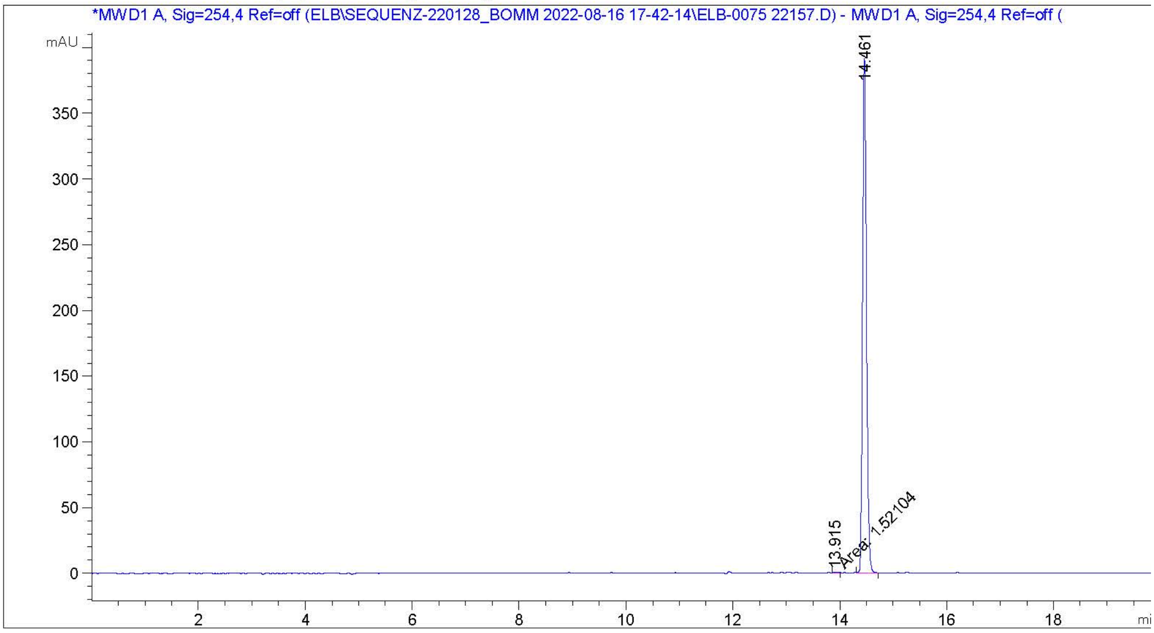


```
=====================================================================
                         Area Percent Report
=====================================================================

Sorted By             :      Signal
Multiplier            :      1.0000
Dilution              :      1.0000
Use Multiplier & Dilution Factor with ISTDs


Signal 1: MWD1 A, Sig=254,4 Ref=off
 Signal has been modified after loading from rawdata file!

Peak RetTime Type  Width     Area      Height     Area
  #   [min]        [min]   [mAU*s]     [mAU]        %
----|-------|----|-------|----------|----------|--------|
   1  13.915 MM     0.0727    1.52104 3.48565e-1   0.0787
   2  14.461 VV     0.0754 1930.66223  392.51517  99.9213

Totals :                  1932.18327  392.86373
```

Compound S1

^{1}H-NMR

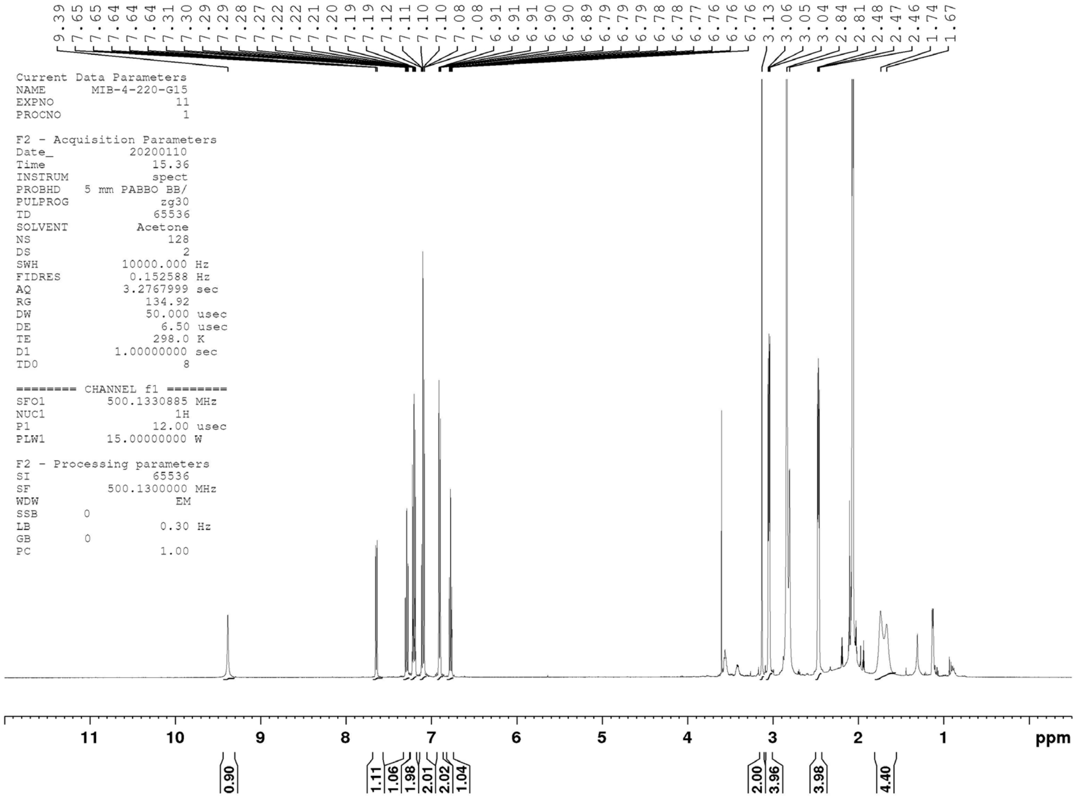

^{13}C-NMR

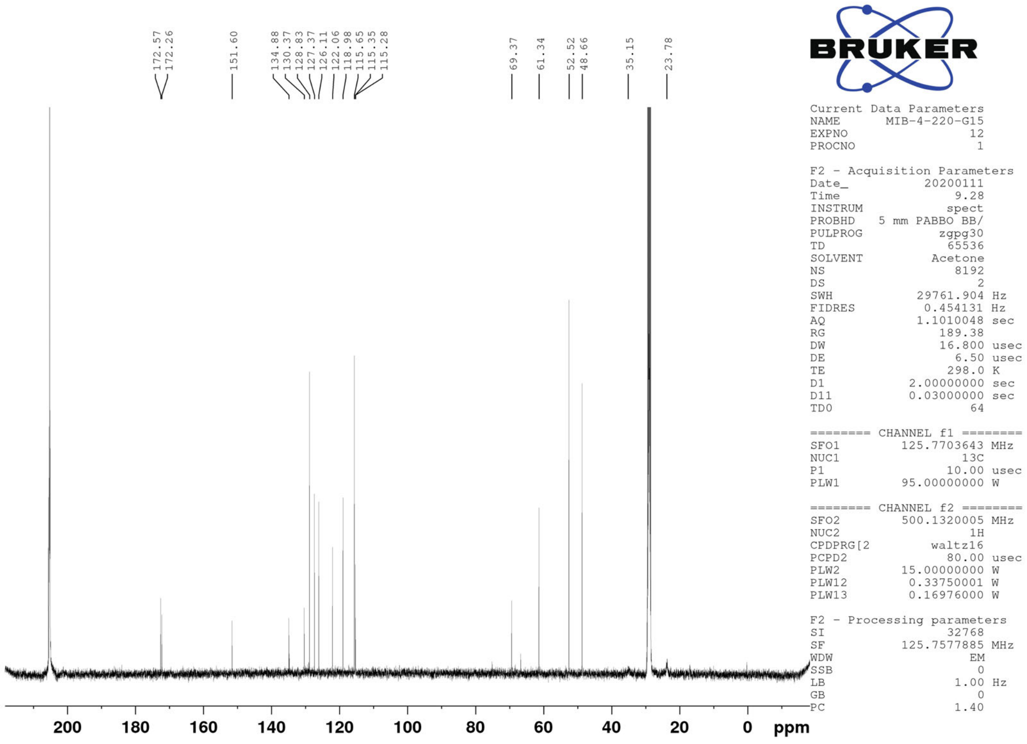

LC

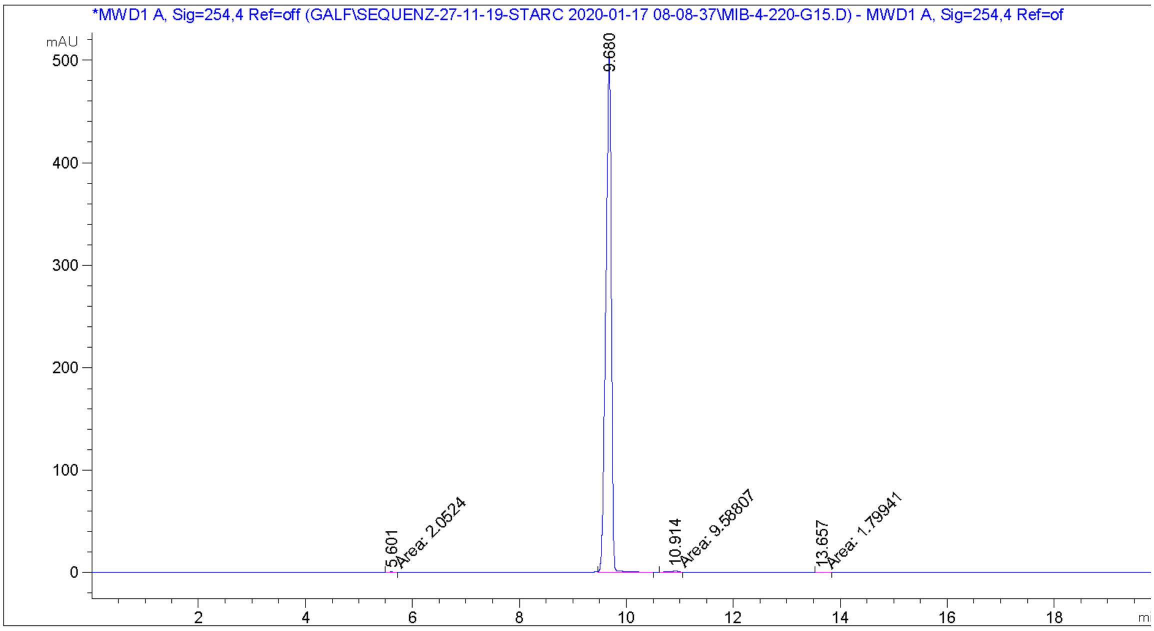


```
=====================================================================
                         Area Percent Report
=====================================================================

Sorted By             :      Signal
Multiplier            :      1.0000
Dilution              :      1.0000
Use Multiplier & Dilution Factor with ISTDs


Signal 1: MWD1 A, Sig=254,4 Ref=off
 Signal has been modified after loading from rawdata file!

Peak RetTime Type  Width     Area      Height     Area
  #   [min]        [min]   [mAU*s]     [mAU]        %
----|-------|----|-------|----------|----------|--------|
   1   5.601 MM    0.0631    2.05240 5.42125e-1   0.0582
   2   9.680 VB    0.1015 3515.44995  503.16556  99.6191
   3  10.914 MM    0.1296    9.58807    1.23314   0.2717
   4  13.657 MM    0.1266    1.79941 2.36958e-1   0.0510
```

Compound S16

^{1}H-NMR

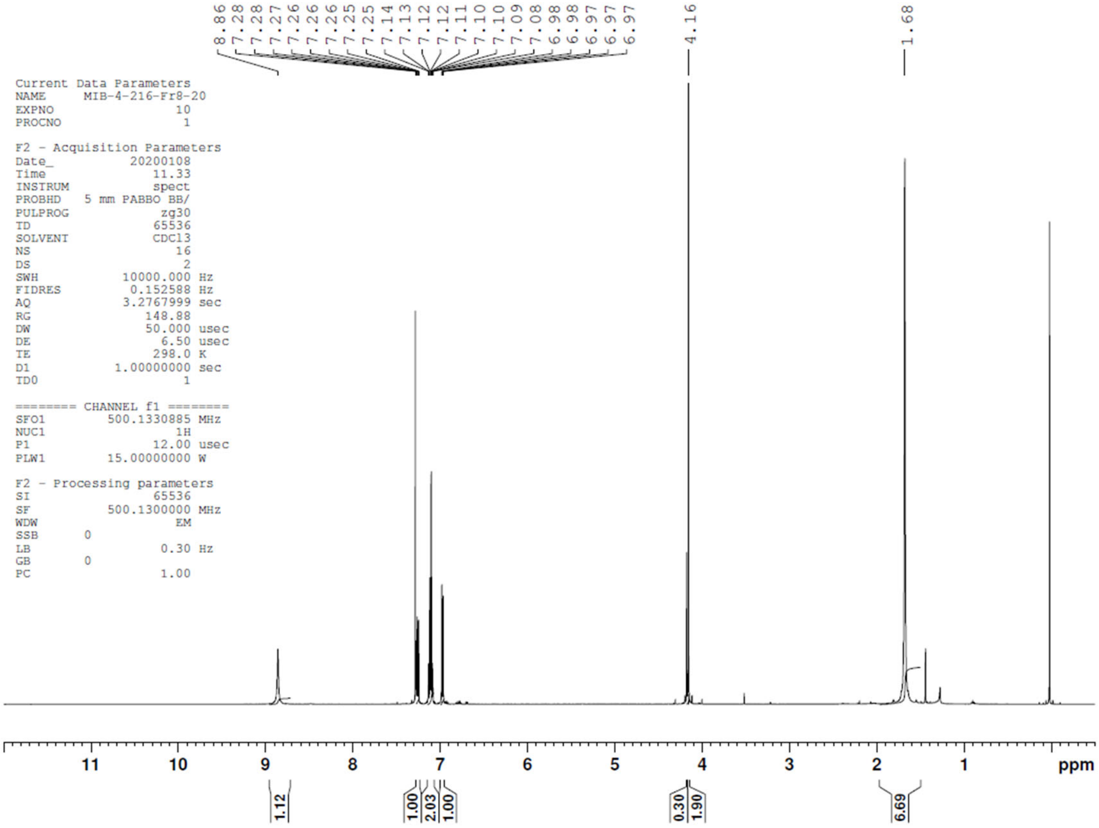

^{13}C-NMR

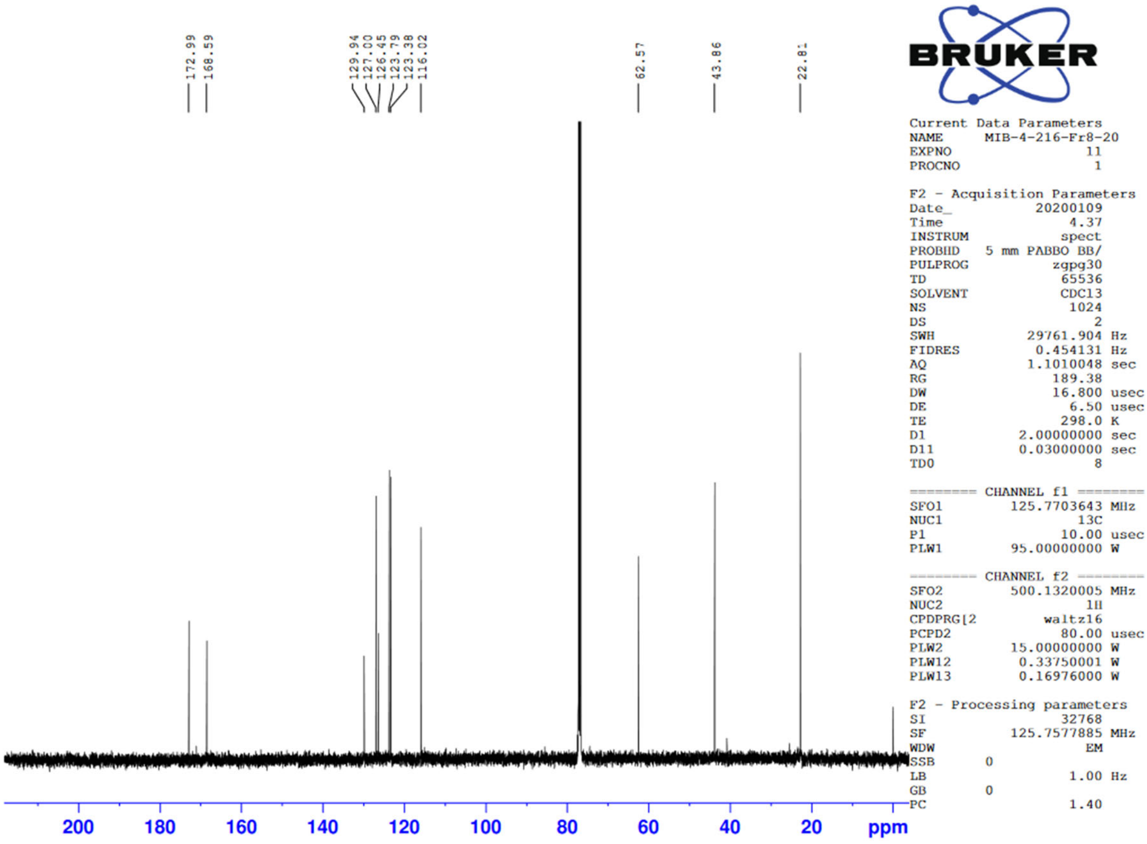

Compound S2

^{1}H-NMR

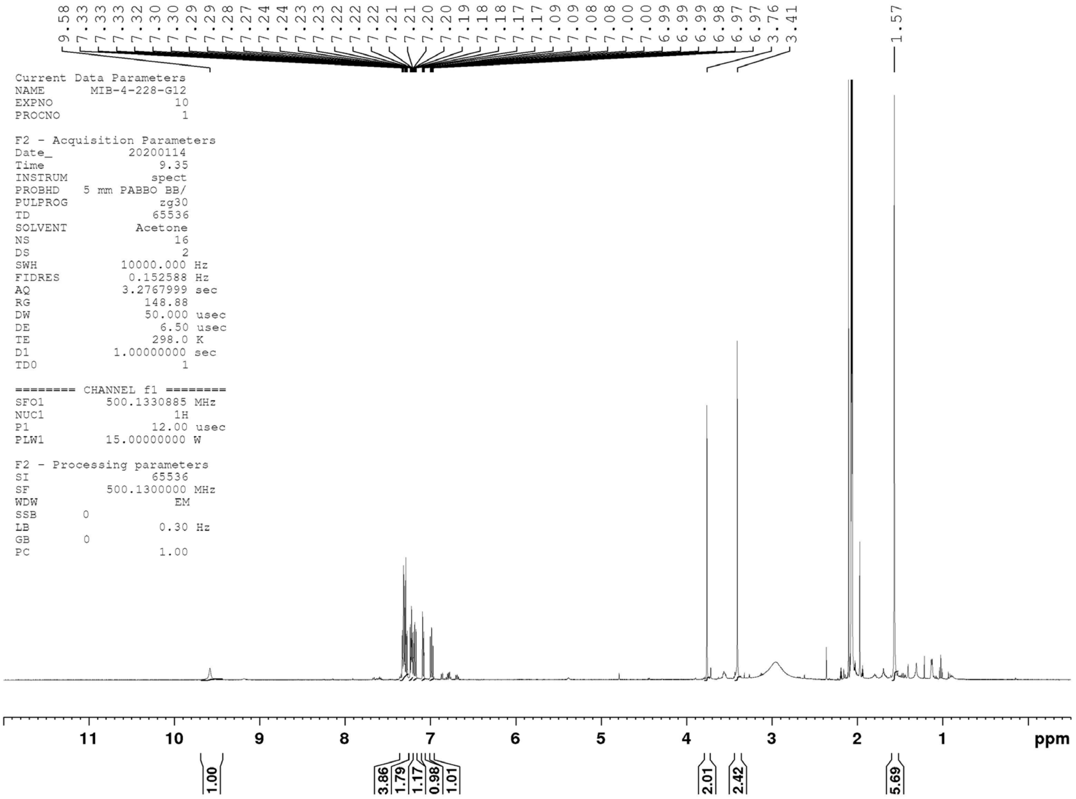

^{13}C-NMR

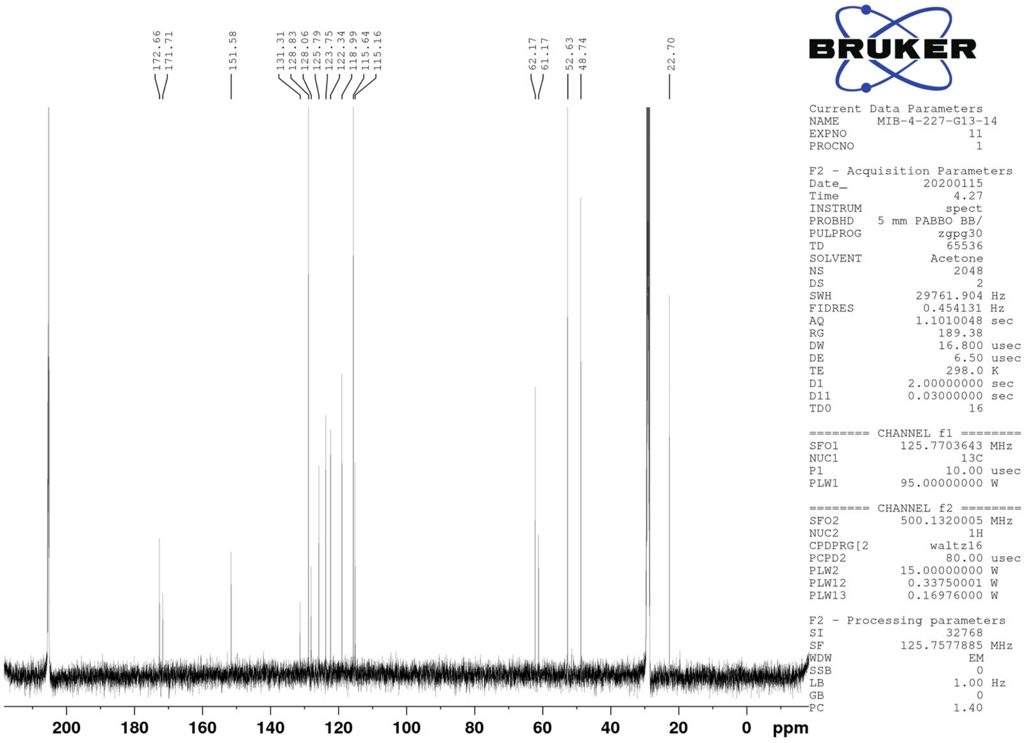

LC

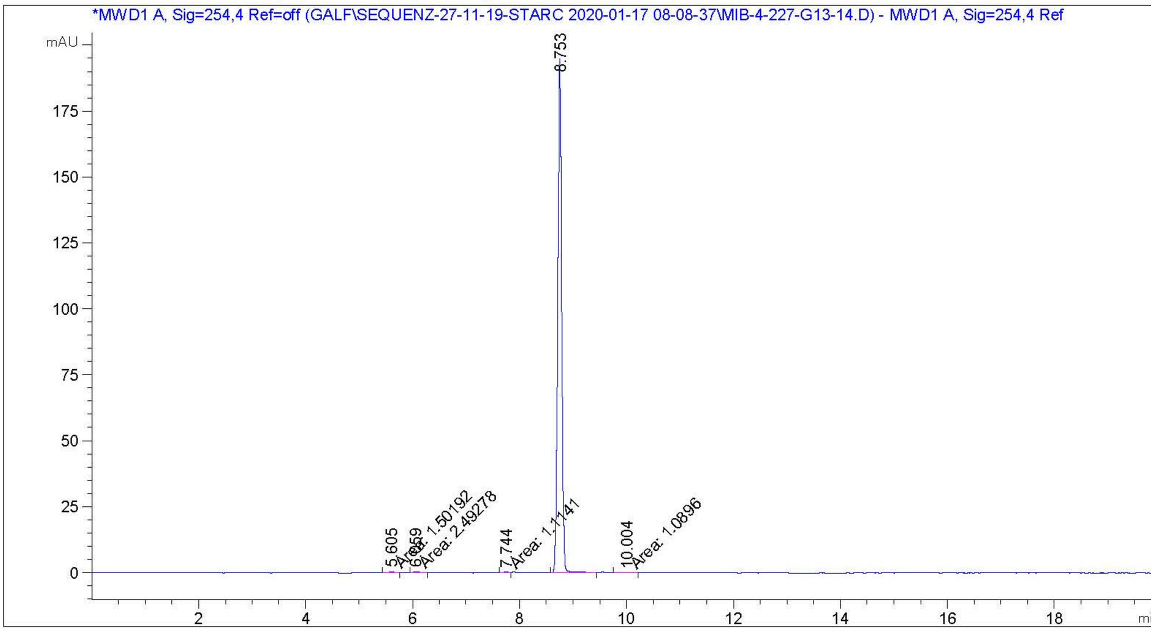


```
=====================================================================
                         Area Percent Report
=====================================================================

Sorted By             :      Signal
Multiplier            :      1.0000
Dilution              :      1.0000
Use Multiplier & Dilution Factor with ISTDs


Signal 1: MWD1 A, Sig=254,4 Ref=off
 Signal has been modified after loading from rawdata file!
```

Peak #	RetTime [min]	Type	Width [min]	Area [mAU*s]	Height [mAU]	Area %
1	5.605	MM	0.0717	1.50192	3.49111e-1	0.1508
2	6.059	MM	0.0970	2.49278	4.28321e-1	0.2503
3	7.744	MM	0.0747	1.11410	2.48439e-1	0.1119
4	8.753	BB	0.0773	989.83307	194.83113	99.3777
5	10.004	MM	0.1779	1.08960	1.02083e-1	0.1094

Compound S3

^{1}H-NMR

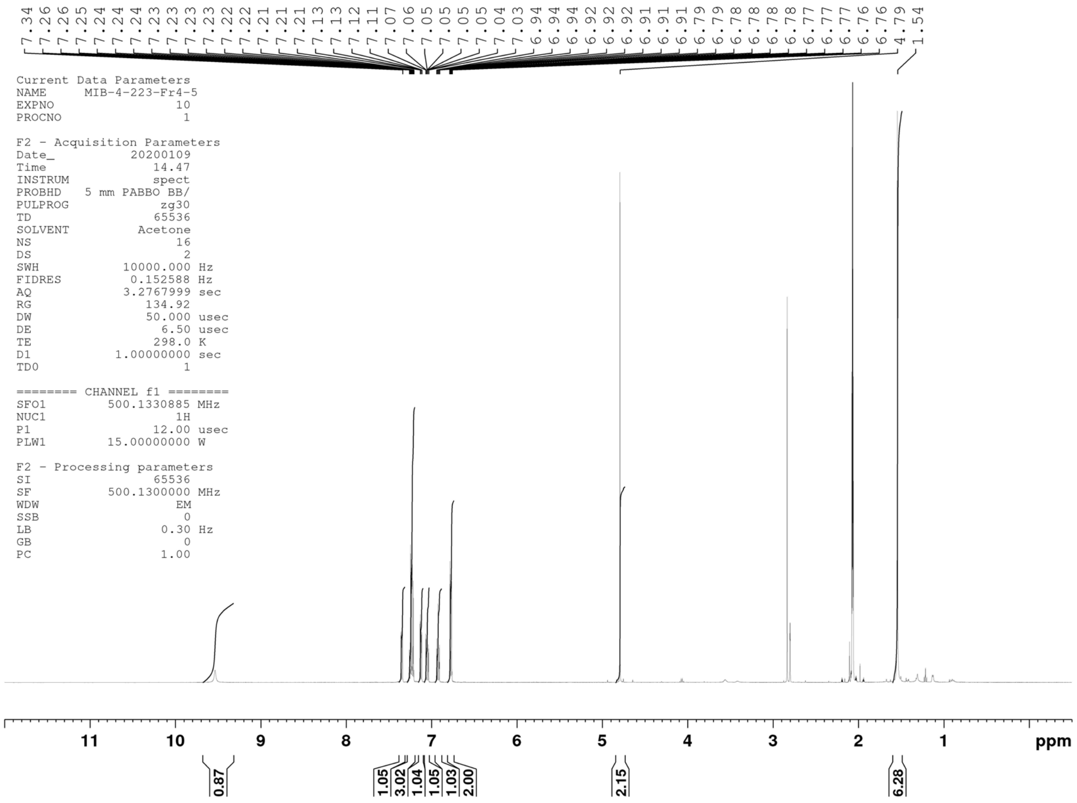

^{13}C-NMR

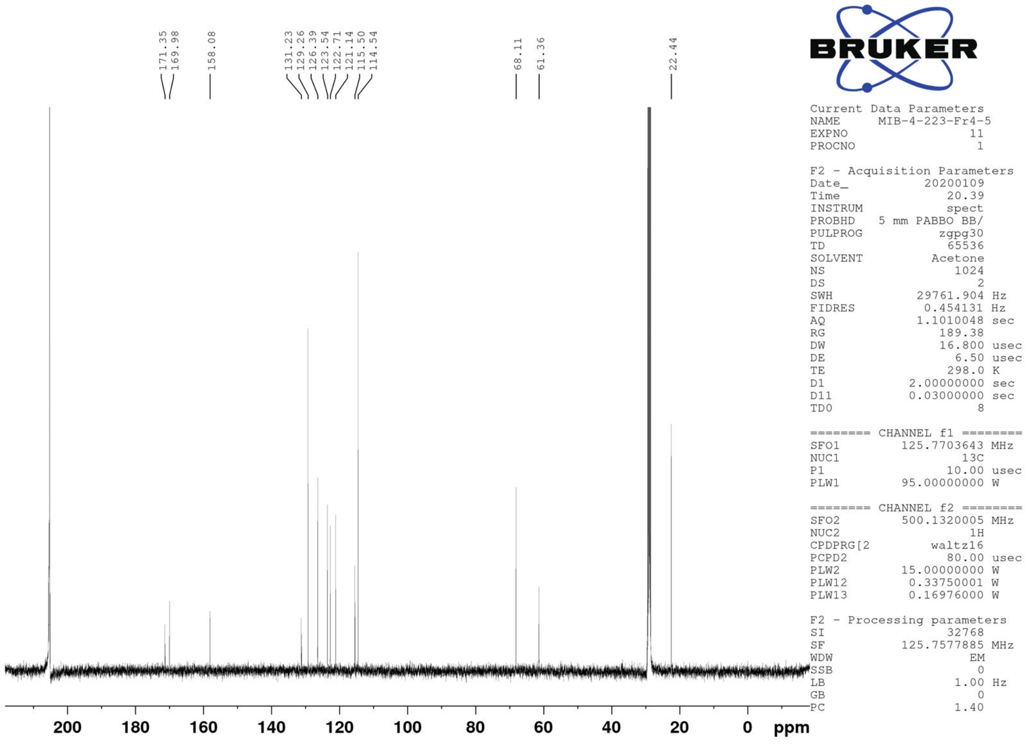

LC

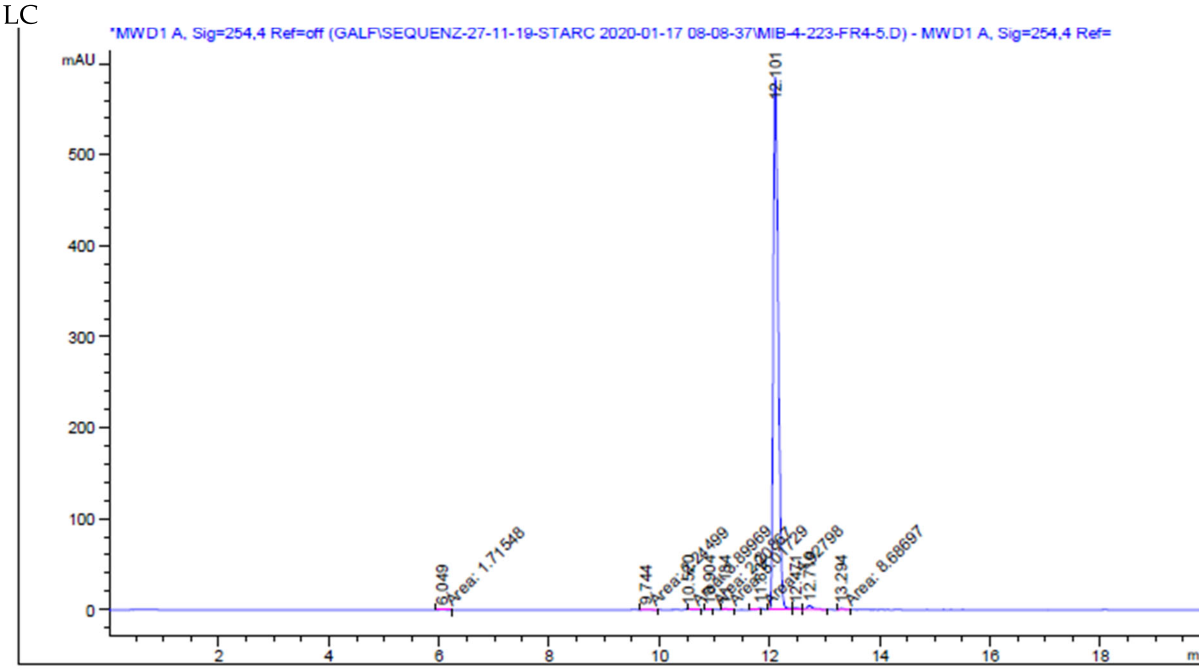


```
========================================================================
                          Area Percent Report
========================================================================

Sorted By             :      Signal
Multiplier            :      1.0000
Dilution              :      1.0000
Use Multiplier & Dilution Factor with ISTDs


Signal 1: MWD1 A, Sig=254,4 Ref=off
 Signal has been modified after loading from rawdata file!
```

Peak #	RetTime [min]	Type	Width [min]	Area [mAU*s]	Height [mAU]	Area %
1	6.049	MM	0.1192	1.71548	2.39828e-1	0.0465
2	9.744	MM	0.1080	2.24499	3.46585e-1	0.0609
3	10.520	MM	0.0773	3.89969	8.40470e-1	0.1057
4	10.904	MM	0.0900	2.20867	4.09218e-1	0.0599
5	11.184	MM	0.0968	5.01729	8.64126e-1	0.1361
6	11.822	MM	0.0696	4.92798	1.18017	0.1336
7	12.101	BV	0.0958	3606.28735	585.83331	97.7934

Compound S4

^{1}H-NMR

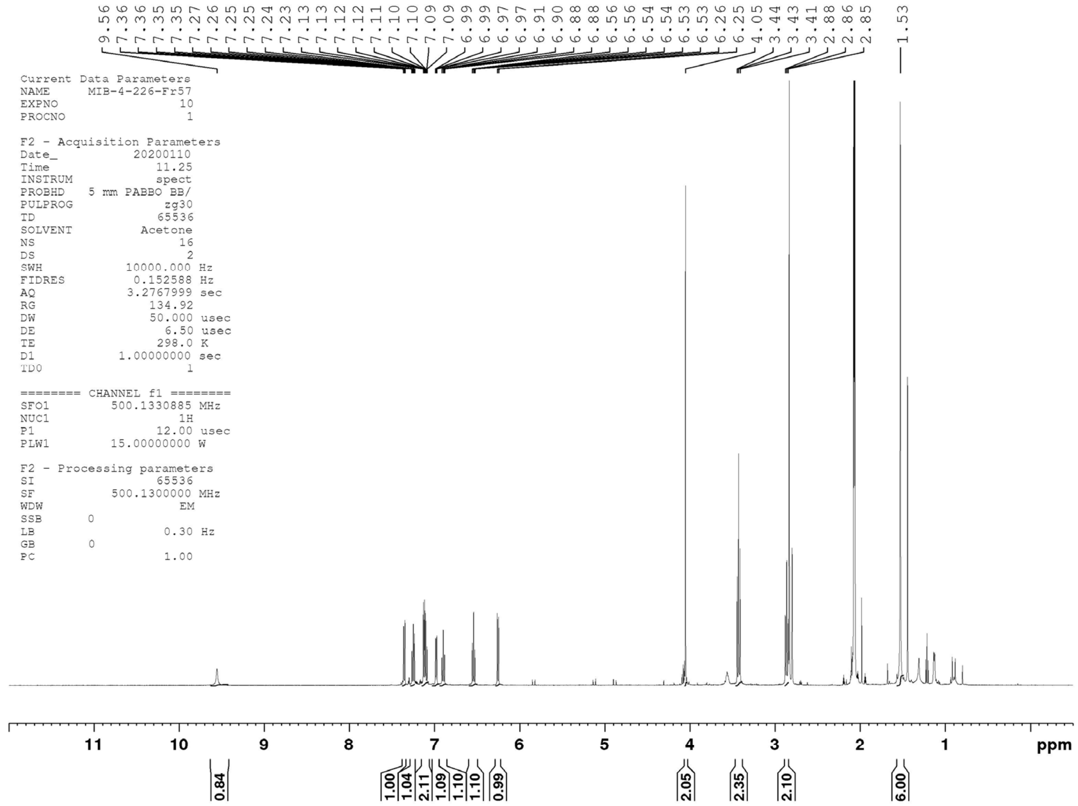

^{13}C-NMR

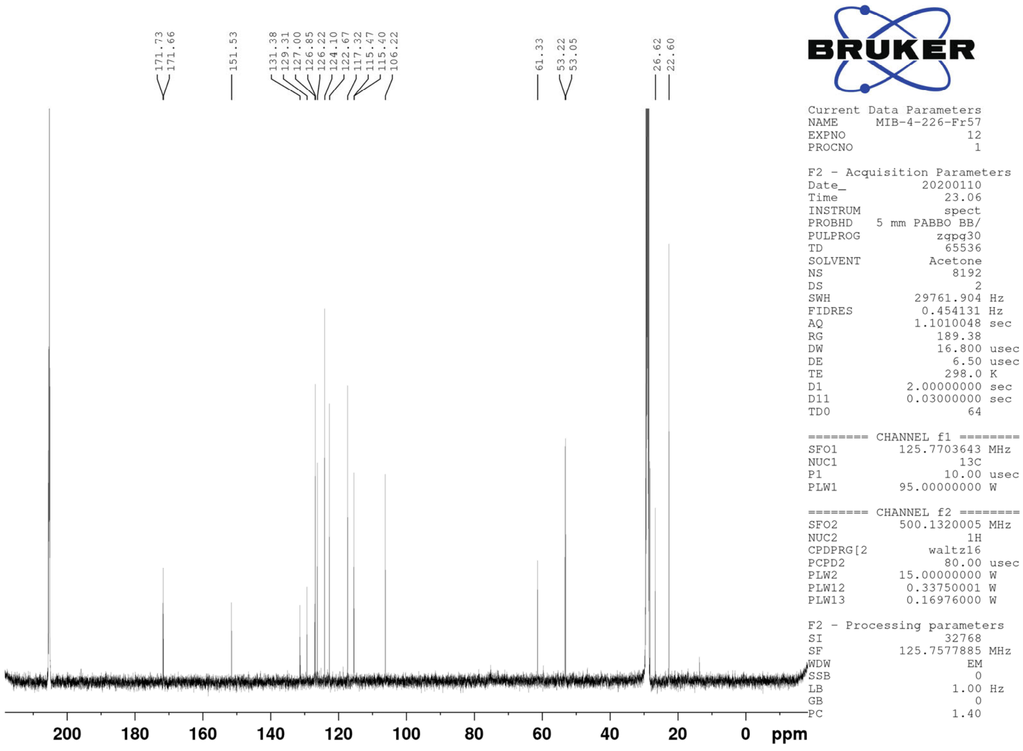

LC

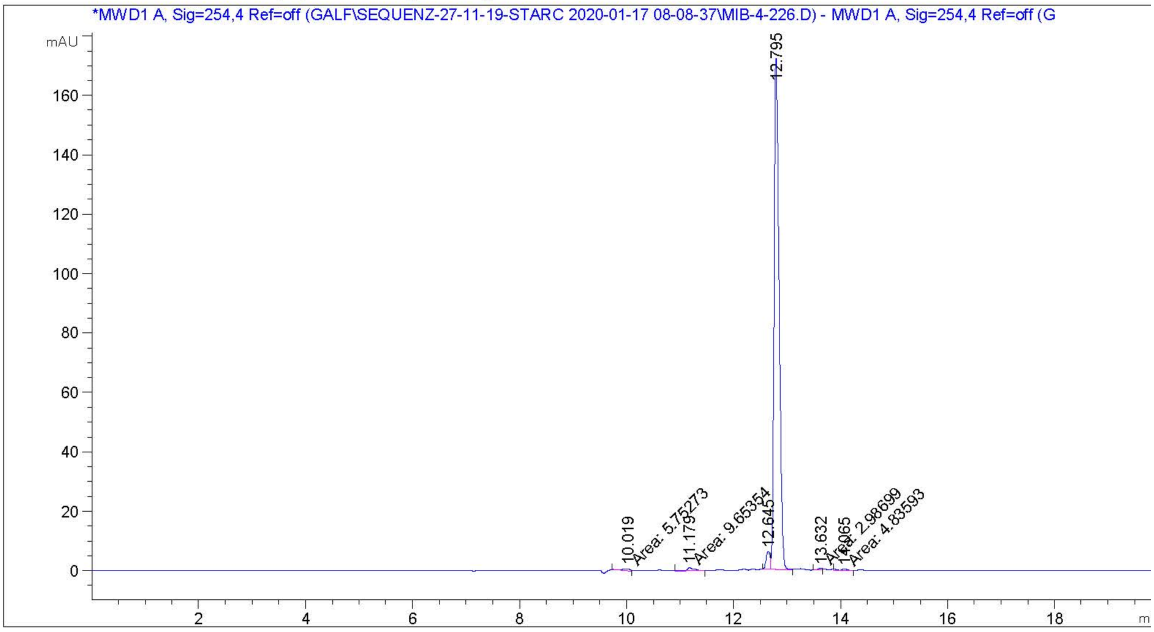


```
=====================================================================
                         Area Percent Report
=====================================================================

Sorted By             :      Signal
Multiplier            :      1.0000
Dilution              :      1.0000
Use Multiplier & Dilution Factor with ISTDs


Signal 1: MWD1 A, Sig=254,4 Ref=off
 Signal has been modified after loading from rawdata file!
```

Peak #	RetTime [min]	Type	Width [min]	Area [mAU*s]	Height [mAU]	Area %
1	10.019	MM	0.1493	5.75273	6.42100e-1	0.4942
2	11.179	MM	0.1706	9.65354	9.43049e-1	0.8292
3	12.645	BV	0.0792	30.15967	5.94530	2.5907
4	12.795	VV	0.1031	1110.74243	172.51054	95.4138
5	13.632	MM	0.0877	2.98699	5.67707e-1	0.2566
6	14.065	MM	0.1874	4.83593	4.29990e-1	0.4154

Compound S5

^{1}H-NMR

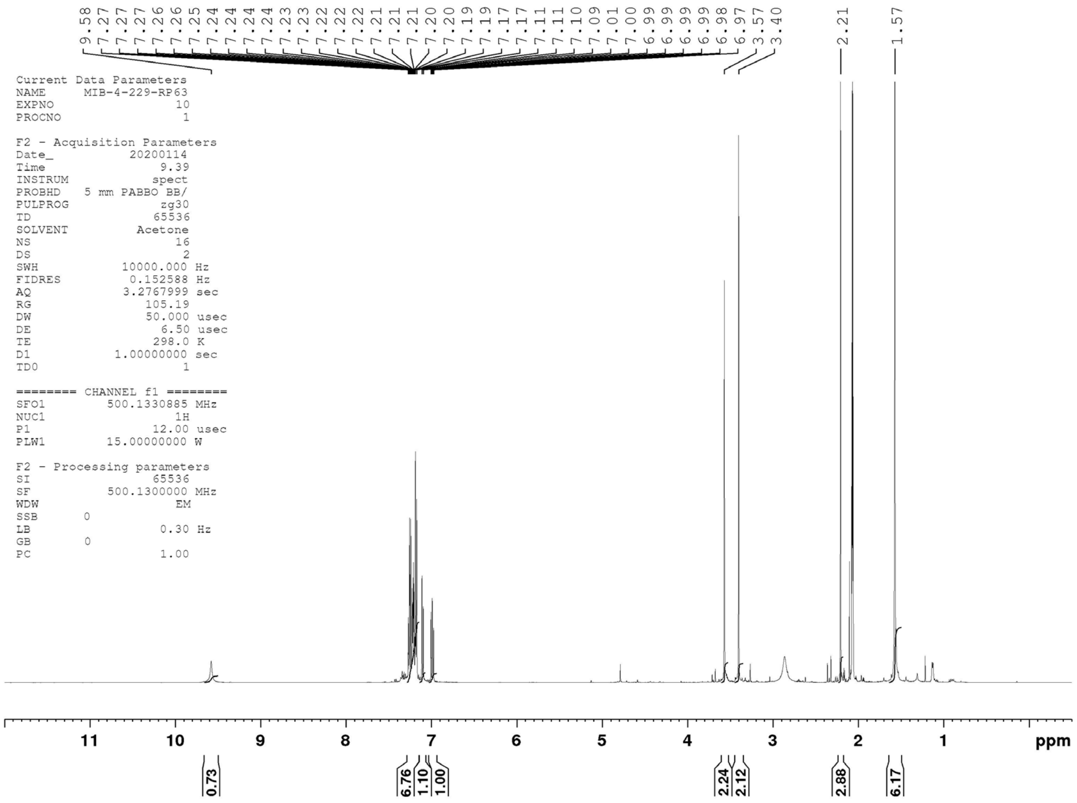

^{13}C-NMR

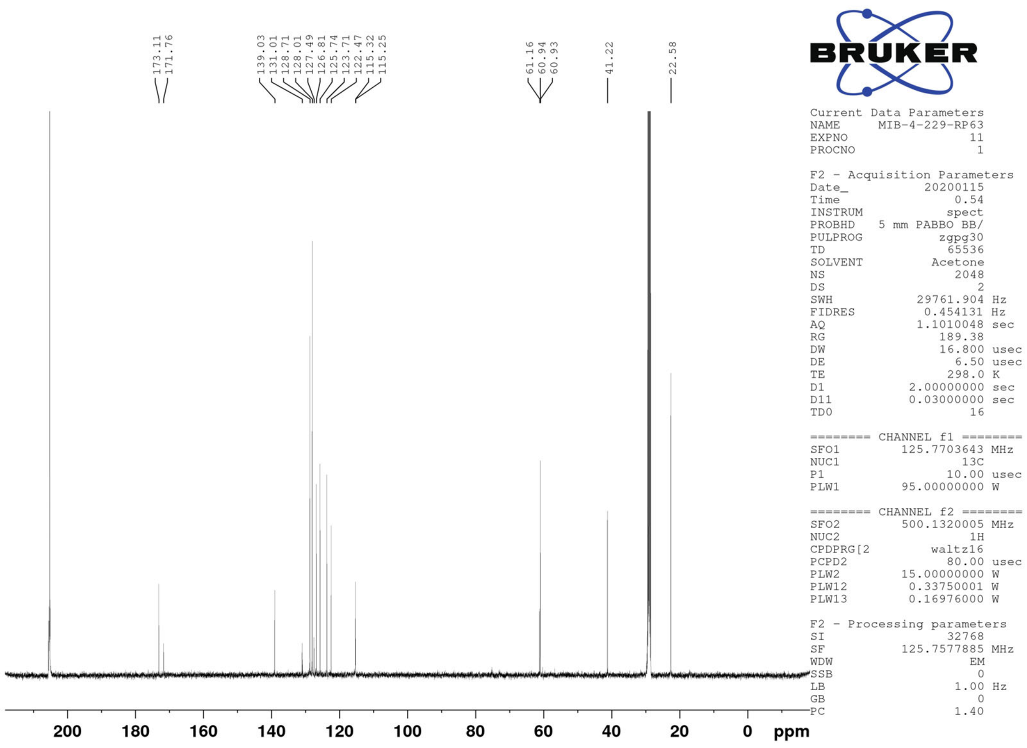

LC

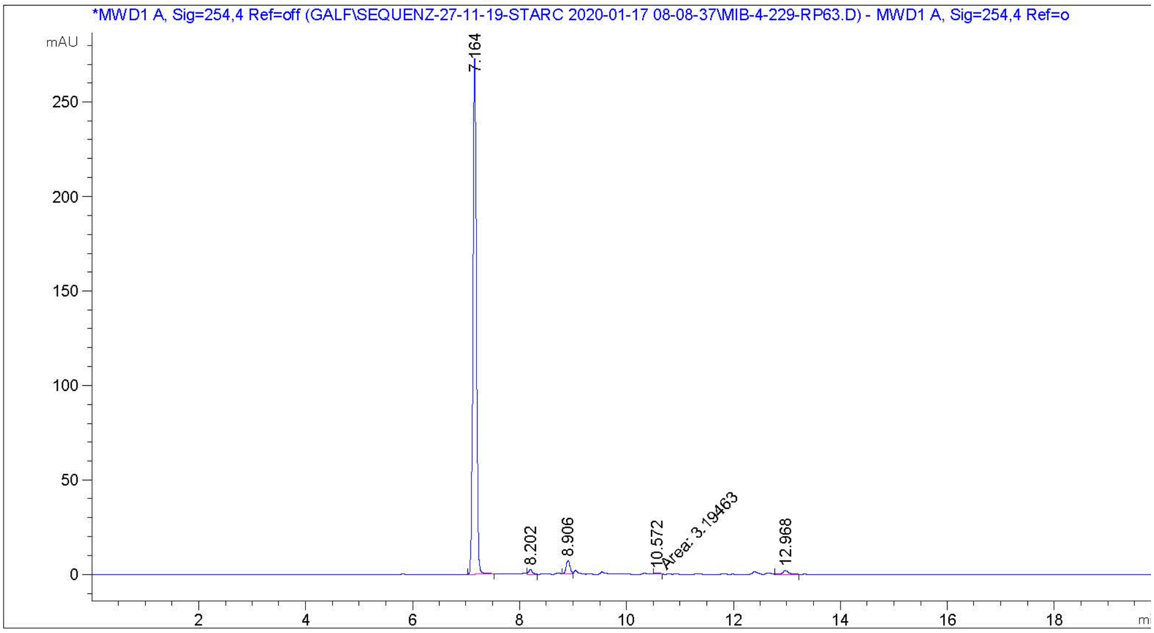


```
=====================================================================
                         Area Percent Report
=====================================================================

Sorted By             :      Signal
Multiplier            :      1.0000
Dilution              :      1.0000
Use Multiplier & Dilution Factor with ISTDs


Signal 1: MWD1 A, Sig=254,4 Ref=off
 Signal has been modified after loading from rawdata file!
```

Peak #	RetTime [min]	Type	Width [min]	Area [mAU*s]	Height [mAU]	Area %
1	7.164	BB	0.0682	1226.65686	273.96463	95.0209
2	8.202	VB	0.0727	11.47877	2.45046	0.8892
3	8.906	BV	0.0747	34.56721	7.10991	2.6777
4	10.572	MM	0.0875	3.19463	6.08688e-1	0.2475
5	12.968	VB	0.1181	15.03613	1.90542	1.1647

Compound S6

^{1}H-NMR

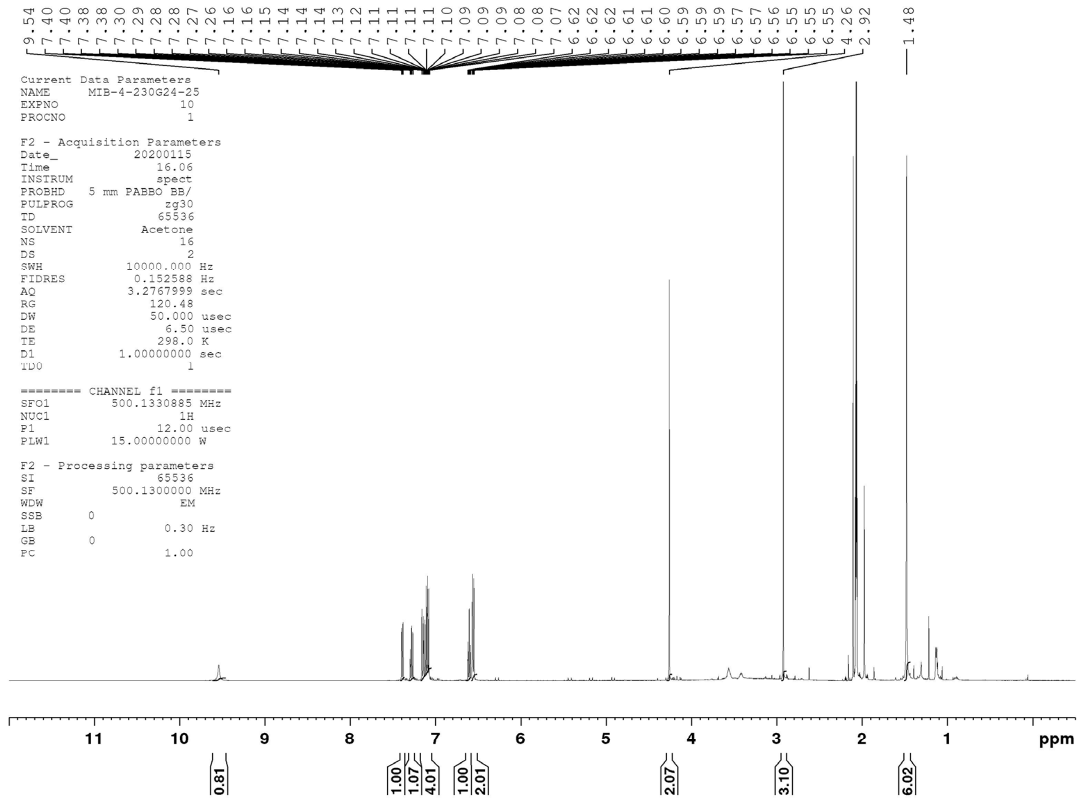

^{13}C-NMR

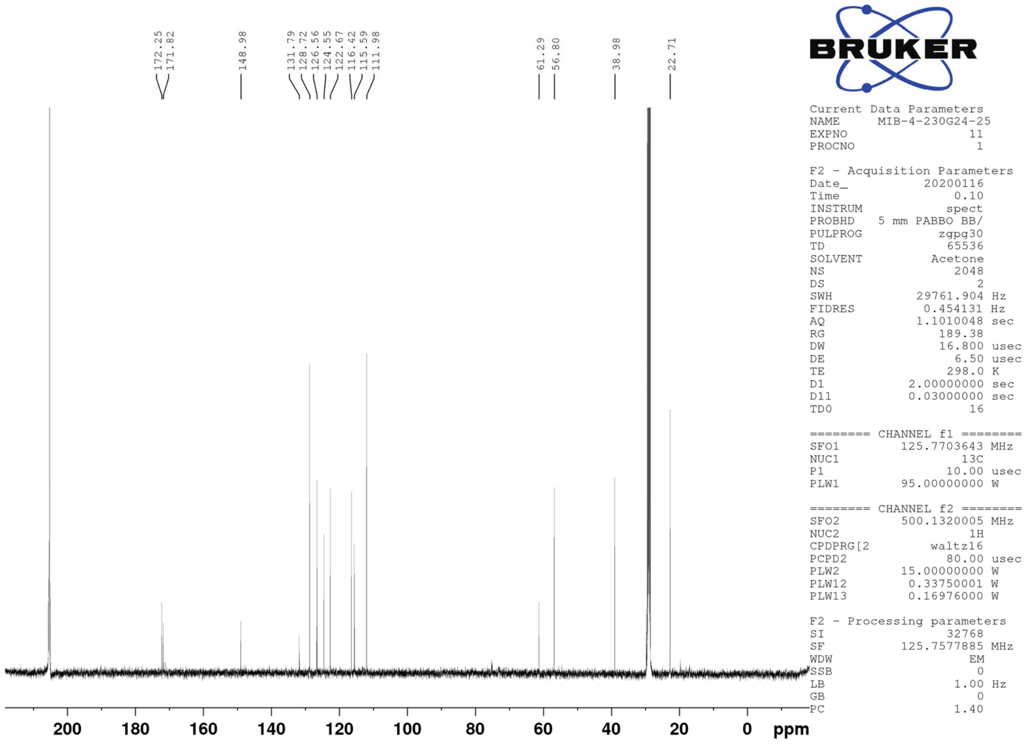

LC

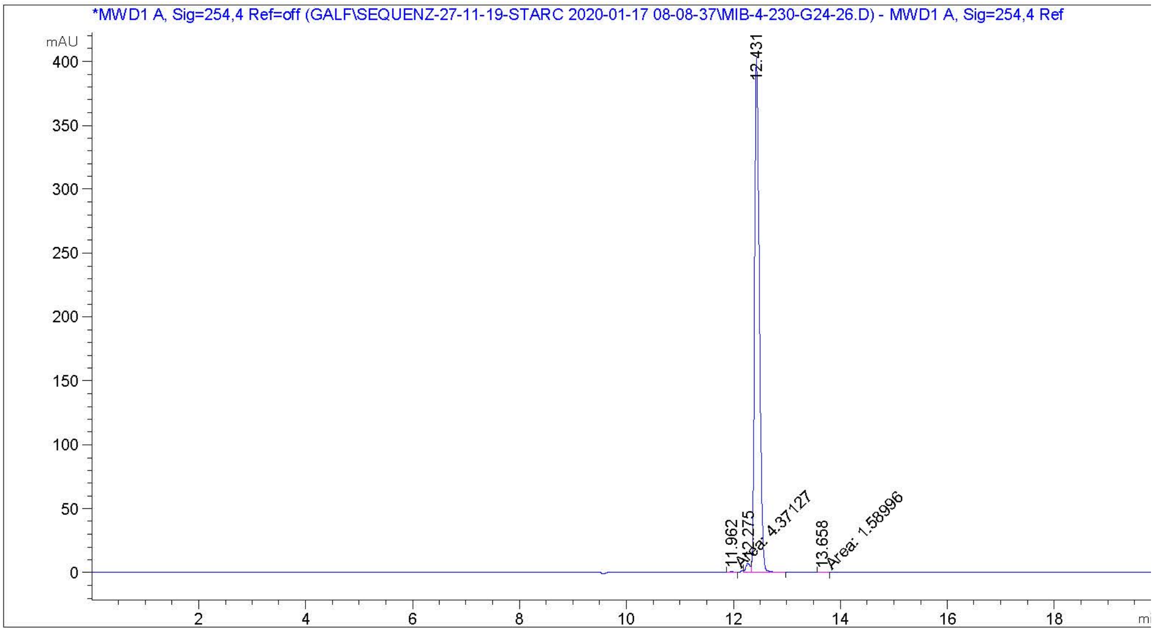


```
=====================================================================
                          Area Percent Report
=====================================================================

Sorted By             :      Signal
Multiplier            :      1.0000
Dilution              :      1.0000
Use Multiplier & Dilution Factor with ISTDs


Signal 1: MWD1 A, Sig=254,4 Ref=off
 Signal has been modified after loading from rawdata file!

Peak RetTime Type  Width     Area      Height     Area
  #   [min]        [min]   [mAU*s]     [mAU]        %
----|-------|----|-------|----------|----------|--------|
   1  11.962 MM    0.1017    4.37127 7.16553e-1   0.1694
   2  12.275 VV    0.0831   37.99773    7.02580   1.4728
   3  12.431 VB    0.0994 2536.02856  403.13416  98.2962
   4  13.658 MM    0.1413    1.58996 1.87573e-1   0.0616
```

Compound S7

^{1}H-NMR

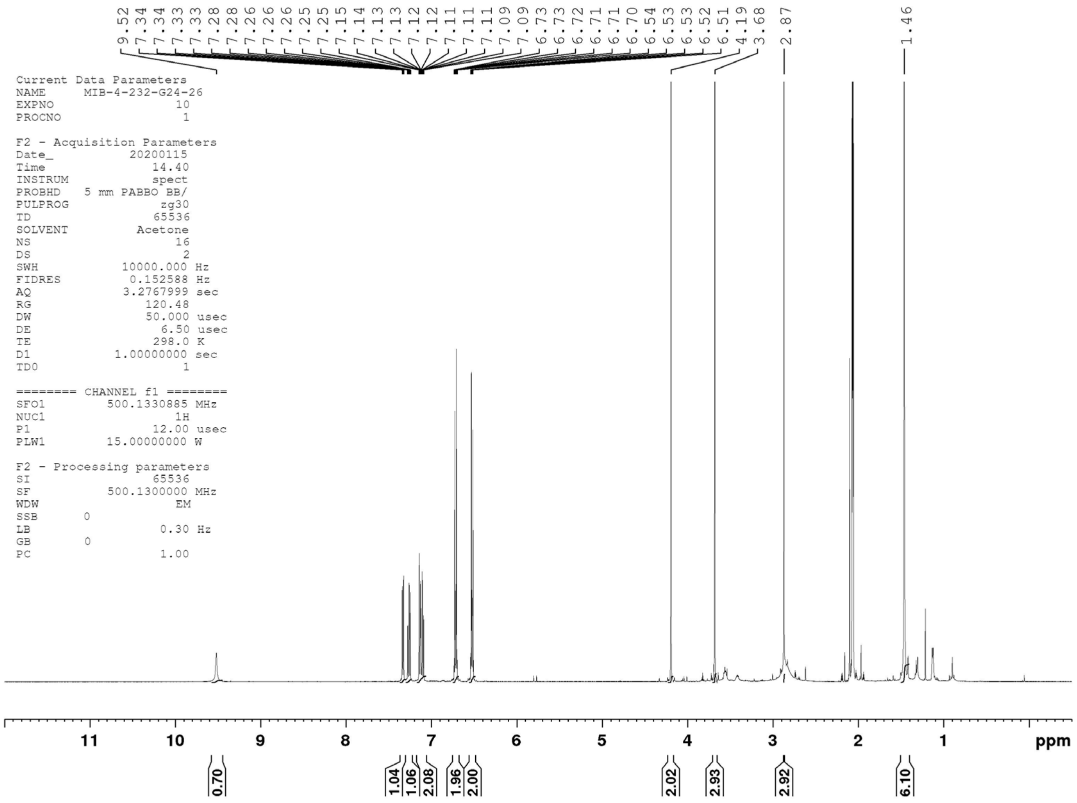

^{13}C-NMR

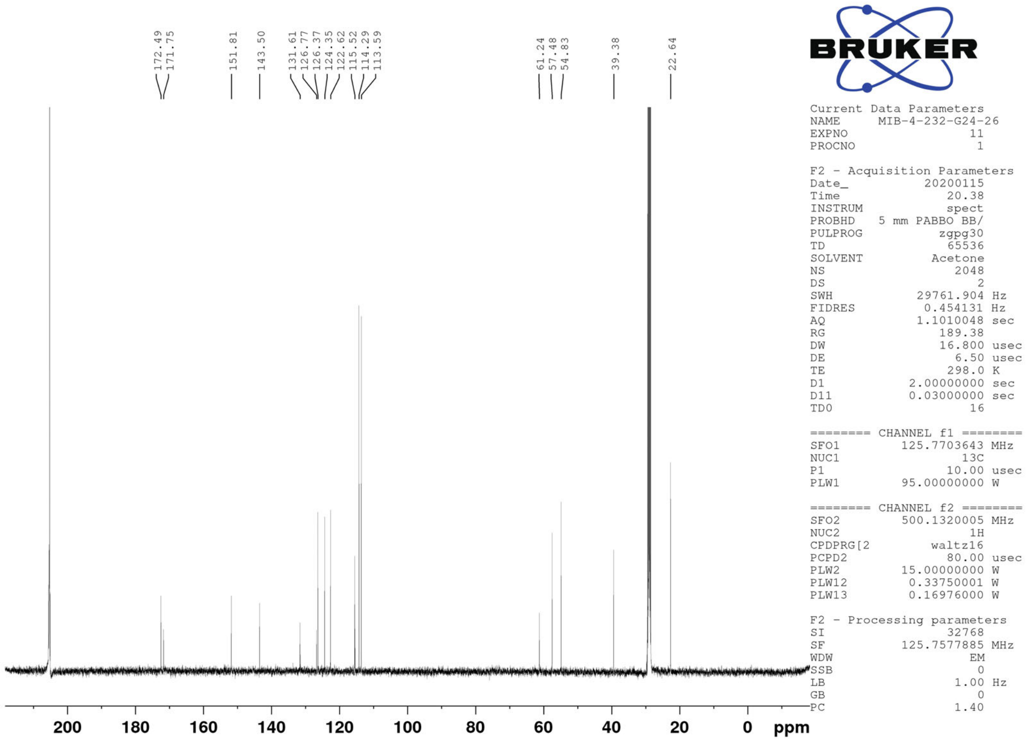

LC

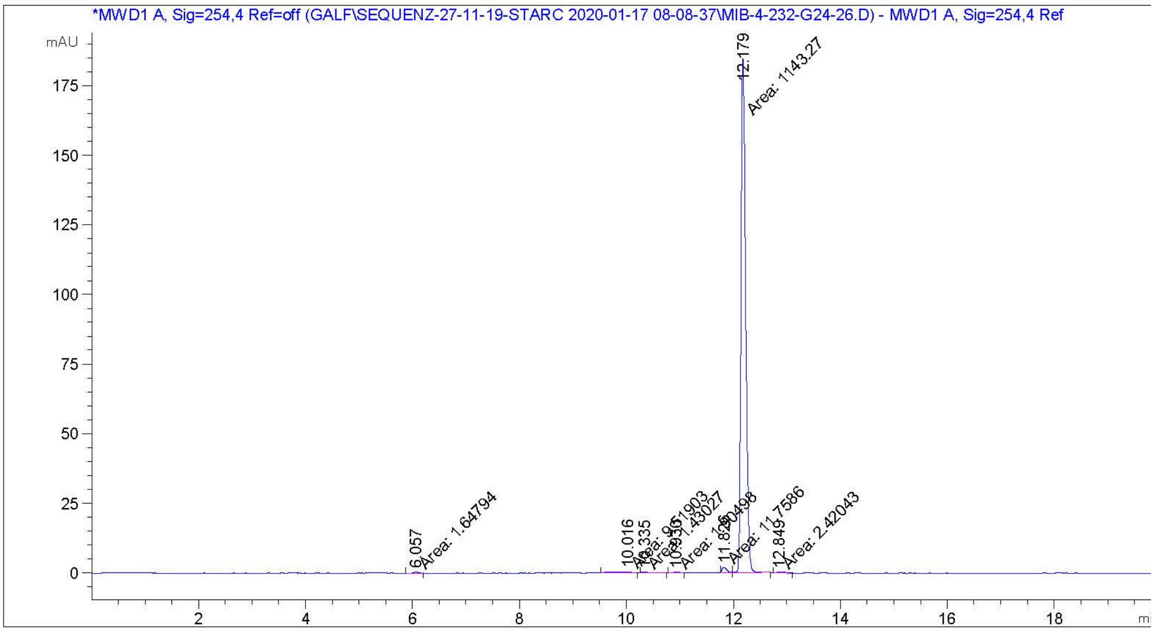


```
=====================================================================
                       Area Percent Report
=====================================================================

Sorted By             :      Signal
Multiplier            :      1.0000
Dilution              :      1.0000
Use Multiplier & Dilution Factor with ISTDs


Signal 1: MWD1 A, Sig=254,4 Ref=off
 Signal has been modified after loading from rawdata file!
```

Peak #	RetTime [min]	Type	Width [min]	Area [mAU*s]	Height [mAU]	Area %
1	6.057	MM	0.0964	1.64794	2.84888e-1	0.1406
2	10.016	MM	0.3419	9.51903	4.64042e-1	0.8122
3	10.335	MM	0.1199	1.43027	1.98747e-1	0.1220
4	10.930	MM	0.1140	1.90498	2.78574e-1	0.1625
5	11.826	MF	0.1023	11.75859	1.91577	1.0033
6	12.179	FM	0.1028	1143.27441	185.28415	97.5527
7	12.849	MM	0.1930	2.42043	2.09038e-1	0.2065

Compound S9

^{1}H-NMR

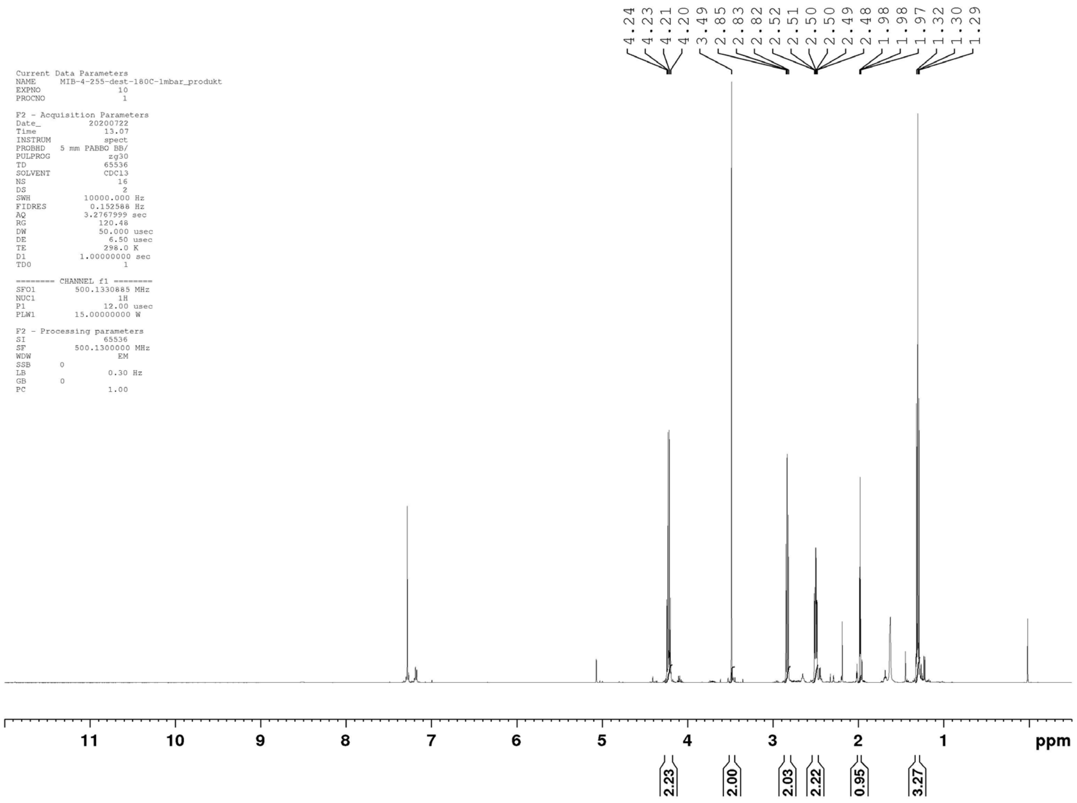

^{13}C-NMR

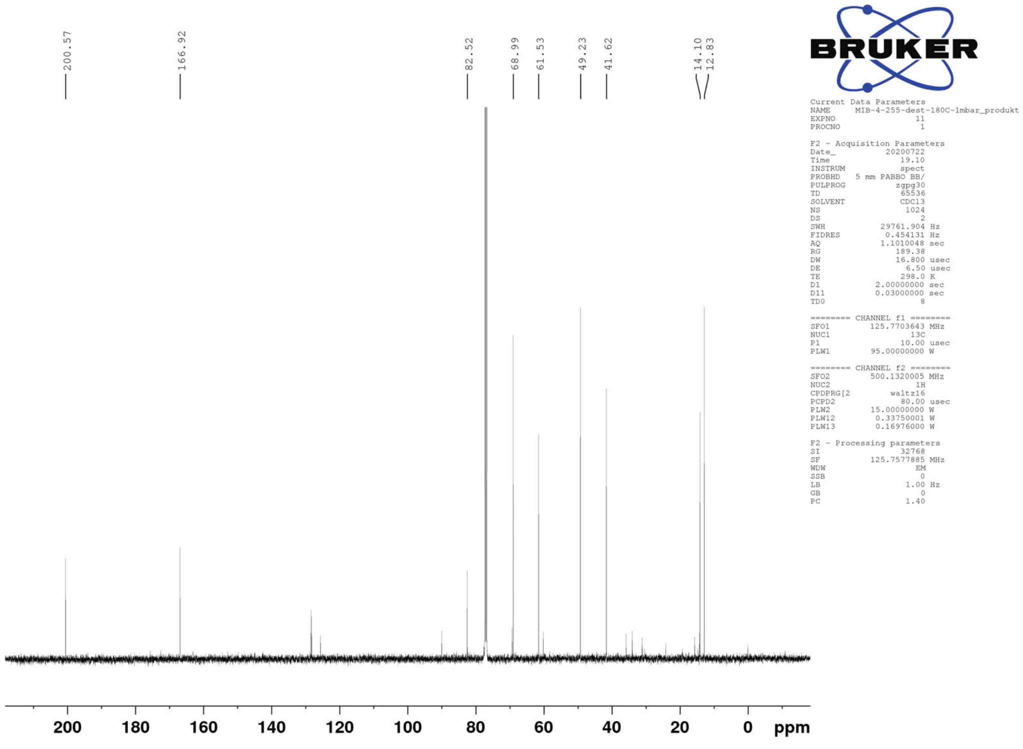

Compound S10

^{1}H-NMR

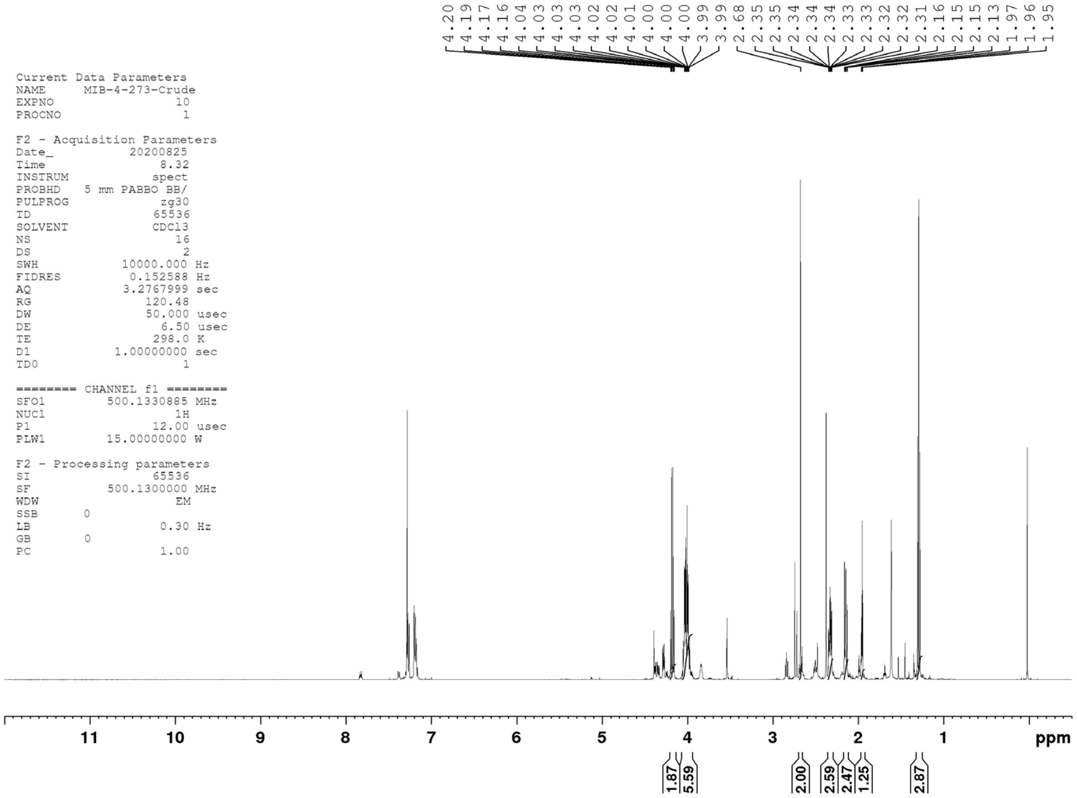

Compound S13

1H-NMR

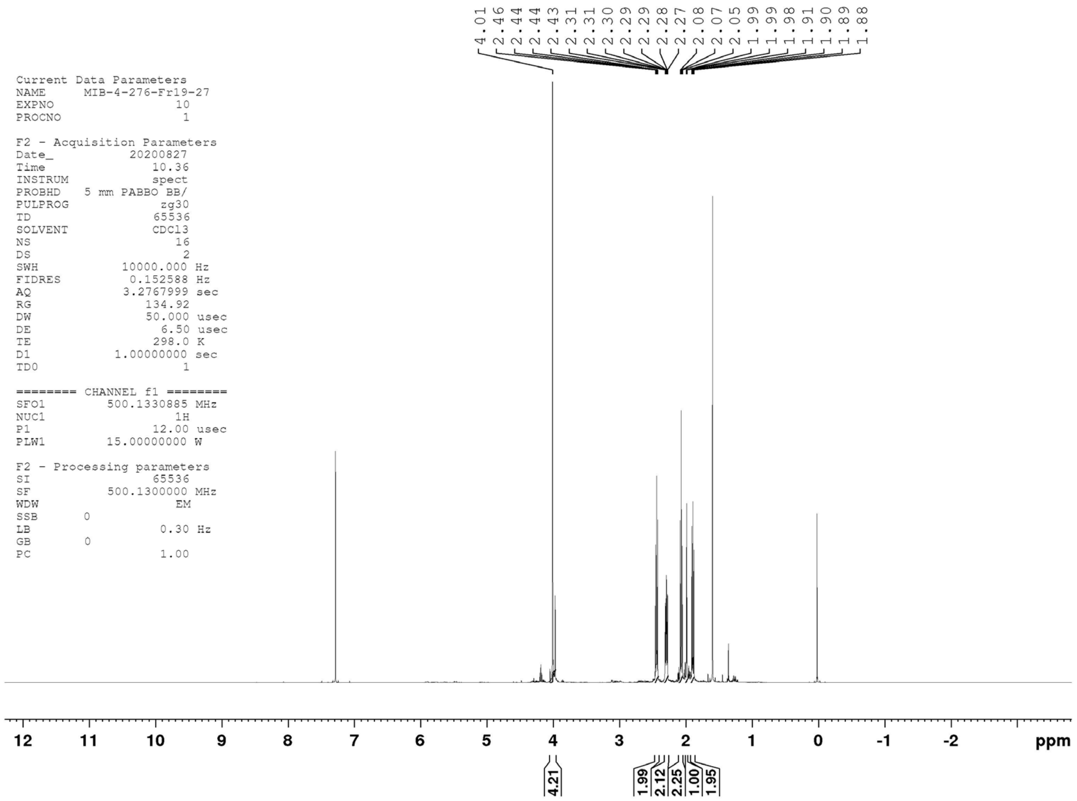

13C-NMR

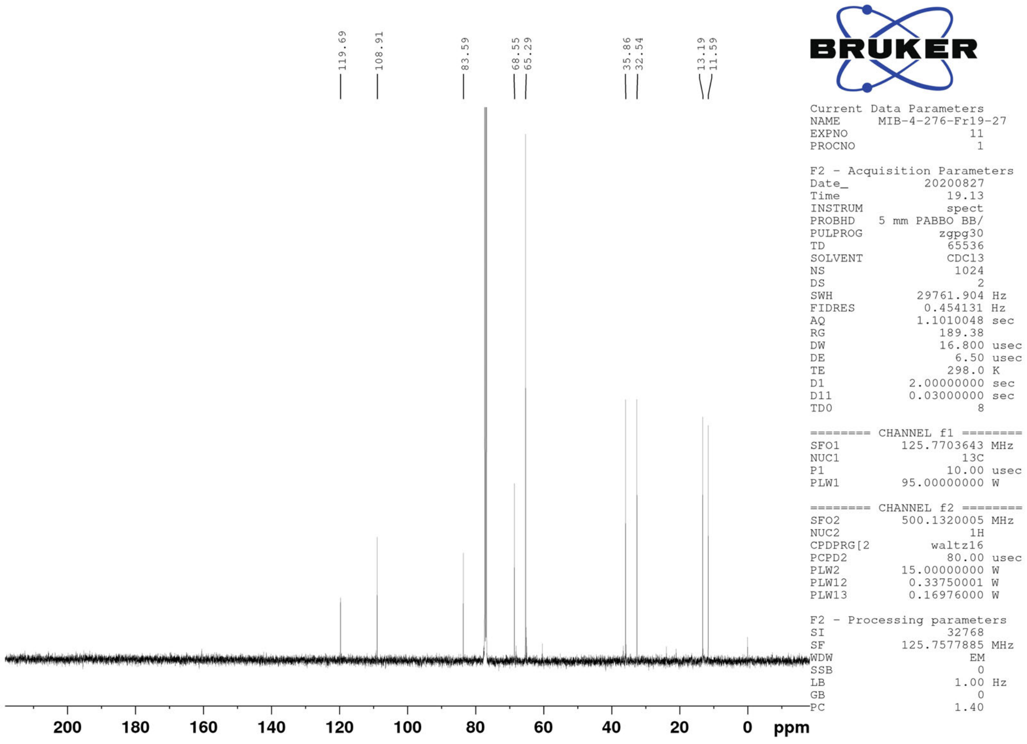

Compound S14

¹H-NMR

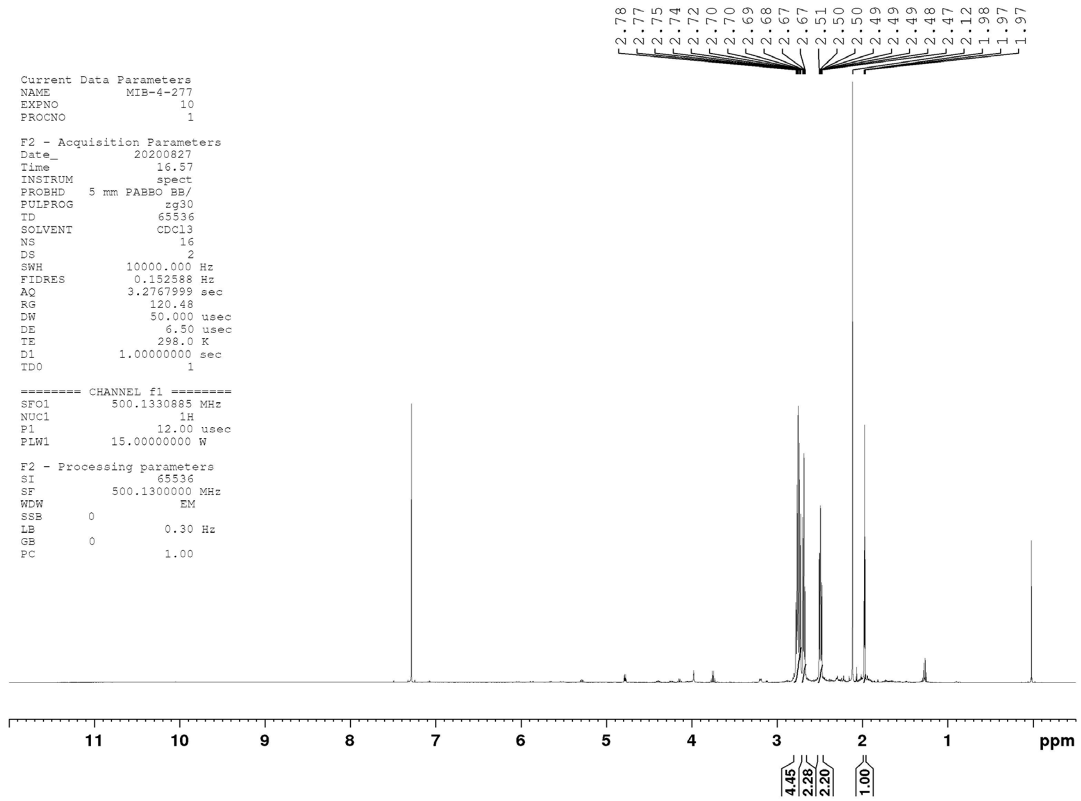

¹³C-NMR

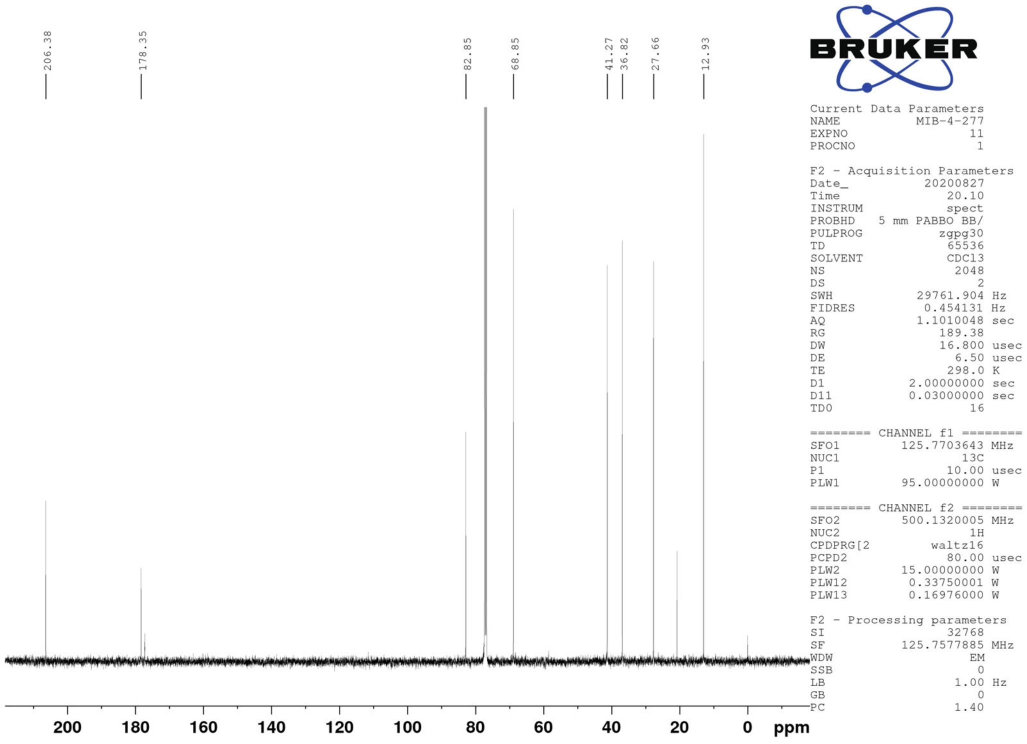

Compound S15

^{1}H-NMR

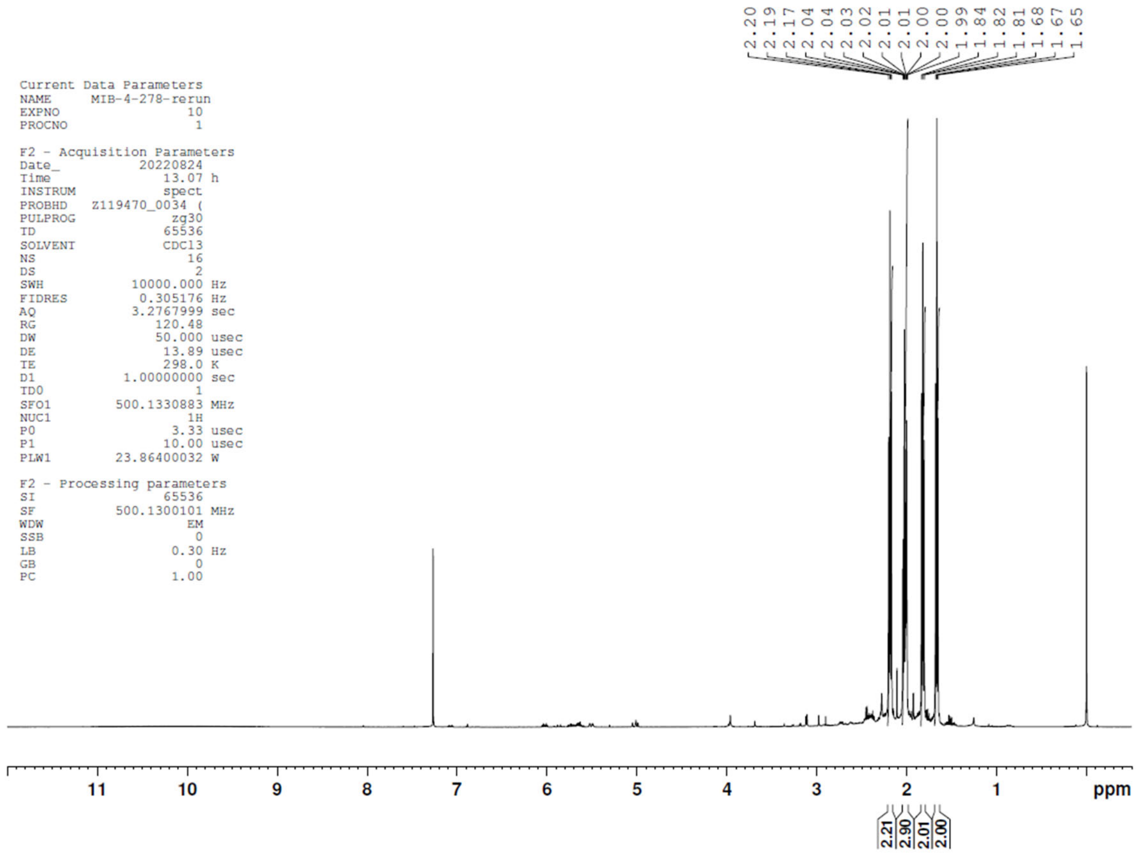

^{13}C-NMR

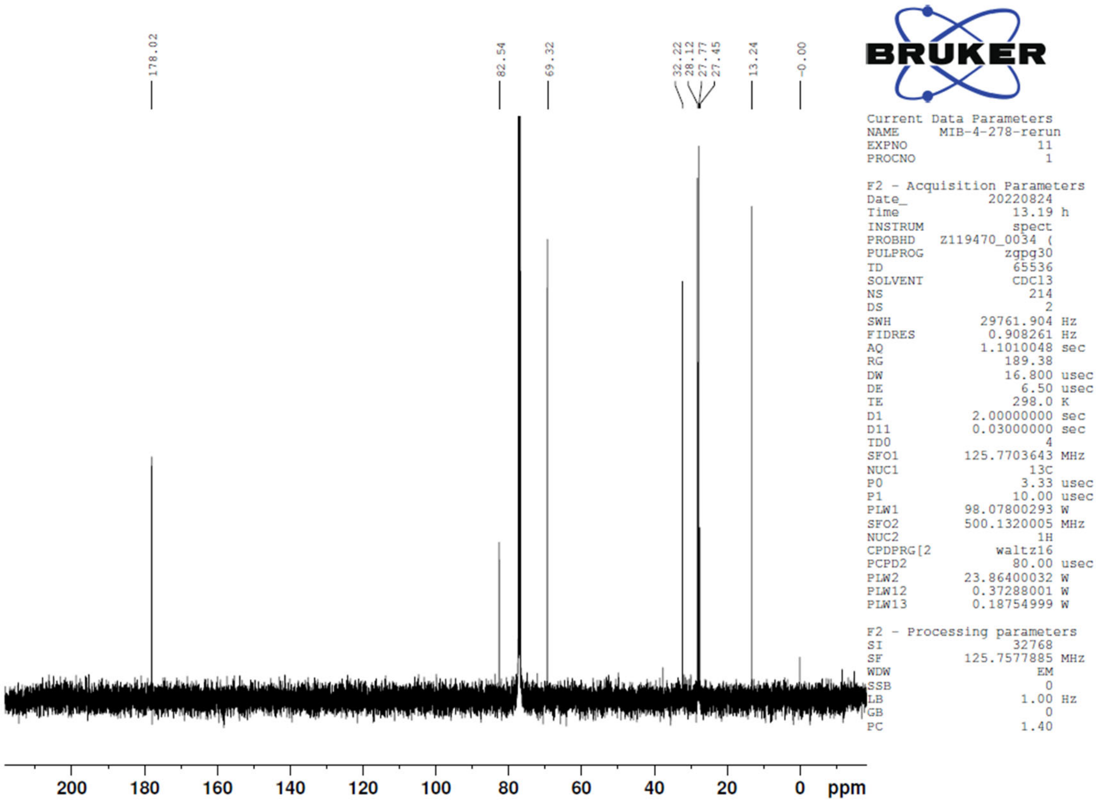

3.2. Biological assay results

3.2.1. SENP1-SUMO1-AMC

Compound 11 screening library

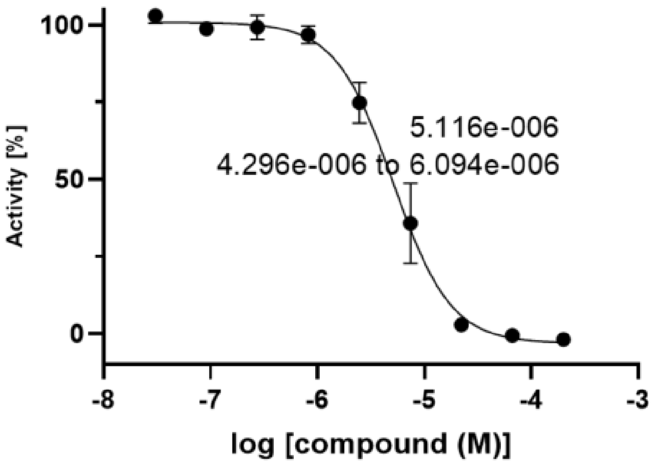

Conc log[M]	%	%
-3.70	-1.5	-2.4
-4.18	-0.9	-0.3
-4.65	2.0	3.8
-5.13	44.9	26.6
-5.61	79.5	70.0
-6.08	98.8	94.7
-6.56	102.0	96.4
-7.04	99.4	98.0
DMSO	104.6	101.3

Compound 11 synthesised

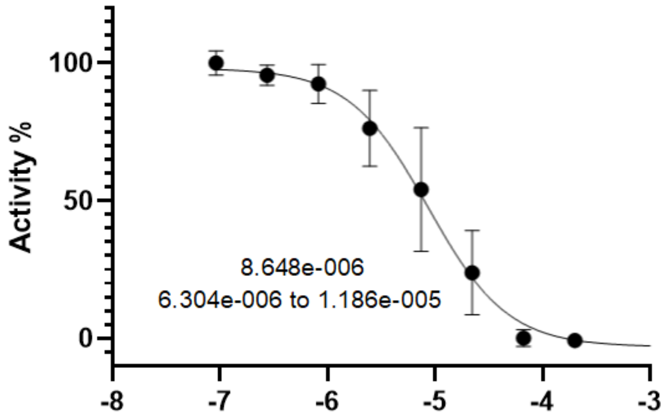

Compound 12

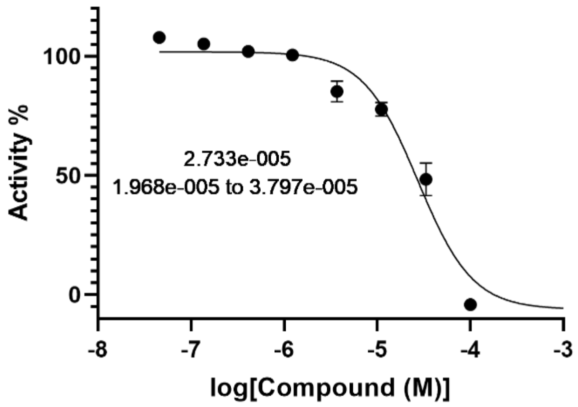

Conc log[M]	%	%
-3.82	-3.7	-4.7
-4.30	53.3	43.5
-4.78	79.7	75.7
-5.26	88.3	82.0
-5.73	100.2	100.7
-6.21	103.6	100.4
-6.69	104.4	105.8
-7.16	107.7	108.0
DMSO	97.3	97.3

Compound 13

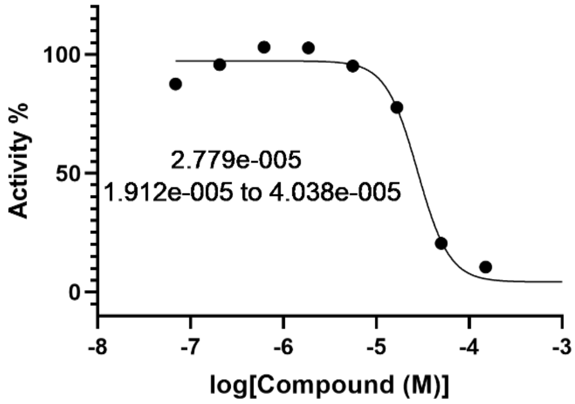

Conc log[M]	%
-3.82	10.6
-4.30	20.5
-4.78	77.7
-5.26	95.1
-5.73	102.7
-6.21	103.1
-6.69	95.7
-7.16	87.6
DMSO	97.3

Compound 23

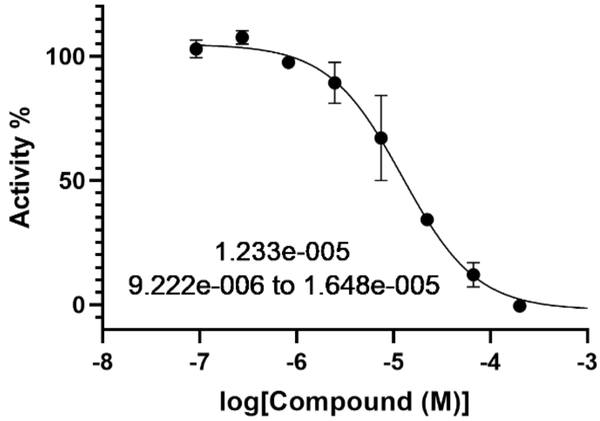

Conc log[M]	%	%
-3.70	-0.8	-0.3
-4.18	15.5	8.5
-4.65	34.5	34.1
-5.13	79.2	55.0
-5.61	95.2	83.5
-6.08	98.8	96.2
-6.56	109.5	105.7
-7.04	105.5	100.4
DMSO	99.3	100.6

Compound 24

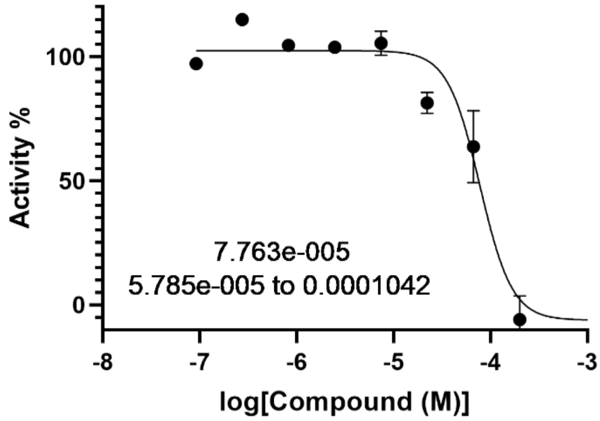

Conc log[M]	%	%
-3.70	-12.7	0.8
-4.18	74.0	53.4
-4.65	84.4	78.4
-5.13	108.9	102.0
-5.61	102.8	104.8
-6.08	103.0	106.1
-6.56	115.5	114.3
-7.04	98.0	96.4
DMSO	111.3	105.3

Fragments

Compound 15

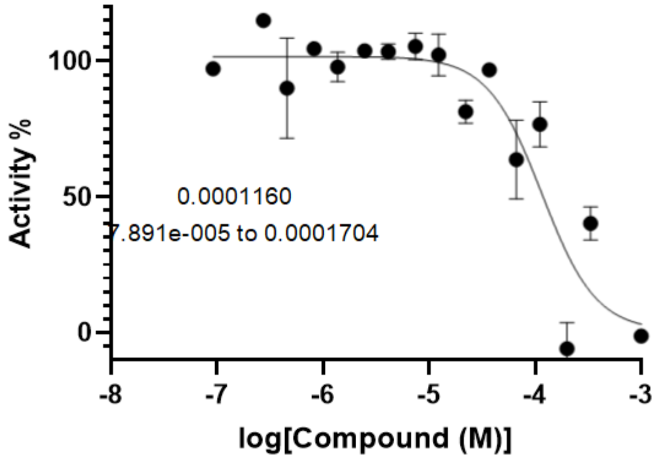

Compound 16

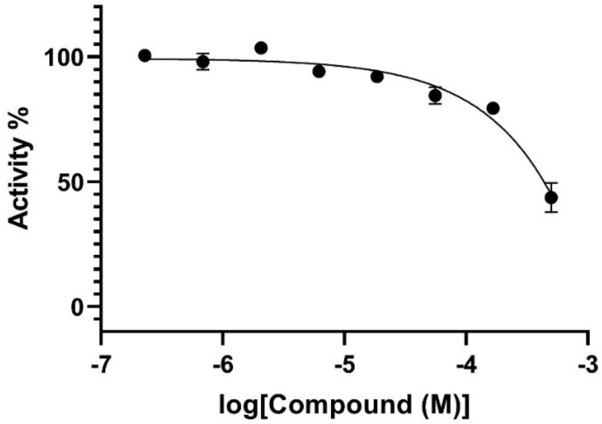

Conc log[M]	%	%
-3.30	39.6	47.8
-3.78	78.3	80.6
-4.26	82.2	86.9
-4.73	92.1	92.1
-5.21	95.1	93.3
-5.69	104.9	102.3
-6.16	100.4	95.7
-6.64	144.5	100.6
DMSO	103.2	102.1

Compound 26

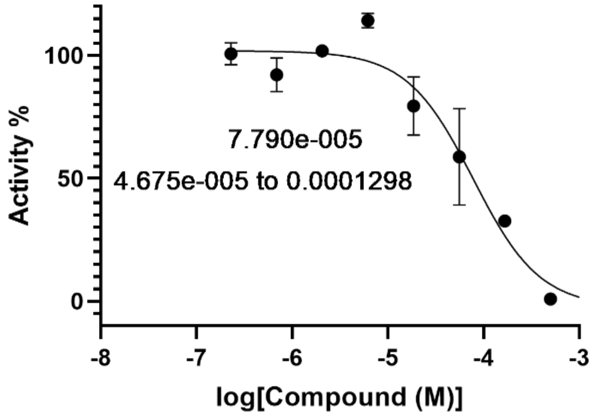

Conc log[M]	%	%
-3.30	0.4	1.3
-3.78	31.0	34.3
-4.26	44.8	72.6
-4.73	71.0	87.8
-5.21	116.2	112.1
-5.69	101.7	102.1
-6.16	97.0	87.3
-6.64	103.8	97.5
DMSO	107.2	104.3

Compound 27

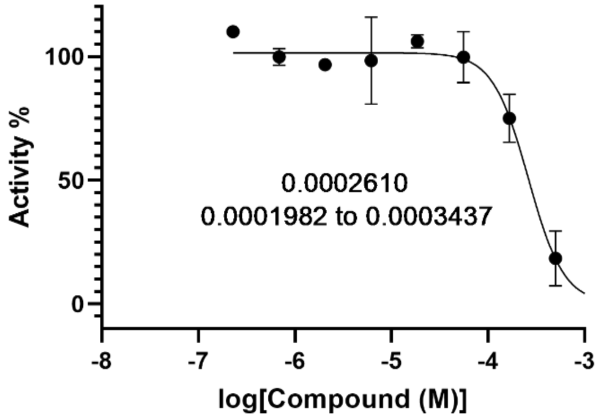

Conc log[M]	%	%
-3.30	26.3	10.6
-3.78	81.8	68.1
-4.26	107.0	92.4
-4.73	107.9	104.3
-5.21	85.9	110.7
-5.69	95.8	97.5
-6.16	97.5	102.2
-6.64	110.0	76.9
DMSO	102.3	106.9

data point omitted

Compound 28

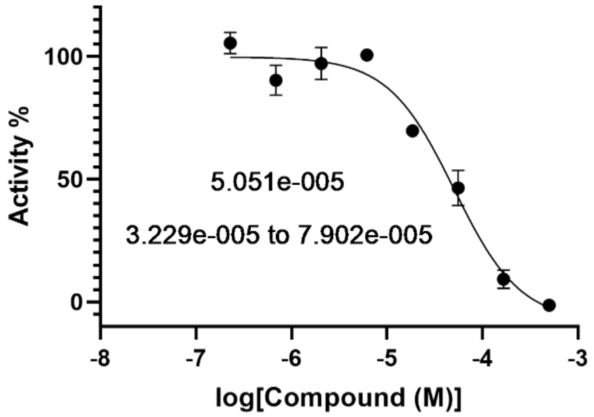

Conc log[M]	%	%
-3.30	-0.2	-2.7
-3.78	11.9	6.7
-4.26	51.5	41.3
-4.73	69.2	70.1
-5.21	98.8	102.4
-5.69	101.7	92.5
-6.16	94.6	86.0
-6.64	102.3	108.5
DMSO	93.2	104.7

Compound 29

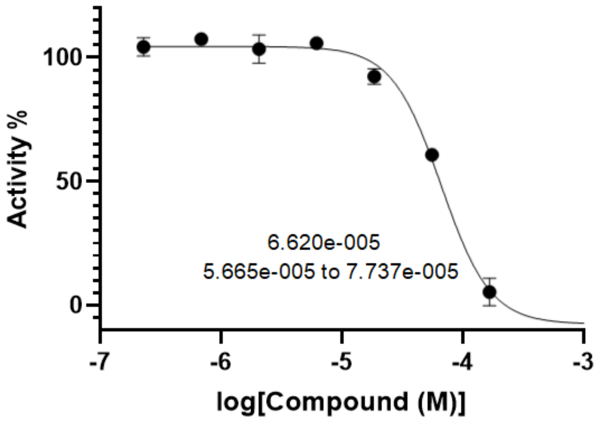

Conc log[M]	%	%
-3.30	-12.7	-14.7
-3.78	1.4	9.2
-4.26	60.7	60.7
-4.73	90.1	94.6
-5.21	104.6	106.8
-5.69	99.3	107.4
-6.16	106.1	108.6
-6.64	101.6	106.9
DMSO	105.3	102.8

Compound 30

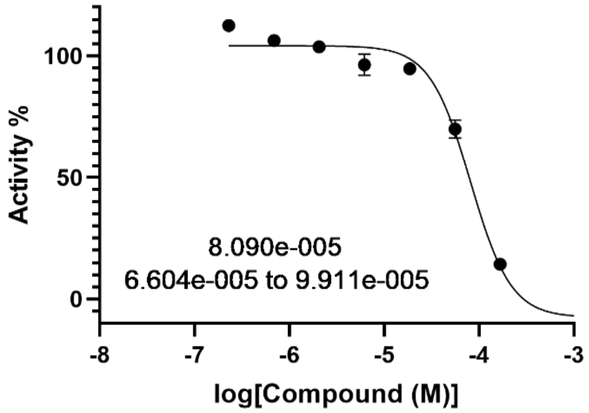

Conc log[M]	%	%
-3.30	-12.6	-14.7
-3.78	15.1	13.6
-4.26	72.5	67.2
-4.73	94.1	95.4
-5.21	99.4	93.2
-5.69	103.5	103.8
-6.16	106.3	106.4
-6.64	112.7	112.2
DMSO	107.4	99.6

Compound 31

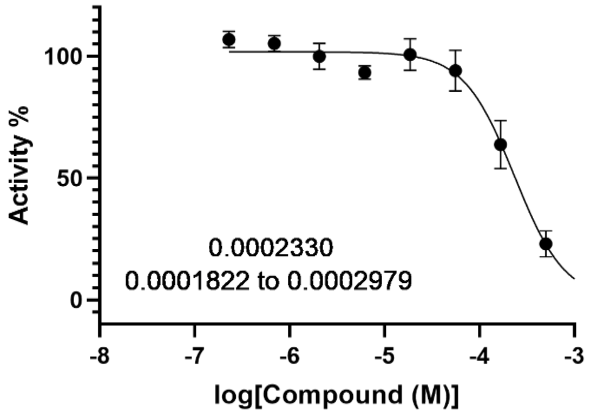

Conc log[M]	%	%
-3.30	19.3	26.8
-3.78	56.7	70.7
-4.26	88.1	100.0
-4.73	96.0	105.2
-5.21	91.3	95.2
-5.69	96.2	103.6
-6.16	102.9	107.6
-6.64	104.5	109.2
DMSO	100.0	112.3

Compound 32

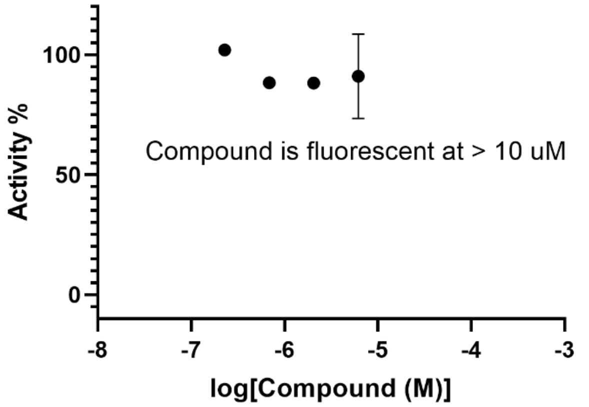

Conc log[M]	%	%
-3.30	fluorescent	
-3.78	fluorescent	
-4.26	fluorescent	
-4.73	fluorescent	
-5.21	103.5	78.5
-5.69	89.3	87.0
-6.16	90.0	86.7
-6.64	103.3	100.5
DMSO	107.4	99.6

Compound 33

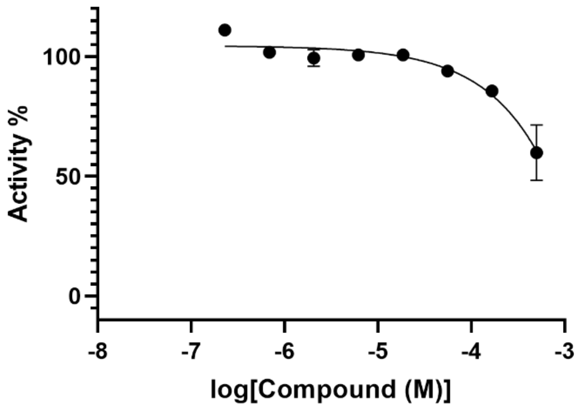

Conc log[M]	%	%
-3.30	51.6	68.1
-3.78	83.9	87.3
-4.26	92.1	95.7
-4.73	99.4	101.9
-5.21	101.2	100.1
-5.69	101.8	96.9
-6.16	101.7	101.9
-6.64	110.1	112.1
DMSO	103.4	99.2

Compound 34

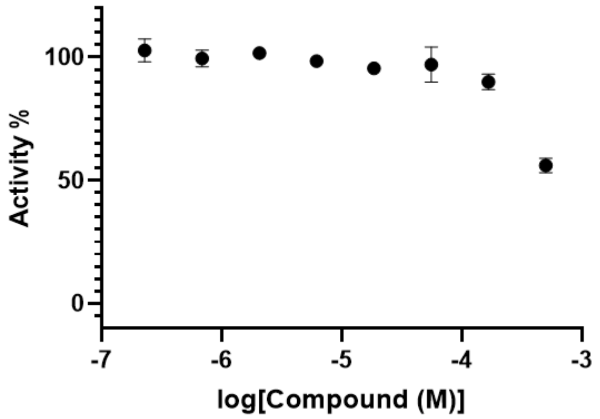

Conc log[M]	%	%
-3.30	58.1	54.0
-3.78	87.6	92.2
-4.26	91.9	102.1
-4.73	95.9	95.0
-5.21	98.1	98.7
-5.69	103.1	100.1
-6.16	97.1	101.9
-6.64	106.1	99.4
DMSO	97.3	103.7

Compound 35

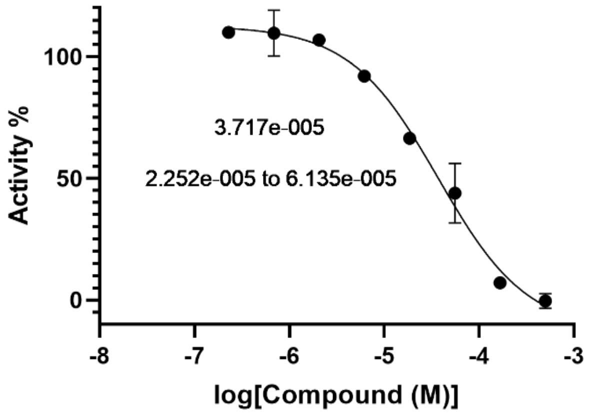

Conc log[M]	%	%
-3.30	1.8	-2.6
-3.78	8.0	6.2
-4.26	52.6	35.2
-4.73	66.5	66.3
-5.21	32.1	92.0
-5.69	108.6	105.0
-6.16	116.3	102.9
-6.64	110.0	68.9
DMSO	112.3	103.4

data point omitted

Compound 36

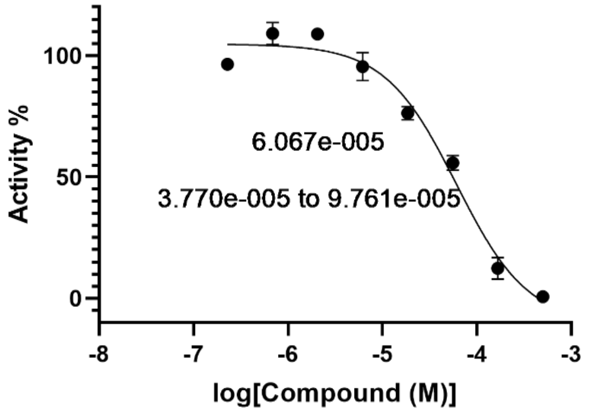

Conc log[M]	%	%
-3.30	-0.5	1.9
-3.78	9.3	15.5
-4.26	58.0	53.7
-4.73	74.4	78.2
-5.21	99.5	91.4
-5.69	108.4	109.3
-6.16	112.4	105.9
-6.64	95.3	97.5
DMSO	98.3	105.0

Compound 37

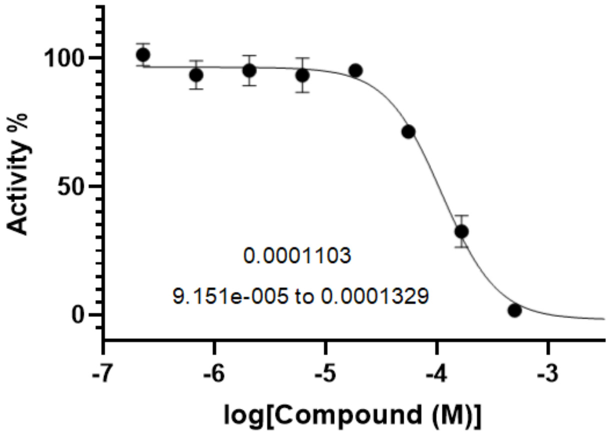

Conc log[M]	%	%
-3.30	2.4	1.1
-3.78	28.2	37.0
-4.26	69.6	73.3
-4.73	94.5	95.9
-5.21	98.1	88.8
-5.69	99.5	91.1
-6.16	97.4	89.6
-6.64	98.4	104.5
DMSO	100.2	97.8

Compound 38

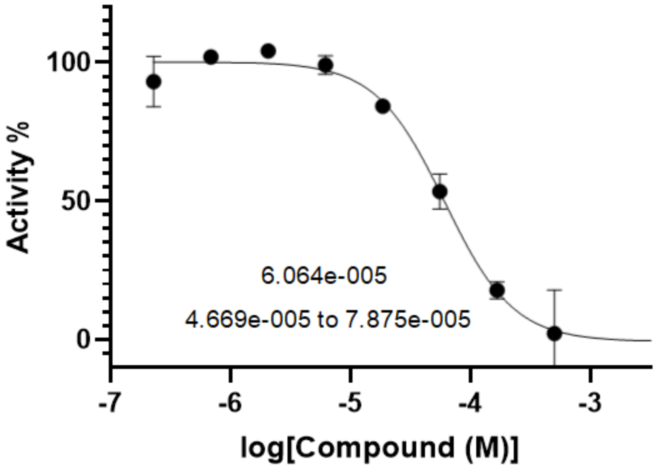

Conc log[M]	%	%
-3.30	13.3	-8.9
-3.78	19.9	15.6
-4.26	57.9	49.0
-4.73	83.1	85.3
-5.21	101.4	96.6
-5.69	104.4	103.8
-6.16	100.8	103.1
-6.64	99.5	86.7
DMSO	102.2	95.4

Compound 39

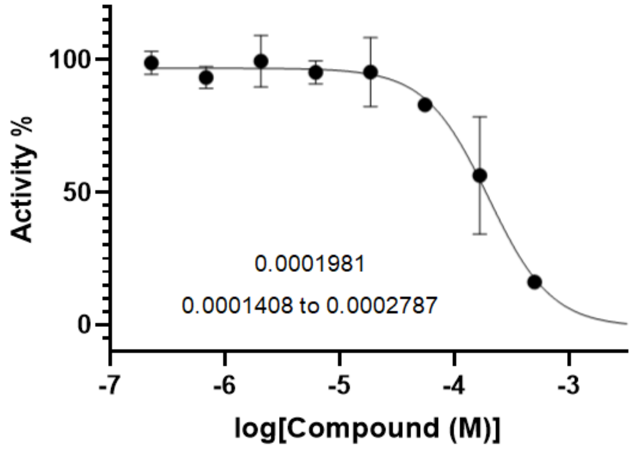

Conc log[M]	%	%
-3.30	14.4	17.9
-3.78	40.7	71.9
-4.26	84.1	81.8
-4.73	86.1	104.6
-5.21	92.2	98.4
-5.69	92.6	106.4
-6.16	90.4	96.3
-6.64	101.9	95.8
DMSO	97.5	95.4

Compound 40

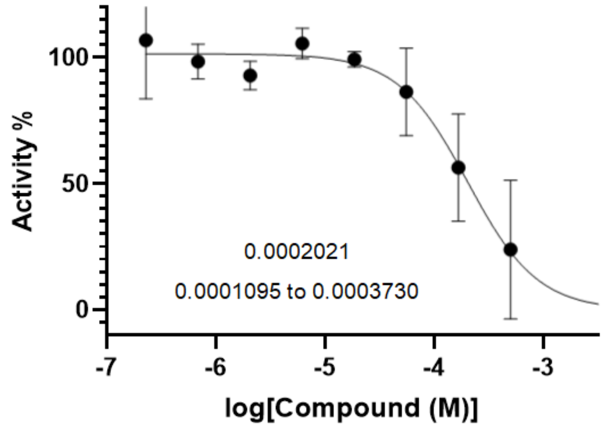

Conc log[M]	%	%
-3.30	43.3	4.4
-3.78	71.3	41.3
-4.26	98.6	74.0
-4.73	97.1	101.4
-5.21	109.8	101.3
-5.69	88.9	96.8
-6.16	103.3	93.5
-6.64	123.2	90.5
DMSO	97.5	95.4

Compound 41

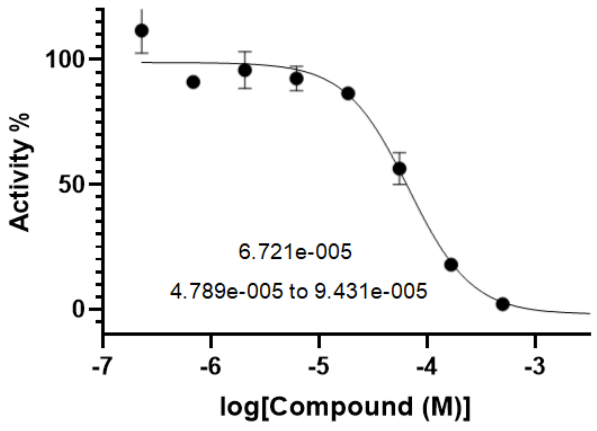

Conc log[M]	%	%
-3.30	0.9	3.3
-3.78	17.2	18.6
-4.26	51.8	60.9
-4.73	85.5	87.5
-5.21	89.0	95.9
-5.69	90.5	101.0
-6.16	92.2	89.7
-6.64	105.2	118.0
DMSO	103.2	107.5

Tetrazol modifications

Compound 44

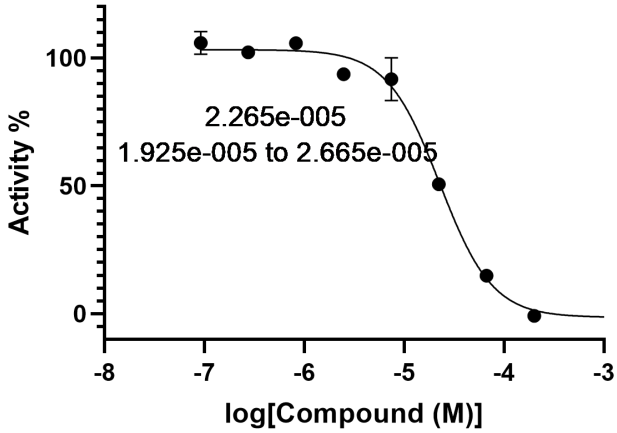

Conc log[M]	%	%
-3.70	-0.8	-0.9
-4.18	14.3	15.5
-4.65	51.7	49.5
-5.13	85.8	97.6
-5.61	95.5	91.8
-6.08	106.9	104.6
-6.56	100.7	103.7
-7.04	102.7	109.0
DMSO	104.0	106.3

Compound 45

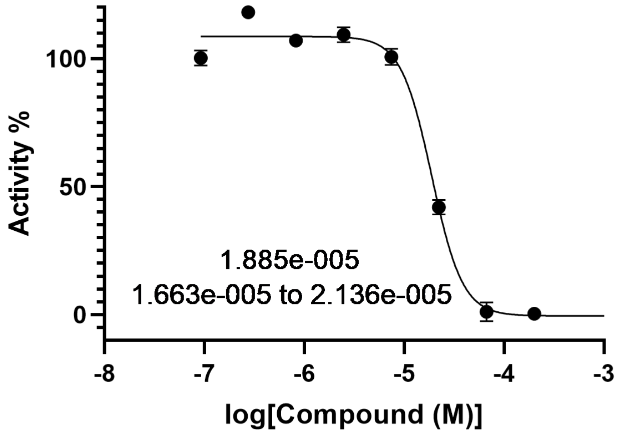

Conc log[M]	%	%
-3.70	-0.2	0.9
-4.18	3.7	-1.6
-4.65	44.0	40.0
-5.13	98.4	102.8
-5.61	107.3	111.4
-6.08	106.9	107.3
-6.56	118.6	117.7
-7.04	102.4	98.2
DMSO	100.2	107.3

Compound 46

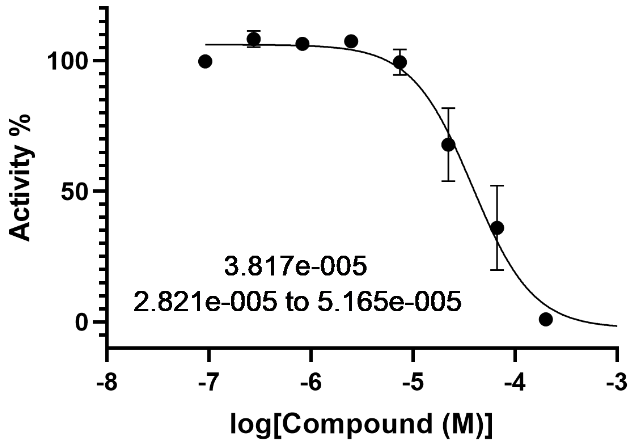

Conc log[M]	%	%
-3.70	0.4	1.5
-4.18	47.5	24.6
-4.65	77.7	57.9
-5.13	102.8	95.9
-5.61	107.4	107.3
-6.08	107.6	105.1
-6.56	110.5	106.0
-7.04	100.9	98.4
DMSO	98.1	103.4

Compound 47

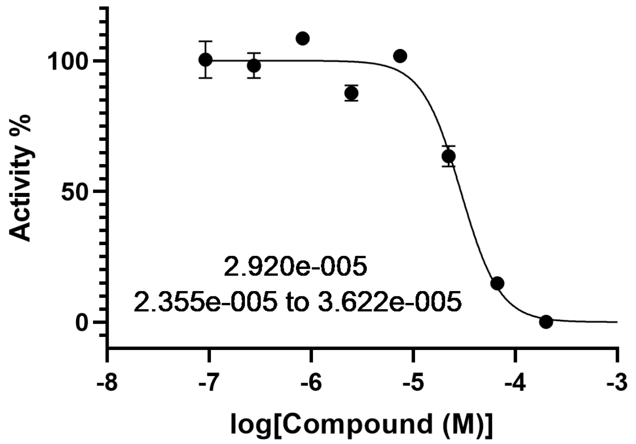

Conc log[M]	%	%
-3.70	0.1	0.3
-4.18	13.0	16.6
-4.65	60.7	66.3
-5.13	101.7	102.1
-5.61	85.6	89.7
-6.08	109.9	107.2
-6.56	94.8	101.6
-7.04	105.4	95.5
DMSO	103.0	97.8

Compound 48

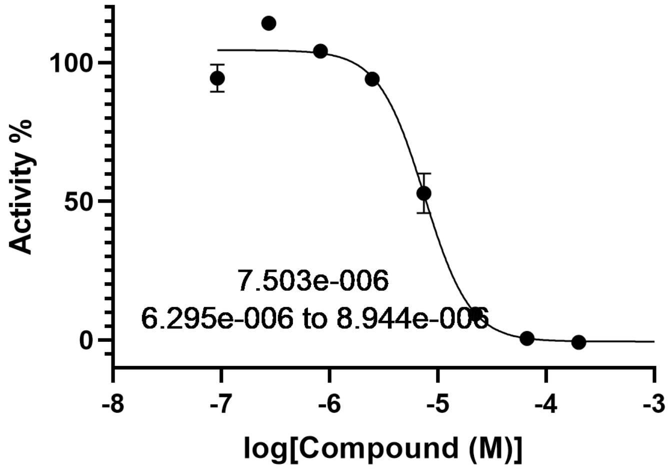

Conc log[M]	%	%
-3.70	-1.3	-0.5
-4.18	0.1	0.9
-4.65	10.6	8.1
-5.13	47.8	58.0
-5.61	95.4	92.6
-6.08	103.9	104.4
-6.56	114.9	113.6
-7.04	90.9	97.9
DMSO	94.3	97.8

Compound 49

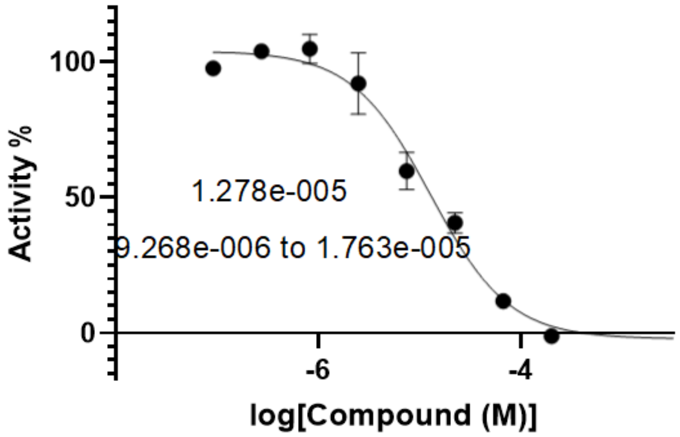

Conc log[M]	%	%
-3.70	-1.1	-1.3
-4.18	10.5	12.8
-4.65	37.9	43.2
-5.13	64.5	54.7
-5.61	100.1	84.0
-6.08	108.5	101.0
-6.56	104.5	103.1
-7.04	99.4	95.8
DMSO	106.7	102.5

Compound 50

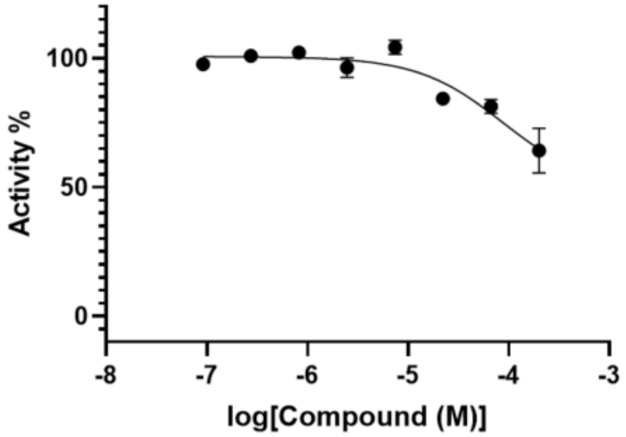

Conc log[M]	%	%
-3.70	58.1	70.3
-4.18	79.3	83.2
-4.65	84.9	83.7
-5.13	102.3	106.1
-5.61	93.6	99.1
-6.08	101.2	103.3
-6.56	101.4	100.3
-7.04	96.3	99.0
DMSO	104.0	106.3

Compound 51

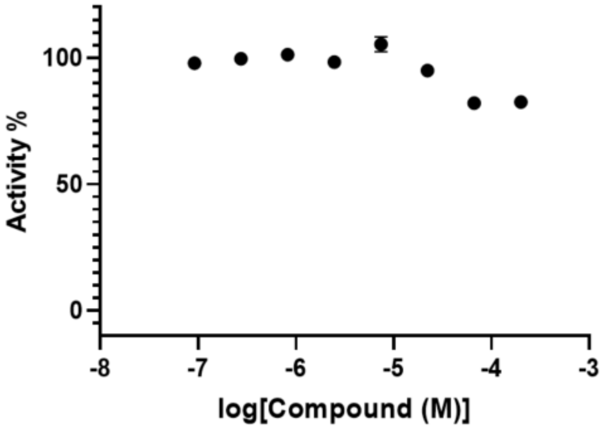

Conc log[M]	%	%
-3.70	82.1	82.8
-4.18	82.9	81.2
-4.65	96.6	93.2
-5.13	107.4	103.1
-5.61	99.6	97.0
-6.08	102.5	99.8
-6.56	99.3	99.8
-7.04	97.8	97.9
DMSO	104.0	106.3

Compound 52

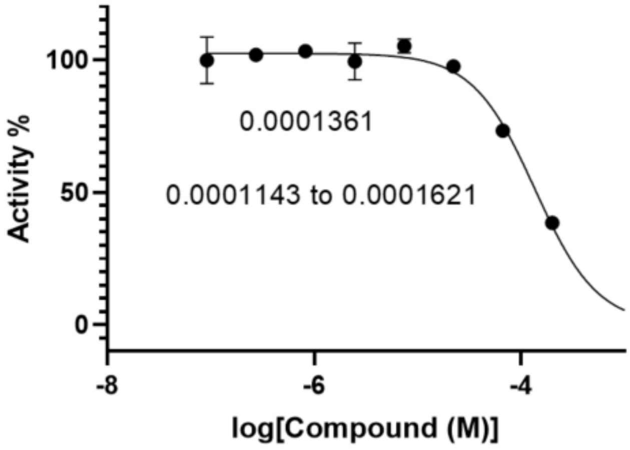

Conc log[M]	%	%
-3.70	37.4	39.4
-4.18	72.0	74.4
-4.65	97.6	97.5
-5.13	107.0	103.3
-5.61	104.3	94.5
-6.08	103.3	103.3
-6.56	101.1	102.6
-7.04	106.0	93.5
DMSO	104.0	106.3

Compound 53

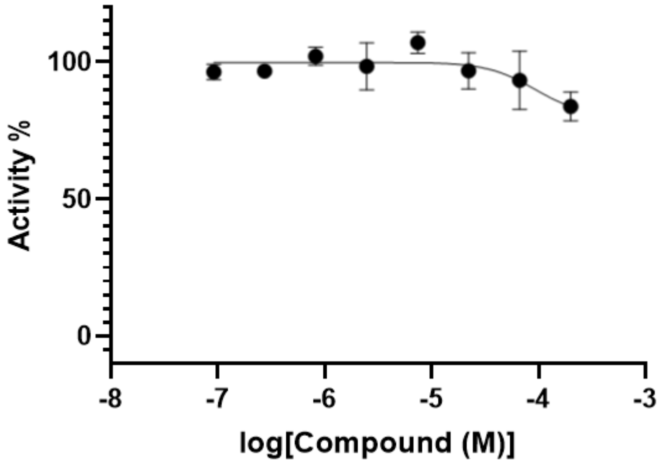

Conc log[M]	%	%
-3.70	80.1	87.5
-4.18	85.8	100.8
-4.65	92.0	101.4
-5.13	104.2	109.8
-5.61	92.3	104.5
-6.08	99.7	104.4
-6.56	95.9	97.5
-7.04	94.4	98.3
DMSO	104.0	106.3

Compound 54

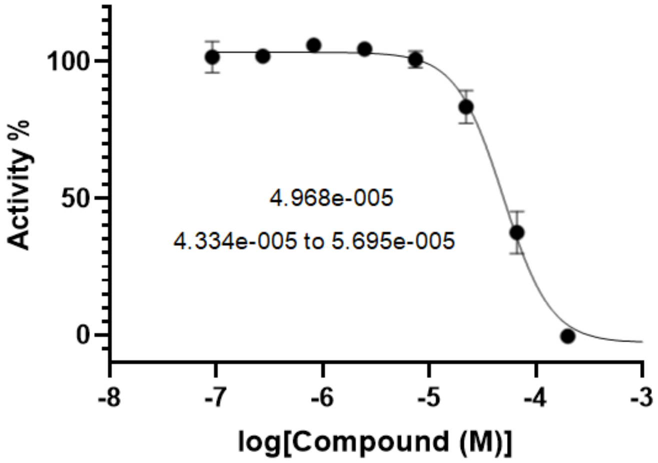

Conc log[M]	%	%
-3.70	-1.7	1.0
-4.18	42.9	32.0
-4.65	87.6	79.2
-5.13	102.9	98.6
-5.61	103.4	105.8
-6.08	105.0	106.8
-6.56	101.2	102.8
-7.04	97.5	105.7
DMSO	102.3	106.7

Compound 55

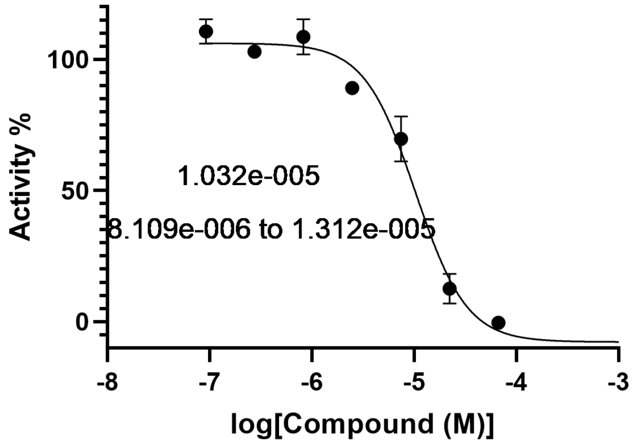

Conc log[M]	%	%
-3.70	-16.1	-16.3
-4.18	-0.2	-0.6
-4.65	8.6	16.6
-5.13	75.7	63.6
-5.61	88.7	89.5
-6.08	103.9	113.3
-6.56	104.5	101.3
-7.04	113.9	107.2
DMSO	106.3	100.8

Compound 56

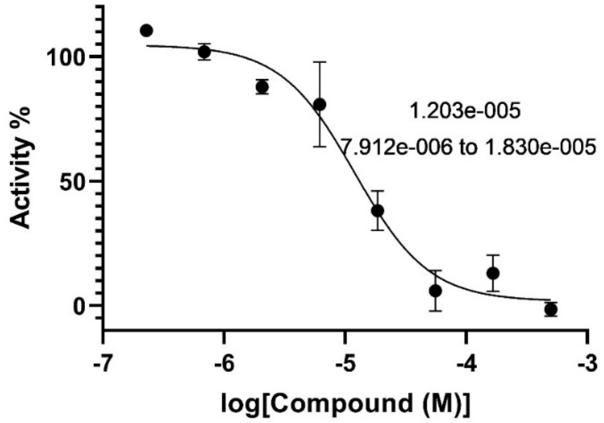

Conc log[M]	%	%
-3.30	-3.4	0.4
-3.78	7.8	18.2
-4.26	0.2	11.8
-4.73	43.7	32.5
-5.21	68.8	92.8
-5.69	85.9	89.9
-6.16	104.3	99.7
-6.64	111.4	109.6
DMSO	97.9	100.4

Compound 57

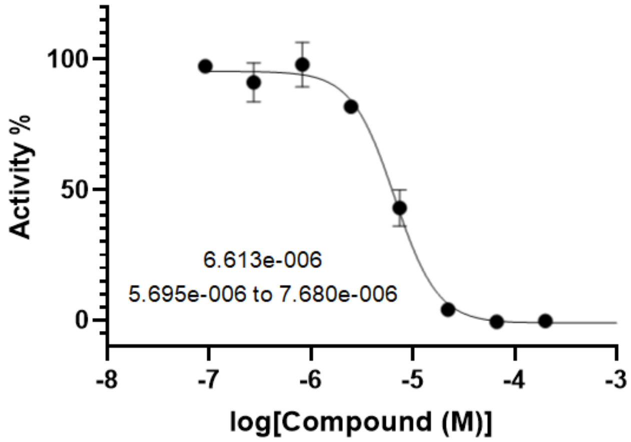

Conc log[M]	%	%
-3.70	1.1	-1.7
-4.18	-0.4	-0.8
-4.65	4.1	3.9
-5.13	47.9	38.1
-5.61	80.4	83.2
-6.08	104.1	91.9
-6.56	96.4	85.8
-7.04	98.6	95.9
DMSO	102.3	106.7

Compound 7

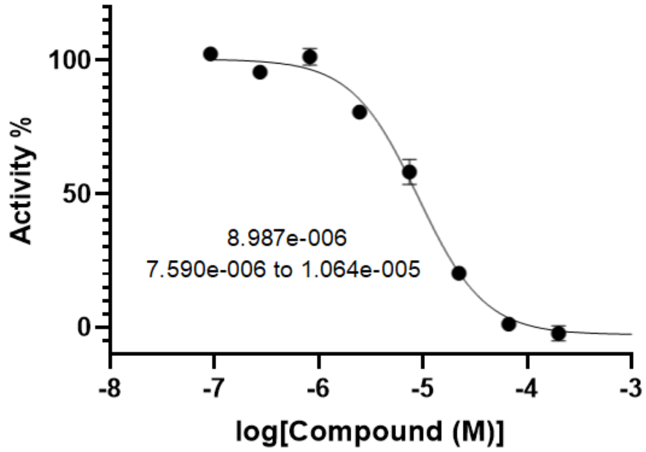

Conc log[M]	%	%
-3.70	-4.3	-0.3
-4.18	1.0	1.4
-4.65	21.4	19.2
-5.13	61.5	54.8
-5.61	81.8	79.5
-6.08	99.1	103.5
-6.56	95.8	95.4
-7.04	102.6	102.1
DMSO	97.3	102.0

SI compounds

Compound S1

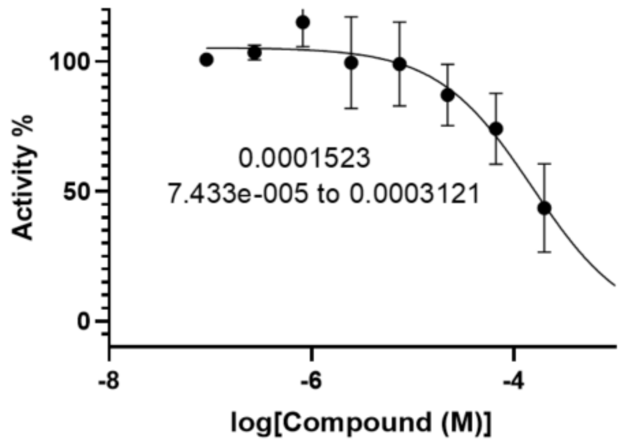

Conc log[M]	%	%
-3.70	55.6	31.4
-4.18	83.8	64.5
-4.65	95.6	78.8
-5.13	110.5	87.6
-5.61	112.1	87.0
-6.08	108.5	121.8
-6.56	101.5	105.5
-7.04	102.0	99.5
DMSO	103.2	99.7

Compound S2

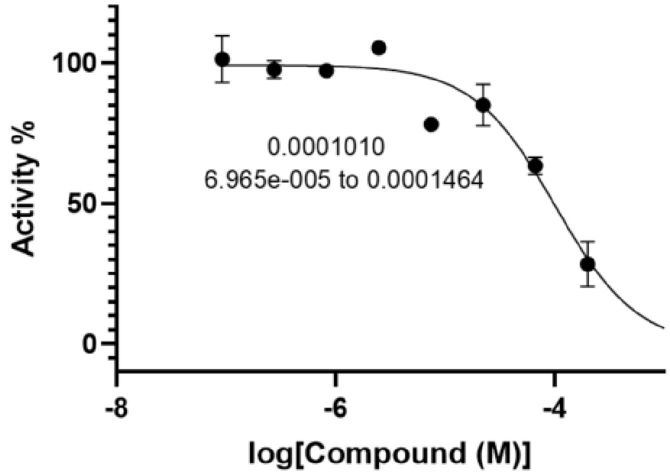

Conc log[M]	%	%
-3.70	34.1	22.8
-4.18	61.2	65.6
-4.65	79.7	90.2
-5.13	30.2	78.1
-5.61	105.7	104.8
-6.08	95.5	99.0
-6.56	95.4	99.8
-7.04	95.4	107.3
DMSO	103.2	99.7

data point omitted

Compound S3

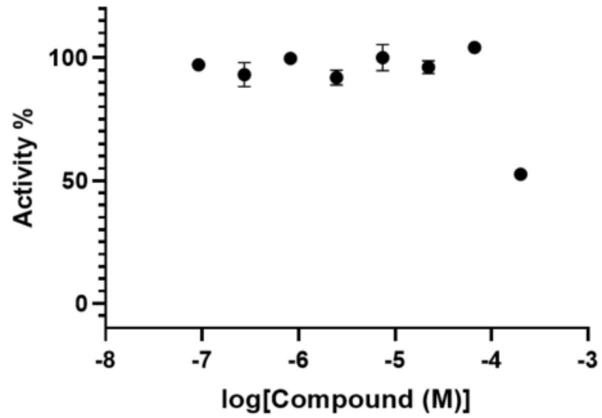

Conc log[M]	%	%
-3.70	51.4	53.7
-4.18	102.7	105.5
-4.65	97.9	94.2
-5.13	96.3	103.8
-5.61	94.1	89.7
-6.08	99.1	100.3
-6.56	89.6	96.6
-7.04	96.9	97.2
DMSO	98.4	101.2

Compound S4

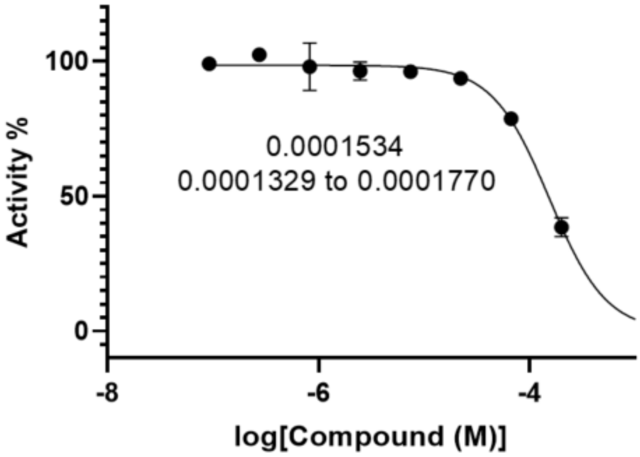

Conc log[M]	%	%
-3.70	41.0	36.0
-4.18	77.5	79.9
-4.65	95.2	92.1
-5.13	97.4	94.7
-5.61	98.7	94.0
-6.08	91.7	104.1
-6.56	104.0	100.7
-7.04	100.7	97.4
DMSO	98.4	101.2

Compound S5

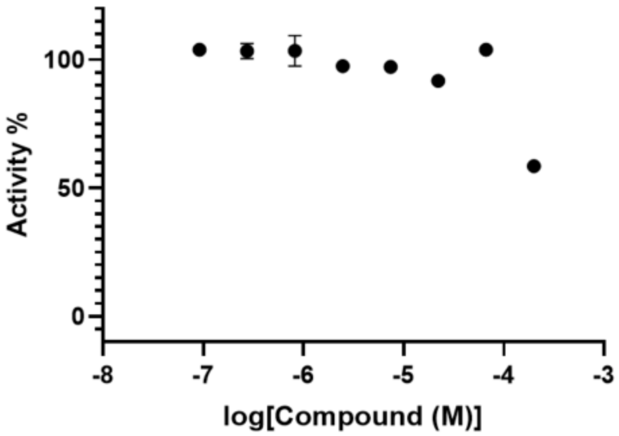

Conc log[M]	%	%
-3.70	57.6	59.4
-4.18	102.7	105.1
-4.65	93.5	90.1
-5.13	96.5	97.8
-5.61	98.2	96.8
-6.08	107.7	99.2
-6.56	105.5	101.3
-7.04	104.7	103.0
DMSO	98.4	101.2

Compound S6

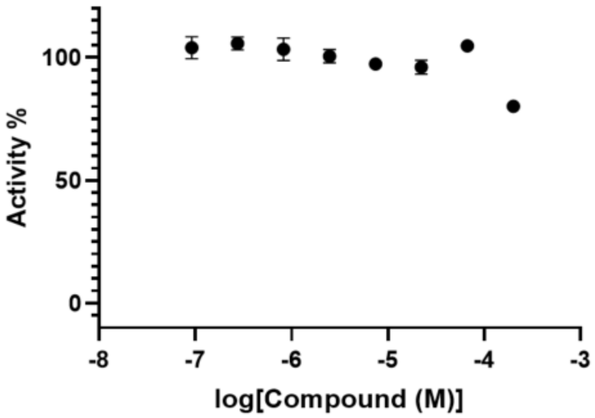

Conc log[M]	%	%
-3.70	78.6	81.5
-4.18	105.9	103.5
-4.65	98.0	94.0
-5.13	99.1	95.6
-5.61	102.4	98.6
-6.08	106.5	100.1
-6.56	107.4	103.8
-7.04	107.1	100.8
DMSO	98.4	101.2

Compound S7

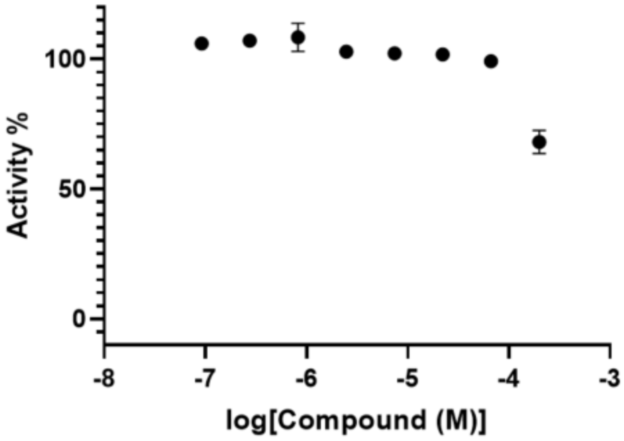

Conc log[M]	%	%
-3.70	64.8	71.2
-4.18	100.2	98.0
-4.65	99.9	103.5
-5.13	101.8	102.5
-5.61	104.2	101.5
-6.08	112.2	104.4
-6.56	107.2	107.0
-7.04	105.7	106.3
DMSO	98.4	101.2

3.2.2.SENP1-SUMO2-AMC

Compound 11

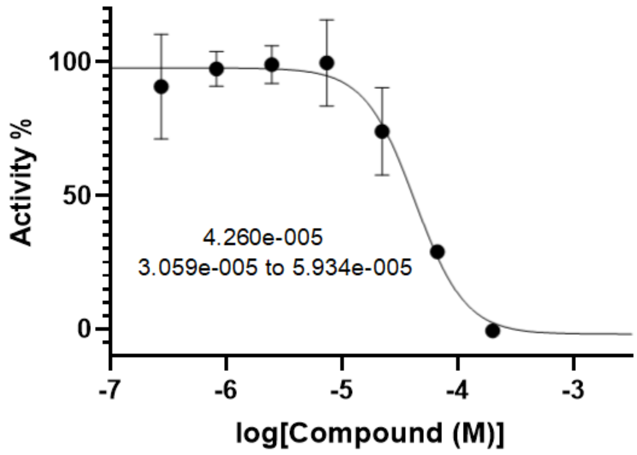

Conc log[M]	%	%
-3.70	-1.7	0.5
-4.18	30.6	27.2
-4.65	62.4	85.6
-5.13	88.3	111.1
-5.61	94.1	104.0
-6.08	102.1	92.8
-6.56	104.7	76.9
-7.04	102.9	97.5
DMSO	97.3	103.7

Compound 55

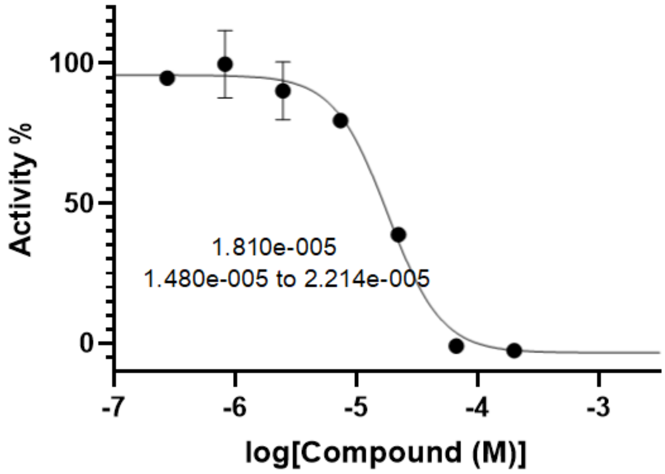

Conc log[M]	%	%
-3.70	-3.7	-1.5
-4.18	-0.8	-1.2
-4.65	38.6	39.0
-5.13	78.2	80.7
-5.61	82.9	97.4
-6.08	91.1	108.2
-6.56	93.1	96.3
-7.04	103.6	90.6
DMSO	95.3	98.7

Compound 7

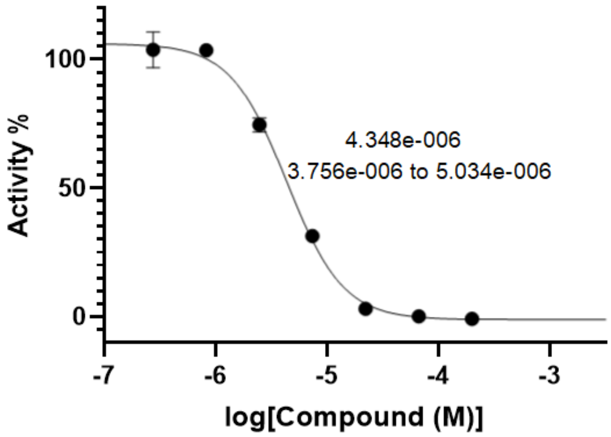

Conc log[M]	%	%
-3.82	-3.7	-4.7
-4.30	53.3	43.5
-4.78	79.7	75.7
-5.26	88.3	82.0
-5.73	100.2	100.7
-6.21	103.6	100.4
-6.69	104.4	105.8
-7.16	107.7	108.0
DMSO	97.3	97.3

3.2.3.SENP1-SUMO3-AMC

4. Compound 11

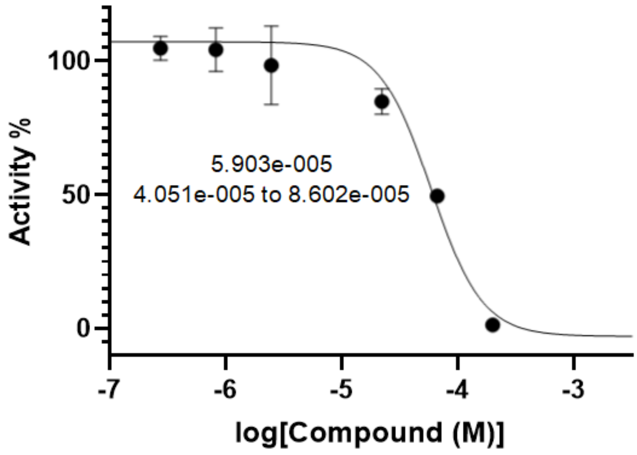

Conc log[M]	%	%
-3.70	0.4	2.2
-4.18	48.4	50.8
-4.65	81.5	88.2
-5.13	139.1	109.5
-5.61	88.0	108.8
-6.08	98.5	110.0
-6.56	107.9	101.6
-7.04	116.6	99.6
DMSO	105.4	109.3

Compound 55

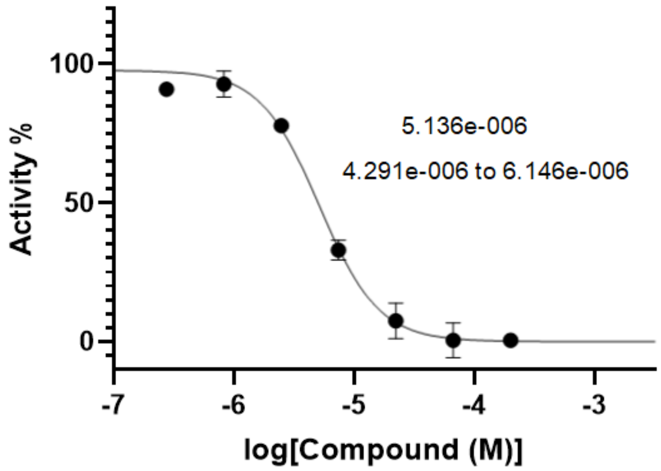

Conc log[M]	%	%
-3.70	0.0	1.0
-4.18	5.0	-4.0
-4.65	12.0	3.0
-5.13	30.4	35.5
-5.61	79.5	76.1
-6.08	89.4	96.1
-6.56	89.6	92.2
-7.04	101.2	107.4
DMSO	102.4	96.9

Compound 7

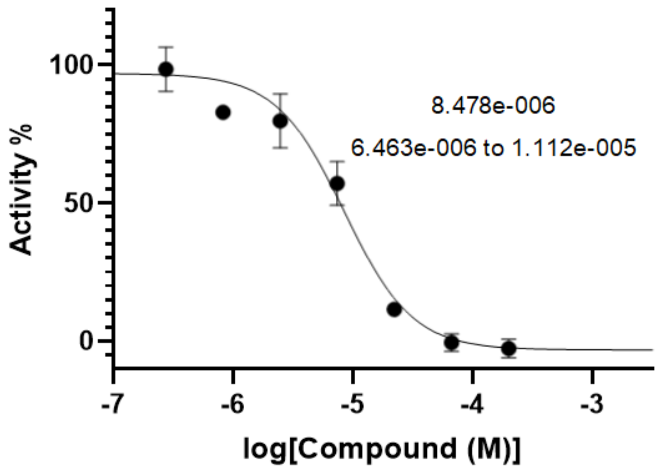

Conc log[M]	%	%
-3.70	-0.3	-5.0
-4.18	1.7	-2.8
-4.65	11.6	11.4
-5.13	62.7	51.4
-5.61	86.7	72.8
-6.08	137.2	82.9
-6.56	104.3	92.8
-7.04	100.4	103.2
DMSO	97.7	100.4

data point omitted

4.1.1.UCHL1

Compound 11

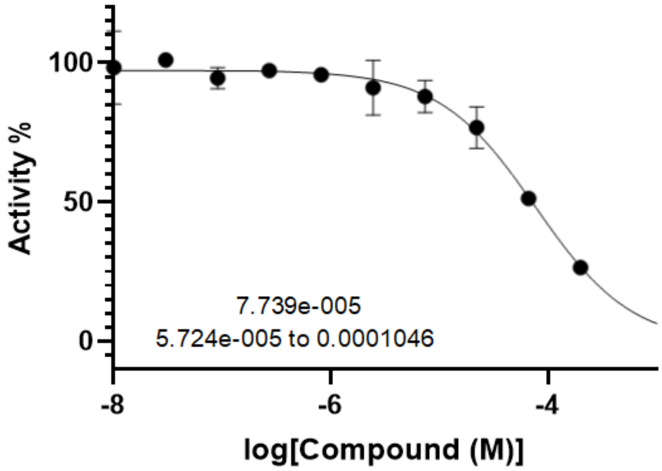

Conc log[M]	%	%
-3.70	26.2	26.7
-4.18	52.4	50.2
-4.65	82.0	71.4
-5.13	83.7	91.9
-5.61	98.0	84.1
-6.08	96.6	94.7
-6.56	98.9	95.5
-7.04	97.2	91.8
-7.52	102.23	99.83
-7.99	107.66	89.00

Compound 55

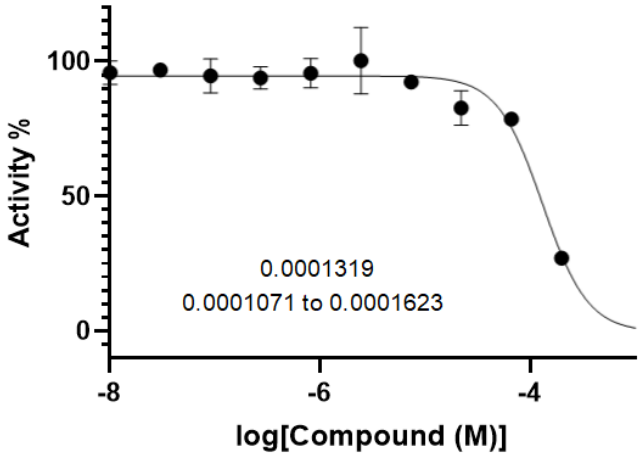

Conc log[M]	%	%
-3.70	26.4	27.6
-4.18	79.9	77.2
-4.65	87.2	78.2
-5.13	92.3	92.2
-5.61	91.5	109.0
-6.08	91.7	99.4
-6.56	90.9	96.7
-7.04	90.1	99.0
-7.52	97.70	95.80
-7.99	98.83	90.57

Compound 7

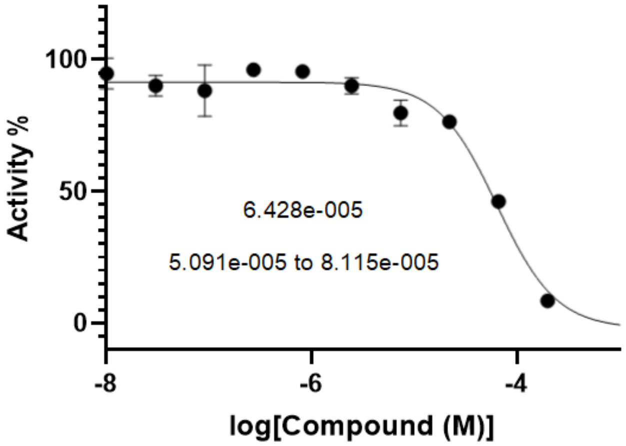

Conc log[M]	%	%
-3.70	7.1	9.8
-4.18	46.1	46.3
-4.65	75.3	77.6
-5.13	83.2	76.3
-5.61	92.3	87.9
-6.08	96.4	94.5
-6.56	95.3	96.8
-7.04	81.3	95.1
-7.52	87.29	92.89
-7.99	98.83	90.57

4.1.2. Ataxin-3

Compound 11

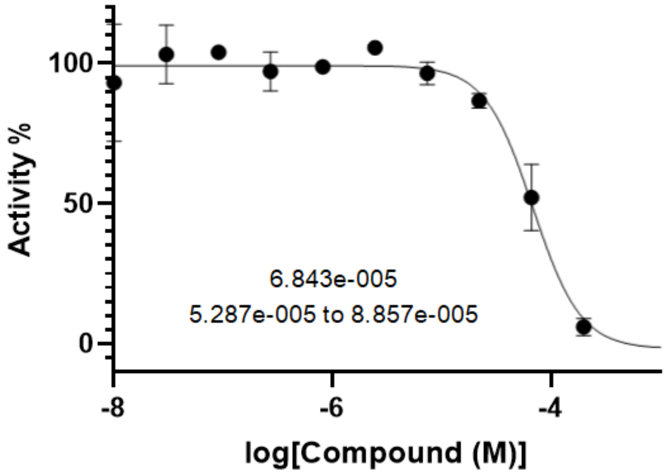

Conc log[M]	%	%
-3.70	8.1	3.9
-4.18	60.5	43.7
-4.65	88.5	84.7
-5.13	99.2	93.6
-5.61	105.5	
-6.08	98.1	99.3
-6.56	102.1	92.2
-7.04	104.8	102.9
-7.52	110.5	95.8
-7.99	78.3	107.8

Compound 55

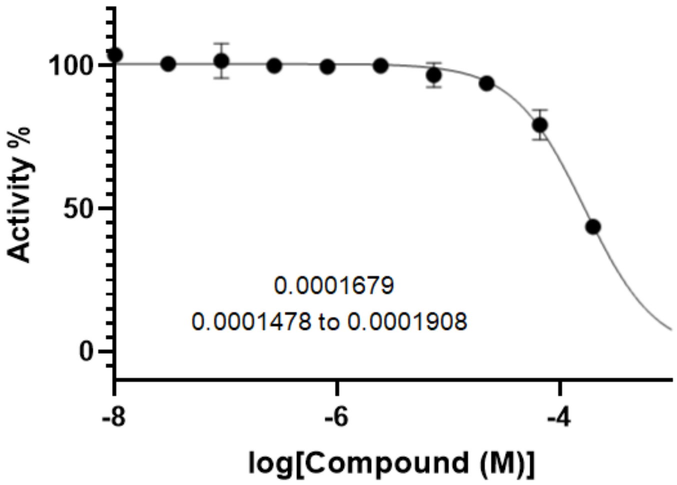

Conc log[M]	%	%
-3.70	44.5	42.8
-4.18	75.7	83.0
-4.65	92.8	94.8
-5.13	93.8	99.8
-5.61	98.5	101.6
-6.08	98.8	100.6
-6.56	100.1	99.9
-7.04	97.4	106.1
-7.52	102.2	99.1
-7.99	103.0	104.5

Compound 7

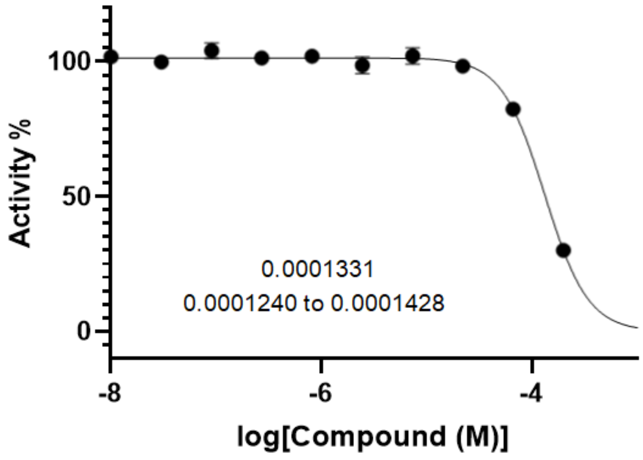

Conc log[M]	%	%
-3.70	31.1	28.9
-4.18	82.8	82.0
-4.65	98.3	
-5.13	104.2	99.8
-5.61	100.8	96.3
-6.08	103.1	100.9
-6.56	102.3	100.4
-7.04	106.1	102.0
-7.52	99.2	100.4
-7.99	103.0	100.5
